# Supplementary figures and images for: Adaptation to glucose starvation is associated with molecular reorganization of the circadian clock in Neurospora crassa
Source: eLife. 2023 Jan 10;12:e79765. doi: 10.7554/eLife.79765 (PMC9831608; doi:10.7554/eLife.79765)

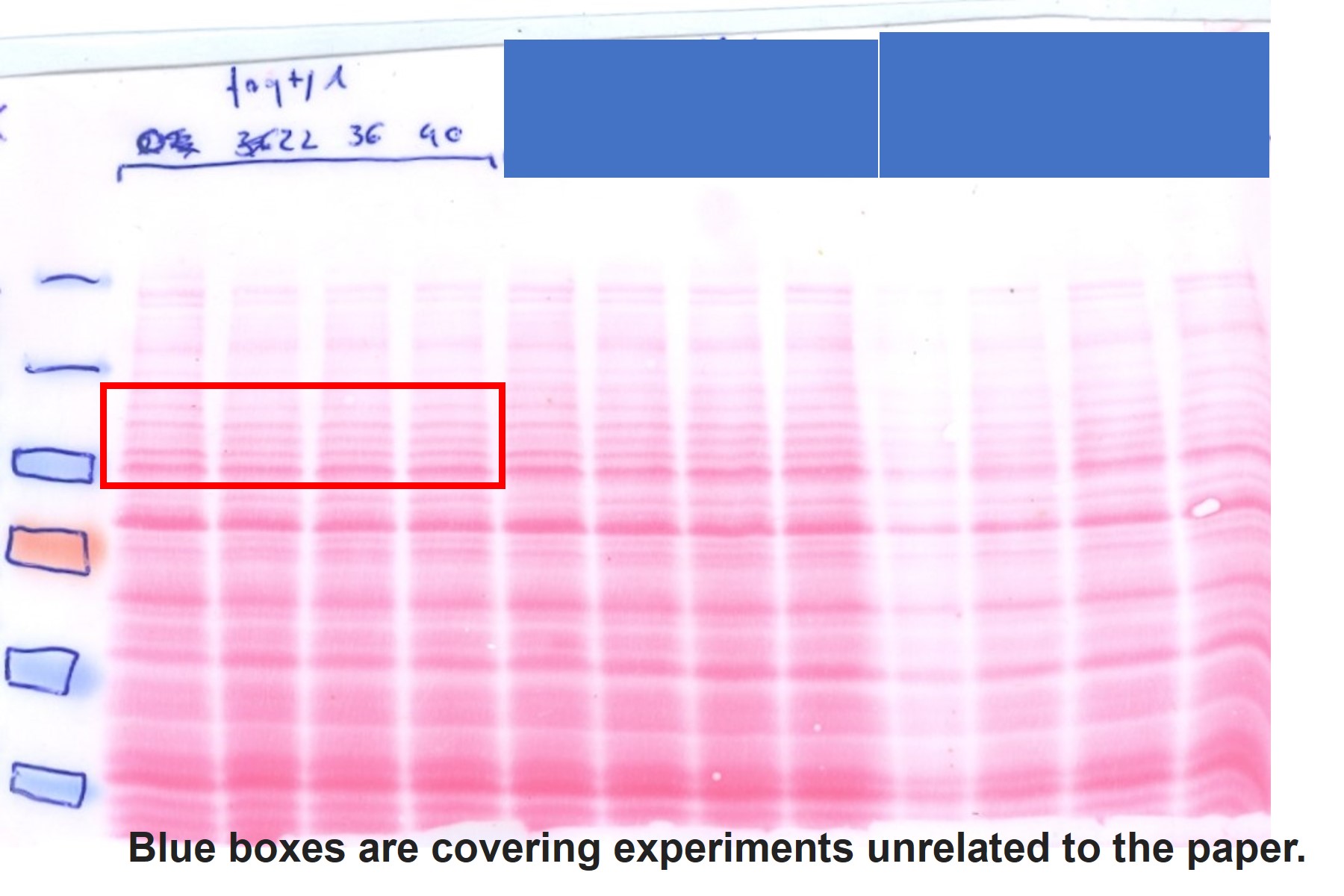

Supplement: Figure 1—source data 1. — (a) Western blots were used to detect expression of FRQ in the indicated samples for Figure 1B. (b) Figure with the area highlighted was used to develop the Figure 1B for FRQ. (c) Western blots were used to detect expression of WC-1 in the indicated samples for Figure 1B. (d) Figure with the area highlighted was used to develop the Figure 1B for WC-1. (e) Western blots were used to detect expression of WC-2 in the indicated samples for Figure 1B. (f) Figure with the area highlighted was used to develop the Figure 1B for WC-2. (g) Western blots were used to detect expression of RGB1 in the indicated samples for Figure 1B. (h) Figure with the area highlighted was used to develop the Figure 1B for RGB1. (i) Ponceau S staining was used to detect loading control of the indicated samples for Figure 1B. (j) Figure with the area highlighted was used for LC of Figure 1B . [file elife-79765-fig1-data1.zip › 79765Figure1B/Figure_1-source_data_1j.jpg]

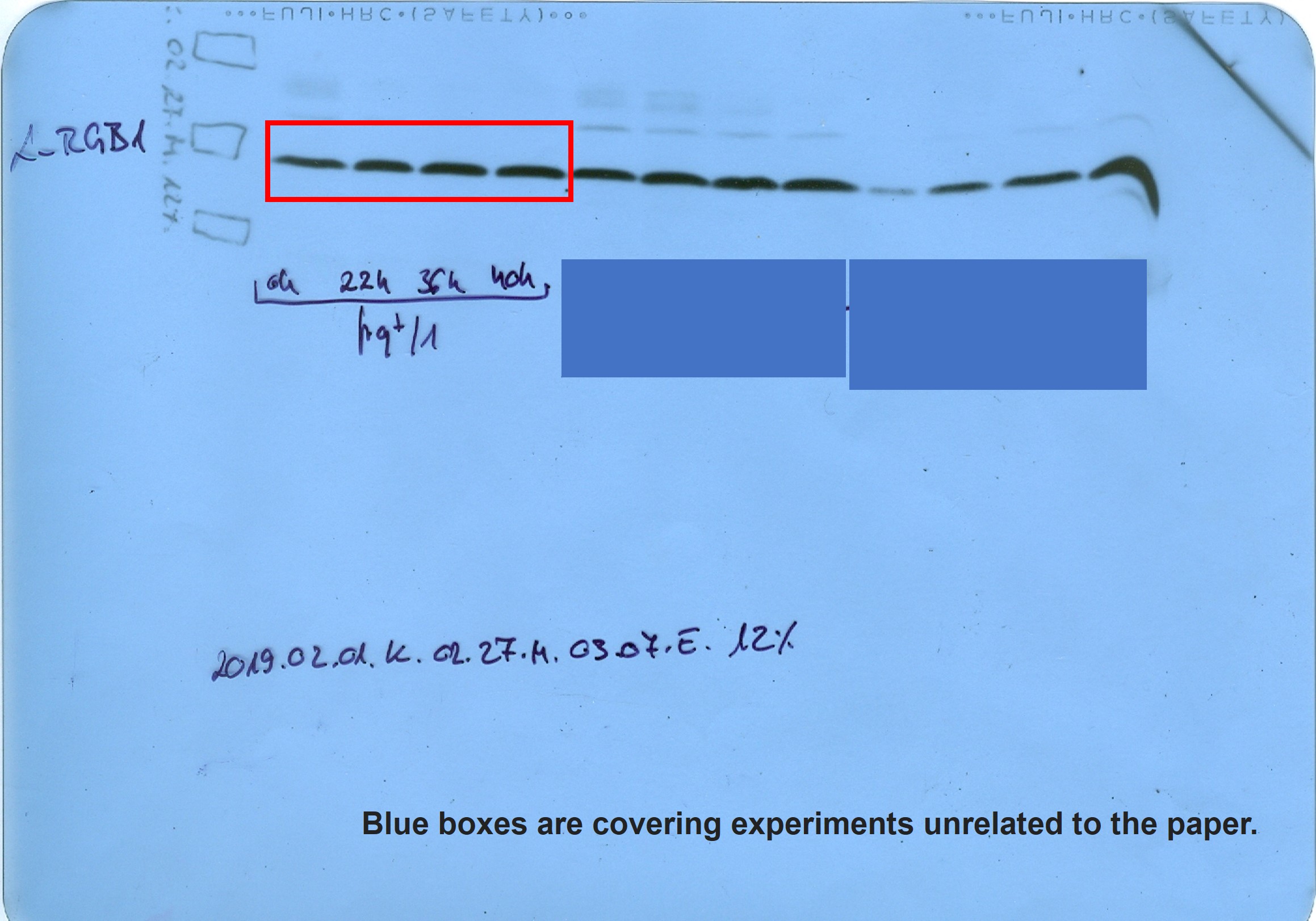

Supplement: Figure 1—source data 1. — (a) Western blots were used to detect expression of FRQ in the indicated samples for Figure 1B. (b) Figure with the area highlighted was used to develop the Figure 1B for FRQ. (c) Western blots were used to detect expression of WC-1 in the indicated samples for Figure 1B. (d) Figure with the area highlighted was used to develop the Figure 1B for WC-1. (e) Western blots were used to detect expression of WC-2 in the indicated samples for Figure 1B. (f) Figure with the area highlighted was used to develop the Figure 1B for WC-2. (g) Western blots were used to detect expression of RGB1 in the indicated samples for Figure 1B. (h) Figure with the area highlighted was used to develop the Figure 1B for RGB1. (i) Ponceau S staining was used to detect loading control of the indicated samples for Figure 1B. (j) Figure with the area highlighted was used for LC of Figure 1B . [file elife-79765-fig1-data1.zip › 79765Figure1B/Figure_1-source_data_1h.jpg]

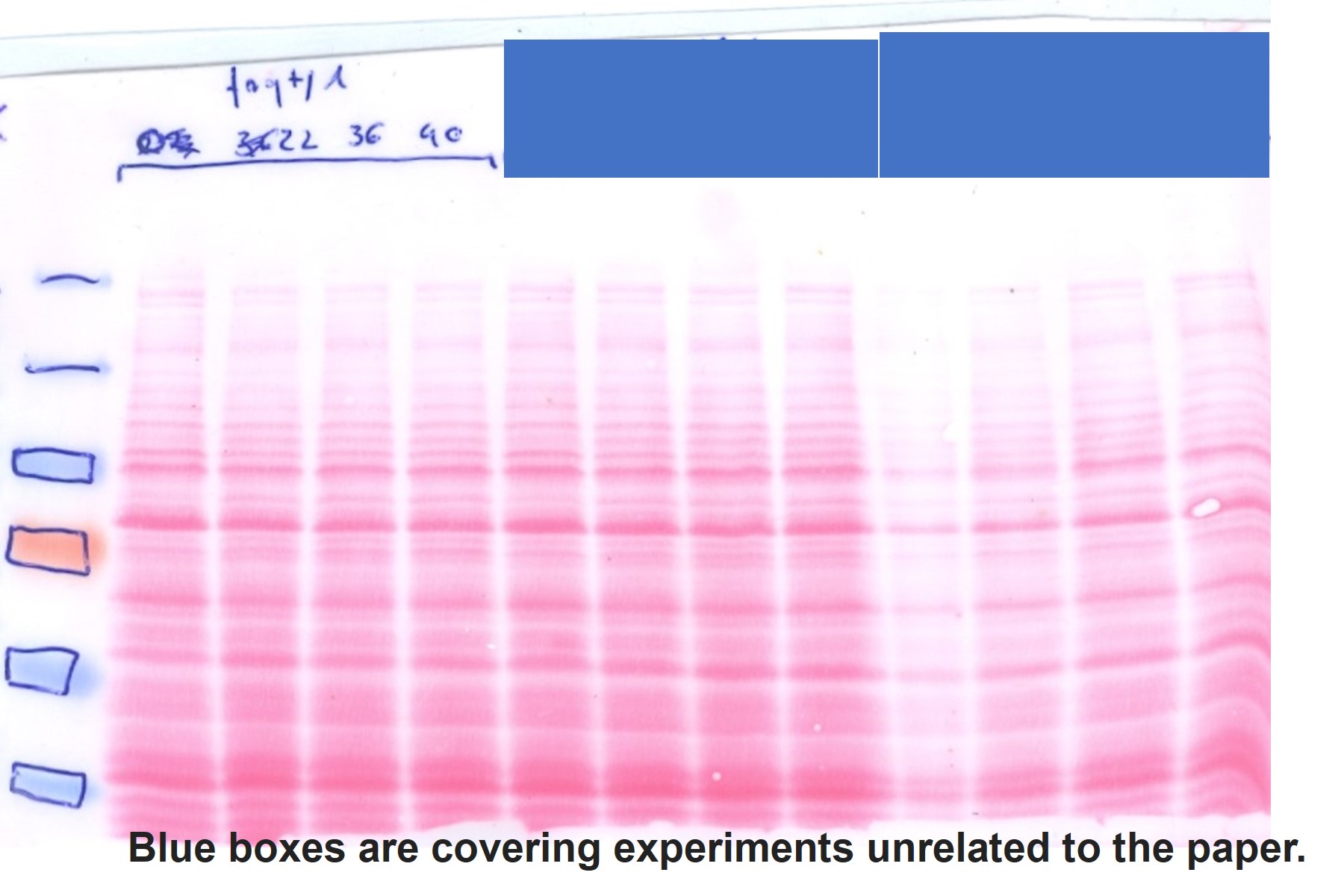

Supplement: Figure 1—source data 1. — (a) Western blots were used to detect expression of FRQ in the indicated samples for Figure 1B. (b) Figure with the area highlighted was used to develop the Figure 1B for FRQ. (c) Western blots were used to detect expression of WC-1 in the indicated samples for Figure 1B. (d) Figure with the area highlighted was used to develop the Figure 1B for WC-1. (e) Western blots were used to detect expression of WC-2 in the indicated samples for Figure 1B. (f) Figure with the area highlighted was used to develop the Figure 1B for WC-2. (g) Western blots were used to detect expression of RGB1 in the indicated samples for Figure 1B. (h) Figure with the area highlighted was used to develop the Figure 1B for RGB1. (i) Ponceau S staining was used to detect loading control of the indicated samples for Figure 1B. (j) Figure with the area highlighted was used for LC of Figure 1B . [file elife-79765-fig1-data1.zip › 79765Figure1B/Figure_1-source_data_1i.jpg]

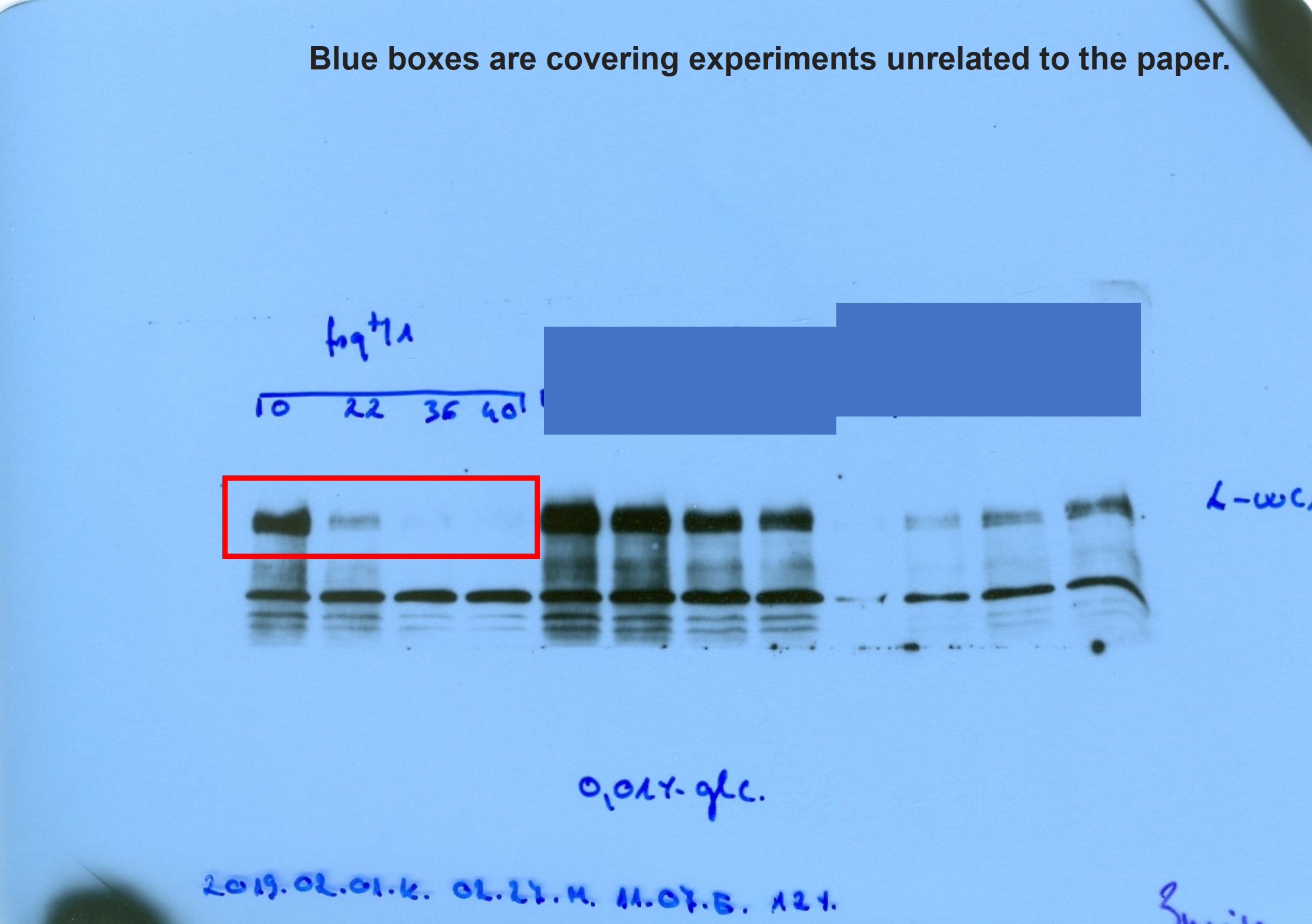

Supplement: Figure 1—source data 1. — (a) Western blots were used to detect expression of FRQ in the indicated samples for Figure 1B. (b) Figure with the area highlighted was used to develop the Figure 1B for FRQ. (c) Western blots were used to detect expression of WC-1 in the indicated samples for Figure 1B. (d) Figure with the area highlighted was used to develop the Figure 1B for WC-1. (e) Western blots were used to detect expression of WC-2 in the indicated samples for Figure 1B. (f) Figure with the area highlighted was used to develop the Figure 1B for WC-2. (g) Western blots were used to detect expression of RGB1 in the indicated samples for Figure 1B. (h) Figure with the area highlighted was used to develop the Figure 1B for RGB1. (i) Ponceau S staining was used to detect loading control of the indicated samples for Figure 1B. (j) Figure with the area highlighted was used for LC of Figure 1B . [file elife-79765-fig1-data1.zip › 79765Figure1B/Figure_1-source_data_1d.jpg]

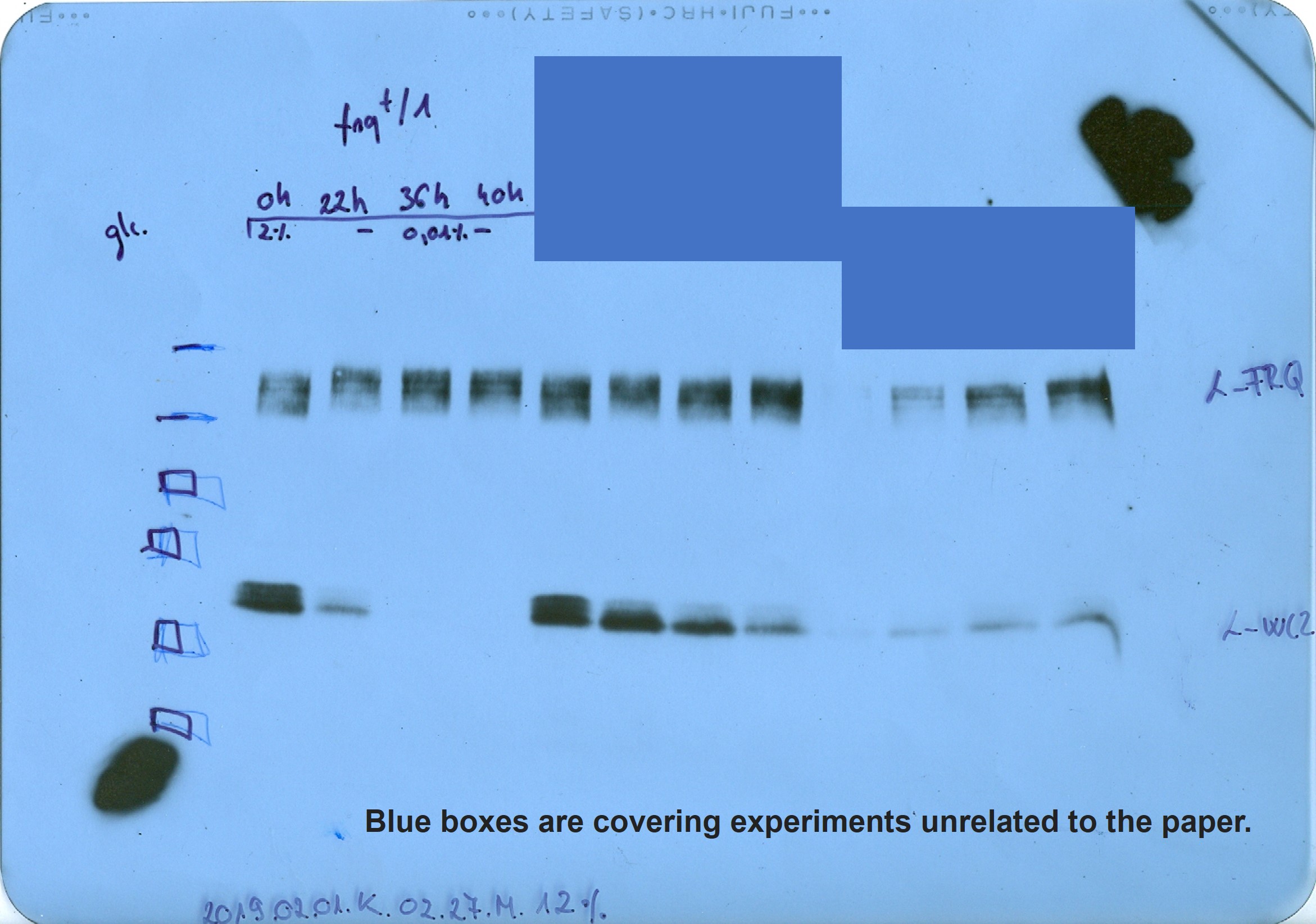

Supplement: Figure 1—source data 1. — (a) Western blots were used to detect expression of FRQ in the indicated samples for Figure 1B. (b) Figure with the area highlighted was used to develop the Figure 1B for FRQ. (c) Western blots were used to detect expression of WC-1 in the indicated samples for Figure 1B. (d) Figure with the area highlighted was used to develop the Figure 1B for WC-1. (e) Western blots were used to detect expression of WC-2 in the indicated samples for Figure 1B. (f) Figure with the area highlighted was used to develop the Figure 1B for WC-2. (g) Western blots were used to detect expression of RGB1 in the indicated samples for Figure 1B. (h) Figure with the area highlighted was used to develop the Figure 1B for RGB1. (i) Ponceau S staining was used to detect loading control of the indicated samples for Figure 1B. (j) Figure with the area highlighted was used for LC of Figure 1B . [file elife-79765-fig1-data1.zip › 79765Figure1B/Figure_1-source_data_1e.jpg]

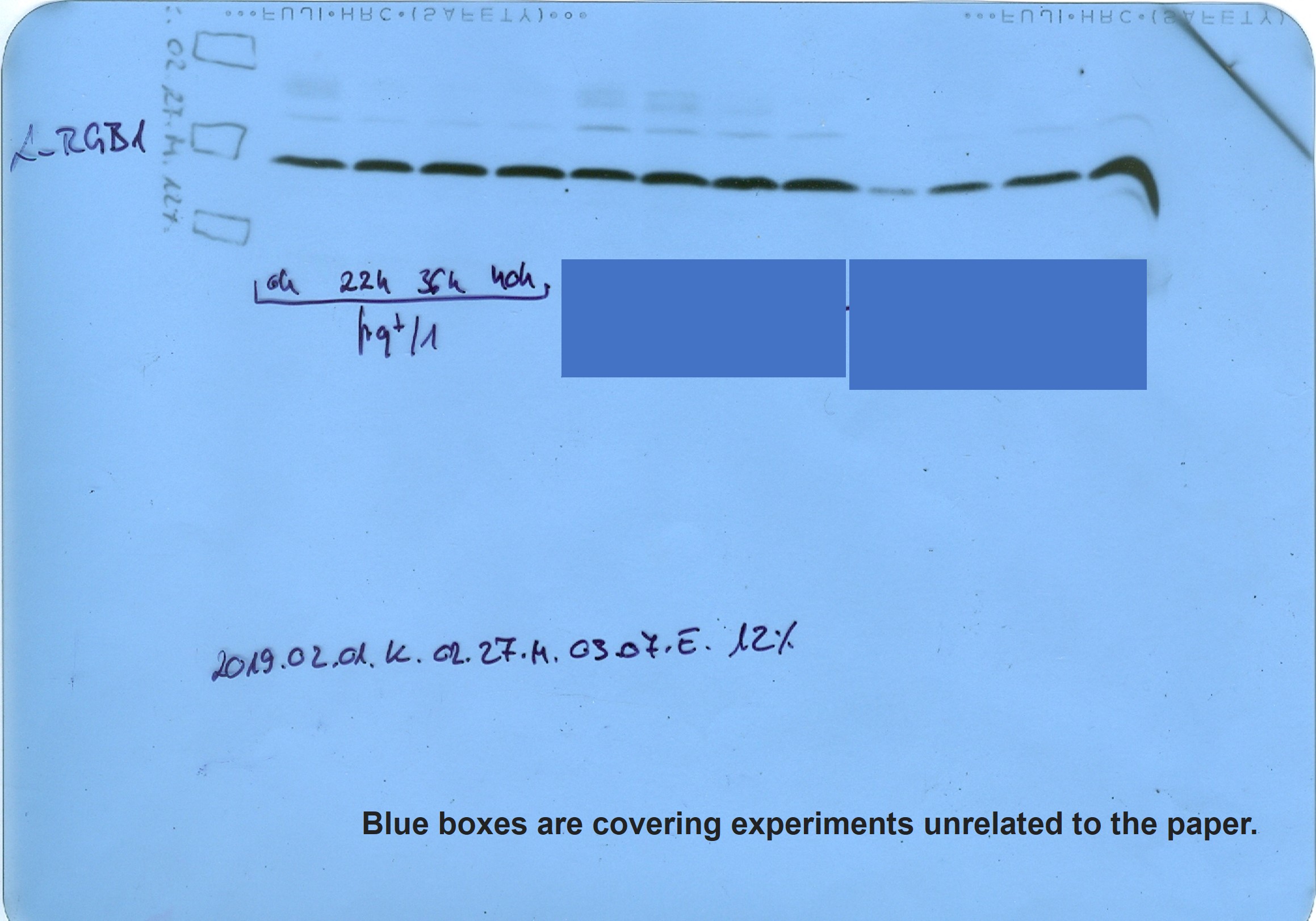

Supplement: Figure 1—source data 1. — (a) Western blots were used to detect expression of FRQ in the indicated samples for Figure 1B. (b) Figure with the area highlighted was used to develop the Figure 1B for FRQ. (c) Western blots were used to detect expression of WC-1 in the indicated samples for Figure 1B. (d) Figure with the area highlighted was used to develop the Figure 1B for WC-1. (e) Western blots were used to detect expression of WC-2 in the indicated samples for Figure 1B. (f) Figure with the area highlighted was used to develop the Figure 1B for WC-2. (g) Western blots were used to detect expression of RGB1 in the indicated samples for Figure 1B. (h) Figure with the area highlighted was used to develop the Figure 1B for RGB1. (i) Ponceau S staining was used to detect loading control of the indicated samples for Figure 1B. (j) Figure with the area highlighted was used for LC of Figure 1B . [file elife-79765-fig1-data1.zip › 79765Figure1B/Figure_1-source_data_1g.jpg]

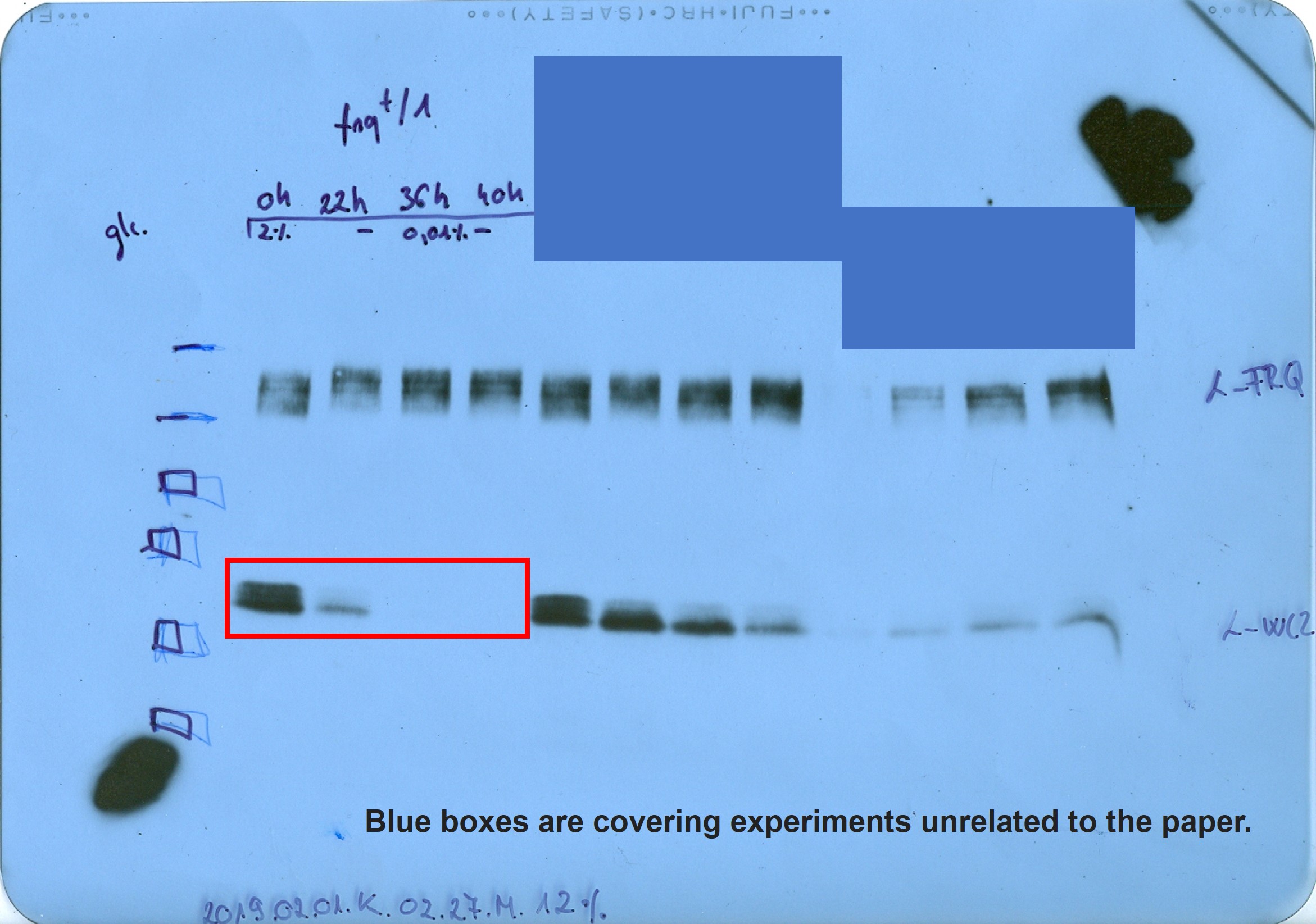

Supplement: Figure 1—source data 1. — (a) Western blots were used to detect expression of FRQ in the indicated samples for Figure 1B. (b) Figure with the area highlighted was used to develop the Figure 1B for FRQ. (c) Western blots were used to detect expression of WC-1 in the indicated samples for Figure 1B. (d) Figure with the area highlighted was used to develop the Figure 1B for WC-1. (e) Western blots were used to detect expression of WC-2 in the indicated samples for Figure 1B. (f) Figure with the area highlighted was used to develop the Figure 1B for WC-2. (g) Western blots were used to detect expression of RGB1 in the indicated samples for Figure 1B. (h) Figure with the area highlighted was used to develop the Figure 1B for RGB1. (i) Ponceau S staining was used to detect loading control of the indicated samples for Figure 1B. (j) Figure with the area highlighted was used for LC of Figure 1B . [file elife-79765-fig1-data1.zip › 79765Figure1B/Figure_1-source_data_1f.jpg]

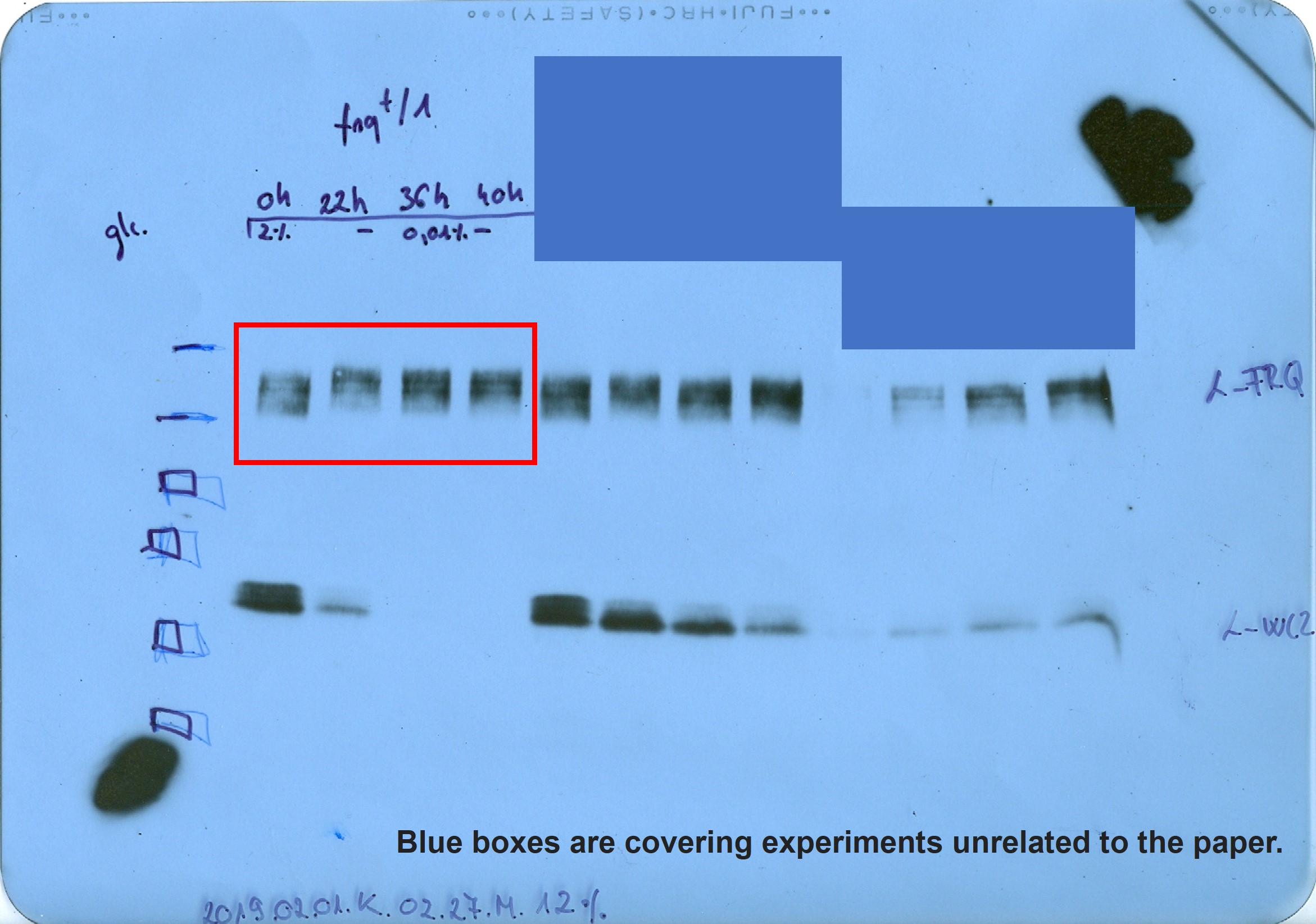

Supplement: Figure 1—source data 1. — (a) Western blots were used to detect expression of FRQ in the indicated samples for Figure 1B. (b) Figure with the area highlighted was used to develop the Figure 1B for FRQ. (c) Western blots were used to detect expression of WC-1 in the indicated samples for Figure 1B. (d) Figure with the area highlighted was used to develop the Figure 1B for WC-1. (e) Western blots were used to detect expression of WC-2 in the indicated samples for Figure 1B. (f) Figure with the area highlighted was used to develop the Figure 1B for WC-2. (g) Western blots were used to detect expression of RGB1 in the indicated samples for Figure 1B. (h) Figure with the area highlighted was used to develop the Figure 1B for RGB1. (i) Ponceau S staining was used to detect loading control of the indicated samples for Figure 1B. (j) Figure with the area highlighted was used for LC of Figure 1B . [file elife-79765-fig1-data1.zip › 79765Figure1B/Figure_1-source_data_1b.jpg]

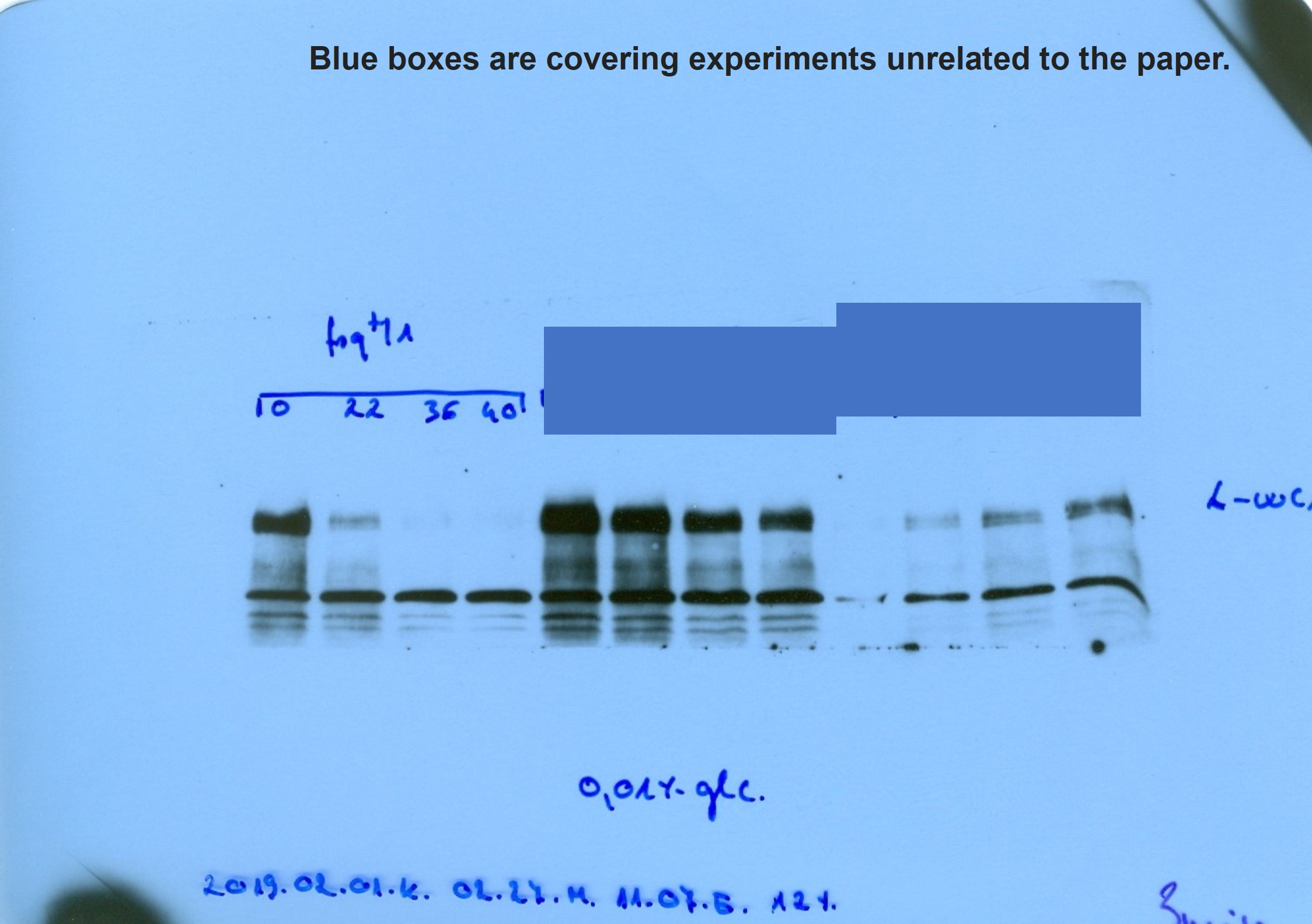

Supplement: Figure 1—source data 1. — (a) Western blots were used to detect expression of FRQ in the indicated samples for Figure 1B. (b) Figure with the area highlighted was used to develop the Figure 1B for FRQ. (c) Western blots were used to detect expression of WC-1 in the indicated samples for Figure 1B. (d) Figure with the area highlighted was used to develop the Figure 1B for WC-1. (e) Western blots were used to detect expression of WC-2 in the indicated samples for Figure 1B. (f) Figure with the area highlighted was used to develop the Figure 1B for WC-2. (g) Western blots were used to detect expression of RGB1 in the indicated samples for Figure 1B. (h) Figure with the area highlighted was used to develop the Figure 1B for RGB1. (i) Ponceau S staining was used to detect loading control of the indicated samples for Figure 1B. (j) Figure with the area highlighted was used for LC of Figure 1B . [file elife-79765-fig1-data1.zip › 79765Figure1B/Figure_1-source_data_1c.jpg]

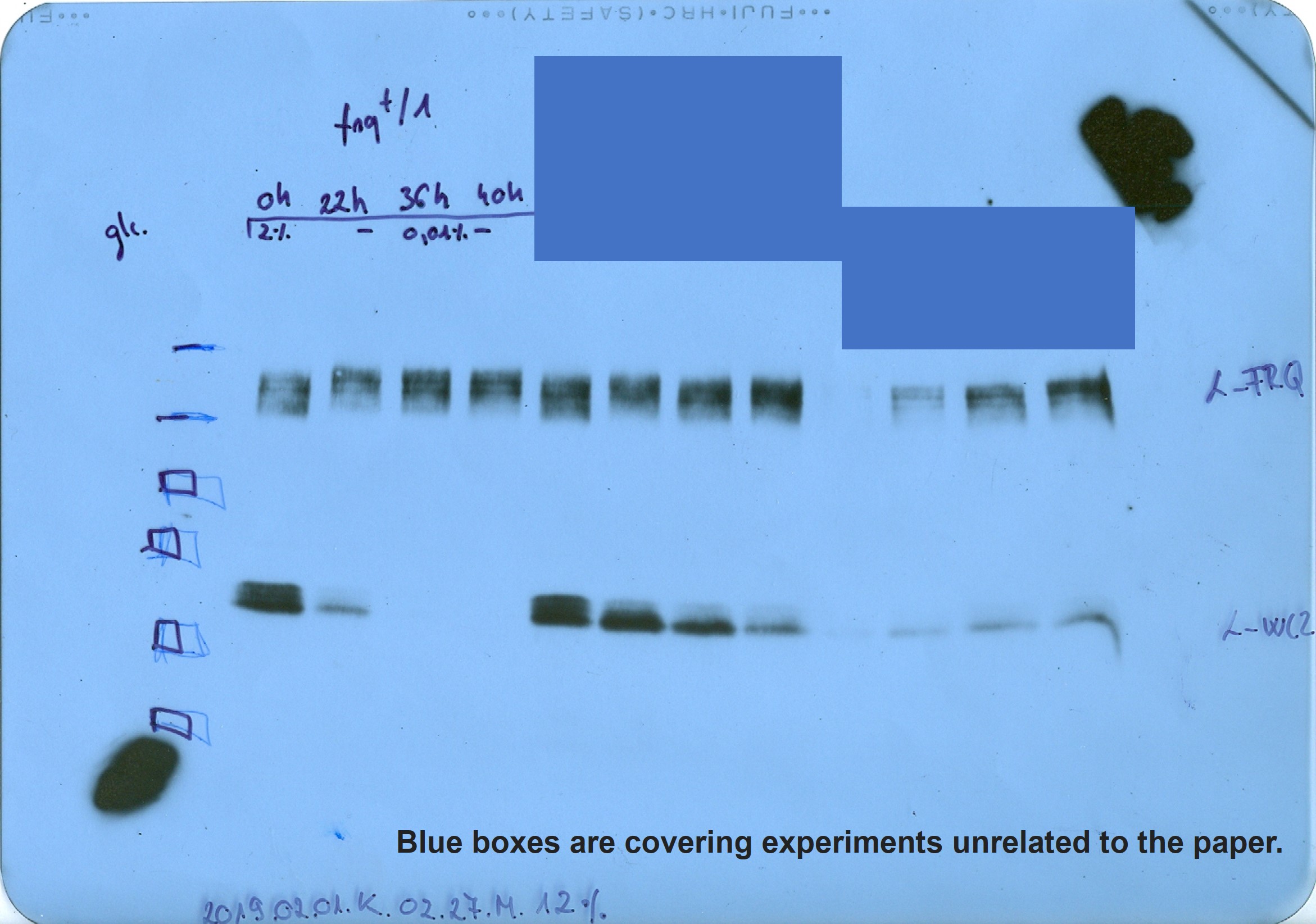

Supplement: Figure 1—source data 1. — (a) Western blots were used to detect expression of FRQ in the indicated samples for Figure 1B. (b) Figure with the area highlighted was used to develop the Figure 1B for FRQ. (c) Western blots were used to detect expression of WC-1 in the indicated samples for Figure 1B. (d) Figure with the area highlighted was used to develop the Figure 1B for WC-1. (e) Western blots were used to detect expression of WC-2 in the indicated samples for Figure 1B. (f) Figure with the area highlighted was used to develop the Figure 1B for WC-2. (g) Western blots were used to detect expression of RGB1 in the indicated samples for Figure 1B. (h) Figure with the area highlighted was used to develop the Figure 1B for RGB1. (i) Ponceau S staining was used to detect loading control of the indicated samples for Figure 1B. (j) Figure with the area highlighted was used for LC of Figure 1B . [file elife-79765-fig1-data1.zip › 79765Figure1B/Figure_1-source_data_1a.jpg]

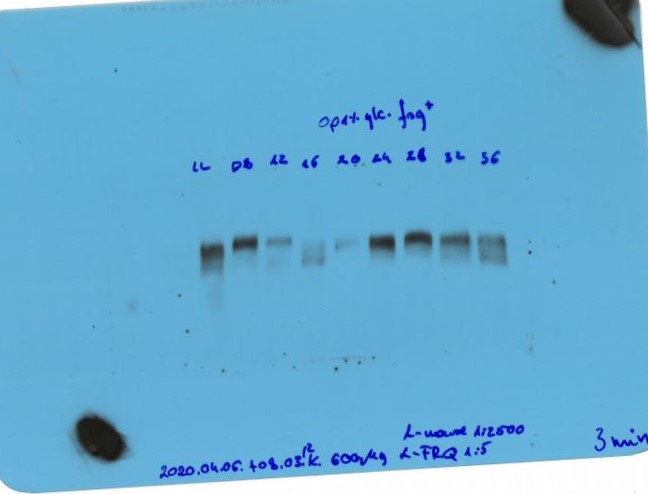

Supplement: Figure 1—source data 2. — (a) Western blots were used to detect expression of FRQ in the indicated samples grown in 0.01% glucose containing medium for Figure 1E. (b) Figure with the area highlighted was used to develop the Figure 1E for FRQ in 0.01% glucose. (c) Western blots were used to detect expression of FRQ in the indicated samples grown in 2% glucose containing medium for Figure 1E. (d) Figure with the area highlighted was used to develop the Figure 1E for FRQ in 2% glucose. (e) Ponceau S staining was used to detect loading control of the indicated samples for Figure 1E (0.01% glucose). (f) Figure with the area highlighted was used to develop the Figure 1E for LC (0.01% glucose). (g) Ponceau S staining was used to detect loading control of the indicated samples for Figure 1E (2% glucose). (h) Figure with the area highlighted was used to detect loading control of the indicated samples for Figure 1E (2% glucose). [file elife-79765-fig1-data2.zip › 79765Figure1E/Figure_1-source_data_2a.JPG]

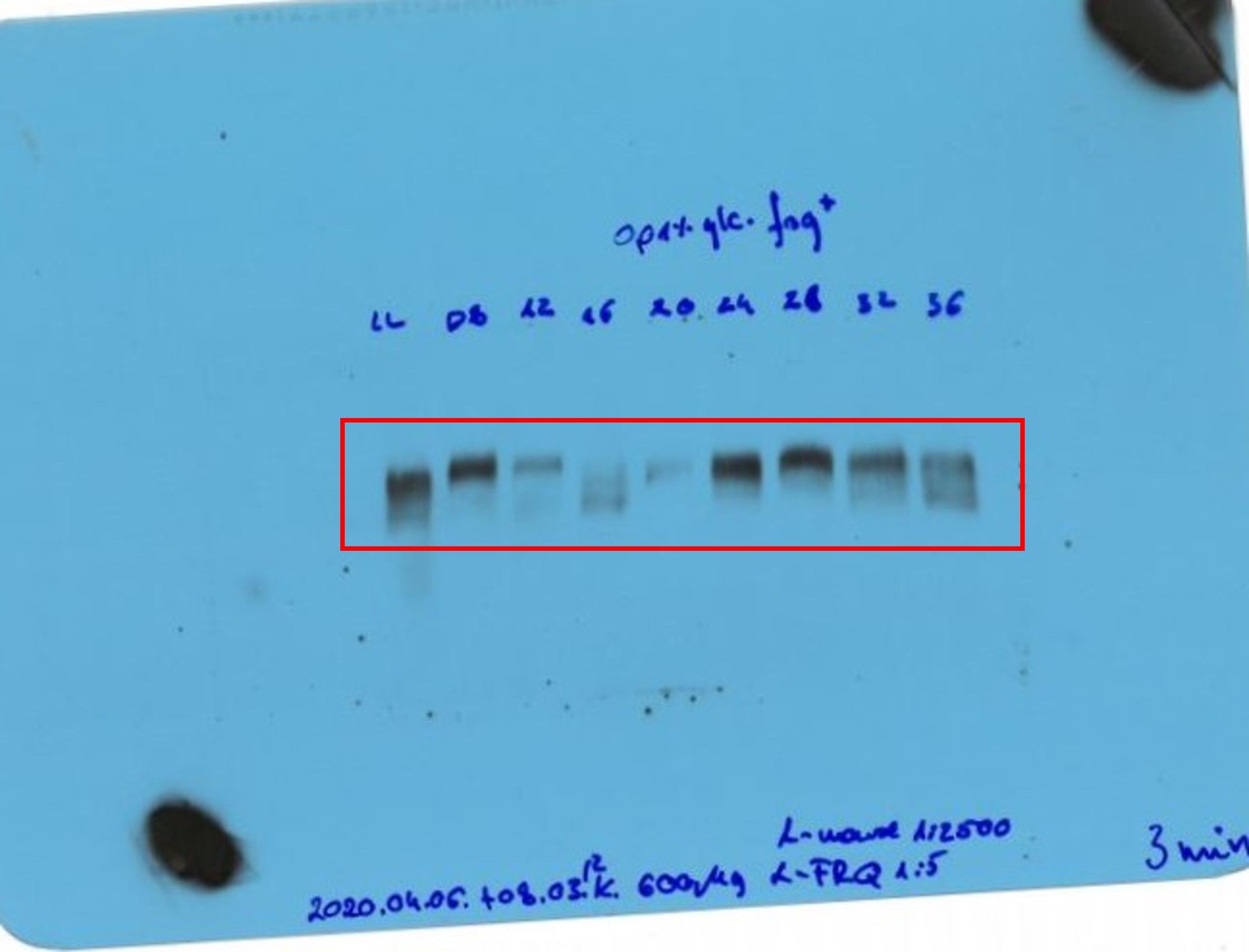

Supplement: Figure 1—source data 2. — (a) Western blots were used to detect expression of FRQ in the indicated samples grown in 0.01% glucose containing medium for Figure 1E. (b) Figure with the area highlighted was used to develop the Figure 1E for FRQ in 0.01% glucose. (c) Western blots were used to detect expression of FRQ in the indicated samples grown in 2% glucose containing medium for Figure 1E. (d) Figure with the area highlighted was used to develop the Figure 1E for FRQ in 2% glucose. (e) Ponceau S staining was used to detect loading control of the indicated samples for Figure 1E (0.01% glucose). (f) Figure with the area highlighted was used to develop the Figure 1E for LC (0.01% glucose). (g) Ponceau S staining was used to detect loading control of the indicated samples for Figure 1E (2% glucose). (h) Figure with the area highlighted was used to detect loading control of the indicated samples for Figure 1E (2% glucose). [file elife-79765-fig1-data2.zip › 79765Figure1E/Figure_1-source_data_2b.JPG]

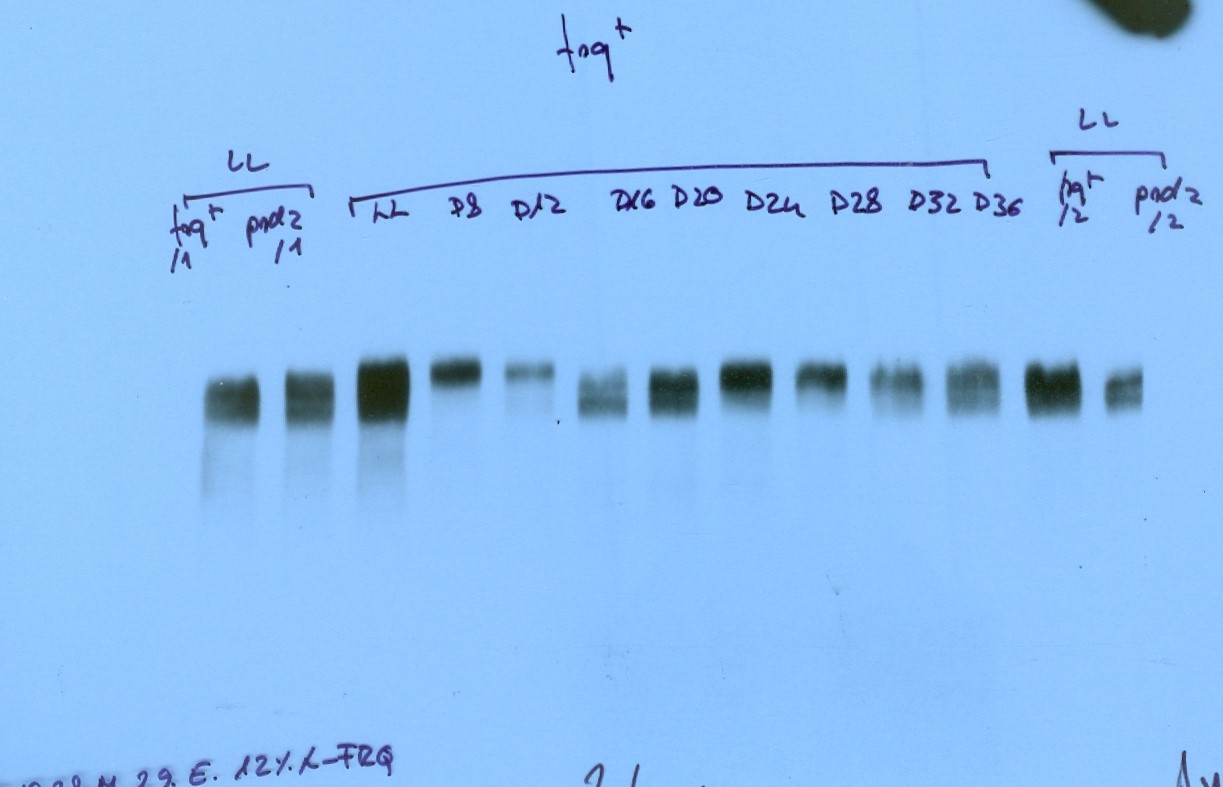

Supplement: Figure 1—source data 2. — (a) Western blots were used to detect expression of FRQ in the indicated samples grown in 0.01% glucose containing medium for Figure 1E. (b) Figure with the area highlighted was used to develop the Figure 1E for FRQ in 0.01% glucose. (c) Western blots were used to detect expression of FRQ in the indicated samples grown in 2% glucose containing medium for Figure 1E. (d) Figure with the area highlighted was used to develop the Figure 1E for FRQ in 2% glucose. (e) Ponceau S staining was used to detect loading control of the indicated samples for Figure 1E (0.01% glucose). (f) Figure with the area highlighted was used to develop the Figure 1E for LC (0.01% glucose). (g) Ponceau S staining was used to detect loading control of the indicated samples for Figure 1E (2% glucose). (h) Figure with the area highlighted was used to detect loading control of the indicated samples for Figure 1E (2% glucose). [file elife-79765-fig1-data2.zip › 79765Figure1E/Figure_1-source_data_2c.jpg]

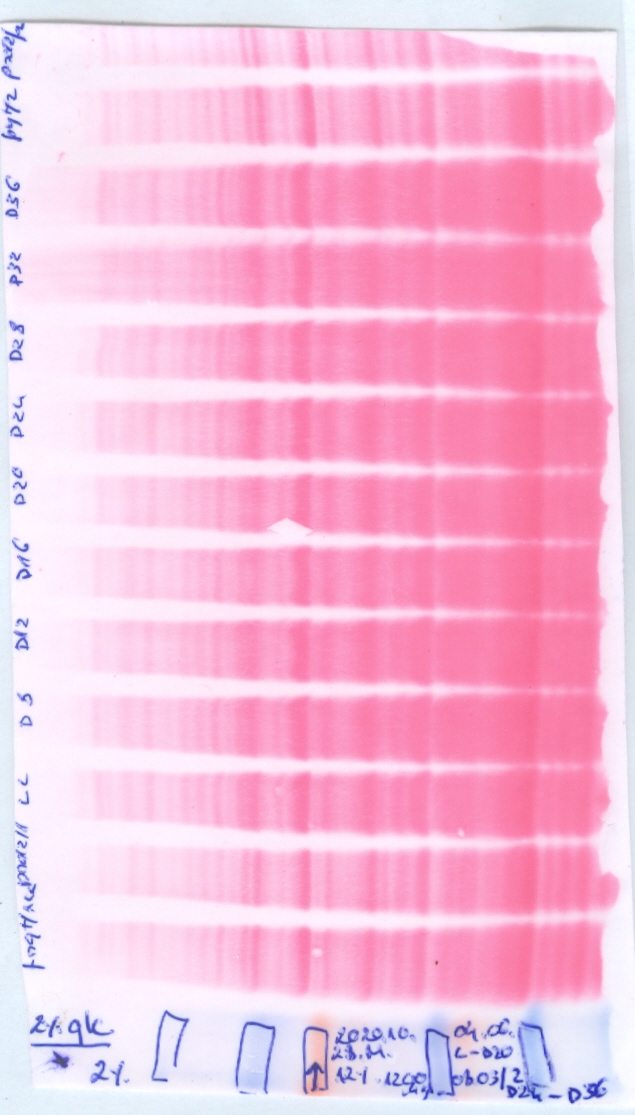

Supplement: Figure 1—source data 2. — (a) Western blots were used to detect expression of FRQ in the indicated samples grown in 0.01% glucose containing medium for Figure 1E. (b) Figure with the area highlighted was used to develop the Figure 1E for FRQ in 0.01% glucose. (c) Western blots were used to detect expression of FRQ in the indicated samples grown in 2% glucose containing medium for Figure 1E. (d) Figure with the area highlighted was used to develop the Figure 1E for FRQ in 2% glucose. (e) Ponceau S staining was used to detect loading control of the indicated samples for Figure 1E (0.01% glucose). (f) Figure with the area highlighted was used to develop the Figure 1E for LC (0.01% glucose). (g) Ponceau S staining was used to detect loading control of the indicated samples for Figure 1E (2% glucose). (h) Figure with the area highlighted was used to detect loading control of the indicated samples for Figure 1E (2% glucose). [file elife-79765-fig1-data2.zip › 79765Figure1E/Figure_1-source_data_2g.jpg]

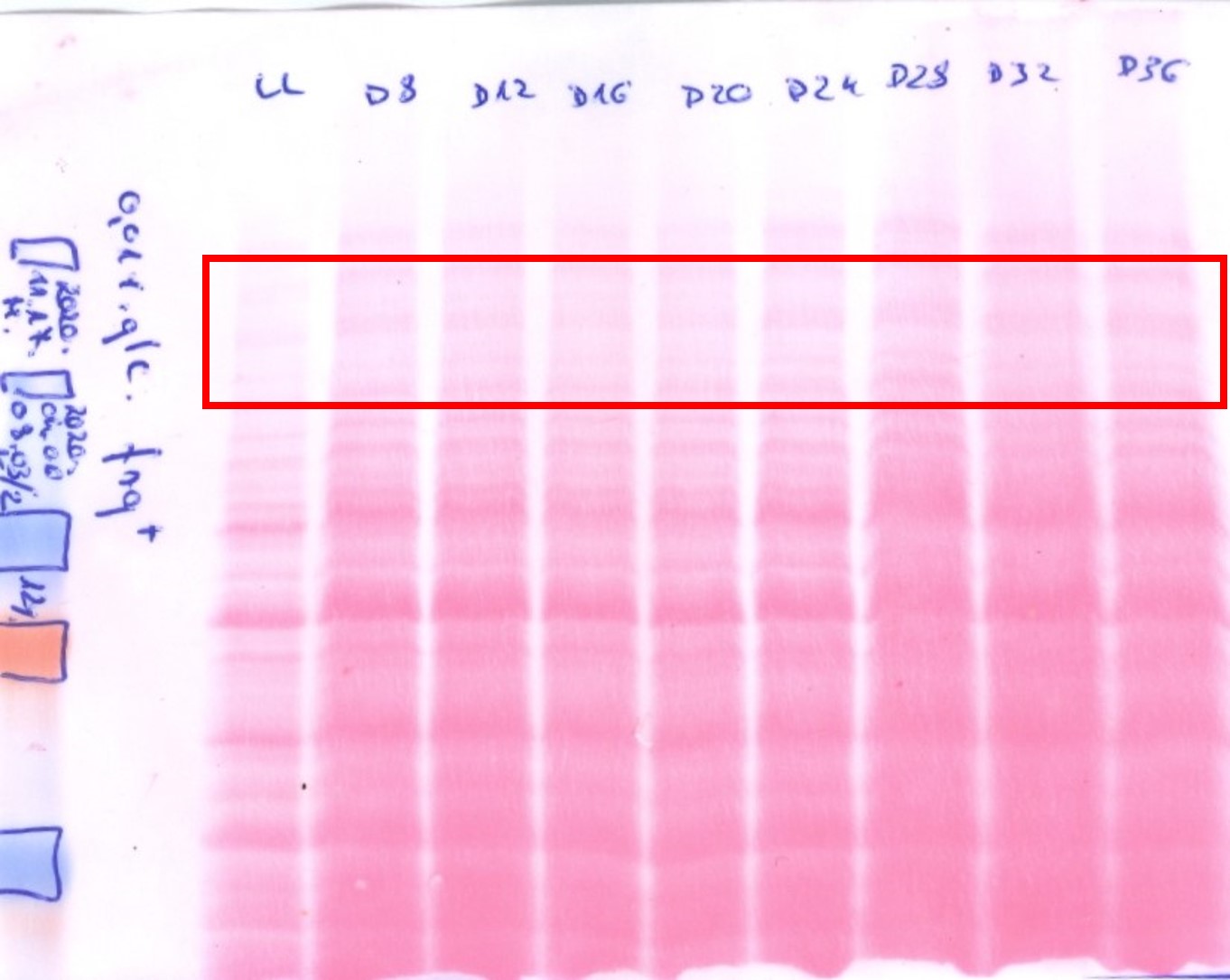

Supplement: Figure 1—source data 2. — (a) Western blots were used to detect expression of FRQ in the indicated samples grown in 0.01% glucose containing medium for Figure 1E. (b) Figure with the area highlighted was used to develop the Figure 1E for FRQ in 0.01% glucose. (c) Western blots were used to detect expression of FRQ in the indicated samples grown in 2% glucose containing medium for Figure 1E. (d) Figure with the area highlighted was used to develop the Figure 1E for FRQ in 2% glucose. (e) Ponceau S staining was used to detect loading control of the indicated samples for Figure 1E (0.01% glucose). (f) Figure with the area highlighted was used to develop the Figure 1E for LC (0.01% glucose). (g) Ponceau S staining was used to detect loading control of the indicated samples for Figure 1E (2% glucose). (h) Figure with the area highlighted was used to detect loading control of the indicated samples for Figure 1E (2% glucose). [file elife-79765-fig1-data2.zip › 79765Figure1E/Figure_1-source_data_2f.jpg]

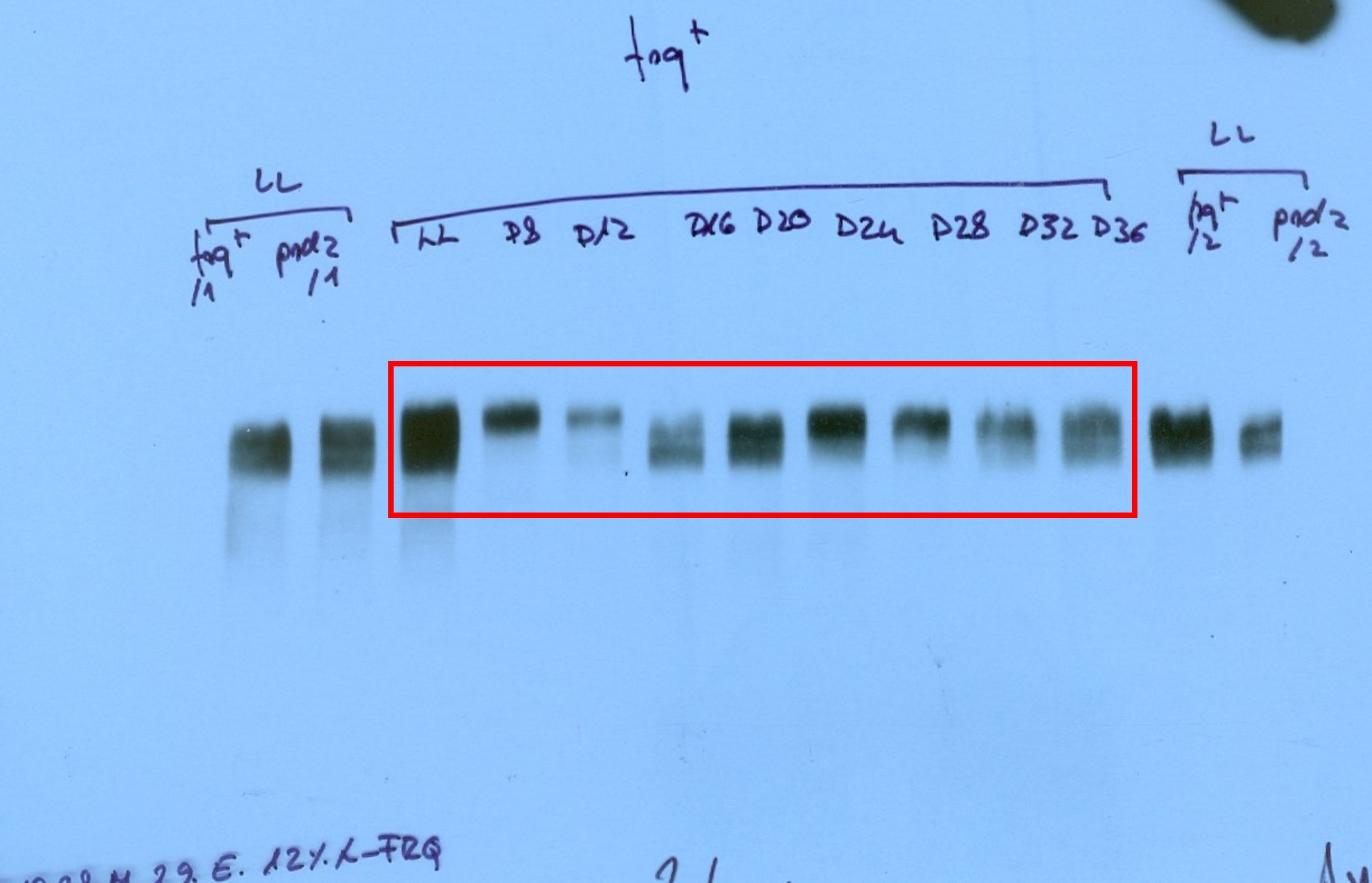

Supplement: Figure 1—source data 2. — (a) Western blots were used to detect expression of FRQ in the indicated samples grown in 0.01% glucose containing medium for Figure 1E. (b) Figure with the area highlighted was used to develop the Figure 1E for FRQ in 0.01% glucose. (c) Western blots were used to detect expression of FRQ in the indicated samples grown in 2% glucose containing medium for Figure 1E. (d) Figure with the area highlighted was used to develop the Figure 1E for FRQ in 2% glucose. (e) Ponceau S staining was used to detect loading control of the indicated samples for Figure 1E (0.01% glucose). (f) Figure with the area highlighted was used to develop the Figure 1E for LC (0.01% glucose). (g) Ponceau S staining was used to detect loading control of the indicated samples for Figure 1E (2% glucose). (h) Figure with the area highlighted was used to detect loading control of the indicated samples for Figure 1E (2% glucose). [file elife-79765-fig1-data2.zip › 79765Figure1E/Figure_1-source_data_2d.jpg]

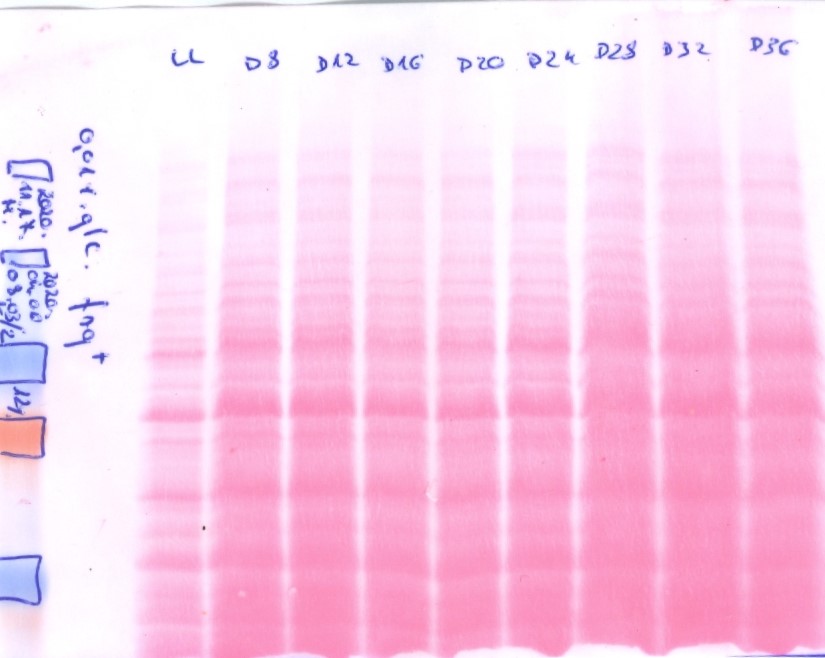

Supplement: Figure 1—source data 2. — (a) Western blots were used to detect expression of FRQ in the indicated samples grown in 0.01% glucose containing medium for Figure 1E. (b) Figure with the area highlighted was used to develop the Figure 1E for FRQ in 0.01% glucose. (c) Western blots were used to detect expression of FRQ in the indicated samples grown in 2% glucose containing medium for Figure 1E. (d) Figure with the area highlighted was used to develop the Figure 1E for FRQ in 2% glucose. (e) Ponceau S staining was used to detect loading control of the indicated samples for Figure 1E (0.01% glucose). (f) Figure with the area highlighted was used to develop the Figure 1E for LC (0.01% glucose). (g) Ponceau S staining was used to detect loading control of the indicated samples for Figure 1E (2% glucose). (h) Figure with the area highlighted was used to detect loading control of the indicated samples for Figure 1E (2% glucose). [file elife-79765-fig1-data2.zip › 79765Figure1E/Figure_1-source_data_2e.jpg]

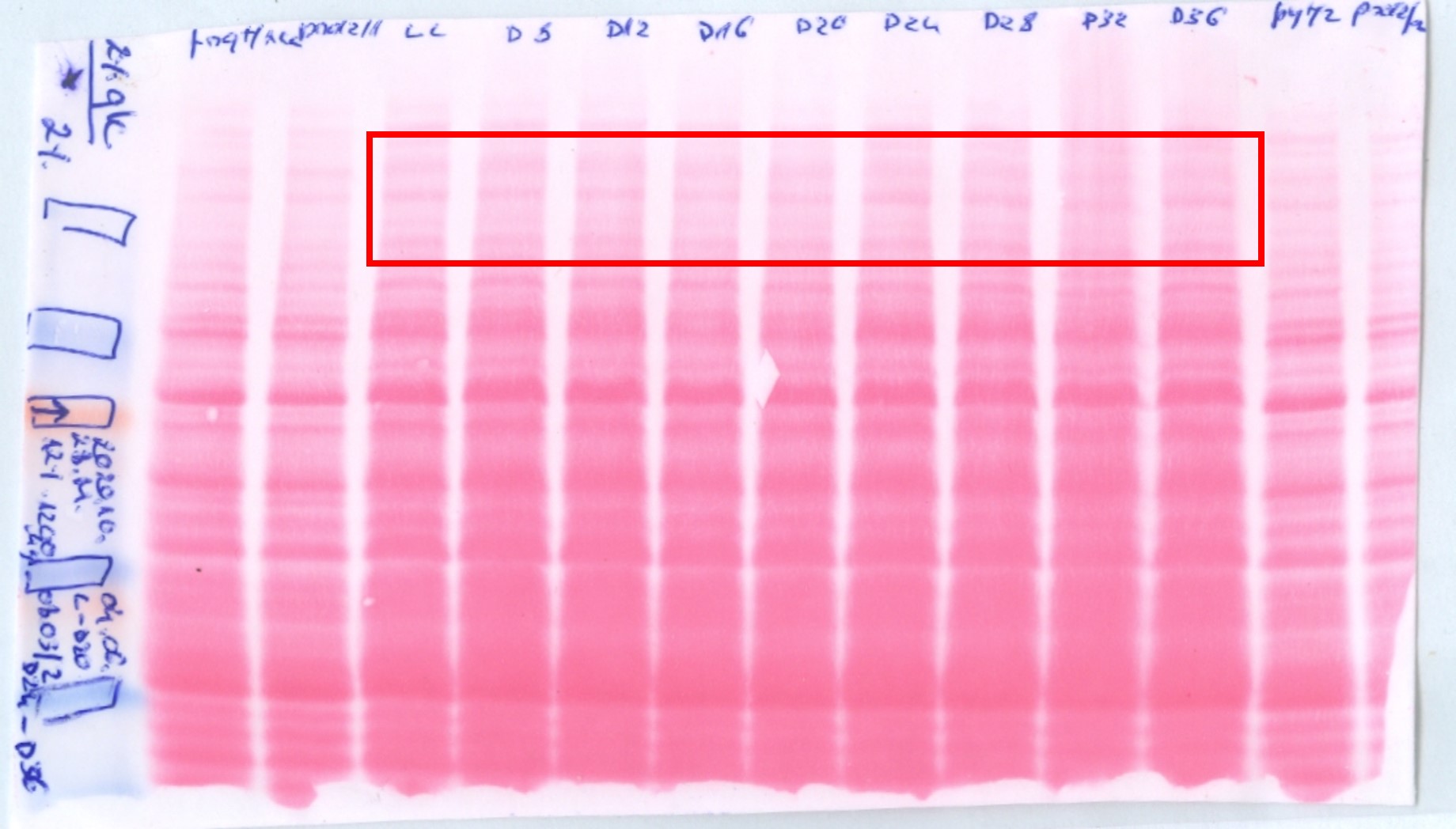

Supplement: Figure 1—source data 2. — (a) Western blots were used to detect expression of FRQ in the indicated samples grown in 0.01% glucose containing medium for Figure 1E. (b) Figure with the area highlighted was used to develop the Figure 1E for FRQ in 0.01% glucose. (c) Western blots were used to detect expression of FRQ in the indicated samples grown in 2% glucose containing medium for Figure 1E. (d) Figure with the area highlighted was used to develop the Figure 1E for FRQ in 2% glucose. (e) Ponceau S staining was used to detect loading control of the indicated samples for Figure 1E (0.01% glucose). (f) Figure with the area highlighted was used to develop the Figure 1E for LC (0.01% glucose). (g) Ponceau S staining was used to detect loading control of the indicated samples for Figure 1E (2% glucose). (h) Figure with the area highlighted was used to detect loading control of the indicated samples for Figure 1E (2% glucose). [file elife-79765-fig1-data2.zip › 79765Figure1E/Figure_1-source_data_2h.jpg]

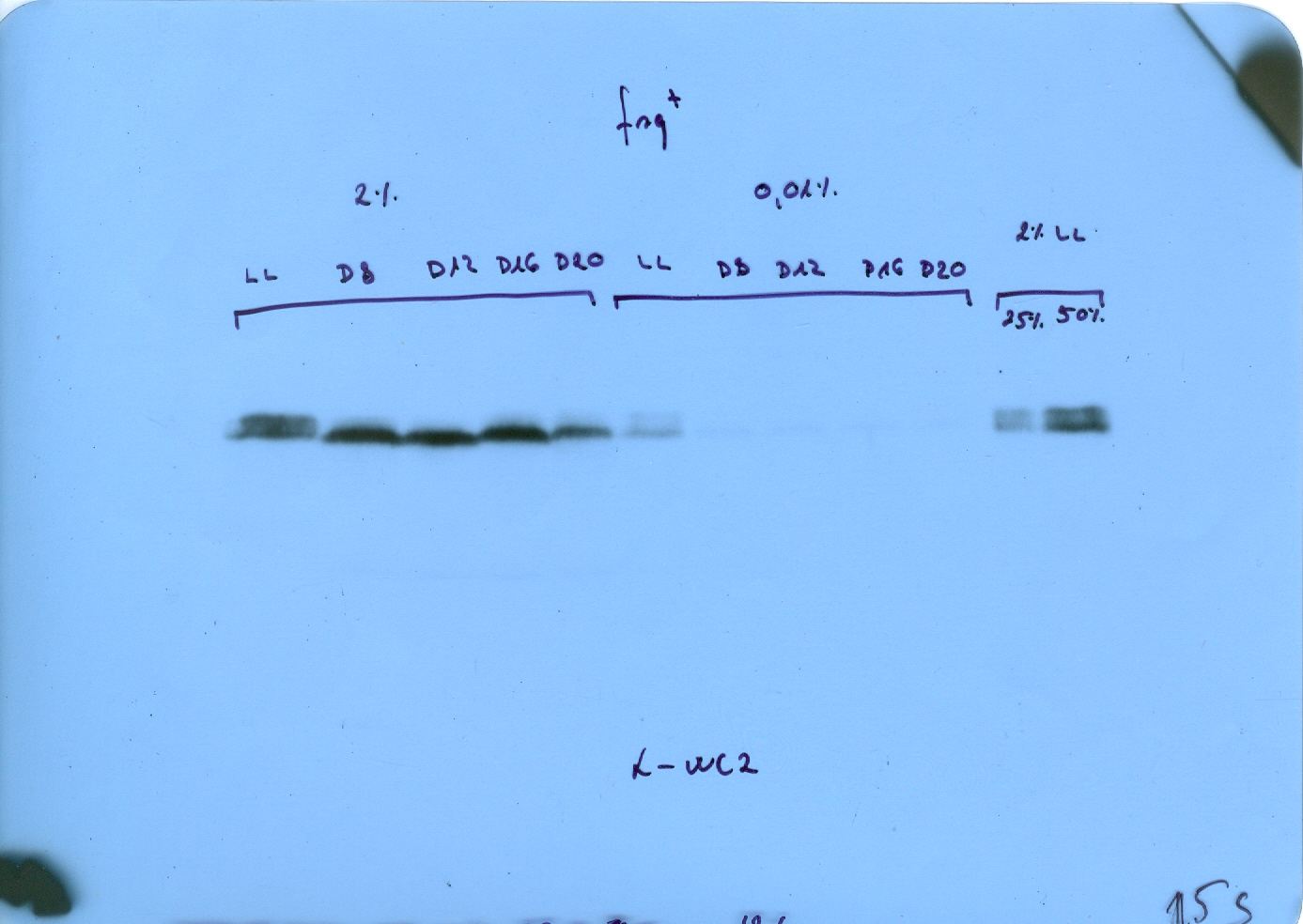

Supplement: Figure 1—source data 3. — (a) Western blots were used to detect expression of FRQ in the indicated samples for Figure 1F. (b) Figure with the area highlighted was used to develop the Figure 1F for FRQ. (c) Western blots were used to detect expression of WC-1 in the indicated samples for Figure 1F. (d) Figure with the area highlighted was used to develop the Figure 1F for WC-1. (e) Western blots were used to detect expression of WC-2 in the indicated samples for Figure 1F. (f) Figure with the area highlighted was used to develop the Figure 1F for WC-2. (g) Ponceau S staining was used to detect loading control of the indicated samples for Figure 1F. (h) Figure with the area highlighted was used as LC for Figure 1F. [file elife-79765-fig1-data3.zip › 79765Figure1F/Figure_1-source_data_3e.jpg]

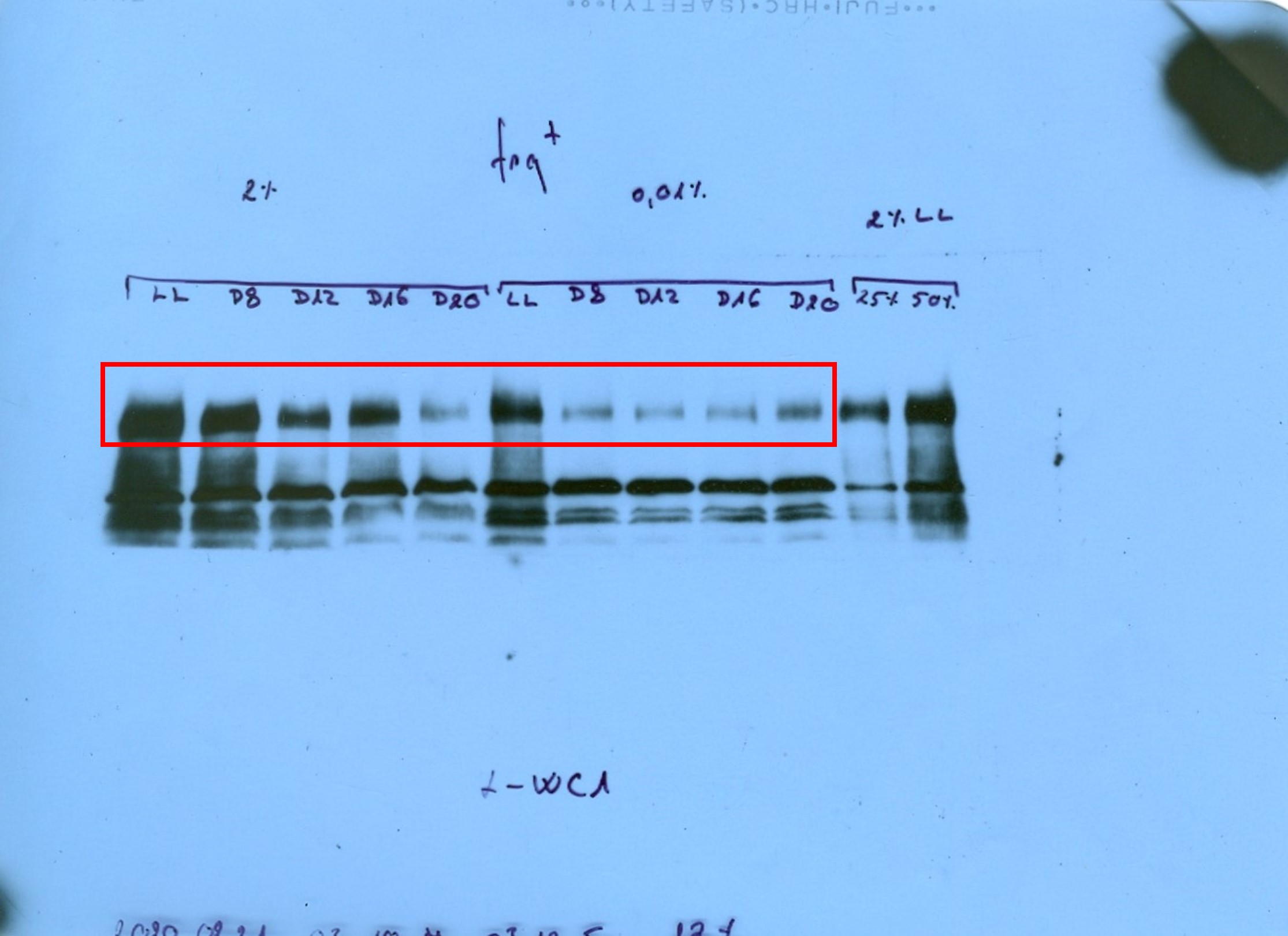

Supplement: Figure 1—source data 3. — (a) Western blots were used to detect expression of FRQ in the indicated samples for Figure 1F. (b) Figure with the area highlighted was used to develop the Figure 1F for FRQ. (c) Western blots were used to detect expression of WC-1 in the indicated samples for Figure 1F. (d) Figure with the area highlighted was used to develop the Figure 1F for WC-1. (e) Western blots were used to detect expression of WC-2 in the indicated samples for Figure 1F. (f) Figure with the area highlighted was used to develop the Figure 1F for WC-2. (g) Ponceau S staining was used to detect loading control of the indicated samples for Figure 1F. (h) Figure with the area highlighted was used as LC for Figure 1F. [file elife-79765-fig1-data3.zip › 79765Figure1F/Figure_1-source_data_3d.jpg]

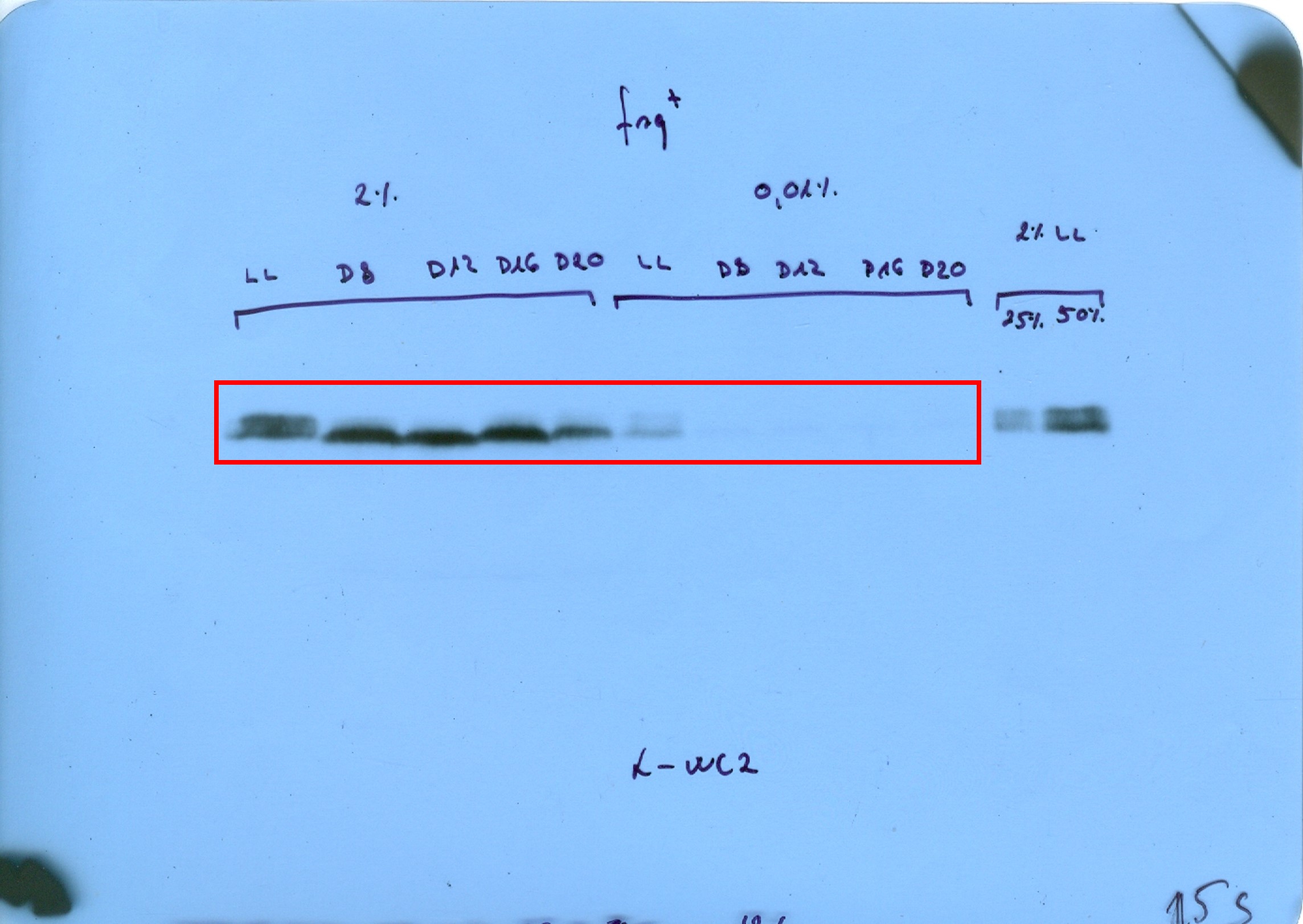

Supplement: Figure 1—source data 3. — (a) Western blots were used to detect expression of FRQ in the indicated samples for Figure 1F. (b) Figure with the area highlighted was used to develop the Figure 1F for FRQ. (c) Western blots were used to detect expression of WC-1 in the indicated samples for Figure 1F. (d) Figure with the area highlighted was used to develop the Figure 1F for WC-1. (e) Western blots were used to detect expression of WC-2 in the indicated samples for Figure 1F. (f) Figure with the area highlighted was used to develop the Figure 1F for WC-2. (g) Ponceau S staining was used to detect loading control of the indicated samples for Figure 1F. (h) Figure with the area highlighted was used as LC for Figure 1F. [file elife-79765-fig1-data3.zip › 79765Figure1F/Figure_1-source_data_3f.jpg]

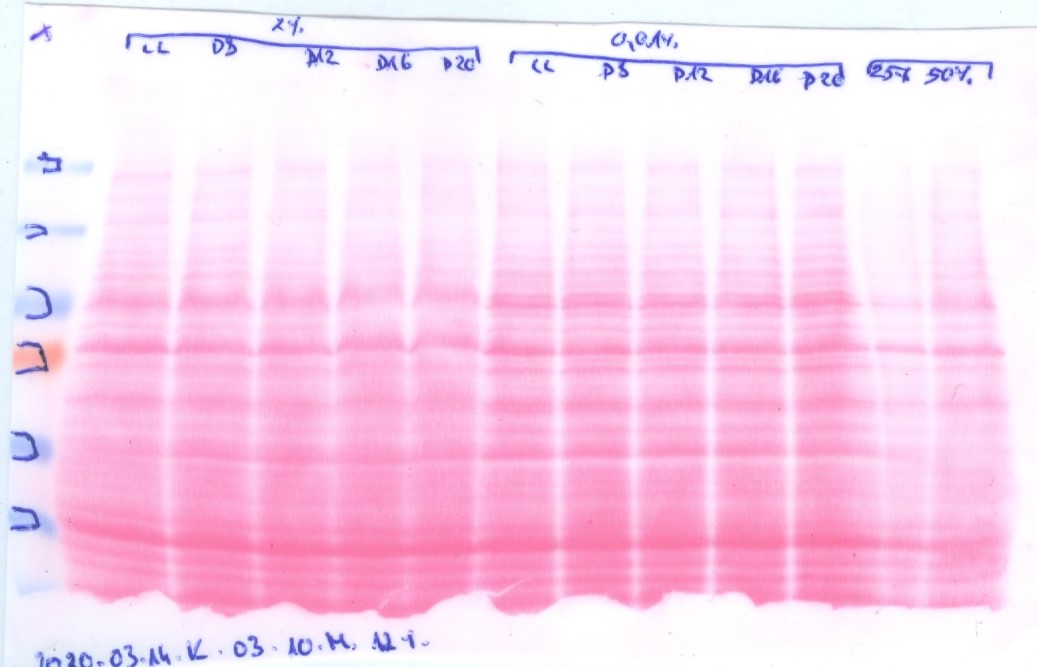

Supplement: Figure 1—source data 3. — (a) Western blots were used to detect expression of FRQ in the indicated samples for Figure 1F. (b) Figure with the area highlighted was used to develop the Figure 1F for FRQ. (c) Western blots were used to detect expression of WC-1 in the indicated samples for Figure 1F. (d) Figure with the area highlighted was used to develop the Figure 1F for WC-1. (e) Western blots were used to detect expression of WC-2 in the indicated samples for Figure 1F. (f) Figure with the area highlighted was used to develop the Figure 1F for WC-2. (g) Ponceau S staining was used to detect loading control of the indicated samples for Figure 1F. (h) Figure with the area highlighted was used as LC for Figure 1F. [file elife-79765-fig1-data3.zip › 79765Figure1F/Figure_1-source_data_3g.jpg]

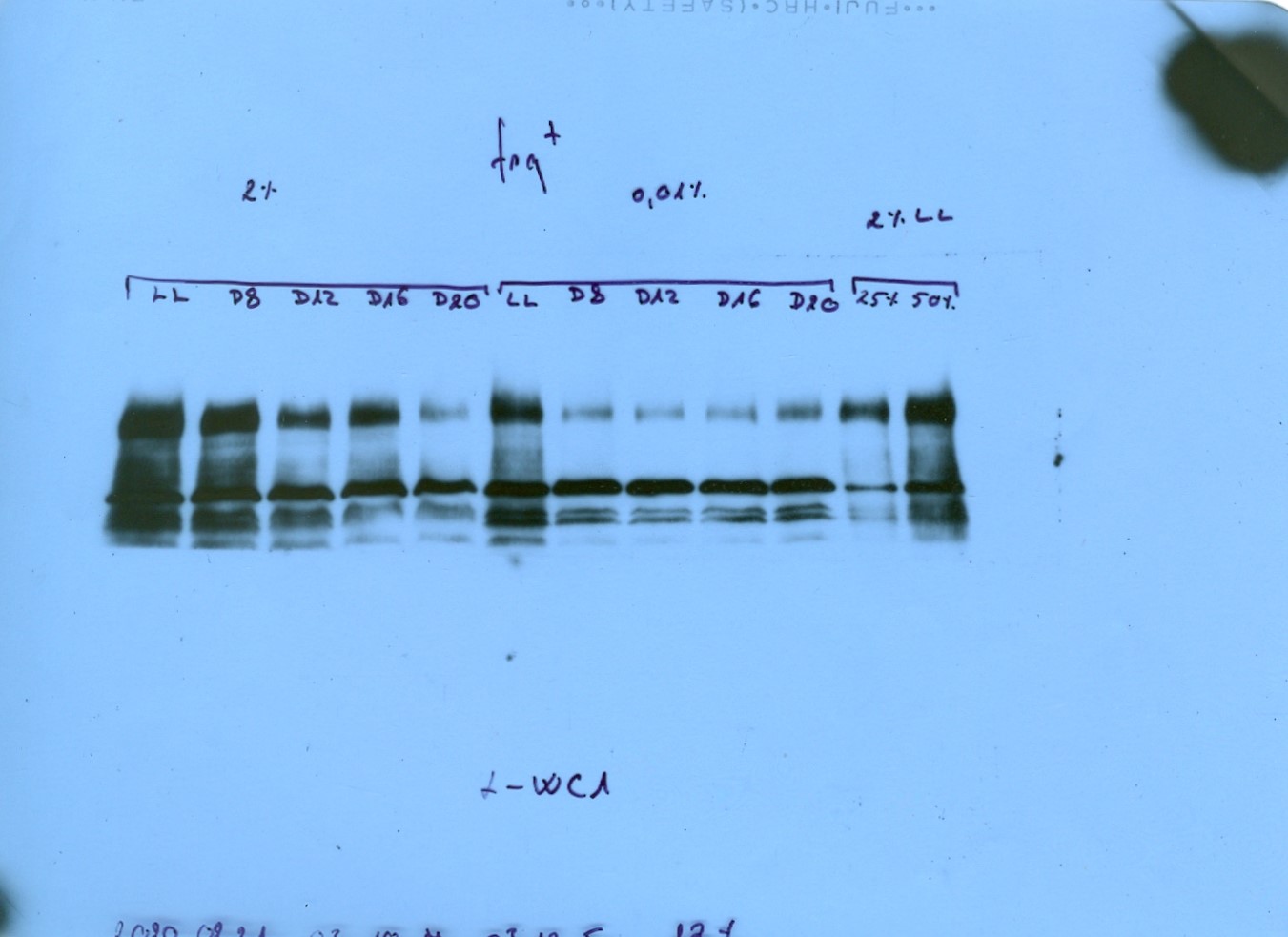

Supplement: Figure 1—source data 3. — (a) Western blots were used to detect expression of FRQ in the indicated samples for Figure 1F. (b) Figure with the area highlighted was used to develop the Figure 1F for FRQ. (c) Western blots were used to detect expression of WC-1 in the indicated samples for Figure 1F. (d) Figure with the area highlighted was used to develop the Figure 1F for WC-1. (e) Western blots were used to detect expression of WC-2 in the indicated samples for Figure 1F. (f) Figure with the area highlighted was used to develop the Figure 1F for WC-2. (g) Ponceau S staining was used to detect loading control of the indicated samples for Figure 1F. (h) Figure with the area highlighted was used as LC for Figure 1F. [file elife-79765-fig1-data3.zip › 79765Figure1F/Figure_1-source_data_3c.jpg]

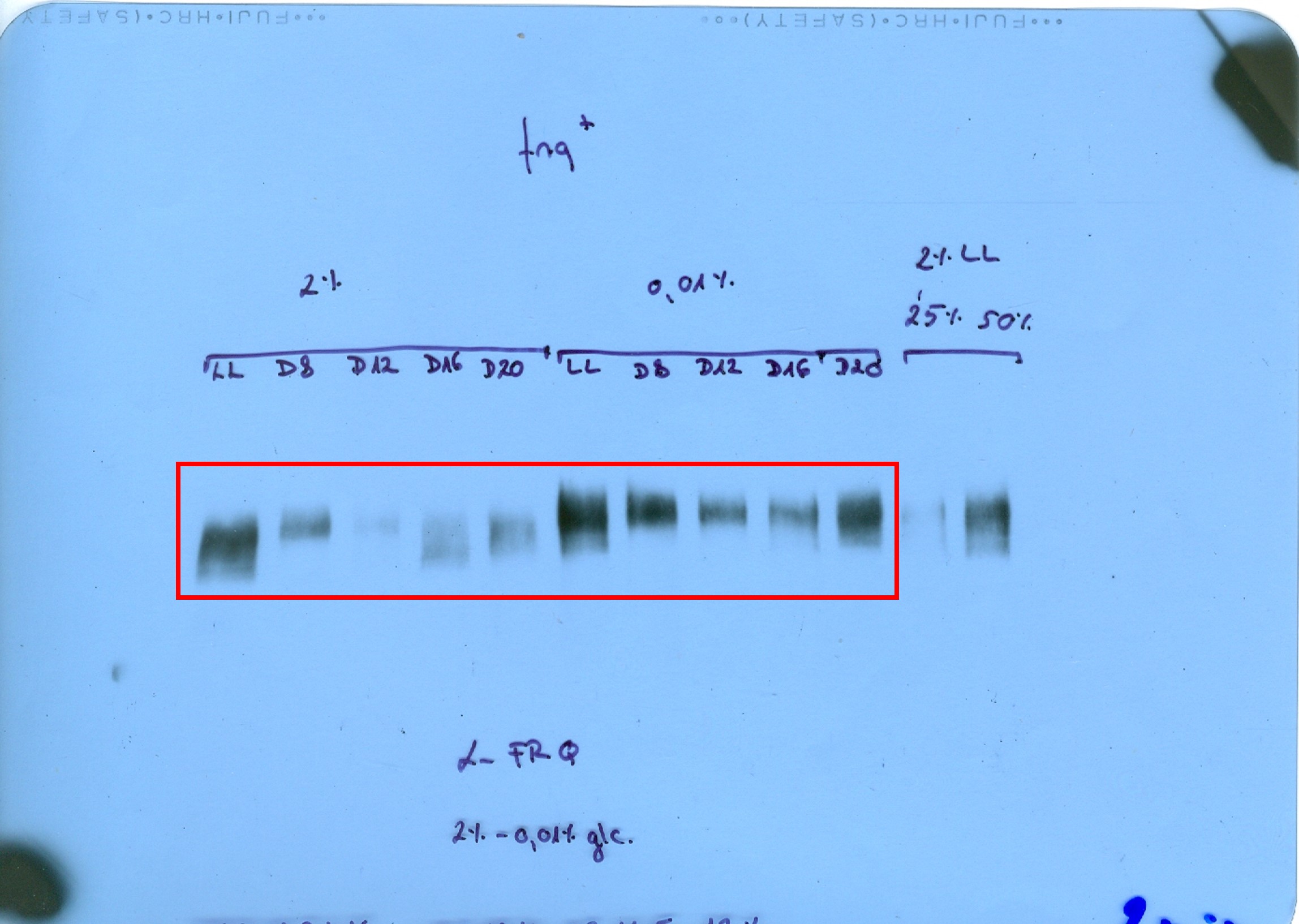

Supplement: Figure 1—source data 3. — (a) Western blots were used to detect expression of FRQ in the indicated samples for Figure 1F. (b) Figure with the area highlighted was used to develop the Figure 1F for FRQ. (c) Western blots were used to detect expression of WC-1 in the indicated samples for Figure 1F. (d) Figure with the area highlighted was used to develop the Figure 1F for WC-1. (e) Western blots were used to detect expression of WC-2 in the indicated samples for Figure 1F. (f) Figure with the area highlighted was used to develop the Figure 1F for WC-2. (g) Ponceau S staining was used to detect loading control of the indicated samples for Figure 1F. (h) Figure with the area highlighted was used as LC for Figure 1F. [file elife-79765-fig1-data3.zip › 79765Figure1F/Figure_1-source_data_3b.jpg]

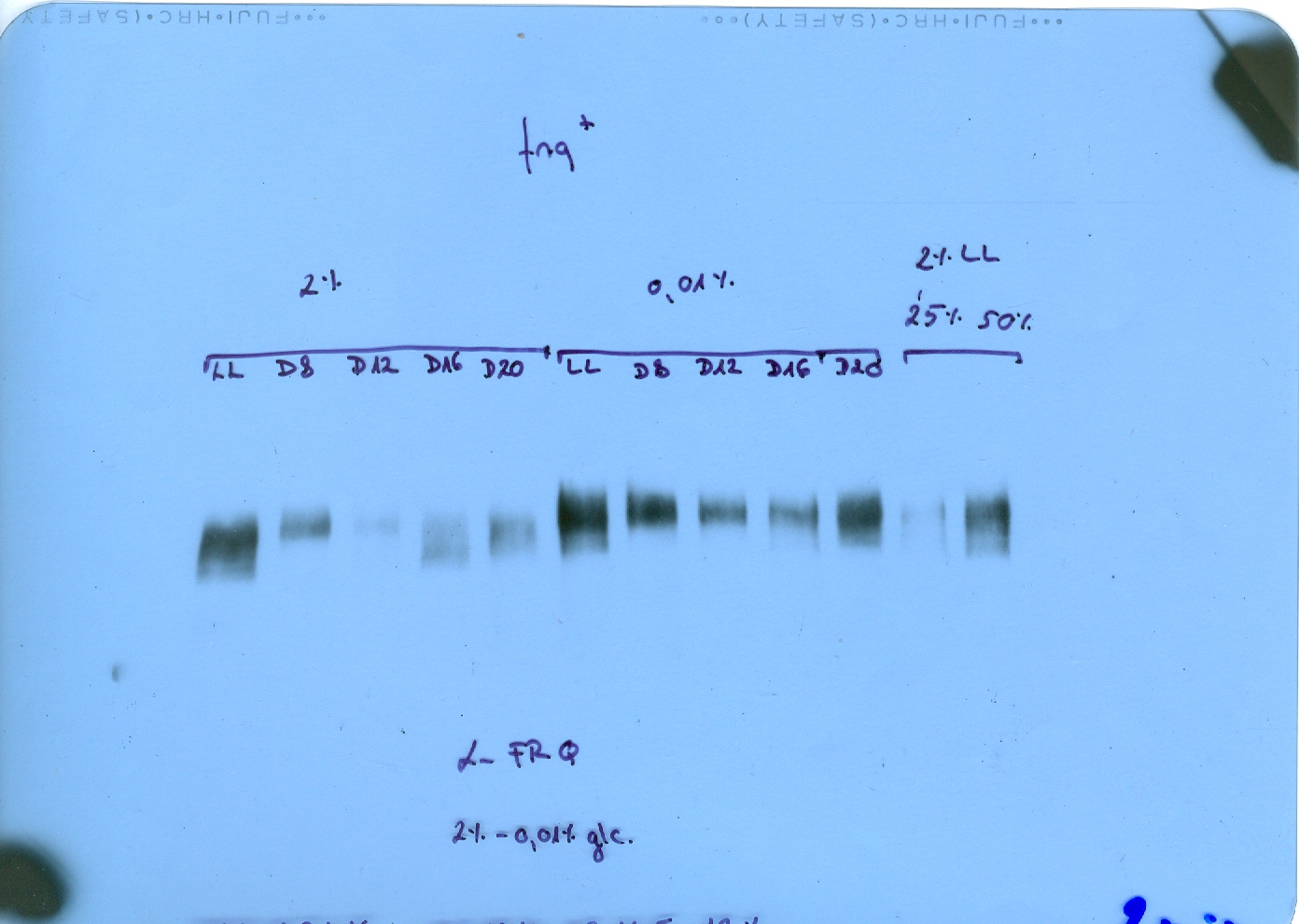

Supplement: Figure 1—source data 3. — (a) Western blots were used to detect expression of FRQ in the indicated samples for Figure 1F. (b) Figure with the area highlighted was used to develop the Figure 1F for FRQ. (c) Western blots were used to detect expression of WC-1 in the indicated samples for Figure 1F. (d) Figure with the area highlighted was used to develop the Figure 1F for WC-1. (e) Western blots were used to detect expression of WC-2 in the indicated samples for Figure 1F. (f) Figure with the area highlighted was used to develop the Figure 1F for WC-2. (g) Ponceau S staining was used to detect loading control of the indicated samples for Figure 1F. (h) Figure with the area highlighted was used as LC for Figure 1F. [file elife-79765-fig1-data3.zip › 79765Figure1F/Figure_1-source_data_3a.jpg]

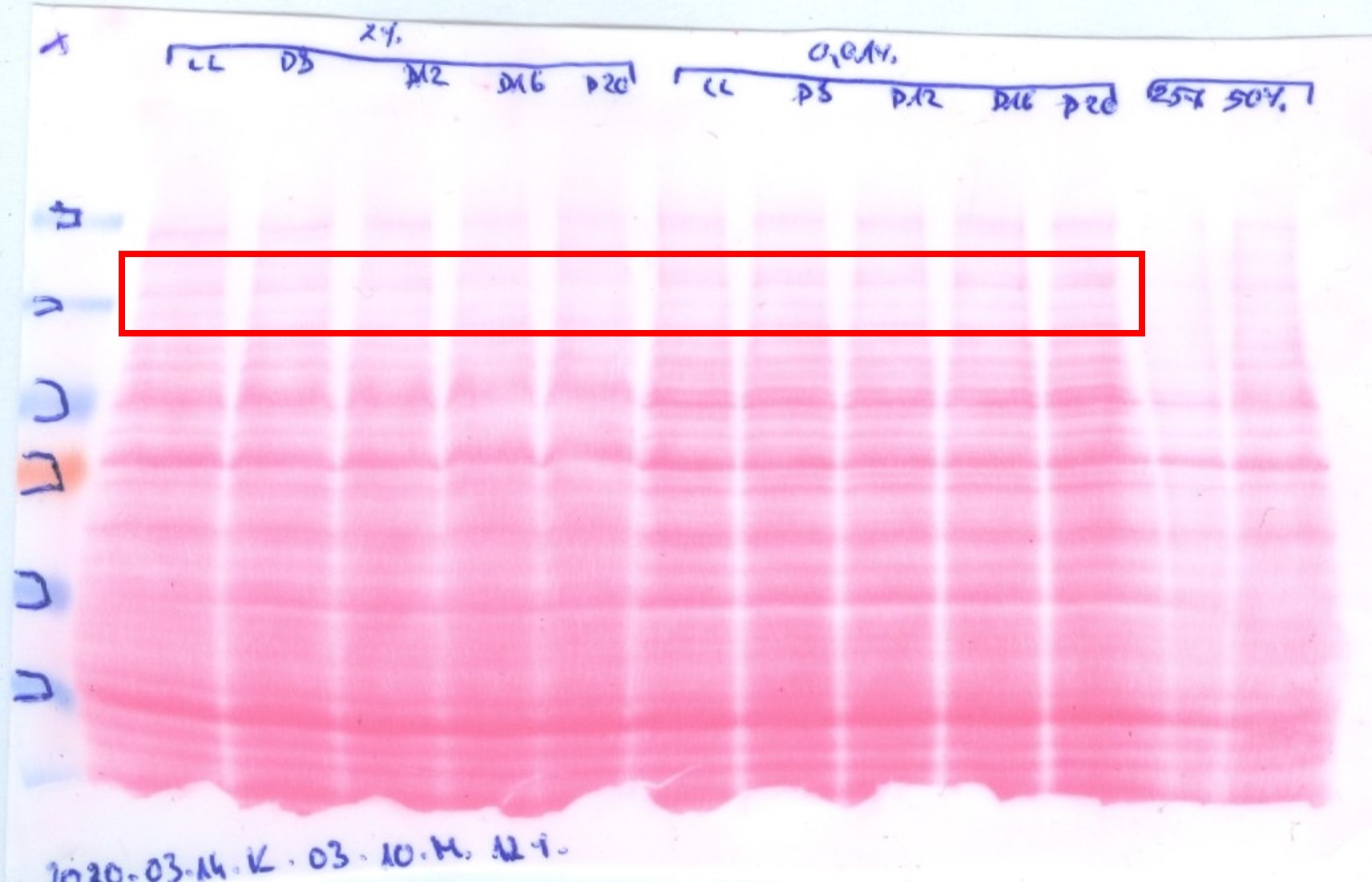

Supplement: Figure 1—source data 3. — (a) Western blots were used to detect expression of FRQ in the indicated samples for Figure 1F. (b) Figure with the area highlighted was used to develop the Figure 1F for FRQ. (c) Western blots were used to detect expression of WC-1 in the indicated samples for Figure 1F. (d) Figure with the area highlighted was used to develop the Figure 1F for WC-1. (e) Western blots were used to detect expression of WC-2 in the indicated samples for Figure 1F. (f) Figure with the area highlighted was used to develop the Figure 1F for WC-2. (g) Ponceau S staining was used to detect loading control of the indicated samples for Figure 1F. (h) Figure with the area highlighted was used as LC for Figure 1F. [file elife-79765-fig1-data3.zip › 79765Figure1F/Figure_1-source_data_3h.jpg]

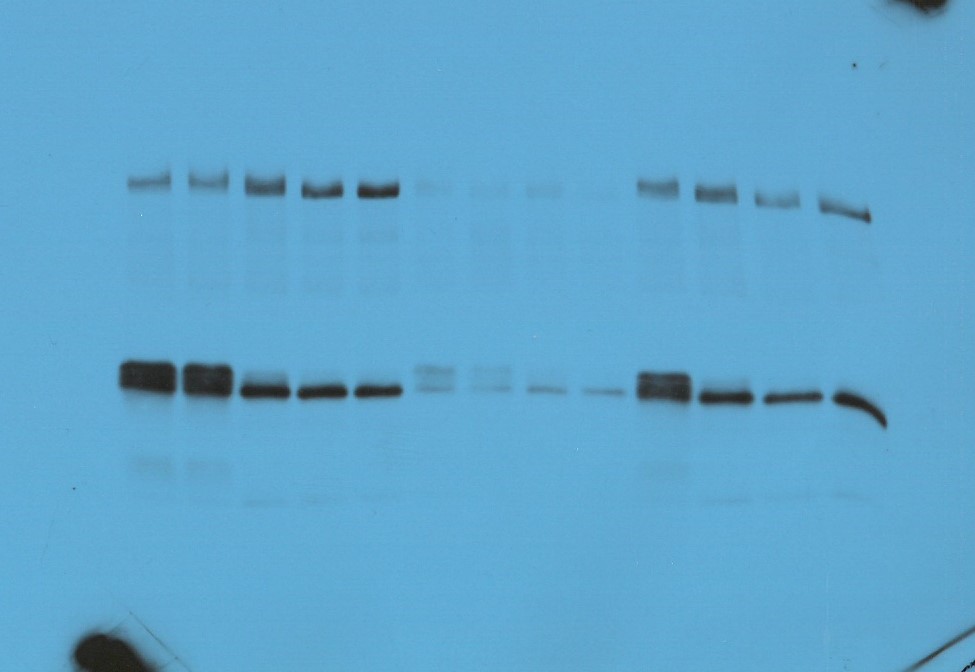

Supplement: Figure 1—figure supplement 1—source data 1. — (a) Western blots were used to detect expression of WC-1 in the indicated samples for Figure 1—figure supplement 1B (s: short exposure). (b) Figure with the area highlighted was used to develop the Figure 1—figure supplement 1B for WC-1 (s: short exposure). (c) Western blots were used to detect expression of WC-1 in the indicated samples for Figure 1—figure supplement 1B (l: long exposure). (d) Figure with the area highlighted was used to develop the Figure 1—figure supplement 1B for WC-1 (l: long exposure). (e) Western blots were used to detect expression of WC-2 in the indicated samples for Figure 1—figure supplement 1B (s: short exposure). (f) Figure with the area highlighted was used to develop the Figure 1—figure supplement 1B for WC-2 (s: short exposure). (g) Western blots were used to detect expression of WC-2 in the indicated samples for Figure 1—figure supplement 1B (l: long exposure). (h) Figure with the area highlighted was used to develop the Figure 1—figure supplement 1B for WC-2 (l: long exposure). (i) Ponceau S staining was used to detect loading control of the indicated samples for Figure 1—figure supplement 1B. (j) Figure with the area highlighted was used as LC of Figure 1—figure supplement 1B. [file elife-79765-fig1-figsupp1-data1.zip › 79765Figure1-S1B/Figure_1-Figure_supplement_1-source_data_1a.jpg]

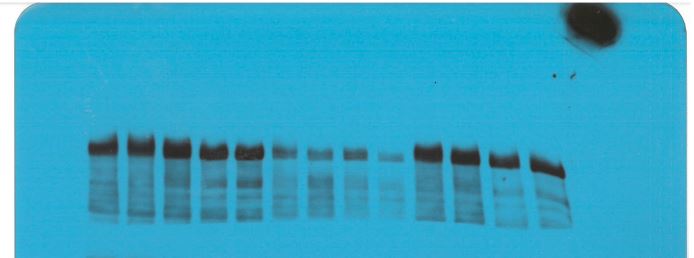

Supplement: Figure 1—figure supplement 1—source data 1. — (a) Western blots were used to detect expression of WC-1 in the indicated samples for Figure 1—figure supplement 1B (s: short exposure). (b) Figure with the area highlighted was used to develop the Figure 1—figure supplement 1B for WC-1 (s: short exposure). (c) Western blots were used to detect expression of WC-1 in the indicated samples for Figure 1—figure supplement 1B (l: long exposure). (d) Figure with the area highlighted was used to develop the Figure 1—figure supplement 1B for WC-1 (l: long exposure). (e) Western blots were used to detect expression of WC-2 in the indicated samples for Figure 1—figure supplement 1B (s: short exposure). (f) Figure with the area highlighted was used to develop the Figure 1—figure supplement 1B for WC-2 (s: short exposure). (g) Western blots were used to detect expression of WC-2 in the indicated samples for Figure 1—figure supplement 1B (l: long exposure). (h) Figure with the area highlighted was used to develop the Figure 1—figure supplement 1B for WC-2 (l: long exposure). (i) Ponceau S staining was used to detect loading control of the indicated samples for Figure 1—figure supplement 1B. (j) Figure with the area highlighted was used as LC of Figure 1—figure supplement 1B. [file elife-79765-fig1-figsupp1-data1.zip › 79765Figure1-S1B/Figure_1-Figure_supplement_1-source_data_1c.JPG]

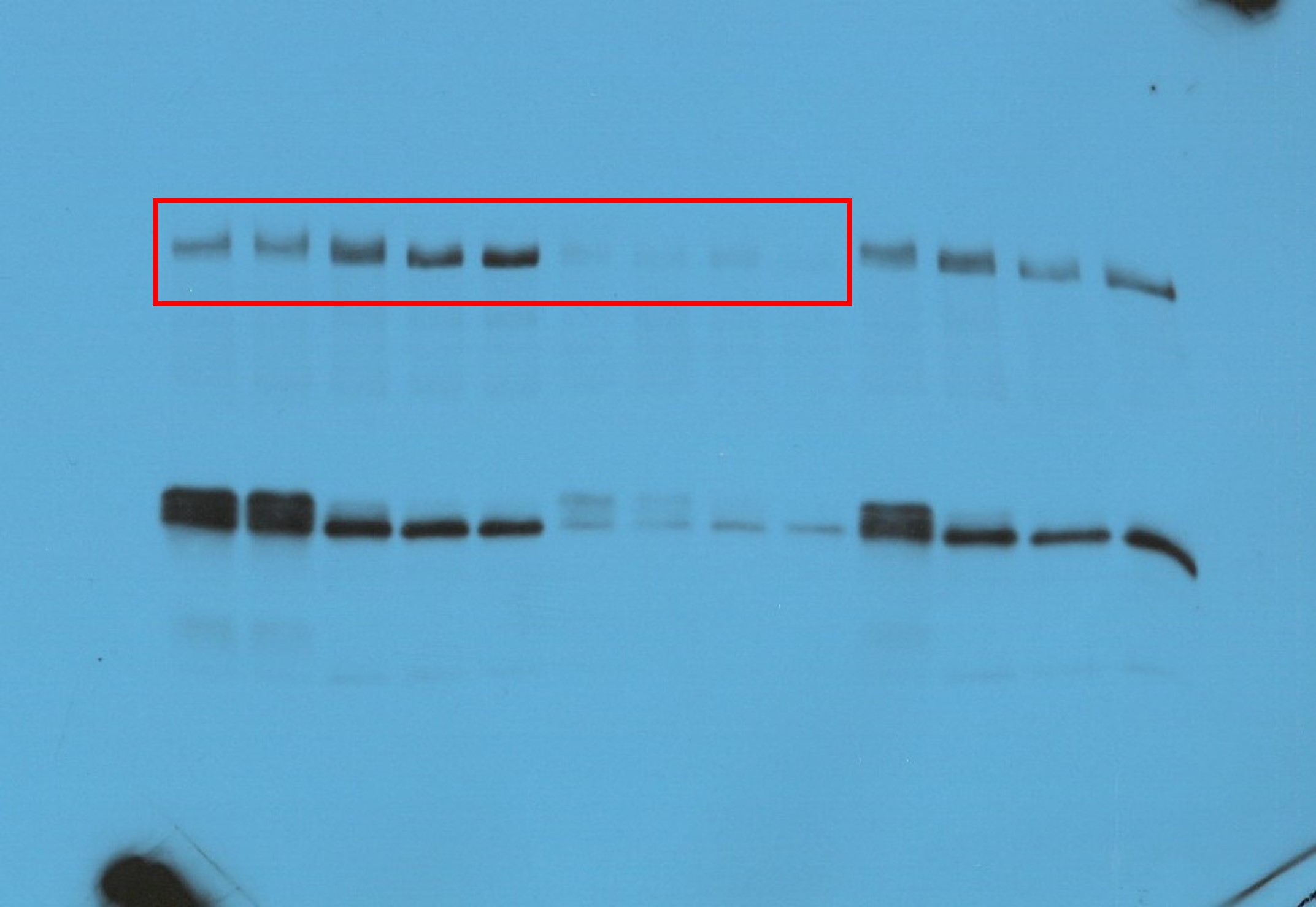

Supplement: Figure 1—figure supplement 1—source data 1. — (a) Western blots were used to detect expression of WC-1 in the indicated samples for Figure 1—figure supplement 1B (s: short exposure). (b) Figure with the area highlighted was used to develop the Figure 1—figure supplement 1B for WC-1 (s: short exposure). (c) Western blots were used to detect expression of WC-1 in the indicated samples for Figure 1—figure supplement 1B (l: long exposure). (d) Figure with the area highlighted was used to develop the Figure 1—figure supplement 1B for WC-1 (l: long exposure). (e) Western blots were used to detect expression of WC-2 in the indicated samples for Figure 1—figure supplement 1B (s: short exposure). (f) Figure with the area highlighted was used to develop the Figure 1—figure supplement 1B for WC-2 (s: short exposure). (g) Western blots were used to detect expression of WC-2 in the indicated samples for Figure 1—figure supplement 1B (l: long exposure). (h) Figure with the area highlighted was used to develop the Figure 1—figure supplement 1B for WC-2 (l: long exposure). (i) Ponceau S staining was used to detect loading control of the indicated samples for Figure 1—figure supplement 1B. (j) Figure with the area highlighted was used as LC of Figure 1—figure supplement 1B. [file elife-79765-fig1-figsupp1-data1.zip › 79765Figure1-S1B/Figure_1-Figure_supplement_1-source_data_1b.jpg]

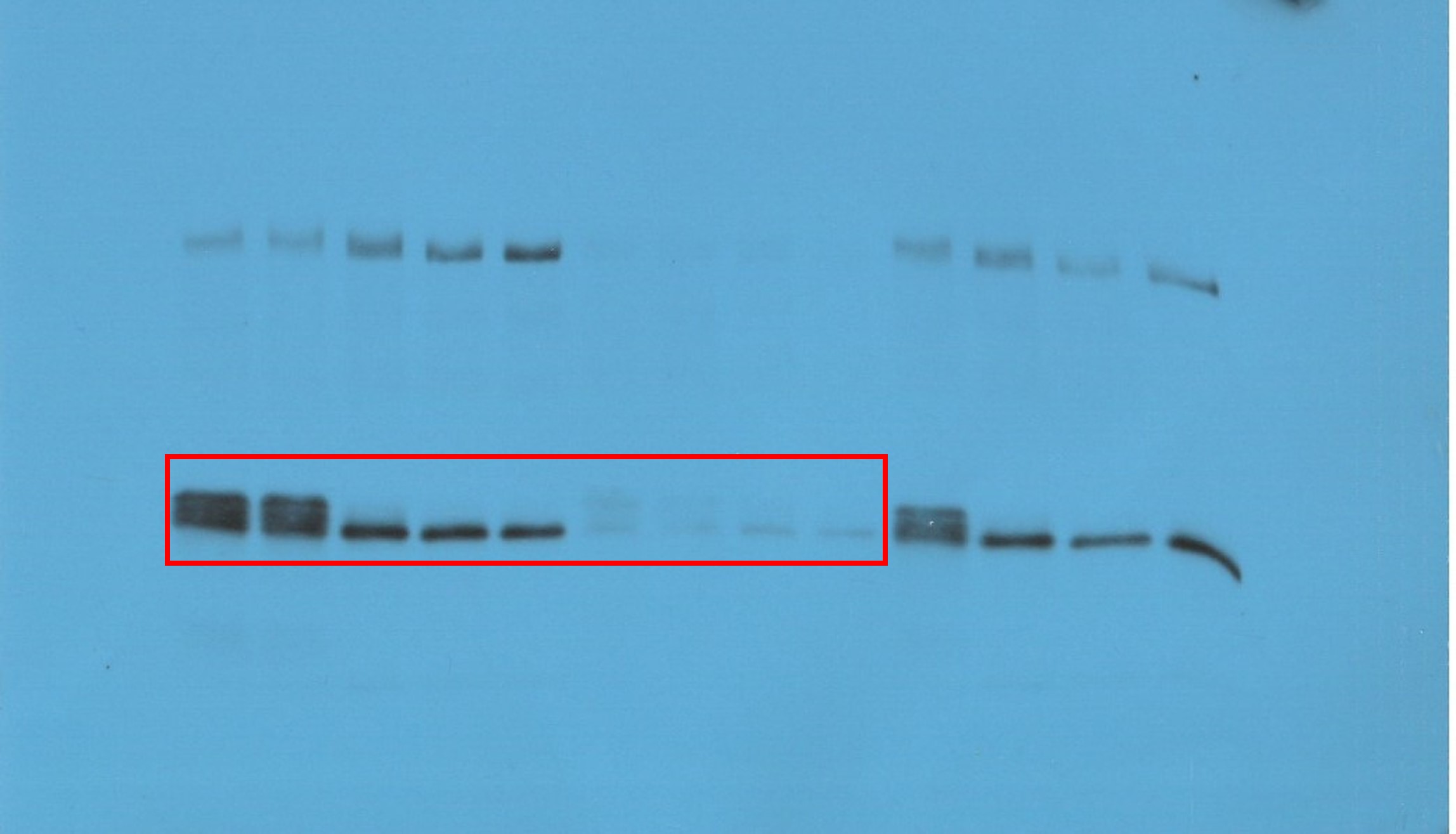

Supplement: Figure 1—figure supplement 1—source data 1. — (a) Western blots were used to detect expression of WC-1 in the indicated samples for Figure 1—figure supplement 1B (s: short exposure). (b) Figure with the area highlighted was used to develop the Figure 1—figure supplement 1B for WC-1 (s: short exposure). (c) Western blots were used to detect expression of WC-1 in the indicated samples for Figure 1—figure supplement 1B (l: long exposure). (d) Figure with the area highlighted was used to develop the Figure 1—figure supplement 1B for WC-1 (l: long exposure). (e) Western blots were used to detect expression of WC-2 in the indicated samples for Figure 1—figure supplement 1B (s: short exposure). (f) Figure with the area highlighted was used to develop the Figure 1—figure supplement 1B for WC-2 (s: short exposure). (g) Western blots were used to detect expression of WC-2 in the indicated samples for Figure 1—figure supplement 1B (l: long exposure). (h) Figure with the area highlighted was used to develop the Figure 1—figure supplement 1B for WC-2 (l: long exposure). (i) Ponceau S staining was used to detect loading control of the indicated samples for Figure 1—figure supplement 1B. (j) Figure with the area highlighted was used as LC of Figure 1—figure supplement 1B. [file elife-79765-fig1-figsupp1-data1.zip › 79765Figure1-S1B/Figure_1-Figure_supplement_1-source_data_1f.jpg]

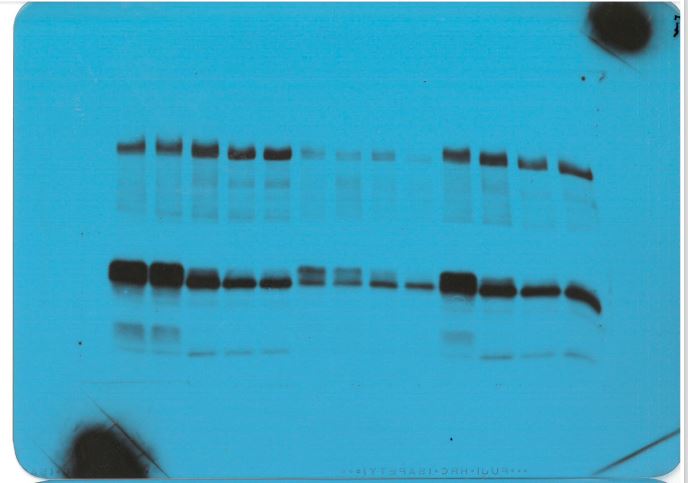

Supplement: Figure 1—figure supplement 1—source data 1. — (a) Western blots were used to detect expression of WC-1 in the indicated samples for Figure 1—figure supplement 1B (s: short exposure). (b) Figure with the area highlighted was used to develop the Figure 1—figure supplement 1B for WC-1 (s: short exposure). (c) Western blots were used to detect expression of WC-1 in the indicated samples for Figure 1—figure supplement 1B (l: long exposure). (d) Figure with the area highlighted was used to develop the Figure 1—figure supplement 1B for WC-1 (l: long exposure). (e) Western blots were used to detect expression of WC-2 in the indicated samples for Figure 1—figure supplement 1B (s: short exposure). (f) Figure with the area highlighted was used to develop the Figure 1—figure supplement 1B for WC-2 (s: short exposure). (g) Western blots were used to detect expression of WC-2 in the indicated samples for Figure 1—figure supplement 1B (l: long exposure). (h) Figure with the area highlighted was used to develop the Figure 1—figure supplement 1B for WC-2 (l: long exposure). (i) Ponceau S staining was used to detect loading control of the indicated samples for Figure 1—figure supplement 1B. (j) Figure with the area highlighted was used as LC of Figure 1—figure supplement 1B. [file elife-79765-fig1-figsupp1-data1.zip › 79765Figure1-S1B/Figure_1-Figure_supplement_1-source_data_1g.JPG]

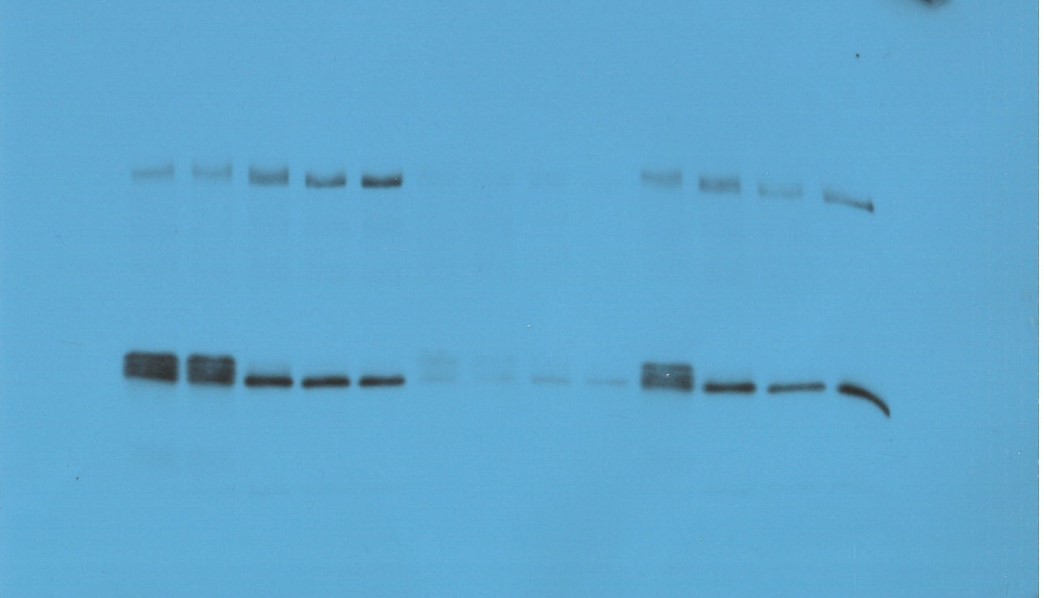

Supplement: Figure 1—figure supplement 1—source data 1. — (a) Western blots were used to detect expression of WC-1 in the indicated samples for Figure 1—figure supplement 1B (s: short exposure). (b) Figure with the area highlighted was used to develop the Figure 1—figure supplement 1B for WC-1 (s: short exposure). (c) Western blots were used to detect expression of WC-1 in the indicated samples for Figure 1—figure supplement 1B (l: long exposure). (d) Figure with the area highlighted was used to develop the Figure 1—figure supplement 1B for WC-1 (l: long exposure). (e) Western blots were used to detect expression of WC-2 in the indicated samples for Figure 1—figure supplement 1B (s: short exposure). (f) Figure with the area highlighted was used to develop the Figure 1—figure supplement 1B for WC-2 (s: short exposure). (g) Western blots were used to detect expression of WC-2 in the indicated samples for Figure 1—figure supplement 1B (l: long exposure). (h) Figure with the area highlighted was used to develop the Figure 1—figure supplement 1B for WC-2 (l: long exposure). (i) Ponceau S staining was used to detect loading control of the indicated samples for Figure 1—figure supplement 1B. (j) Figure with the area highlighted was used as LC of Figure 1—figure supplement 1B. [file elife-79765-fig1-figsupp1-data1.zip › 79765Figure1-S1B/Figure_1-Figure_supplement_1-source_data_1e.jpg]

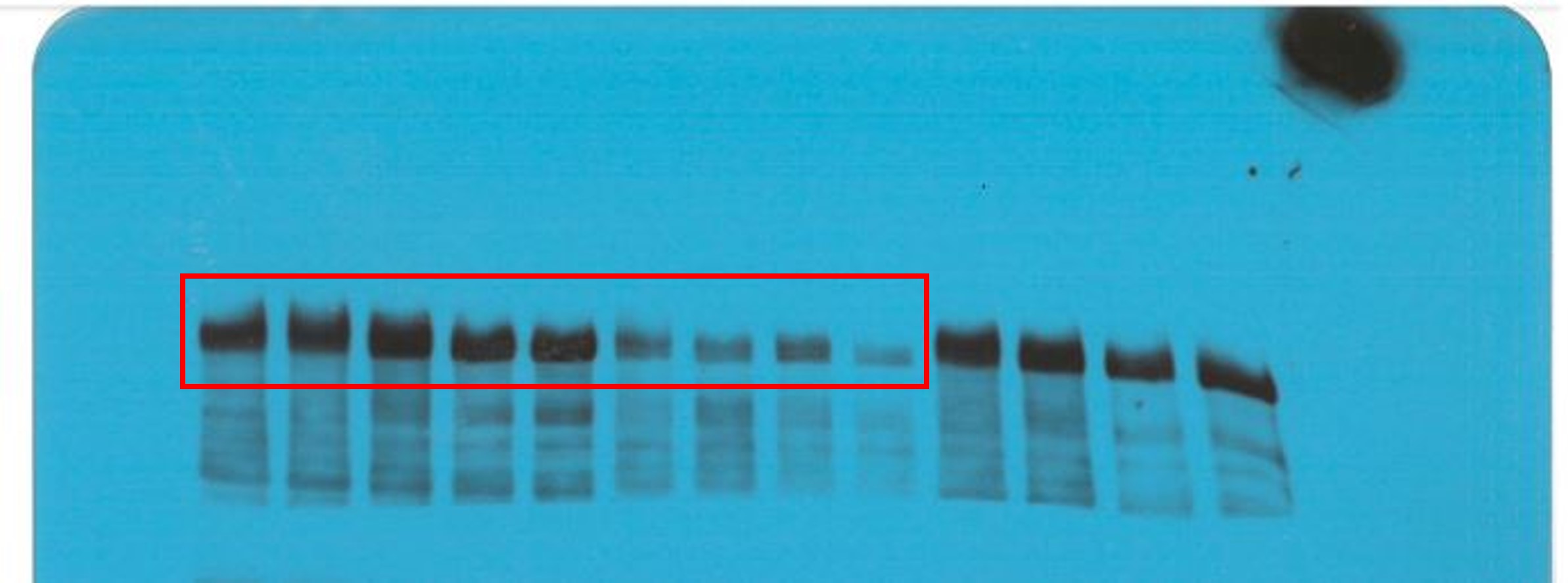

Supplement: Figure 1—figure supplement 1—source data 1. — (a) Western blots were used to detect expression of WC-1 in the indicated samples for Figure 1—figure supplement 1B (s: short exposure). (b) Figure with the area highlighted was used to develop the Figure 1—figure supplement 1B for WC-1 (s: short exposure). (c) Western blots were used to detect expression of WC-1 in the indicated samples for Figure 1—figure supplement 1B (l: long exposure). (d) Figure with the area highlighted was used to develop the Figure 1—figure supplement 1B for WC-1 (l: long exposure). (e) Western blots were used to detect expression of WC-2 in the indicated samples for Figure 1—figure supplement 1B (s: short exposure). (f) Figure with the area highlighted was used to develop the Figure 1—figure supplement 1B for WC-2 (s: short exposure). (g) Western blots were used to detect expression of WC-2 in the indicated samples for Figure 1—figure supplement 1B (l: long exposure). (h) Figure with the area highlighted was used to develop the Figure 1—figure supplement 1B for WC-2 (l: long exposure). (i) Ponceau S staining was used to detect loading control of the indicated samples for Figure 1—figure supplement 1B. (j) Figure with the area highlighted was used as LC of Figure 1—figure supplement 1B. [file elife-79765-fig1-figsupp1-data1.zip › 79765Figure1-S1B/Figure_1-Figure_supplement_1-source_data_1d.JPG]

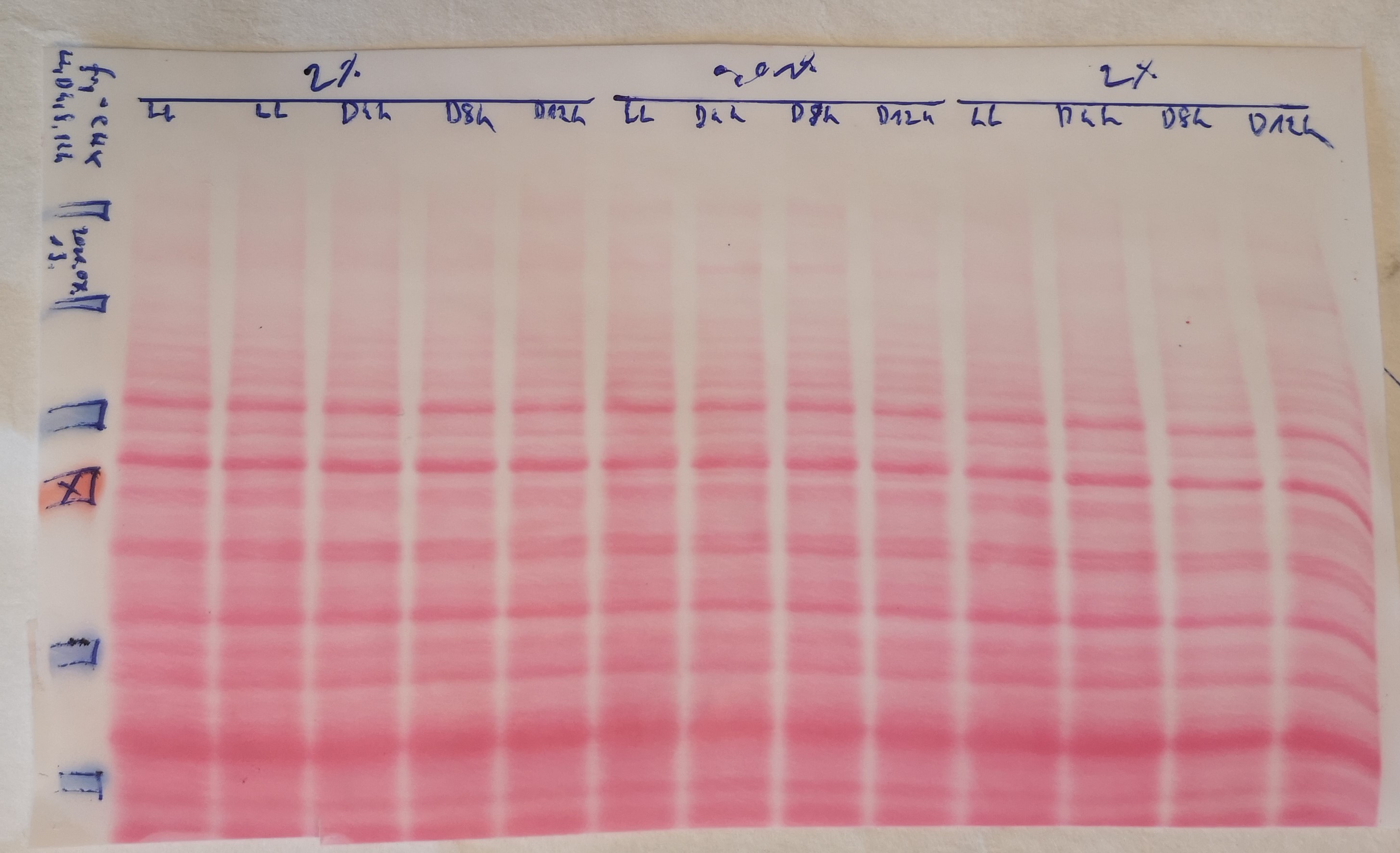

Supplement: Figure 1—figure supplement 1—source data 1. — (a) Western blots were used to detect expression of WC-1 in the indicated samples for Figure 1—figure supplement 1B (s: short exposure). (b) Figure with the area highlighted was used to develop the Figure 1—figure supplement 1B for WC-1 (s: short exposure). (c) Western blots were used to detect expression of WC-1 in the indicated samples for Figure 1—figure supplement 1B (l: long exposure). (d) Figure with the area highlighted was used to develop the Figure 1—figure supplement 1B for WC-1 (l: long exposure). (e) Western blots were used to detect expression of WC-2 in the indicated samples for Figure 1—figure supplement 1B (s: short exposure). (f) Figure with the area highlighted was used to develop the Figure 1—figure supplement 1B for WC-2 (s: short exposure). (g) Western blots were used to detect expression of WC-2 in the indicated samples for Figure 1—figure supplement 1B (l: long exposure). (h) Figure with the area highlighted was used to develop the Figure 1—figure supplement 1B for WC-2 (l: long exposure). (i) Ponceau S staining was used to detect loading control of the indicated samples for Figure 1—figure supplement 1B. (j) Figure with the area highlighted was used as LC of Figure 1—figure supplement 1B. [file elife-79765-fig1-figsupp1-data1.zip › 79765Figure1-S1B/Figure_1-Figure_supplement_1-source_data_1i.jpg]

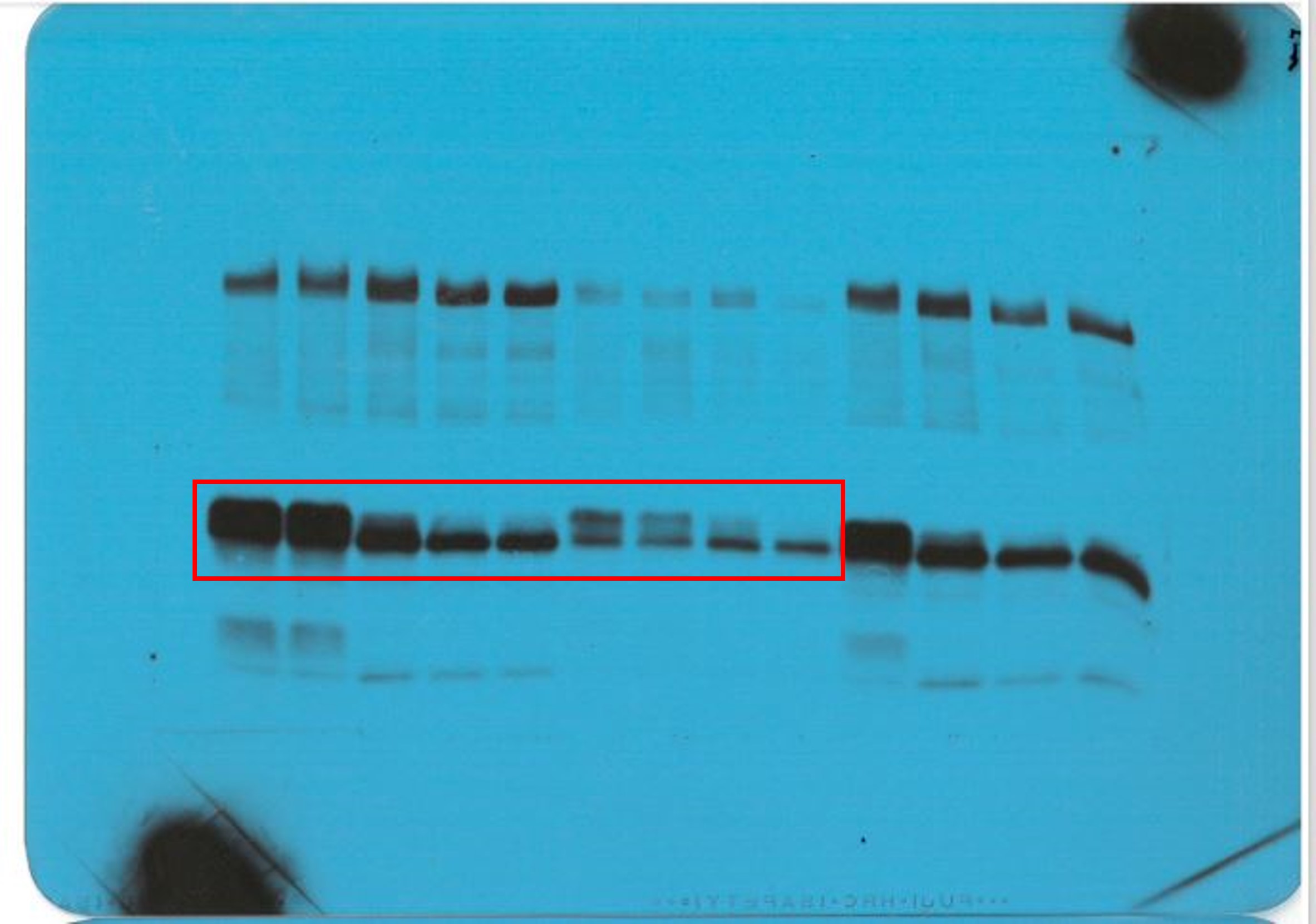

Supplement: Figure 1—figure supplement 1—source data 1. — (a) Western blots were used to detect expression of WC-1 in the indicated samples for Figure 1—figure supplement 1B (s: short exposure). (b) Figure with the area highlighted was used to develop the Figure 1—figure supplement 1B for WC-1 (s: short exposure). (c) Western blots were used to detect expression of WC-1 in the indicated samples for Figure 1—figure supplement 1B (l: long exposure). (d) Figure with the area highlighted was used to develop the Figure 1—figure supplement 1B for WC-1 (l: long exposure). (e) Western blots were used to detect expression of WC-2 in the indicated samples for Figure 1—figure supplement 1B (s: short exposure). (f) Figure with the area highlighted was used to develop the Figure 1—figure supplement 1B for WC-2 (s: short exposure). (g) Western blots were used to detect expression of WC-2 in the indicated samples for Figure 1—figure supplement 1B (l: long exposure). (h) Figure with the area highlighted was used to develop the Figure 1—figure supplement 1B for WC-2 (l: long exposure). (i) Ponceau S staining was used to detect loading control of the indicated samples for Figure 1—figure supplement 1B. (j) Figure with the area highlighted was used as LC of Figure 1—figure supplement 1B. [file elife-79765-fig1-figsupp1-data1.zip › 79765Figure1-S1B/Figure_1-Figure_supplement_1-source_data_1h.jpg]

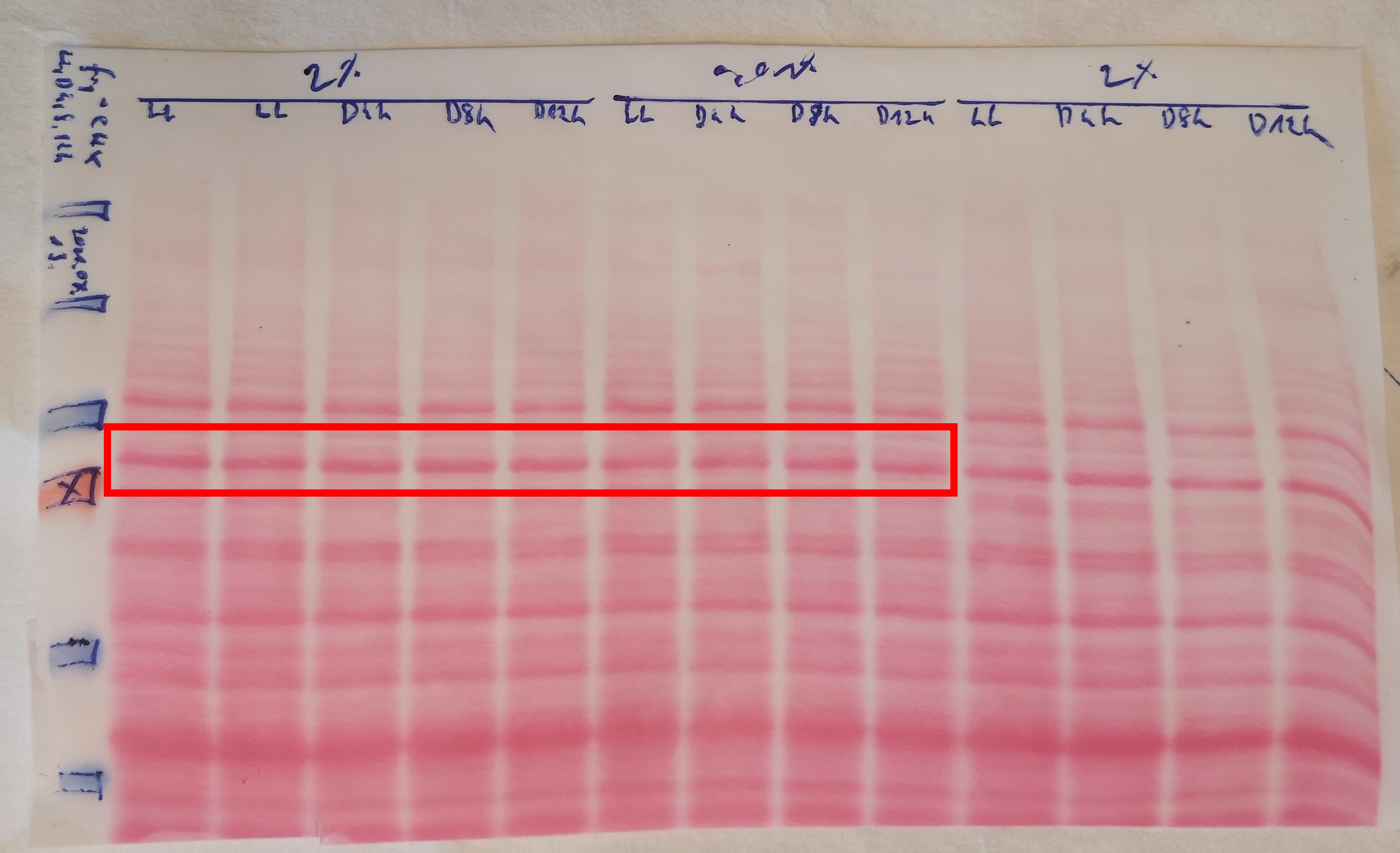

Supplement: Figure 1—figure supplement 1—source data 1. — (a) Western blots were used to detect expression of WC-1 in the indicated samples for Figure 1—figure supplement 1B (s: short exposure). (b) Figure with the area highlighted was used to develop the Figure 1—figure supplement 1B for WC-1 (s: short exposure). (c) Western blots were used to detect expression of WC-1 in the indicated samples for Figure 1—figure supplement 1B (l: long exposure). (d) Figure with the area highlighted was used to develop the Figure 1—figure supplement 1B for WC-1 (l: long exposure). (e) Western blots were used to detect expression of WC-2 in the indicated samples for Figure 1—figure supplement 1B (s: short exposure). (f) Figure with the area highlighted was used to develop the Figure 1—figure supplement 1B for WC-2 (s: short exposure). (g) Western blots were used to detect expression of WC-2 in the indicated samples for Figure 1—figure supplement 1B (l: long exposure). (h) Figure with the area highlighted was used to develop the Figure 1—figure supplement 1B for WC-2 (l: long exposure). (i) Ponceau S staining was used to detect loading control of the indicated samples for Figure 1—figure supplement 1B. (j) Figure with the area highlighted was used as LC of Figure 1—figure supplement 1B. [file elife-79765-fig1-figsupp1-data1.zip › 79765Figure1-S1B/Figure_1-Figure_supplement_1-source_data_1j.jpg]

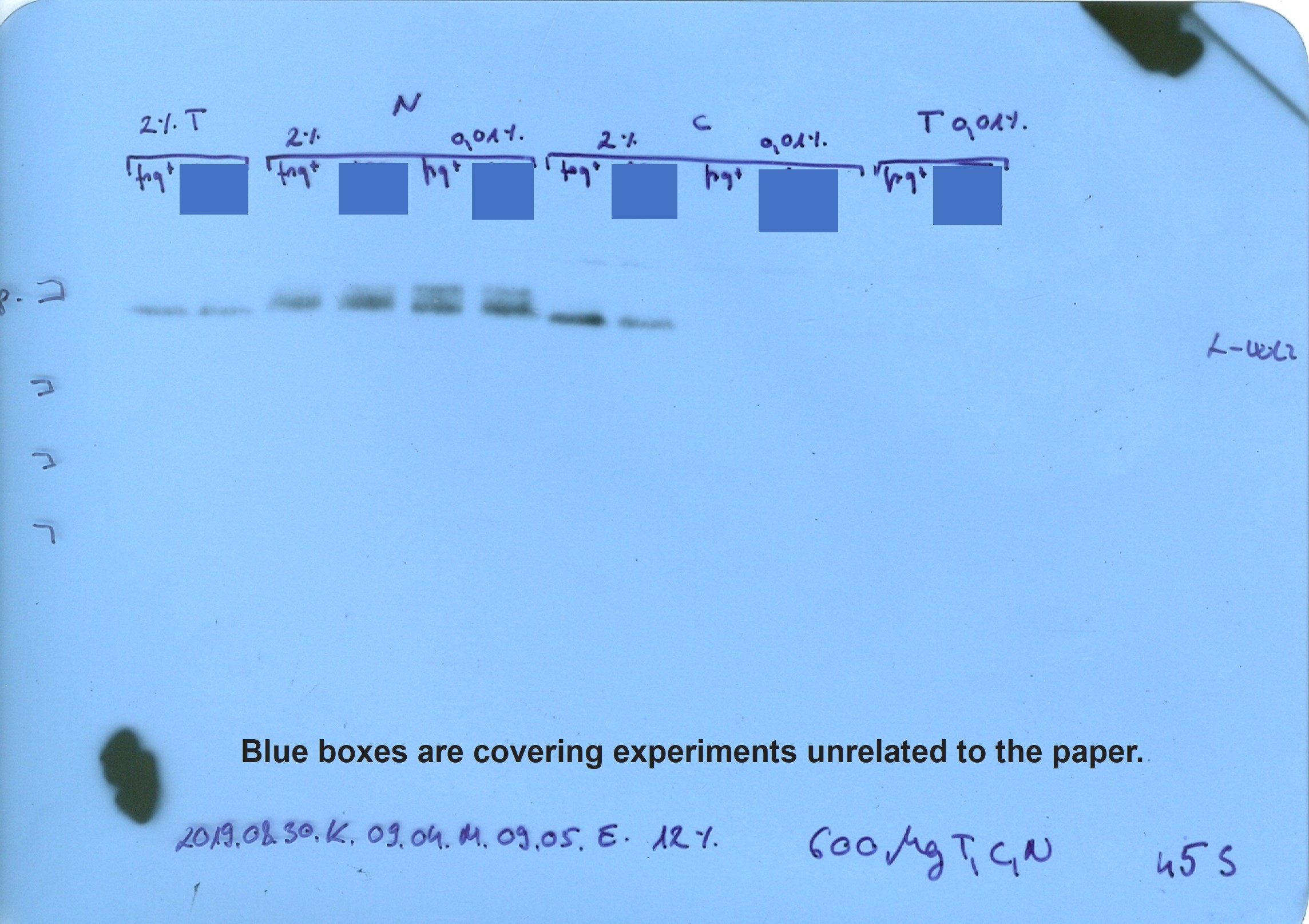

Supplement: Figure 2—source data 1. — (a) Western blots were used to detect expression of FRQ in the indicated samples for Figure 2B (s: short exposure). (b) Figure with the area highlighted was used to develop the Figure 2B for FRQ (s: short exposure). (c) Western blots were used to detect expression of FRQ in the indicated samples for Figure 2B (l: long exposure). (d) Figure with the area highlighted was used to develop the Figure 2B for FRQ (l: long exposure). (e) Western blots were used to detect expression of WC-1 in the indicated samples for Figure 2B (s: short exposure). (f) Figure with the area highlighted was used to develop the Figure 2B for WC-1 (s: short exposure). (g) Western blots were used to detect expression of WC-1 in the indicated samples for Figure 2B (l: long exposure). (h) Figure with the area highlighted was used to develop the Figure 2B for WC-1 (l: long exposure). (i) Western blots were used to detect expression of WC-2 in the indicated samples for Figure 2B (s: short exposure). (j) Figure with the area highlighted was used to develop the Figure 2B for WC-2 (s: short exposure). (k) Western blots were used to detect expression of WC-2 in the indicated samples for Figure 2B (l: long exposure). (l) Figure with the area highlighted was used to develop the Figure 2B for WC-2 (l: long exposure). (m) Ponceau S staining was used to detect loading control of the indicated samples for Figure 2B. (n) Figure with the area highlighted was used as LC for Figure 2B. [file elife-79765-fig2-data1.zip › 79765Figure2B/Figure_2-source_data_1i.jpg]

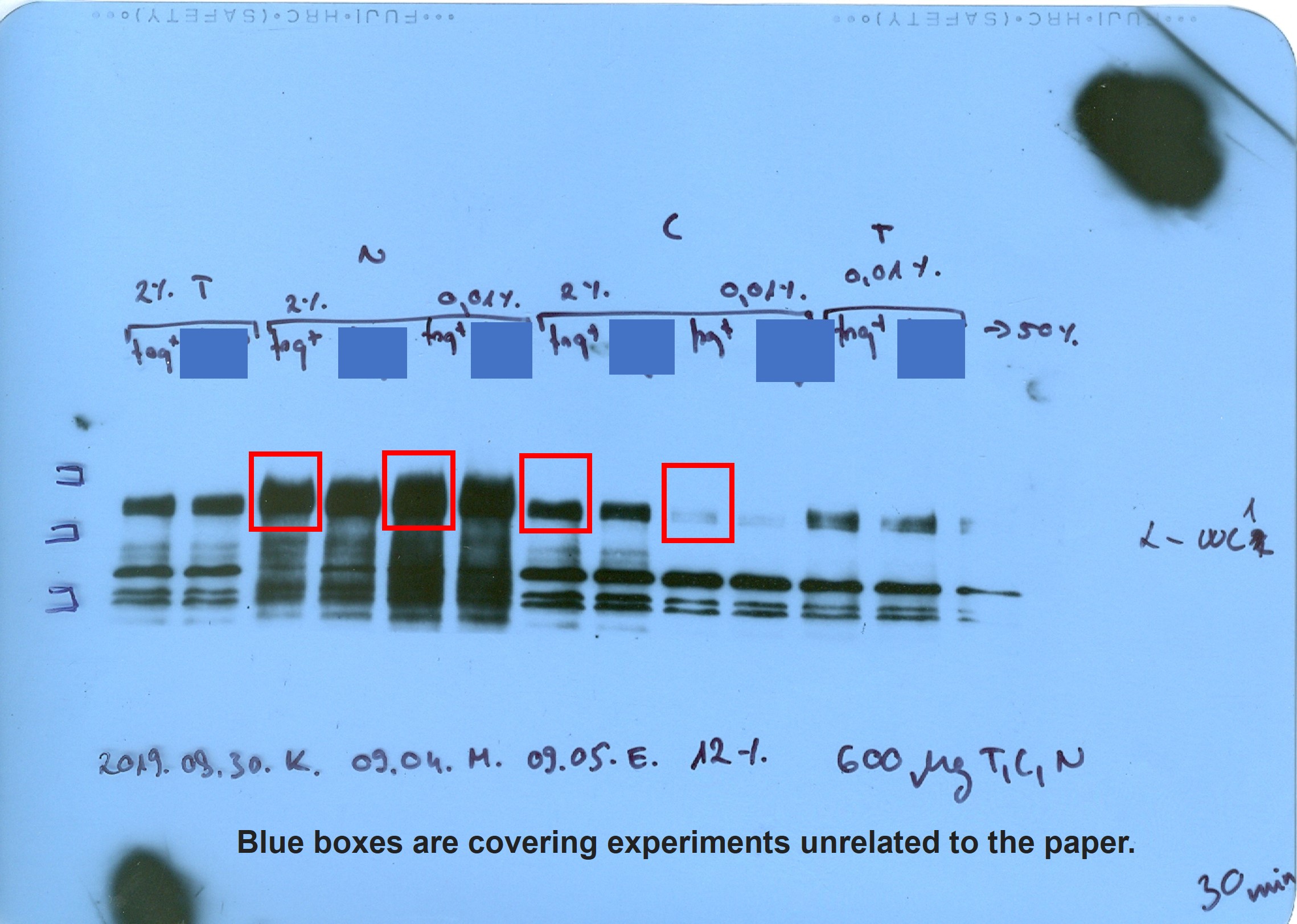

Supplement: Figure 2—source data 1. — (a) Western blots were used to detect expression of FRQ in the indicated samples for Figure 2B (s: short exposure). (b) Figure with the area highlighted was used to develop the Figure 2B for FRQ (s: short exposure). (c) Western blots were used to detect expression of FRQ in the indicated samples for Figure 2B (l: long exposure). (d) Figure with the area highlighted was used to develop the Figure 2B for FRQ (l: long exposure). (e) Western blots were used to detect expression of WC-1 in the indicated samples for Figure 2B (s: short exposure). (f) Figure with the area highlighted was used to develop the Figure 2B for WC-1 (s: short exposure). (g) Western blots were used to detect expression of WC-1 in the indicated samples for Figure 2B (l: long exposure). (h) Figure with the area highlighted was used to develop the Figure 2B for WC-1 (l: long exposure). (i) Western blots were used to detect expression of WC-2 in the indicated samples for Figure 2B (s: short exposure). (j) Figure with the area highlighted was used to develop the Figure 2B for WC-2 (s: short exposure). (k) Western blots were used to detect expression of WC-2 in the indicated samples for Figure 2B (l: long exposure). (l) Figure with the area highlighted was used to develop the Figure 2B for WC-2 (l: long exposure). (m) Ponceau S staining was used to detect loading control of the indicated samples for Figure 2B. (n) Figure with the area highlighted was used as LC for Figure 2B. [file elife-79765-fig2-data1.zip › 79765Figure2B/Figure_2-source_data_1h.jpg]

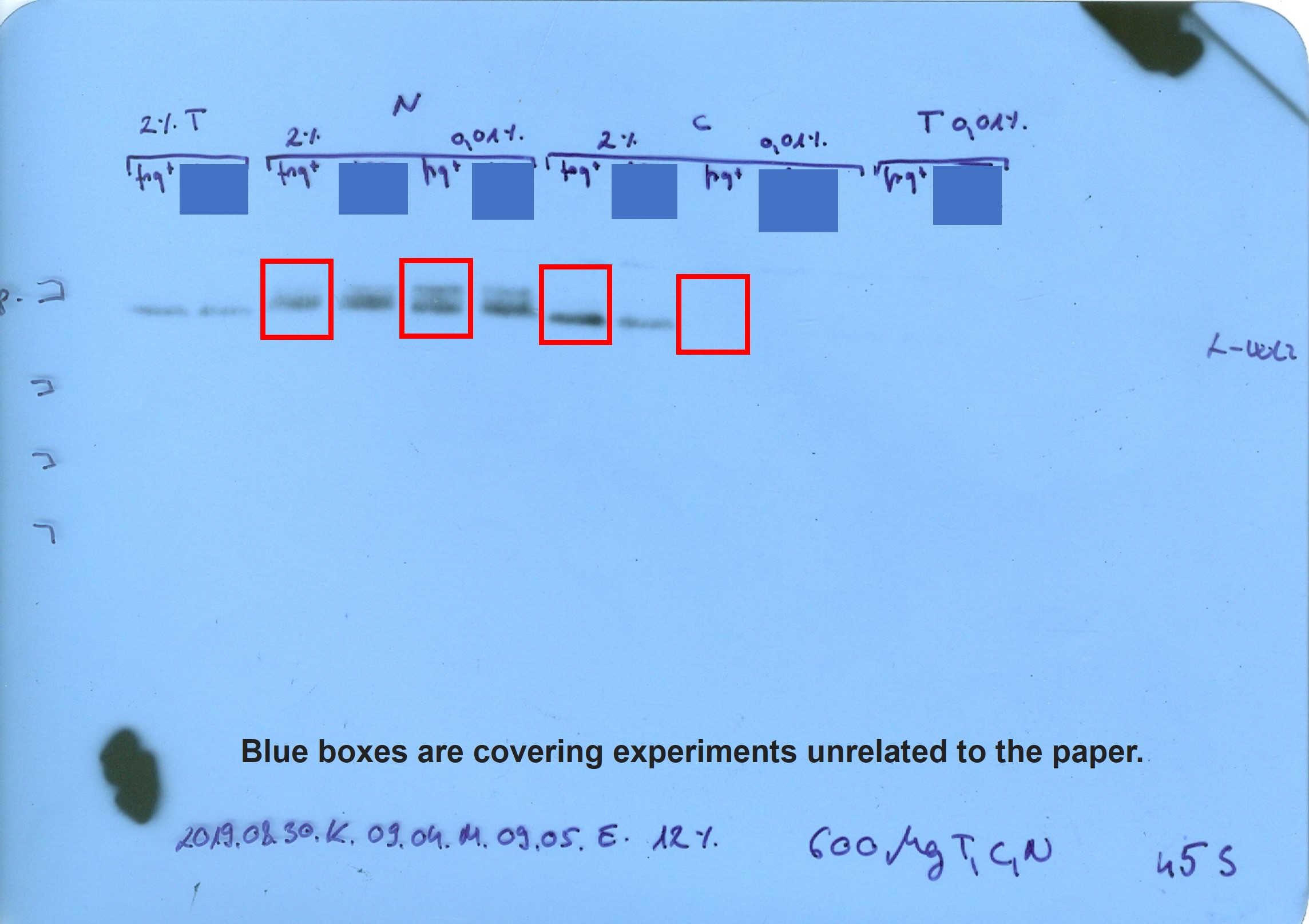

Supplement: Figure 2—source data 1. — (a) Western blots were used to detect expression of FRQ in the indicated samples for Figure 2B (s: short exposure). (b) Figure with the area highlighted was used to develop the Figure 2B for FRQ (s: short exposure). (c) Western blots were used to detect expression of FRQ in the indicated samples for Figure 2B (l: long exposure). (d) Figure with the area highlighted was used to develop the Figure 2B for FRQ (l: long exposure). (e) Western blots were used to detect expression of WC-1 in the indicated samples for Figure 2B (s: short exposure). (f) Figure with the area highlighted was used to develop the Figure 2B for WC-1 (s: short exposure). (g) Western blots were used to detect expression of WC-1 in the indicated samples for Figure 2B (l: long exposure). (h) Figure with the area highlighted was used to develop the Figure 2B for WC-1 (l: long exposure). (i) Western blots were used to detect expression of WC-2 in the indicated samples for Figure 2B (s: short exposure). (j) Figure with the area highlighted was used to develop the Figure 2B for WC-2 (s: short exposure). (k) Western blots were used to detect expression of WC-2 in the indicated samples for Figure 2B (l: long exposure). (l) Figure with the area highlighted was used to develop the Figure 2B for WC-2 (l: long exposure). (m) Ponceau S staining was used to detect loading control of the indicated samples for Figure 2B. (n) Figure with the area highlighted was used as LC for Figure 2B. [file elife-79765-fig2-data1.zip › 79765Figure2B/Figure_2-source_data_1j.jpg]

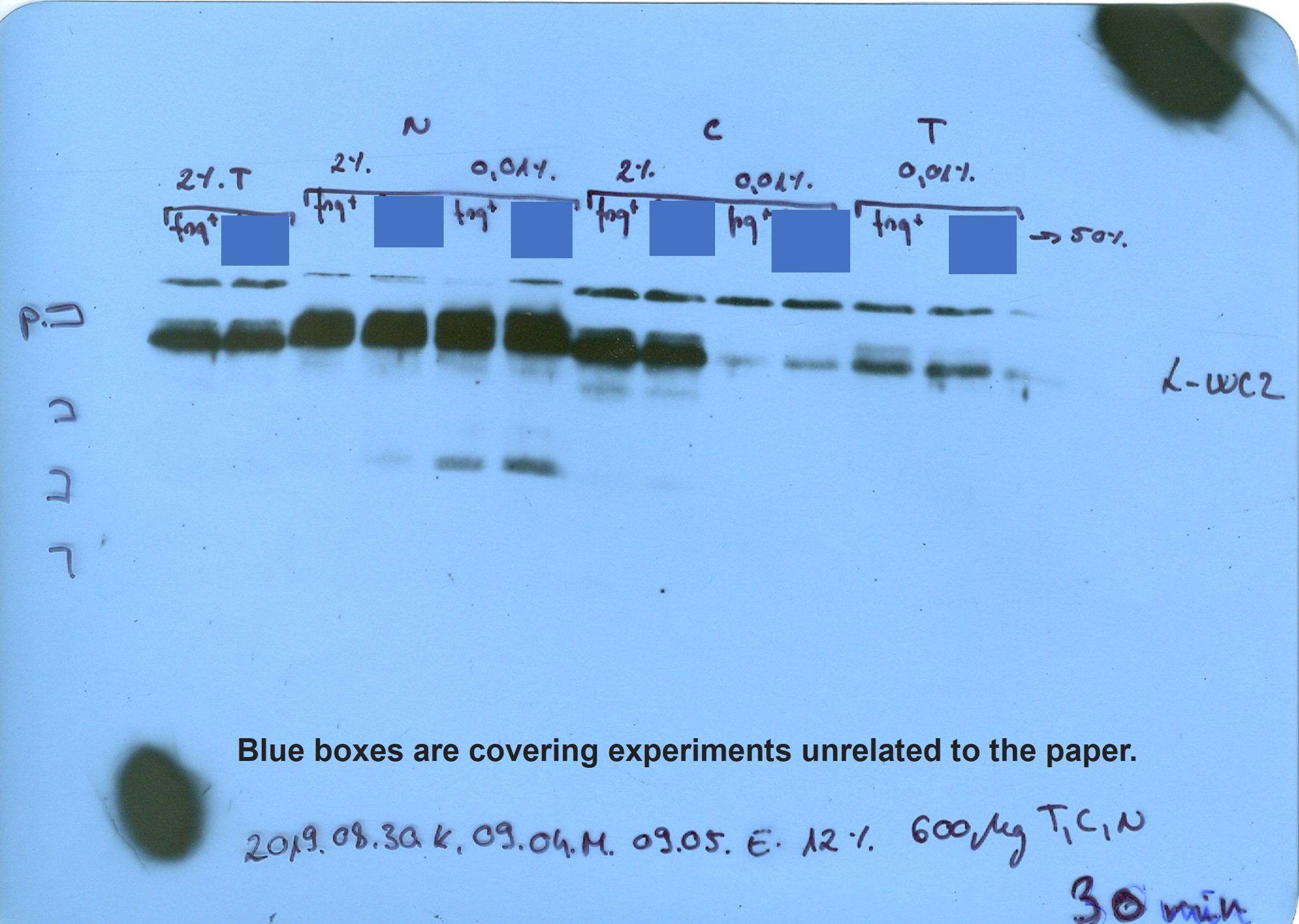

Supplement: Figure 2—source data 1. — (a) Western blots were used to detect expression of FRQ in the indicated samples for Figure 2B (s: short exposure). (b) Figure with the area highlighted was used to develop the Figure 2B for FRQ (s: short exposure). (c) Western blots were used to detect expression of FRQ in the indicated samples for Figure 2B (l: long exposure). (d) Figure with the area highlighted was used to develop the Figure 2B for FRQ (l: long exposure). (e) Western blots were used to detect expression of WC-1 in the indicated samples for Figure 2B (s: short exposure). (f) Figure with the area highlighted was used to develop the Figure 2B for WC-1 (s: short exposure). (g) Western blots were used to detect expression of WC-1 in the indicated samples for Figure 2B (l: long exposure). (h) Figure with the area highlighted was used to develop the Figure 2B for WC-1 (l: long exposure). (i) Western blots were used to detect expression of WC-2 in the indicated samples for Figure 2B (s: short exposure). (j) Figure with the area highlighted was used to develop the Figure 2B for WC-2 (s: short exposure). (k) Western blots were used to detect expression of WC-2 in the indicated samples for Figure 2B (l: long exposure). (l) Figure with the area highlighted was used to develop the Figure 2B for WC-2 (l: long exposure). (m) Ponceau S staining was used to detect loading control of the indicated samples for Figure 2B. (n) Figure with the area highlighted was used as LC for Figure 2B. [file elife-79765-fig2-data1.zip › 79765Figure2B/Figure_2-source_data_1k.jpg]

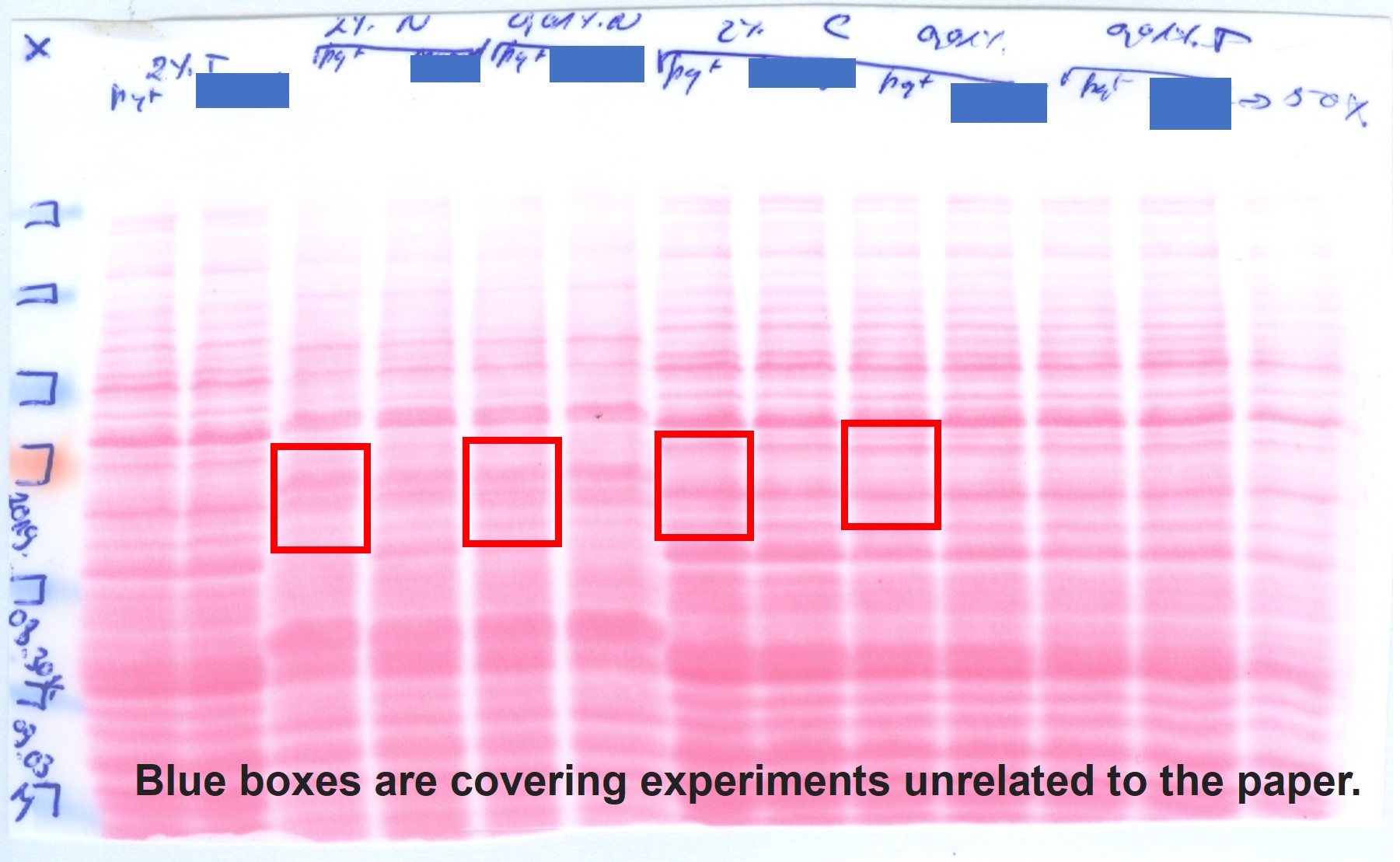

Supplement: Figure 2—source data 1. — (a) Western blots were used to detect expression of FRQ in the indicated samples for Figure 2B (s: short exposure). (b) Figure with the area highlighted was used to develop the Figure 2B for FRQ (s: short exposure). (c) Western blots were used to detect expression of FRQ in the indicated samples for Figure 2B (l: long exposure). (d) Figure with the area highlighted was used to develop the Figure 2B for FRQ (l: long exposure). (e) Western blots were used to detect expression of WC-1 in the indicated samples for Figure 2B (s: short exposure). (f) Figure with the area highlighted was used to develop the Figure 2B for WC-1 (s: short exposure). (g) Western blots were used to detect expression of WC-1 in the indicated samples for Figure 2B (l: long exposure). (h) Figure with the area highlighted was used to develop the Figure 2B for WC-1 (l: long exposure). (i) Western blots were used to detect expression of WC-2 in the indicated samples for Figure 2B (s: short exposure). (j) Figure with the area highlighted was used to develop the Figure 2B for WC-2 (s: short exposure). (k) Western blots were used to detect expression of WC-2 in the indicated samples for Figure 2B (l: long exposure). (l) Figure with the area highlighted was used to develop the Figure 2B for WC-2 (l: long exposure). (m) Ponceau S staining was used to detect loading control of the indicated samples for Figure 2B. (n) Figure with the area highlighted was used as LC for Figure 2B. [file elife-79765-fig2-data1.zip › 79765Figure2B/Figure_2-source_data_1n.jpg]

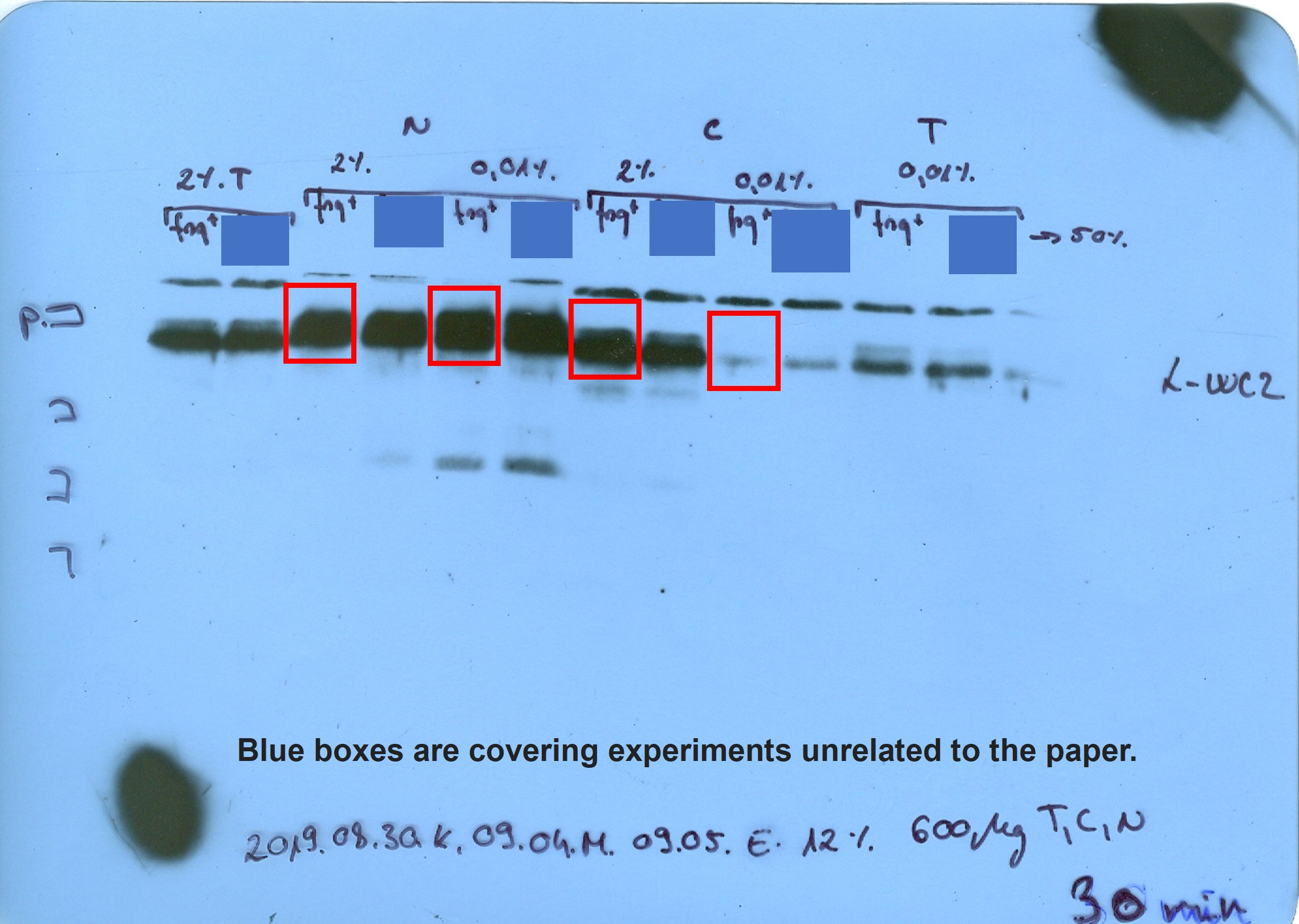

Supplement: Figure 2—source data 1. — (a) Western blots were used to detect expression of FRQ in the indicated samples for Figure 2B (s: short exposure). (b) Figure with the area highlighted was used to develop the Figure 2B for FRQ (s: short exposure). (c) Western blots were used to detect expression of FRQ in the indicated samples for Figure 2B (l: long exposure). (d) Figure with the area highlighted was used to develop the Figure 2B for FRQ (l: long exposure). (e) Western blots were used to detect expression of WC-1 in the indicated samples for Figure 2B (s: short exposure). (f) Figure with the area highlighted was used to develop the Figure 2B for WC-1 (s: short exposure). (g) Western blots were used to detect expression of WC-1 in the indicated samples for Figure 2B (l: long exposure). (h) Figure with the area highlighted was used to develop the Figure 2B for WC-1 (l: long exposure). (i) Western blots were used to detect expression of WC-2 in the indicated samples for Figure 2B (s: short exposure). (j) Figure with the area highlighted was used to develop the Figure 2B for WC-2 (s: short exposure). (k) Western blots were used to detect expression of WC-2 in the indicated samples for Figure 2B (l: long exposure). (l) Figure with the area highlighted was used to develop the Figure 2B for WC-2 (l: long exposure). (m) Ponceau S staining was used to detect loading control of the indicated samples for Figure 2B. (n) Figure with the area highlighted was used as LC for Figure 2B. [file elife-79765-fig2-data1.zip › 79765Figure2B/Figure_2-source_data_1l.jpg]

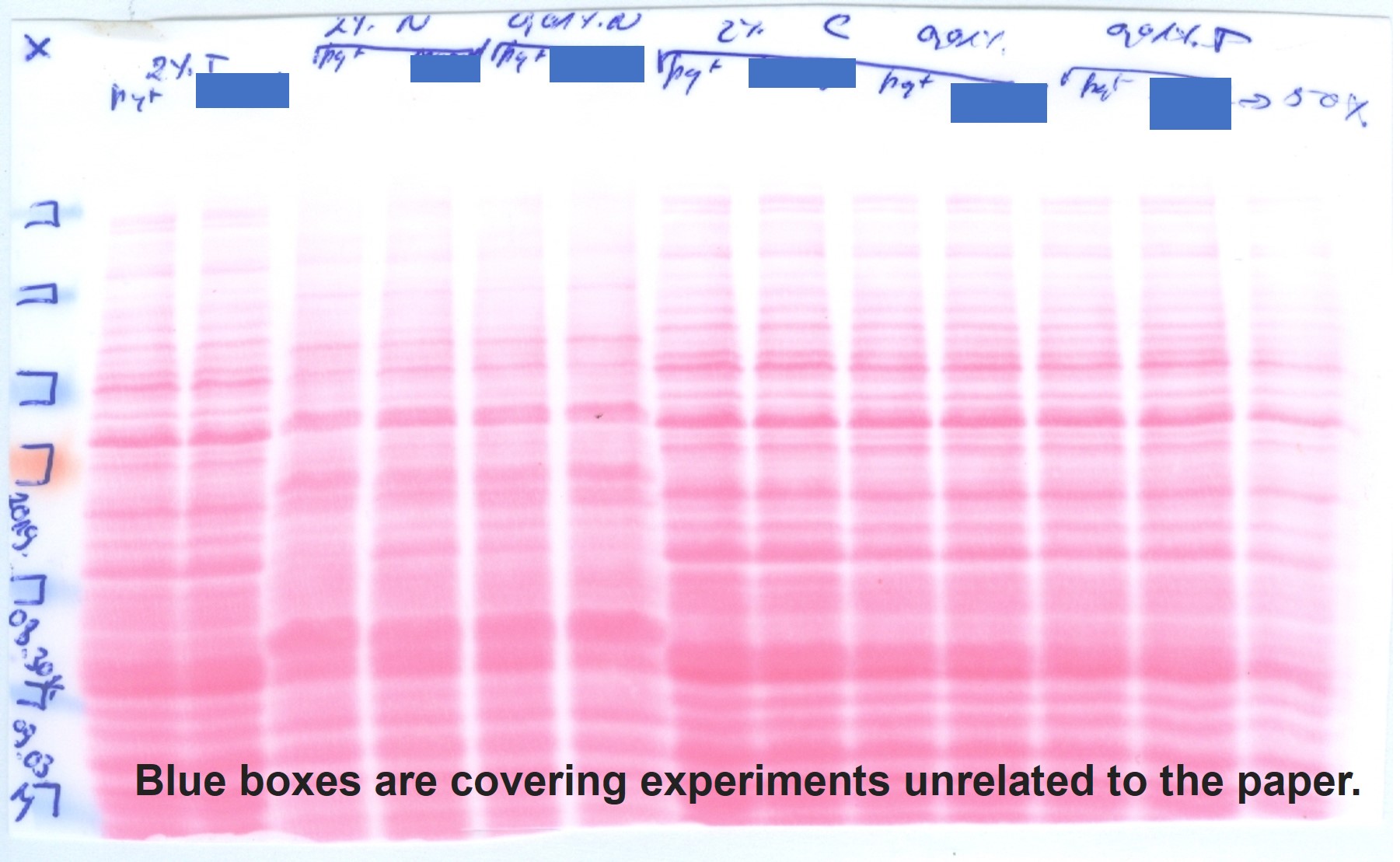

Supplement: Figure 2—source data 1. — (a) Western blots were used to detect expression of FRQ in the indicated samples for Figure 2B (s: short exposure). (b) Figure with the area highlighted was used to develop the Figure 2B for FRQ (s: short exposure). (c) Western blots were used to detect expression of FRQ in the indicated samples for Figure 2B (l: long exposure). (d) Figure with the area highlighted was used to develop the Figure 2B for FRQ (l: long exposure). (e) Western blots were used to detect expression of WC-1 in the indicated samples for Figure 2B (s: short exposure). (f) Figure with the area highlighted was used to develop the Figure 2B for WC-1 (s: short exposure). (g) Western blots were used to detect expression of WC-1 in the indicated samples for Figure 2B (l: long exposure). (h) Figure with the area highlighted was used to develop the Figure 2B for WC-1 (l: long exposure). (i) Western blots were used to detect expression of WC-2 in the indicated samples for Figure 2B (s: short exposure). (j) Figure with the area highlighted was used to develop the Figure 2B for WC-2 (s: short exposure). (k) Western blots were used to detect expression of WC-2 in the indicated samples for Figure 2B (l: long exposure). (l) Figure with the area highlighted was used to develop the Figure 2B for WC-2 (l: long exposure). (m) Ponceau S staining was used to detect loading control of the indicated samples for Figure 2B. (n) Figure with the area highlighted was used as LC for Figure 2B. [file elife-79765-fig2-data1.zip › 79765Figure2B/Figure_2-source_data_1m.jpg]

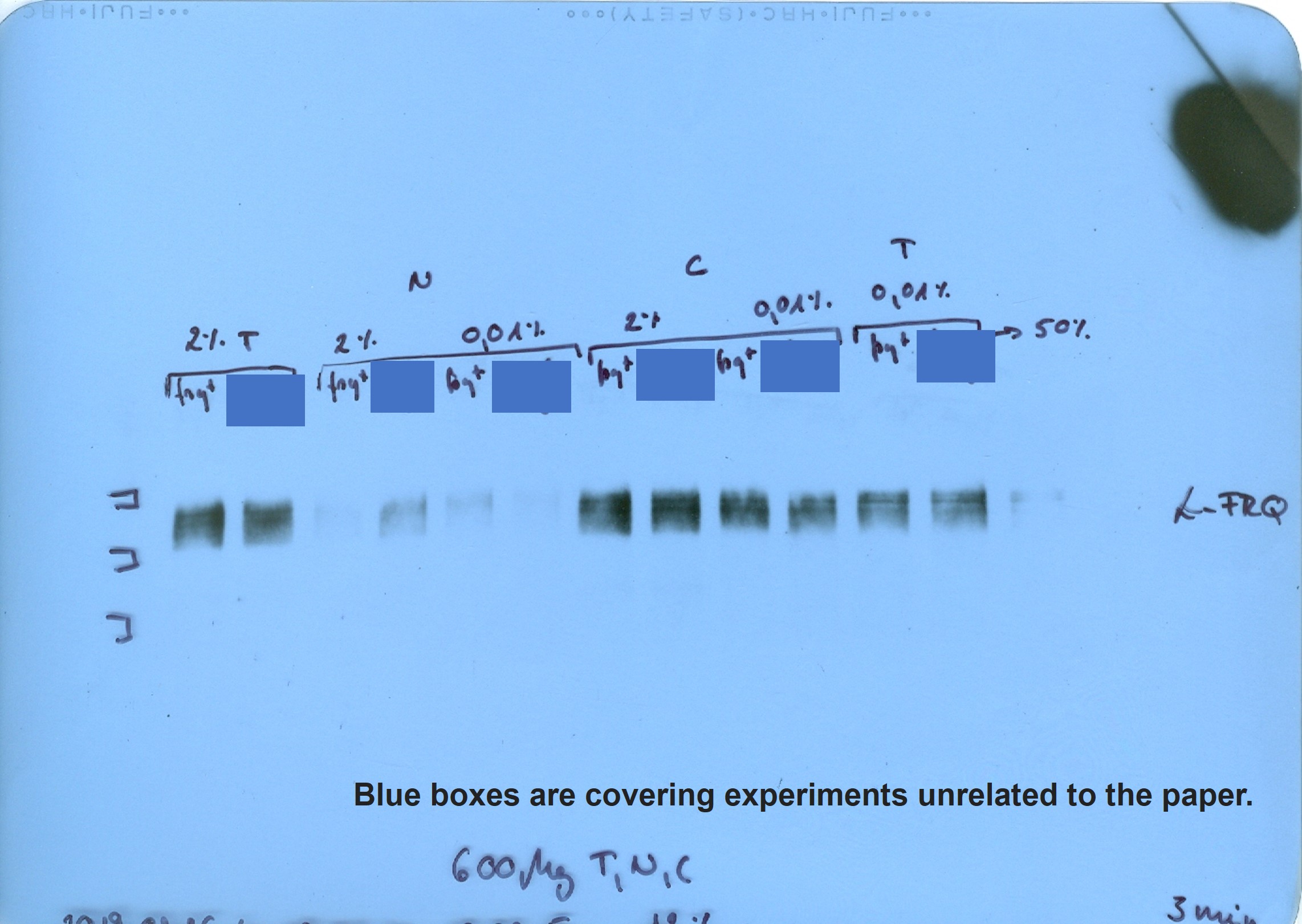

Supplement: Figure 2—source data 1. — (a) Western blots were used to detect expression of FRQ in the indicated samples for Figure 2B (s: short exposure). (b) Figure with the area highlighted was used to develop the Figure 2B for FRQ (s: short exposure). (c) Western blots were used to detect expression of FRQ in the indicated samples for Figure 2B (l: long exposure). (d) Figure with the area highlighted was used to develop the Figure 2B for FRQ (l: long exposure). (e) Western blots were used to detect expression of WC-1 in the indicated samples for Figure 2B (s: short exposure). (f) Figure with the area highlighted was used to develop the Figure 2B for WC-1 (s: short exposure). (g) Western blots were used to detect expression of WC-1 in the indicated samples for Figure 2B (l: long exposure). (h) Figure with the area highlighted was used to develop the Figure 2B for WC-1 (l: long exposure). (i) Western blots were used to detect expression of WC-2 in the indicated samples for Figure 2B (s: short exposure). (j) Figure with the area highlighted was used to develop the Figure 2B for WC-2 (s: short exposure). (k) Western blots were used to detect expression of WC-2 in the indicated samples for Figure 2B (l: long exposure). (l) Figure with the area highlighted was used to develop the Figure 2B for WC-2 (l: long exposure). (m) Ponceau S staining was used to detect loading control of the indicated samples for Figure 2B. (n) Figure with the area highlighted was used as LC for Figure 2B. [file elife-79765-fig2-data1.zip › 79765Figure2B/Figure_2-source_data_1a.jpg]

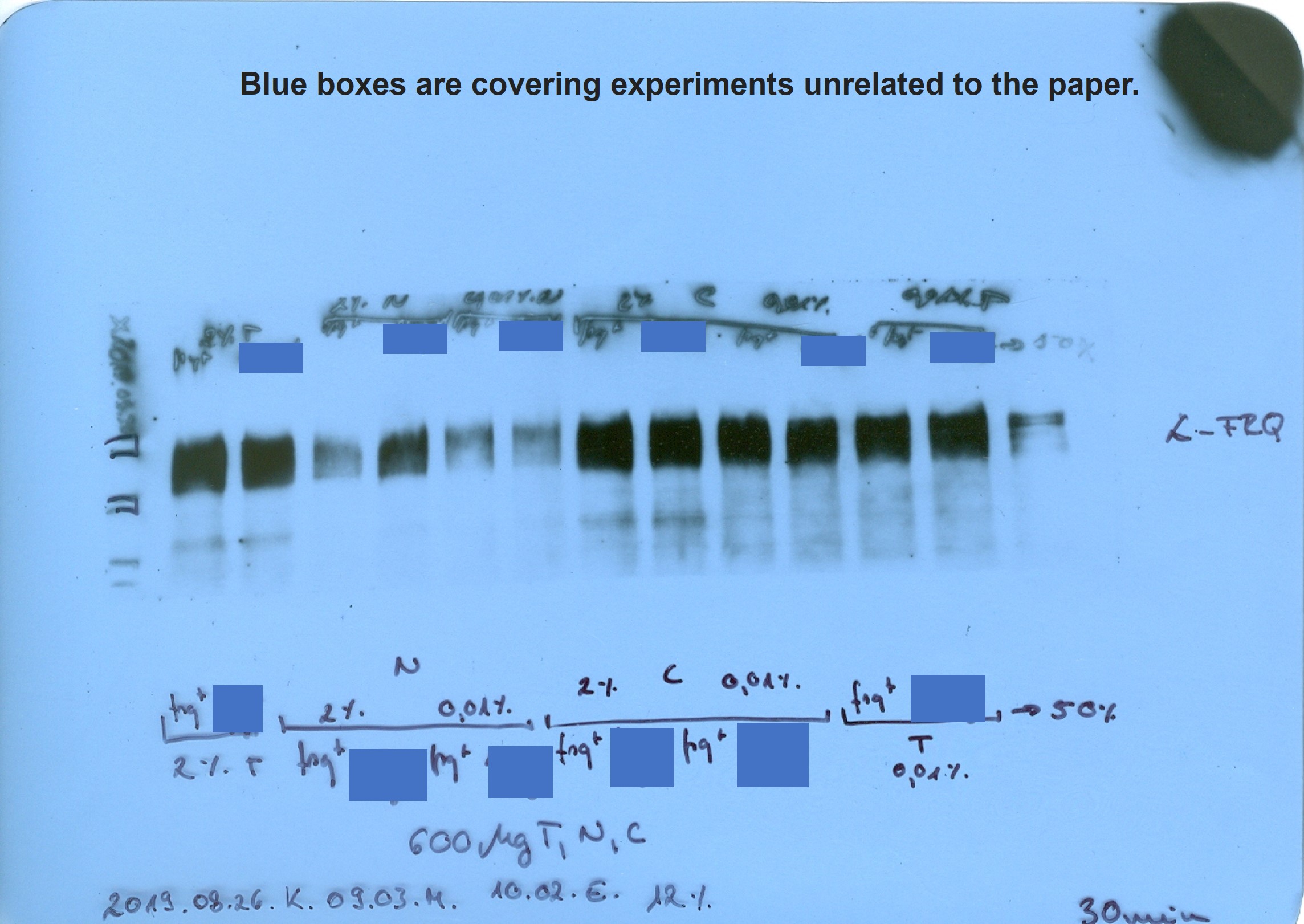

Supplement: Figure 2—source data 1. — (a) Western blots were used to detect expression of FRQ in the indicated samples for Figure 2B (s: short exposure). (b) Figure with the area highlighted was used to develop the Figure 2B for FRQ (s: short exposure). (c) Western blots were used to detect expression of FRQ in the indicated samples for Figure 2B (l: long exposure). (d) Figure with the area highlighted was used to develop the Figure 2B for FRQ (l: long exposure). (e) Western blots were used to detect expression of WC-1 in the indicated samples for Figure 2B (s: short exposure). (f) Figure with the area highlighted was used to develop the Figure 2B for WC-1 (s: short exposure). (g) Western blots were used to detect expression of WC-1 in the indicated samples for Figure 2B (l: long exposure). (h) Figure with the area highlighted was used to develop the Figure 2B for WC-1 (l: long exposure). (i) Western blots were used to detect expression of WC-2 in the indicated samples for Figure 2B (s: short exposure). (j) Figure with the area highlighted was used to develop the Figure 2B for WC-2 (s: short exposure). (k) Western blots were used to detect expression of WC-2 in the indicated samples for Figure 2B (l: long exposure). (l) Figure with the area highlighted was used to develop the Figure 2B for WC-2 (l: long exposure). (m) Ponceau S staining was used to detect loading control of the indicated samples for Figure 2B. (n) Figure with the area highlighted was used as LC for Figure 2B. [file elife-79765-fig2-data1.zip › 79765Figure2B/Figure_2-source_data_1c.jpg]

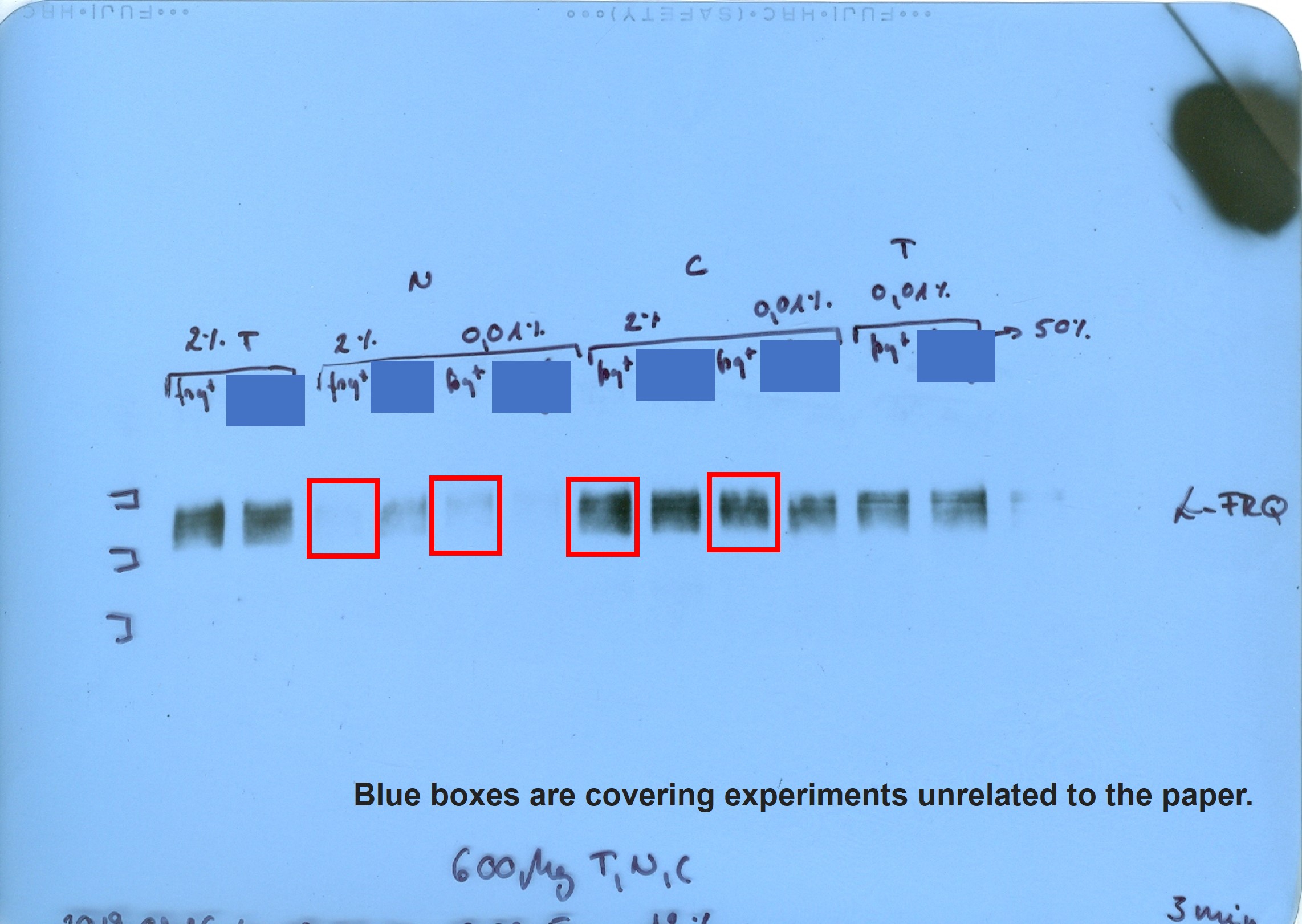

Supplement: Figure 2—source data 1. — (a) Western blots were used to detect expression of FRQ in the indicated samples for Figure 2B (s: short exposure). (b) Figure with the area highlighted was used to develop the Figure 2B for FRQ (s: short exposure). (c) Western blots were used to detect expression of FRQ in the indicated samples for Figure 2B (l: long exposure). (d) Figure with the area highlighted was used to develop the Figure 2B for FRQ (l: long exposure). (e) Western blots were used to detect expression of WC-1 in the indicated samples for Figure 2B (s: short exposure). (f) Figure with the area highlighted was used to develop the Figure 2B for WC-1 (s: short exposure). (g) Western blots were used to detect expression of WC-1 in the indicated samples for Figure 2B (l: long exposure). (h) Figure with the area highlighted was used to develop the Figure 2B for WC-1 (l: long exposure). (i) Western blots were used to detect expression of WC-2 in the indicated samples for Figure 2B (s: short exposure). (j) Figure with the area highlighted was used to develop the Figure 2B for WC-2 (s: short exposure). (k) Western blots were used to detect expression of WC-2 in the indicated samples for Figure 2B (l: long exposure). (l) Figure with the area highlighted was used to develop the Figure 2B for WC-2 (l: long exposure). (m) Ponceau S staining was used to detect loading control of the indicated samples for Figure 2B. (n) Figure with the area highlighted was used as LC for Figure 2B. [file elife-79765-fig2-data1.zip › 79765Figure2B/Figure_2-source_data_1b.jpg]

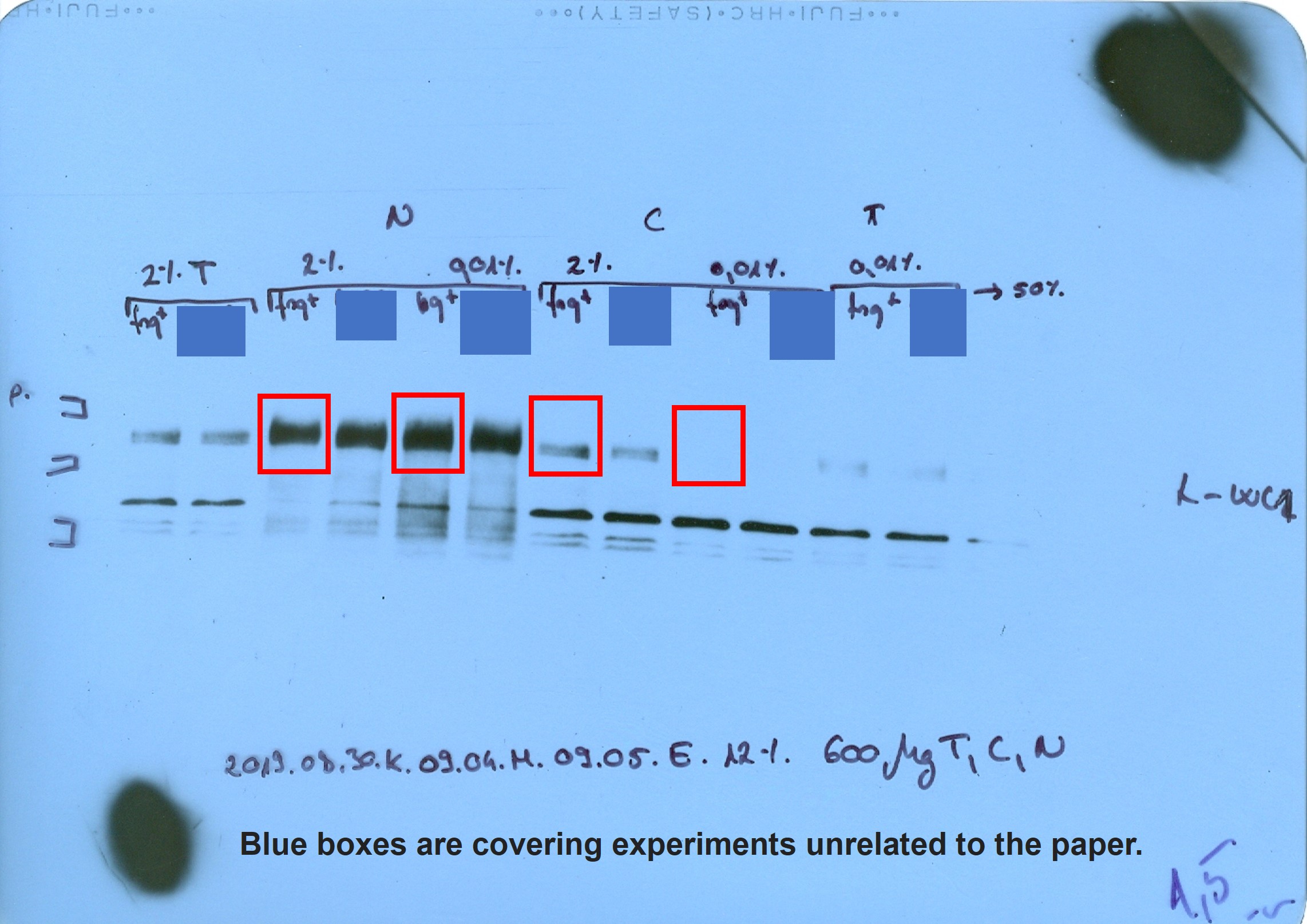

Supplement: Figure 2—source data 1. — (a) Western blots were used to detect expression of FRQ in the indicated samples for Figure 2B (s: short exposure). (b) Figure with the area highlighted was used to develop the Figure 2B for FRQ (s: short exposure). (c) Western blots were used to detect expression of FRQ in the indicated samples for Figure 2B (l: long exposure). (d) Figure with the area highlighted was used to develop the Figure 2B for FRQ (l: long exposure). (e) Western blots were used to detect expression of WC-1 in the indicated samples for Figure 2B (s: short exposure). (f) Figure with the area highlighted was used to develop the Figure 2B for WC-1 (s: short exposure). (g) Western blots were used to detect expression of WC-1 in the indicated samples for Figure 2B (l: long exposure). (h) Figure with the area highlighted was used to develop the Figure 2B for WC-1 (l: long exposure). (i) Western blots were used to detect expression of WC-2 in the indicated samples for Figure 2B (s: short exposure). (j) Figure with the area highlighted was used to develop the Figure 2B for WC-2 (s: short exposure). (k) Western blots were used to detect expression of WC-2 in the indicated samples for Figure 2B (l: long exposure). (l) Figure with the area highlighted was used to develop the Figure 2B for WC-2 (l: long exposure). (m) Ponceau S staining was used to detect loading control of the indicated samples for Figure 2B. (n) Figure with the area highlighted was used as LC for Figure 2B. [file elife-79765-fig2-data1.zip › 79765Figure2B/Figure_2-source_data_1f.jpg]

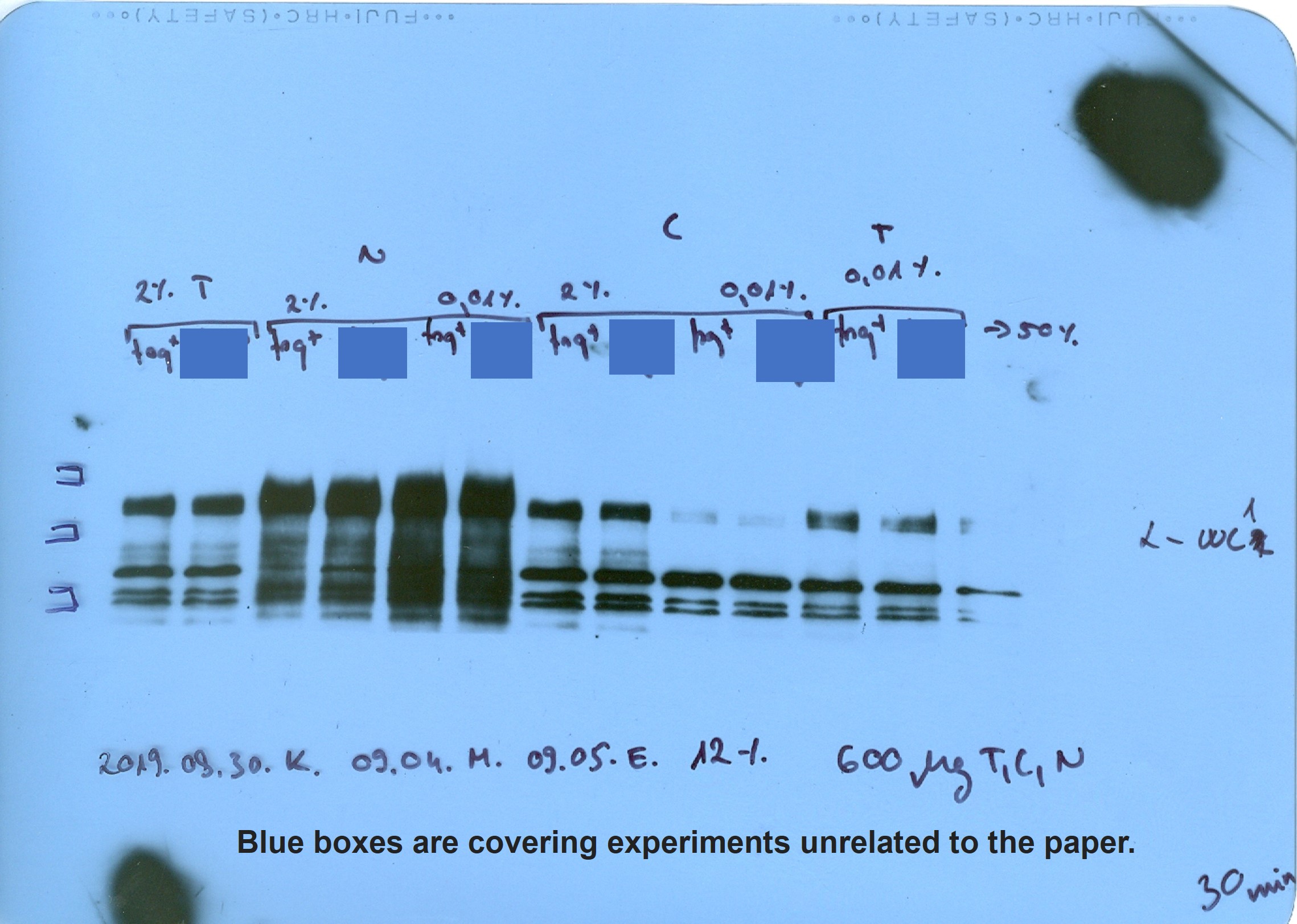

Supplement: Figure 2—source data 1. — (a) Western blots were used to detect expression of FRQ in the indicated samples for Figure 2B (s: short exposure). (b) Figure with the area highlighted was used to develop the Figure 2B for FRQ (s: short exposure). (c) Western blots were used to detect expression of FRQ in the indicated samples for Figure 2B (l: long exposure). (d) Figure with the area highlighted was used to develop the Figure 2B for FRQ (l: long exposure). (e) Western blots were used to detect expression of WC-1 in the indicated samples for Figure 2B (s: short exposure). (f) Figure with the area highlighted was used to develop the Figure 2B for WC-1 (s: short exposure). (g) Western blots were used to detect expression of WC-1 in the indicated samples for Figure 2B (l: long exposure). (h) Figure with the area highlighted was used to develop the Figure 2B for WC-1 (l: long exposure). (i) Western blots were used to detect expression of WC-2 in the indicated samples for Figure 2B (s: short exposure). (j) Figure with the area highlighted was used to develop the Figure 2B for WC-2 (s: short exposure). (k) Western blots were used to detect expression of WC-2 in the indicated samples for Figure 2B (l: long exposure). (l) Figure with the area highlighted was used to develop the Figure 2B for WC-2 (l: long exposure). (m) Ponceau S staining was used to detect loading control of the indicated samples for Figure 2B. (n) Figure with the area highlighted was used as LC for Figure 2B. [file elife-79765-fig2-data1.zip › 79765Figure2B/Figure_2-source_data_1g.jpg]

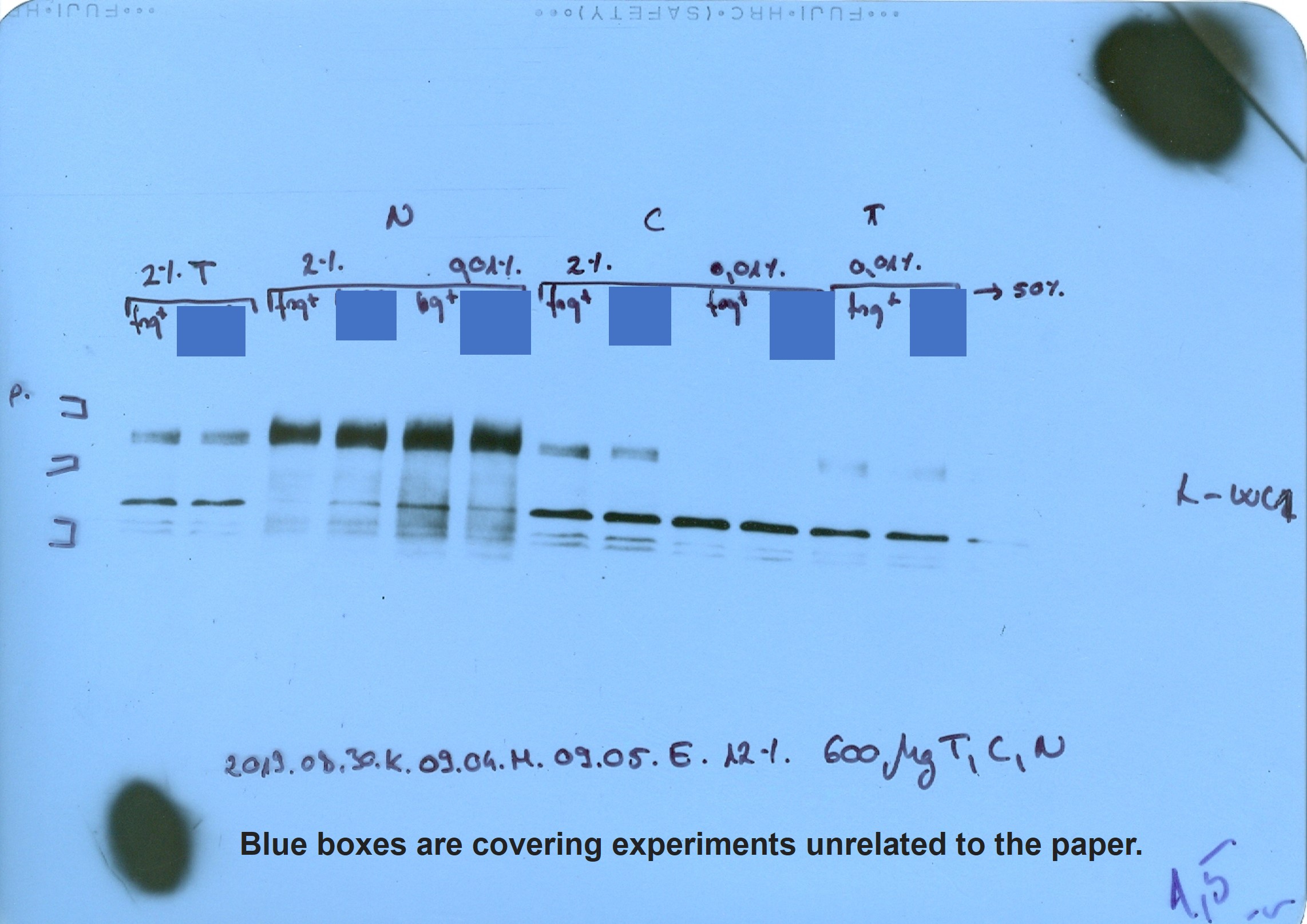

Supplement: Figure 2—source data 1. — (a) Western blots were used to detect expression of FRQ in the indicated samples for Figure 2B (s: short exposure). (b) Figure with the area highlighted was used to develop the Figure 2B for FRQ (s: short exposure). (c) Western blots were used to detect expression of FRQ in the indicated samples for Figure 2B (l: long exposure). (d) Figure with the area highlighted was used to develop the Figure 2B for FRQ (l: long exposure). (e) Western blots were used to detect expression of WC-1 in the indicated samples for Figure 2B (s: short exposure). (f) Figure with the area highlighted was used to develop the Figure 2B for WC-1 (s: short exposure). (g) Western blots were used to detect expression of WC-1 in the indicated samples for Figure 2B (l: long exposure). (h) Figure with the area highlighted was used to develop the Figure 2B for WC-1 (l: long exposure). (i) Western blots were used to detect expression of WC-2 in the indicated samples for Figure 2B (s: short exposure). (j) Figure with the area highlighted was used to develop the Figure 2B for WC-2 (s: short exposure). (k) Western blots were used to detect expression of WC-2 in the indicated samples for Figure 2B (l: long exposure). (l) Figure with the area highlighted was used to develop the Figure 2B for WC-2 (l: long exposure). (m) Ponceau S staining was used to detect loading control of the indicated samples for Figure 2B. (n) Figure with the area highlighted was used as LC for Figure 2B. [file elife-79765-fig2-data1.zip › 79765Figure2B/Figure_2-source_data_1e.jpg]

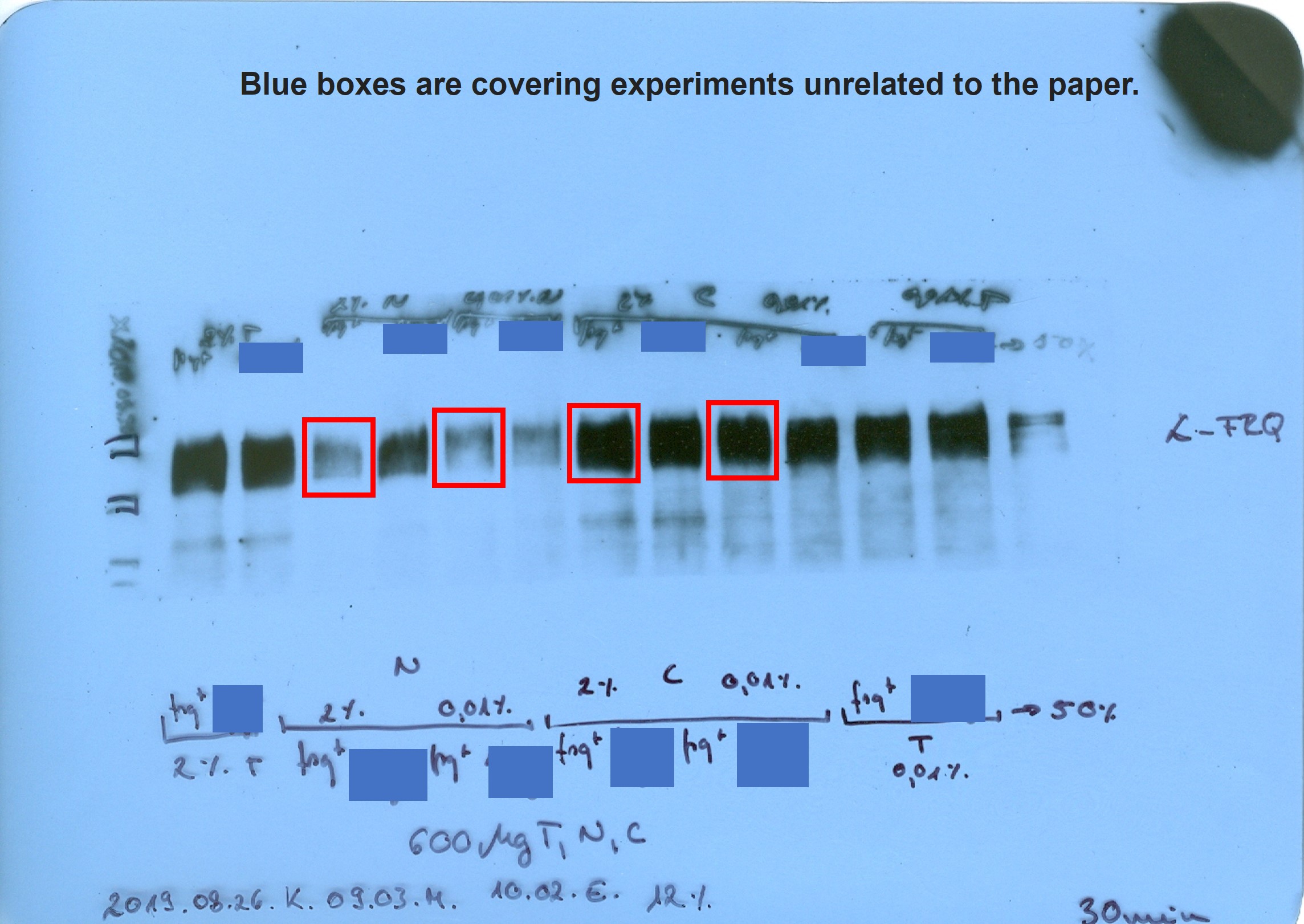

Supplement: Figure 2—source data 1. — (a) Western blots were used to detect expression of FRQ in the indicated samples for Figure 2B (s: short exposure). (b) Figure with the area highlighted was used to develop the Figure 2B for FRQ (s: short exposure). (c) Western blots were used to detect expression of FRQ in the indicated samples for Figure 2B (l: long exposure). (d) Figure with the area highlighted was used to develop the Figure 2B for FRQ (l: long exposure). (e) Western blots were used to detect expression of WC-1 in the indicated samples for Figure 2B (s: short exposure). (f) Figure with the area highlighted was used to develop the Figure 2B for WC-1 (s: short exposure). (g) Western blots were used to detect expression of WC-1 in the indicated samples for Figure 2B (l: long exposure). (h) Figure with the area highlighted was used to develop the Figure 2B for WC-1 (l: long exposure). (i) Western blots were used to detect expression of WC-2 in the indicated samples for Figure 2B (s: short exposure). (j) Figure with the area highlighted was used to develop the Figure 2B for WC-2 (s: short exposure). (k) Western blots were used to detect expression of WC-2 in the indicated samples for Figure 2B (l: long exposure). (l) Figure with the area highlighted was used to develop the Figure 2B for WC-2 (l: long exposure). (m) Ponceau S staining was used to detect loading control of the indicated samples for Figure 2B. (n) Figure with the area highlighted was used as LC for Figure 2B. [file elife-79765-fig2-data1.zip › 79765Figure2B/Figure_2-source_data_1d.jpg]

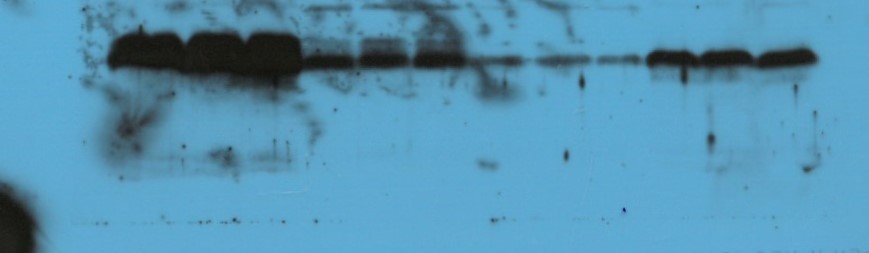

Supplement: Figure 3—source data 1. — (a) Western blots were used to detect expression of WC-1 in the indicated samples for Figure 3B (s: short exposure). (b) Figure with the area highlighted was used to develop the Figure 3B for WC-1 (s: short exposure). (c) Western blots were used to detect expression of WC-1 in the indicated samples for Figure 3B (l: long exposure). (d) Figure with the area highlighted was used to develop the Figure 3B for WC-1 (l: long exposure). (e) Western blots were used to detect expression of WC-2 in the indicated samples for Figure 3B (s: short exposure). (f) Figure with the area highlighted was used to develop the Figure 3B for WC-2 (s: short exposure). (g) Western blots were used to detect expression of WC-2 in the indicated samples for Figure 3B (l: long exposure). (h) Figure with the area highlighted was used to develop the Figure 3B for WC-2 (l: long exposure). (i) Ponceau S staining was used to detect loading control of the indicated samples for Figure 3B. (j) Figure with the area highlighted was used to as LC for WC-1 in Figure 3B. (k) Figure with the area highlighted was used as LC for WC-2 in Figure 3B . [file elife-79765-fig3-data1.zip › 79765Figure3B/Figure_3-source_data_1g.jpg]

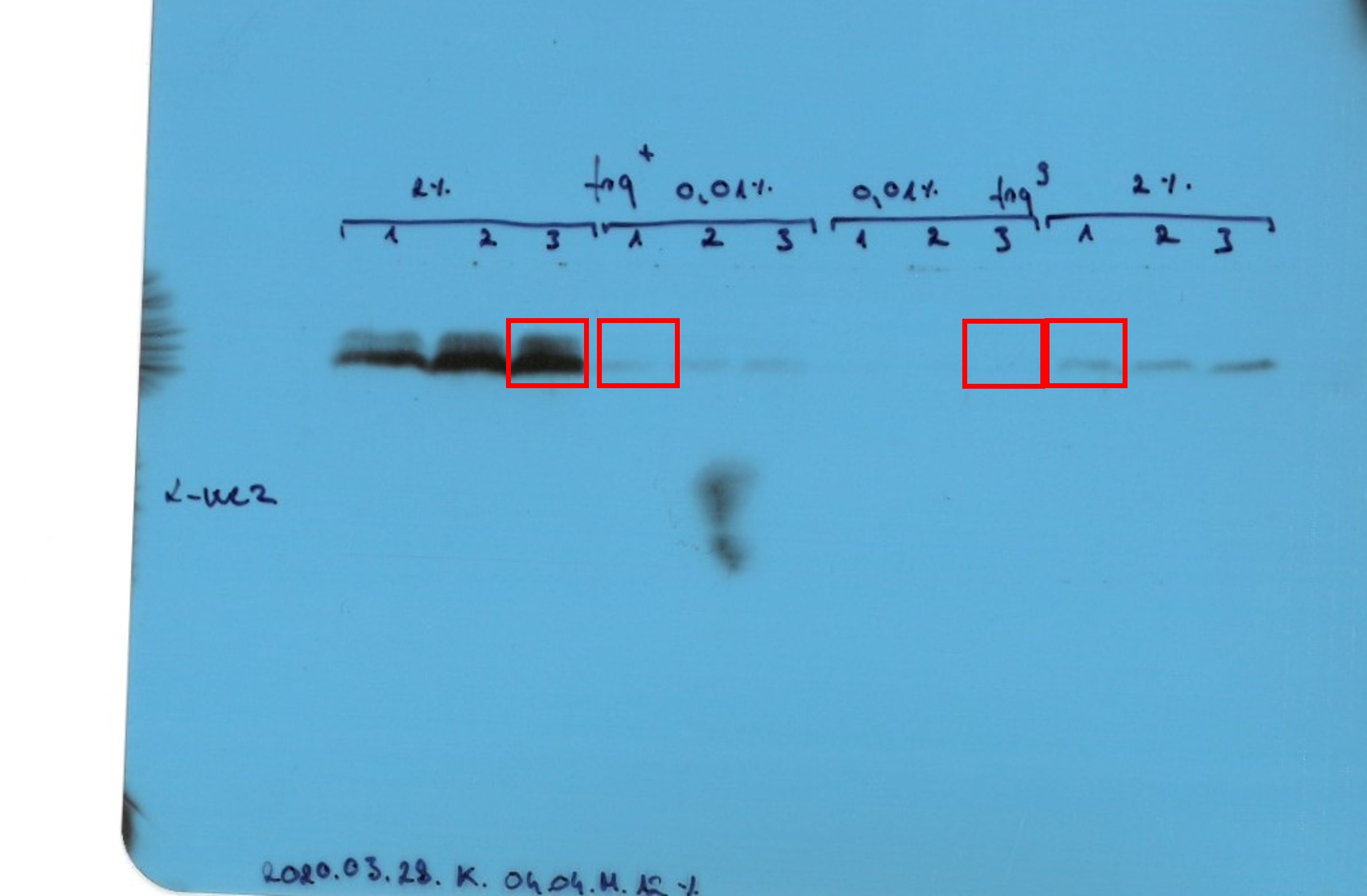

Supplement: Figure 3—source data 1. — (a) Western blots were used to detect expression of WC-1 in the indicated samples for Figure 3B (s: short exposure). (b) Figure with the area highlighted was used to develop the Figure 3B for WC-1 (s: short exposure). (c) Western blots were used to detect expression of WC-1 in the indicated samples for Figure 3B (l: long exposure). (d) Figure with the area highlighted was used to develop the Figure 3B for WC-1 (l: long exposure). (e) Western blots were used to detect expression of WC-2 in the indicated samples for Figure 3B (s: short exposure). (f) Figure with the area highlighted was used to develop the Figure 3B for WC-2 (s: short exposure). (g) Western blots were used to detect expression of WC-2 in the indicated samples for Figure 3B (l: long exposure). (h) Figure with the area highlighted was used to develop the Figure 3B for WC-2 (l: long exposure). (i) Ponceau S staining was used to detect loading control of the indicated samples for Figure 3B. (j) Figure with the area highlighted was used to as LC for WC-1 in Figure 3B. (k) Figure with the area highlighted was used as LC for WC-2 in Figure 3B . [file elife-79765-fig3-data1.zip › 79765Figure3B/Figure_3-source_data_1f.jpg]

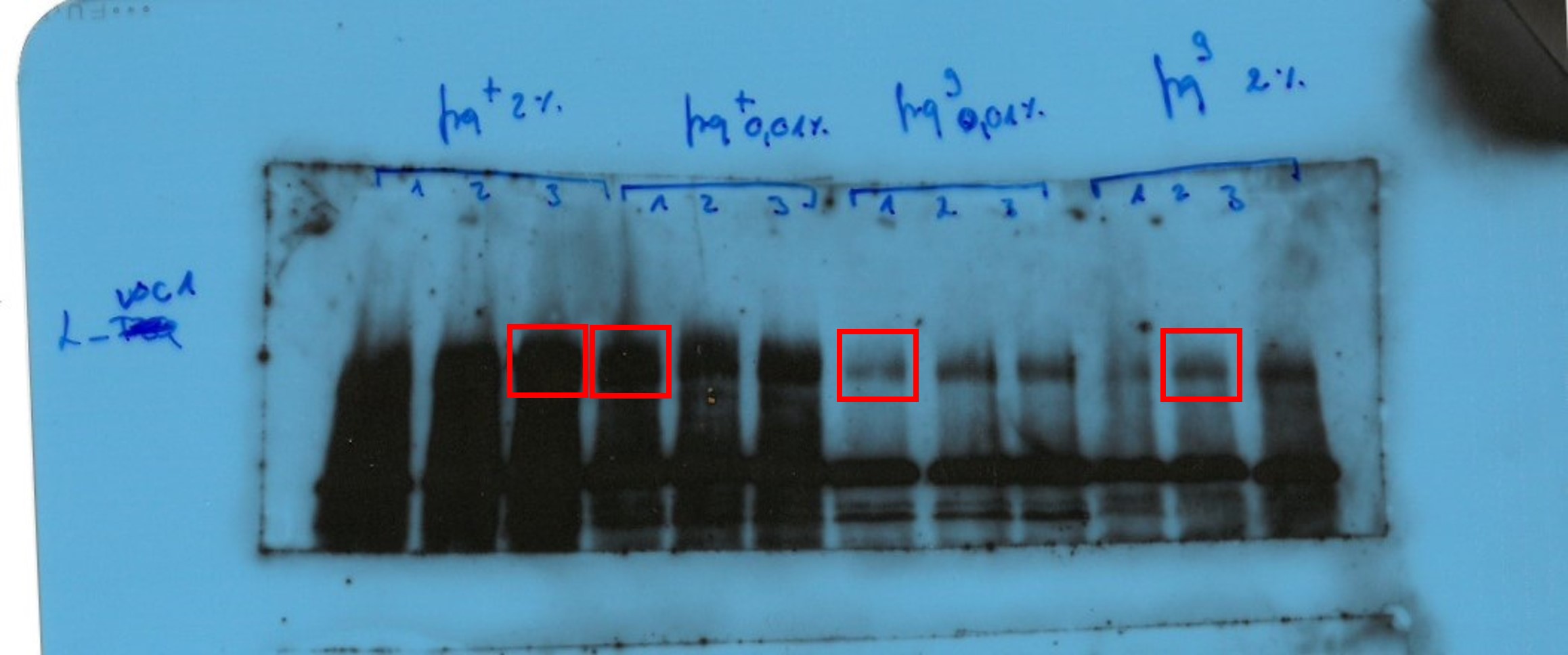

Supplement: Figure 3—source data 1. — (a) Western blots were used to detect expression of WC-1 in the indicated samples for Figure 3B (s: short exposure). (b) Figure with the area highlighted was used to develop the Figure 3B for WC-1 (s: short exposure). (c) Western blots were used to detect expression of WC-1 in the indicated samples for Figure 3B (l: long exposure). (d) Figure with the area highlighted was used to develop the Figure 3B for WC-1 (l: long exposure). (e) Western blots were used to detect expression of WC-2 in the indicated samples for Figure 3B (s: short exposure). (f) Figure with the area highlighted was used to develop the Figure 3B for WC-2 (s: short exposure). (g) Western blots were used to detect expression of WC-2 in the indicated samples for Figure 3B (l: long exposure). (h) Figure with the area highlighted was used to develop the Figure 3B for WC-2 (l: long exposure). (i) Ponceau S staining was used to detect loading control of the indicated samples for Figure 3B. (j) Figure with the area highlighted was used to as LC for WC-1 in Figure 3B. (k) Figure with the area highlighted was used as LC for WC-2 in Figure 3B . [file elife-79765-fig3-data1.zip › 79765Figure3B/Figure_3-source_data_1d.jpg]

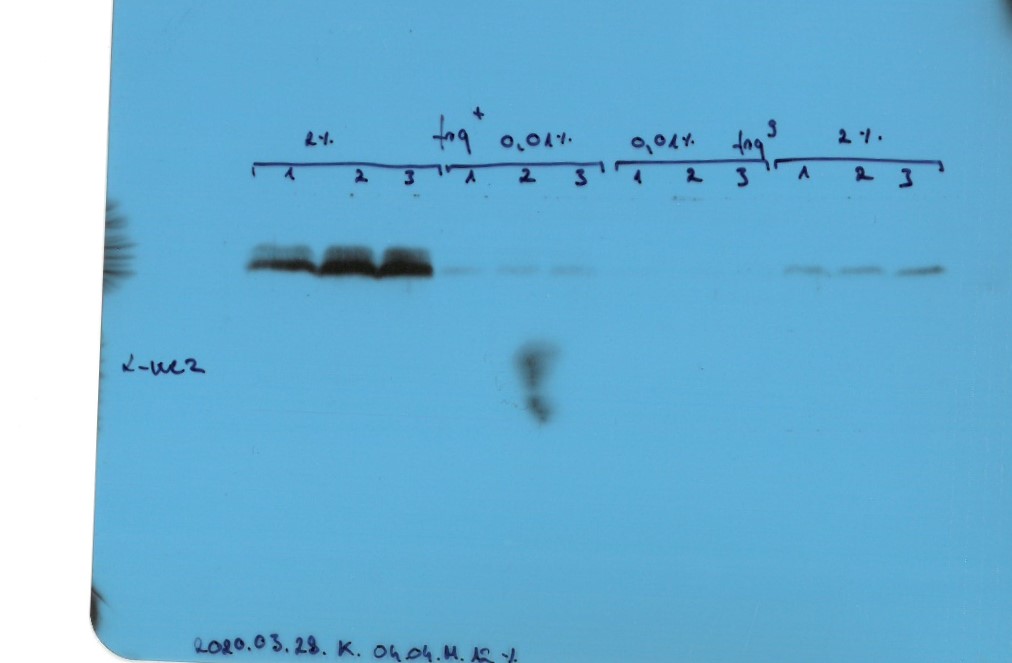

Supplement: Figure 3—source data 1. — (a) Western blots were used to detect expression of WC-1 in the indicated samples for Figure 3B (s: short exposure). (b) Figure with the area highlighted was used to develop the Figure 3B for WC-1 (s: short exposure). (c) Western blots were used to detect expression of WC-1 in the indicated samples for Figure 3B (l: long exposure). (d) Figure with the area highlighted was used to develop the Figure 3B for WC-1 (l: long exposure). (e) Western blots were used to detect expression of WC-2 in the indicated samples for Figure 3B (s: short exposure). (f) Figure with the area highlighted was used to develop the Figure 3B for WC-2 (s: short exposure). (g) Western blots were used to detect expression of WC-2 in the indicated samples for Figure 3B (l: long exposure). (h) Figure with the area highlighted was used to develop the Figure 3B for WC-2 (l: long exposure). (i) Ponceau S staining was used to detect loading control of the indicated samples for Figure 3B. (j) Figure with the area highlighted was used to as LC for WC-1 in Figure 3B. (k) Figure with the area highlighted was used as LC for WC-2 in Figure 3B . [file elife-79765-fig3-data1.zip › 79765Figure3B/Figure_3-source_data_1e.jpg]

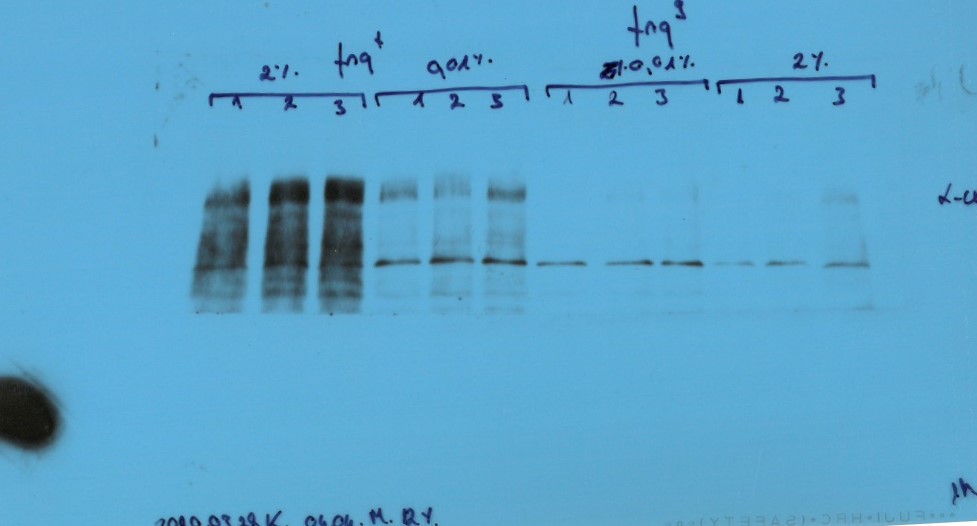

Supplement: Figure 3—source data 1. — (a) Western blots were used to detect expression of WC-1 in the indicated samples for Figure 3B (s: short exposure). (b) Figure with the area highlighted was used to develop the Figure 3B for WC-1 (s: short exposure). (c) Western blots were used to detect expression of WC-1 in the indicated samples for Figure 3B (l: long exposure). (d) Figure with the area highlighted was used to develop the Figure 3B for WC-1 (l: long exposure). (e) Western blots were used to detect expression of WC-2 in the indicated samples for Figure 3B (s: short exposure). (f) Figure with the area highlighted was used to develop the Figure 3B for WC-2 (s: short exposure). (g) Western blots were used to detect expression of WC-2 in the indicated samples for Figure 3B (l: long exposure). (h) Figure with the area highlighted was used to develop the Figure 3B for WC-2 (l: long exposure). (i) Ponceau S staining was used to detect loading control of the indicated samples for Figure 3B. (j) Figure with the area highlighted was used to as LC for WC-1 in Figure 3B. (k) Figure with the area highlighted was used as LC for WC-2 in Figure 3B . [file elife-79765-fig3-data1.zip › 79765Figure3B/Figure_3-source_data_1a.jpg]

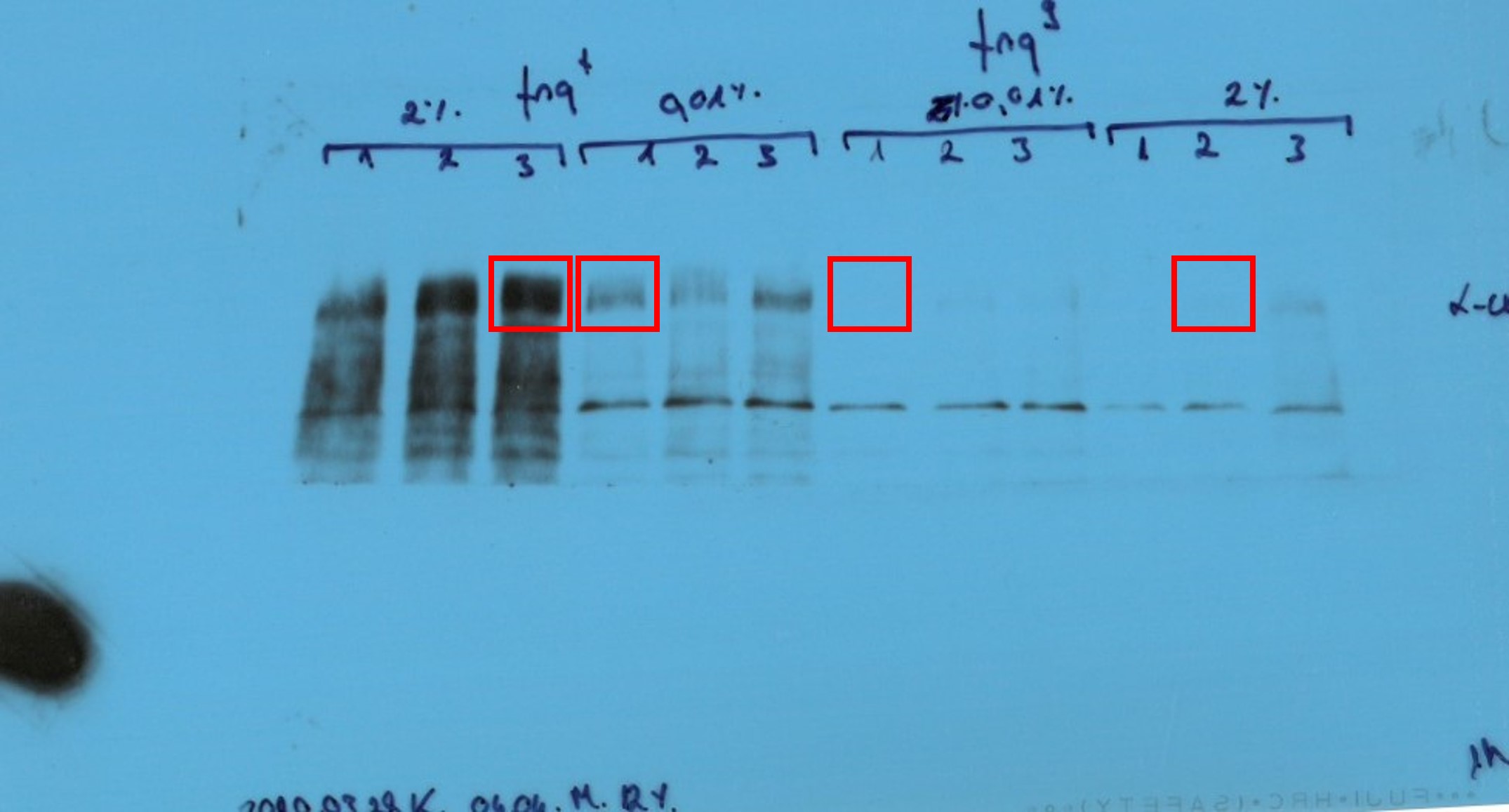

Supplement: Figure 3—source data 1. — (a) Western blots were used to detect expression of WC-1 in the indicated samples for Figure 3B (s: short exposure). (b) Figure with the area highlighted was used to develop the Figure 3B for WC-1 (s: short exposure). (c) Western blots were used to detect expression of WC-1 in the indicated samples for Figure 3B (l: long exposure). (d) Figure with the area highlighted was used to develop the Figure 3B for WC-1 (l: long exposure). (e) Western blots were used to detect expression of WC-2 in the indicated samples for Figure 3B (s: short exposure). (f) Figure with the area highlighted was used to develop the Figure 3B for WC-2 (s: short exposure). (g) Western blots were used to detect expression of WC-2 in the indicated samples for Figure 3B (l: long exposure). (h) Figure with the area highlighted was used to develop the Figure 3B for WC-2 (l: long exposure). (i) Ponceau S staining was used to detect loading control of the indicated samples for Figure 3B. (j) Figure with the area highlighted was used to as LC for WC-1 in Figure 3B. (k) Figure with the area highlighted was used as LC for WC-2 in Figure 3B . [file elife-79765-fig3-data1.zip › 79765Figure3B/Figure_3-source_data_1b.jpg]

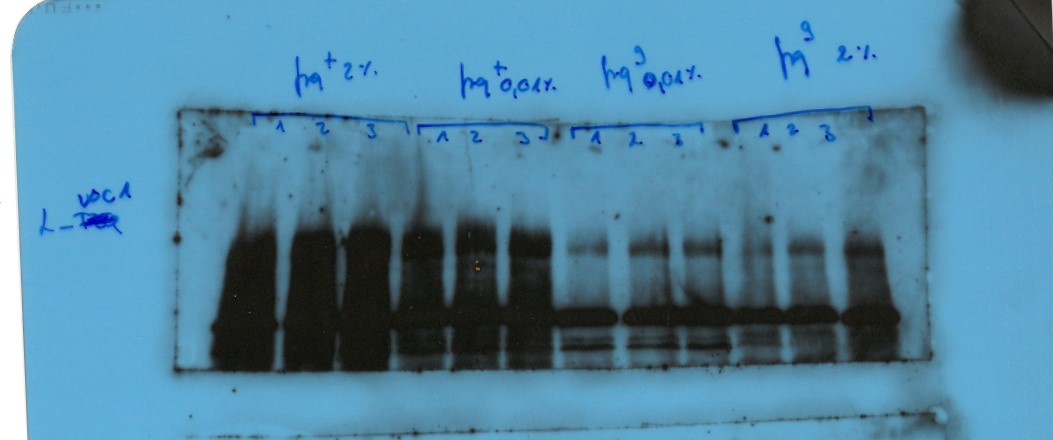

Supplement: Figure 3—source data 1. — (a) Western blots were used to detect expression of WC-1 in the indicated samples for Figure 3B (s: short exposure). (b) Figure with the area highlighted was used to develop the Figure 3B for WC-1 (s: short exposure). (c) Western blots were used to detect expression of WC-1 in the indicated samples for Figure 3B (l: long exposure). (d) Figure with the area highlighted was used to develop the Figure 3B for WC-1 (l: long exposure). (e) Western blots were used to detect expression of WC-2 in the indicated samples for Figure 3B (s: short exposure). (f) Figure with the area highlighted was used to develop the Figure 3B for WC-2 (s: short exposure). (g) Western blots were used to detect expression of WC-2 in the indicated samples for Figure 3B (l: long exposure). (h) Figure with the area highlighted was used to develop the Figure 3B for WC-2 (l: long exposure). (i) Ponceau S staining was used to detect loading control of the indicated samples for Figure 3B. (j) Figure with the area highlighted was used to as LC for WC-1 in Figure 3B. (k) Figure with the area highlighted was used as LC for WC-2 in Figure 3B . [file elife-79765-fig3-data1.zip › 79765Figure3B/Figure_3-source_data_1c.jpg]

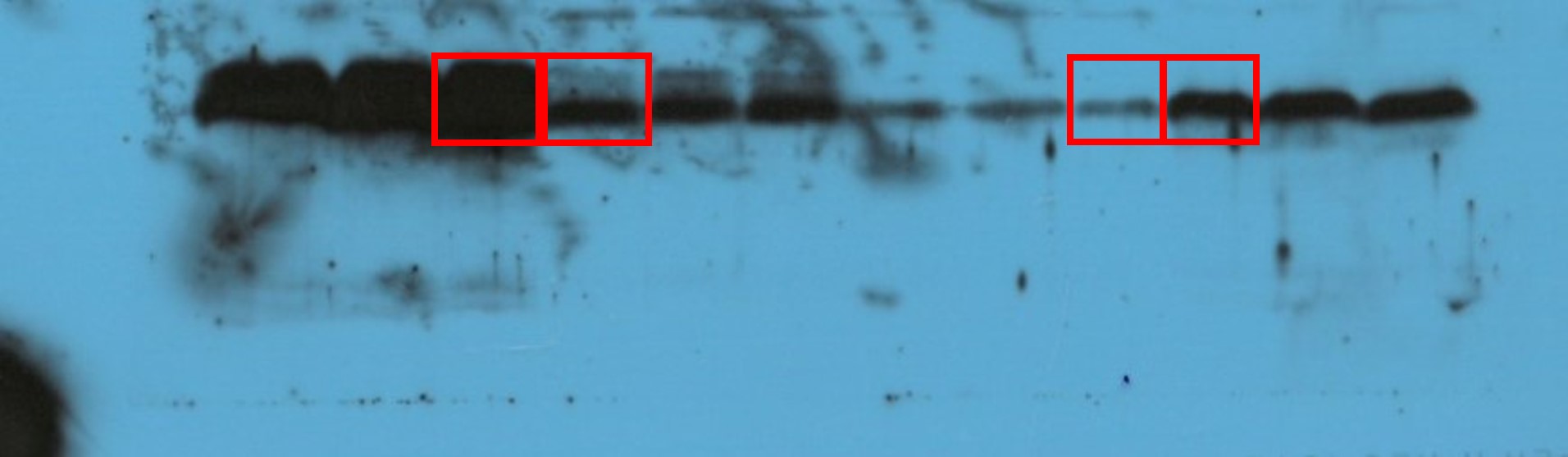

Supplement: Figure 3—source data 1. — (a) Western blots were used to detect expression of WC-1 in the indicated samples for Figure 3B (s: short exposure). (b) Figure with the area highlighted was used to develop the Figure 3B for WC-1 (s: short exposure). (c) Western blots were used to detect expression of WC-1 in the indicated samples for Figure 3B (l: long exposure). (d) Figure with the area highlighted was used to develop the Figure 3B for WC-1 (l: long exposure). (e) Western blots were used to detect expression of WC-2 in the indicated samples for Figure 3B (s: short exposure). (f) Figure with the area highlighted was used to develop the Figure 3B for WC-2 (s: short exposure). (g) Western blots were used to detect expression of WC-2 in the indicated samples for Figure 3B (l: long exposure). (h) Figure with the area highlighted was used to develop the Figure 3B for WC-2 (l: long exposure). (i) Ponceau S staining was used to detect loading control of the indicated samples for Figure 3B. (j) Figure with the area highlighted was used to as LC for WC-1 in Figure 3B. (k) Figure with the area highlighted was used as LC for WC-2 in Figure 3B . [file elife-79765-fig3-data1.zip › 79765Figure3B/Figure_3-source_data_1h.jpg]

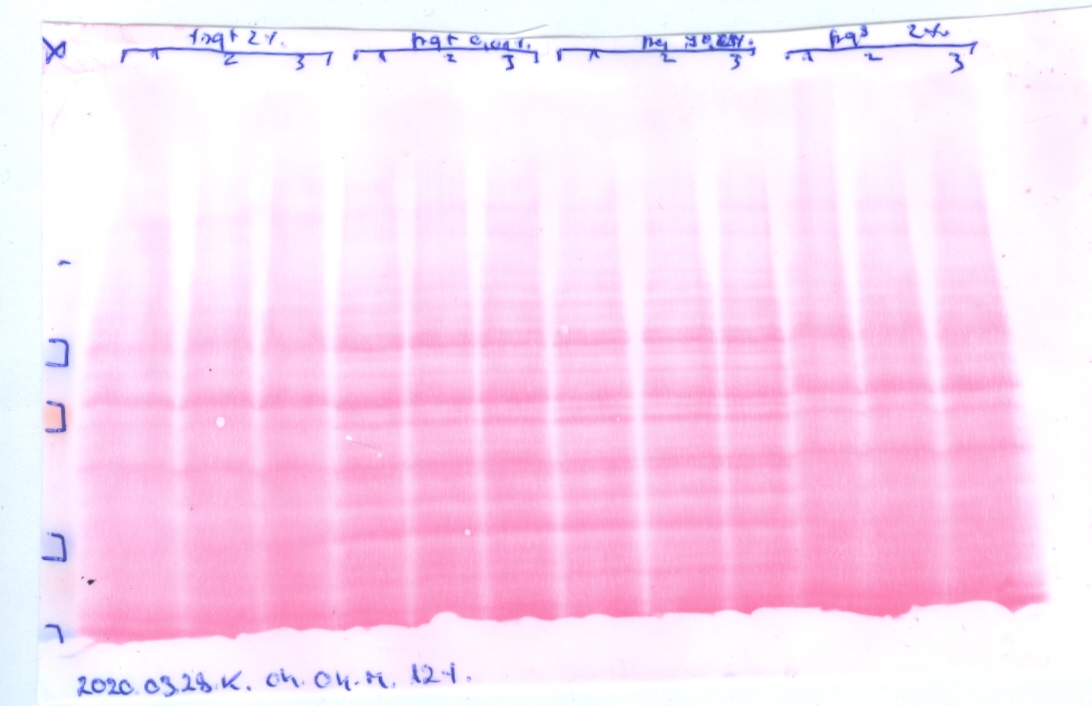

Supplement: Figure 3—source data 1. — (a) Western blots were used to detect expression of WC-1 in the indicated samples for Figure 3B (s: short exposure). (b) Figure with the area highlighted was used to develop the Figure 3B for WC-1 (s: short exposure). (c) Western blots were used to detect expression of WC-1 in the indicated samples for Figure 3B (l: long exposure). (d) Figure with the area highlighted was used to develop the Figure 3B for WC-1 (l: long exposure). (e) Western blots were used to detect expression of WC-2 in the indicated samples for Figure 3B (s: short exposure). (f) Figure with the area highlighted was used to develop the Figure 3B for WC-2 (s: short exposure). (g) Western blots were used to detect expression of WC-2 in the indicated samples for Figure 3B (l: long exposure). (h) Figure with the area highlighted was used to develop the Figure 3B for WC-2 (l: long exposure). (i) Ponceau S staining was used to detect loading control of the indicated samples for Figure 3B. (j) Figure with the area highlighted was used to as LC for WC-1 in Figure 3B. (k) Figure with the area highlighted was used as LC for WC-2 in Figure 3B . [file elife-79765-fig3-data1.zip › 79765Figure3B/Figure_3-source_data_1i.jpg]

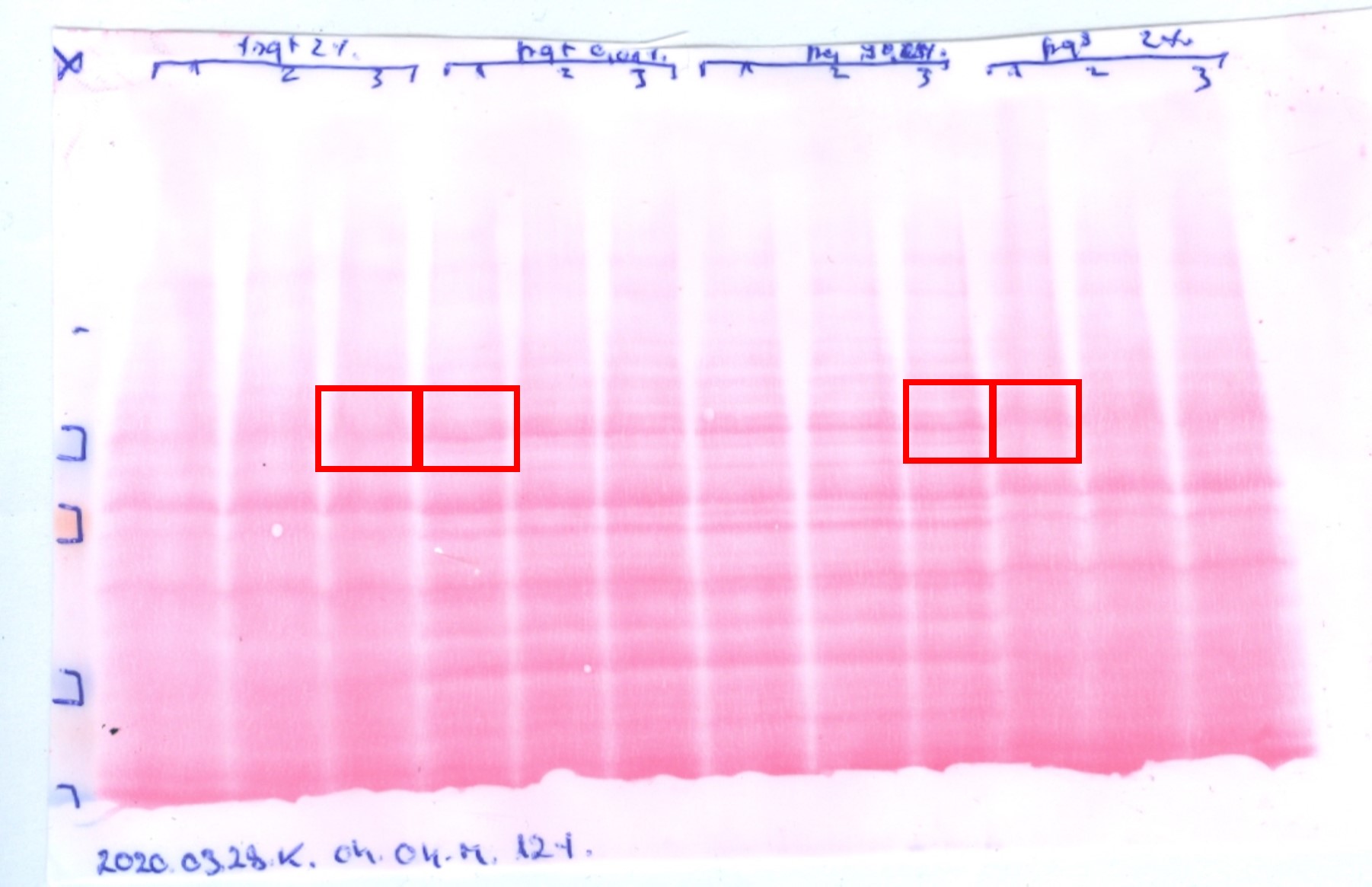

Supplement: Figure 3—source data 1. — (a) Western blots were used to detect expression of WC-1 in the indicated samples for Figure 3B (s: short exposure). (b) Figure with the area highlighted was used to develop the Figure 3B for WC-1 (s: short exposure). (c) Western blots were used to detect expression of WC-1 in the indicated samples for Figure 3B (l: long exposure). (d) Figure with the area highlighted was used to develop the Figure 3B for WC-1 (l: long exposure). (e) Western blots were used to detect expression of WC-2 in the indicated samples for Figure 3B (s: short exposure). (f) Figure with the area highlighted was used to develop the Figure 3B for WC-2 (s: short exposure). (g) Western blots were used to detect expression of WC-2 in the indicated samples for Figure 3B (l: long exposure). (h) Figure with the area highlighted was used to develop the Figure 3B for WC-2 (l: long exposure). (i) Ponceau S staining was used to detect loading control of the indicated samples for Figure 3B. (j) Figure with the area highlighted was used to as LC for WC-1 in Figure 3B. (k) Figure with the area highlighted was used as LC for WC-2 in Figure 3B . [file elife-79765-fig3-data1.zip › 79765Figure3B/Figure_3-source_data_1k.jpg]

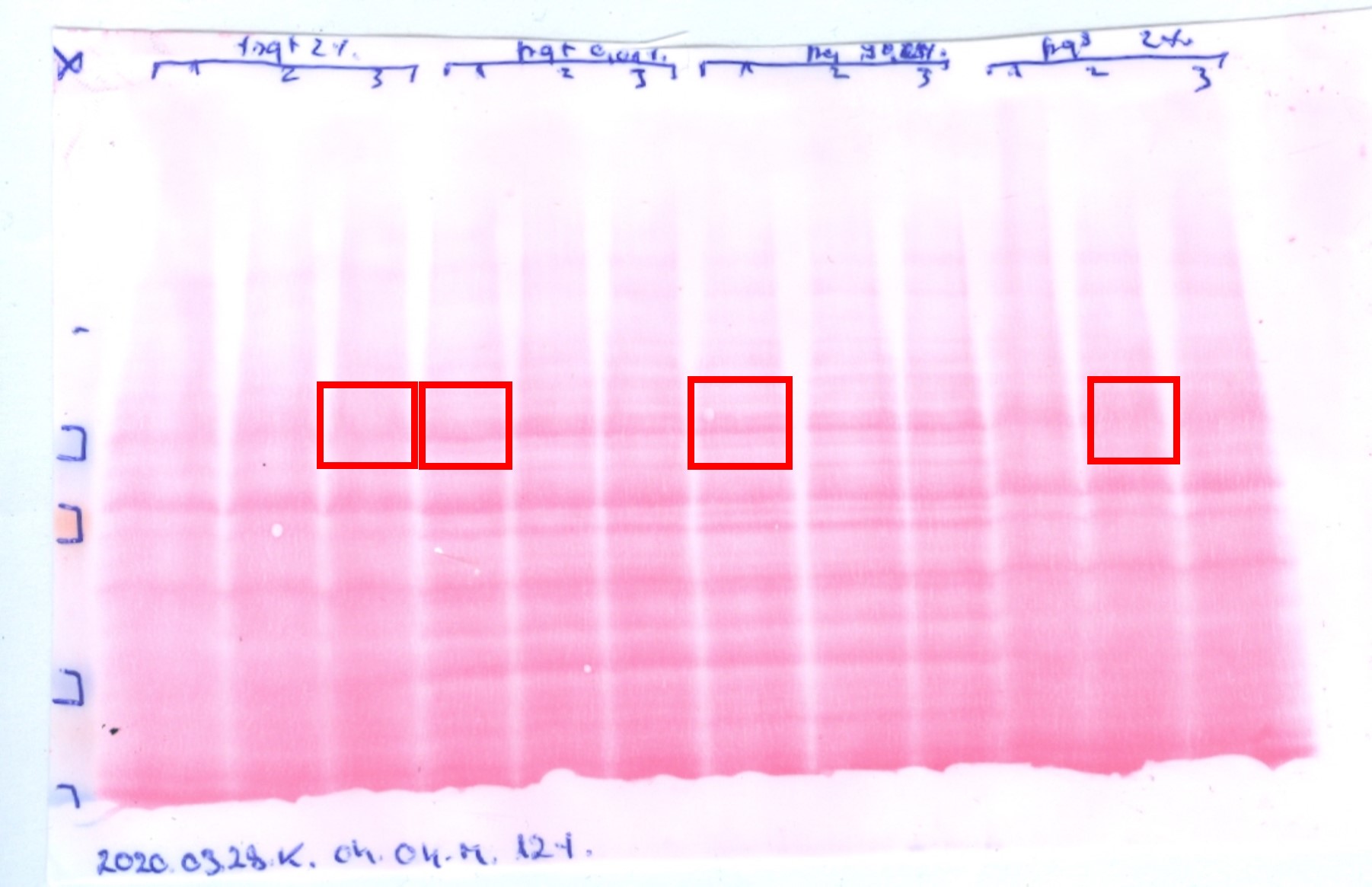

Supplement: Figure 3—source data 1. — (a) Western blots were used to detect expression of WC-1 in the indicated samples for Figure 3B (s: short exposure). (b) Figure with the area highlighted was used to develop the Figure 3B for WC-1 (s: short exposure). (c) Western blots were used to detect expression of WC-1 in the indicated samples for Figure 3B (l: long exposure). (d) Figure with the area highlighted was used to develop the Figure 3B for WC-1 (l: long exposure). (e) Western blots were used to detect expression of WC-2 in the indicated samples for Figure 3B (s: short exposure). (f) Figure with the area highlighted was used to develop the Figure 3B for WC-2 (s: short exposure). (g) Western blots were used to detect expression of WC-2 in the indicated samples for Figure 3B (l: long exposure). (h) Figure with the area highlighted was used to develop the Figure 3B for WC-2 (l: long exposure). (i) Ponceau S staining was used to detect loading control of the indicated samples for Figure 3B. (j) Figure with the area highlighted was used to as LC for WC-1 in Figure 3B. (k) Figure with the area highlighted was used as LC for WC-2 in Figure 3B . [file elife-79765-fig3-data1.zip › 79765Figure3B/Figure_3-source_data_1j.jpg]

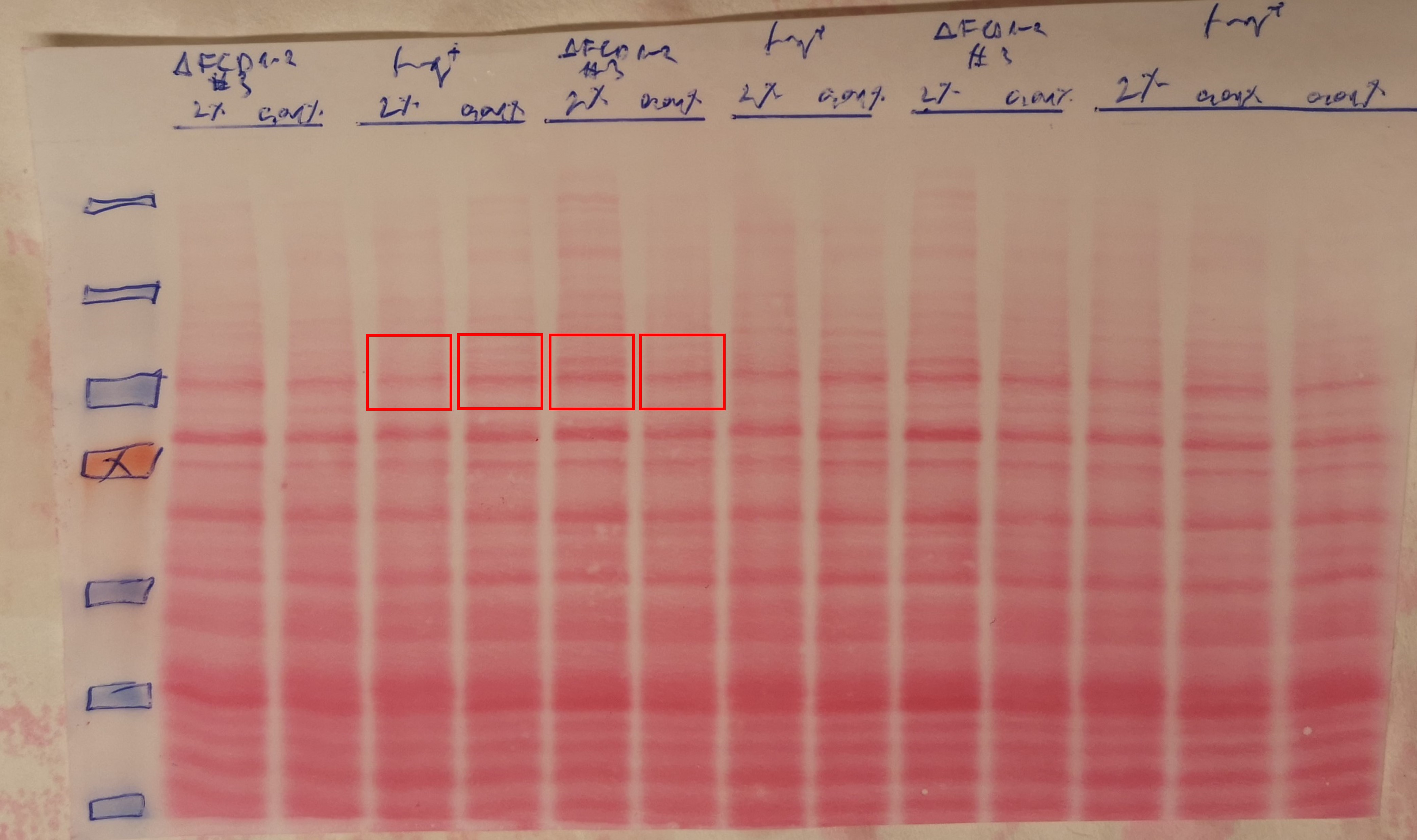

Supplement: Figure 3—source data 2. — (a) Western blots were used to detect expression of FRQ in the indicated samples for Figure 3C. (b) Figure with the area highlighted was used to develop the Figure 3C for FRQ. (c) Western blots were used to detect expression of WC-1 in the indicated samples for Figure 3C. (d) Figure with the area highlighted was used to develop the Figure 3C for WC-1. (e) Western blots were used to detect expression of WC-2 in the indicated samples for Figure 3C. (f) Figure with the area highlighted was used to develop the Figure 3C for WC-2. (g) Ponceau S staining was used to detect loading control of WC-1 for Figure 3C. (h) Figure with the area highlighted was used to develop the Figure 3C for LC for WC-1. (i) Ponceau S staining was used to detect loading control of FRQ and WC-2 for Figure 3C. (j) Figure with the area highlighted was used as LC for FRQ and WC-2 for Figure 3C. [file elife-79765-fig3-data2.zip › 79765Figure3C/Figure_3-source_data_2j.jpg]

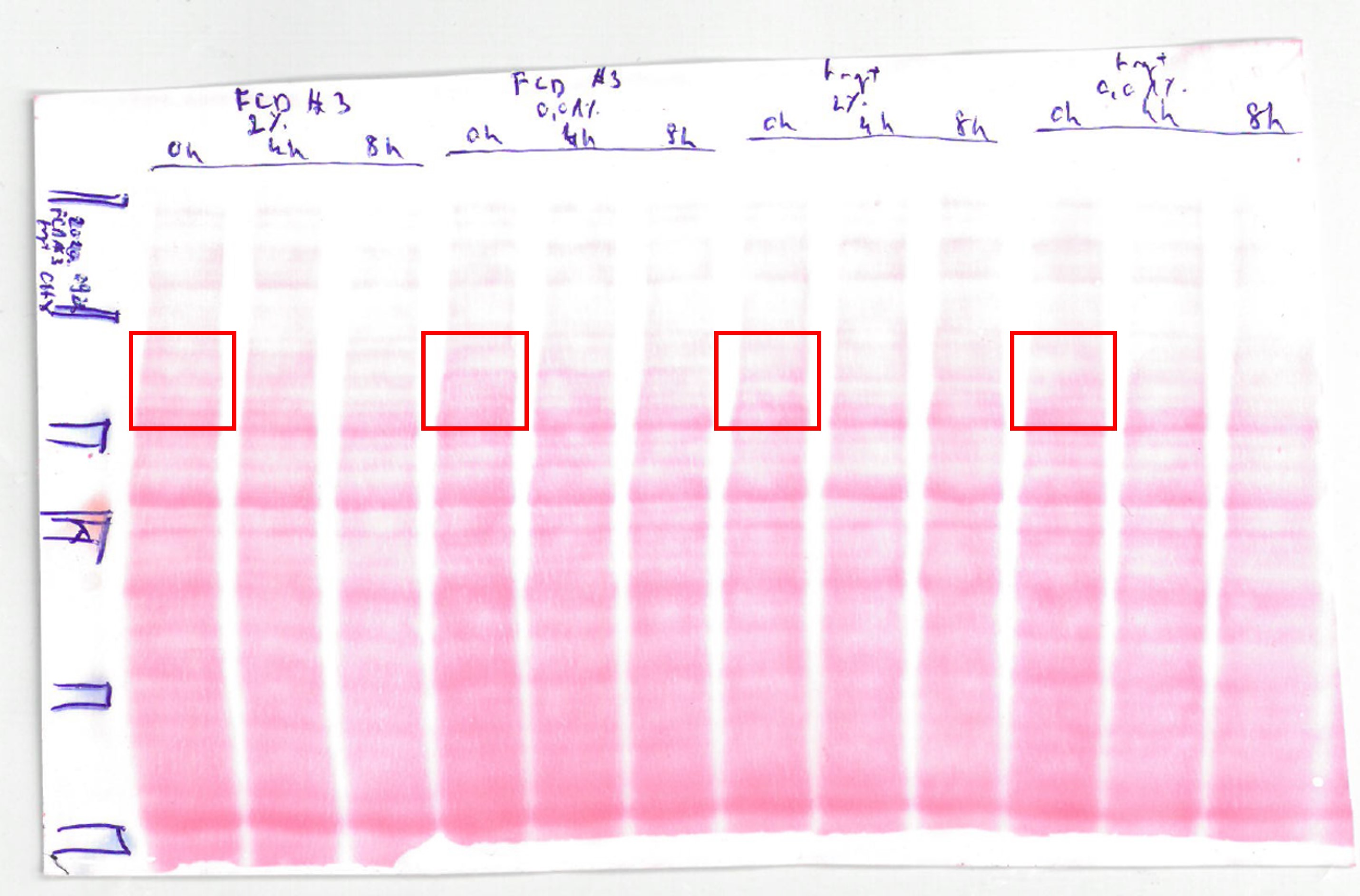

Supplement: Figure 3—source data 2. — (a) Western blots were used to detect expression of FRQ in the indicated samples for Figure 3C. (b) Figure with the area highlighted was used to develop the Figure 3C for FRQ. (c) Western blots were used to detect expression of WC-1 in the indicated samples for Figure 3C. (d) Figure with the area highlighted was used to develop the Figure 3C for WC-1. (e) Western blots were used to detect expression of WC-2 in the indicated samples for Figure 3C. (f) Figure with the area highlighted was used to develop the Figure 3C for WC-2. (g) Ponceau S staining was used to detect loading control of WC-1 for Figure 3C. (h) Figure with the area highlighted was used to develop the Figure 3C for LC for WC-1. (i) Ponceau S staining was used to detect loading control of FRQ and WC-2 for Figure 3C. (j) Figure with the area highlighted was used as LC for FRQ and WC-2 for Figure 3C. [file elife-79765-fig3-data2.zip › 79765Figure3C/Figure_3-source_data_2h.jpg]

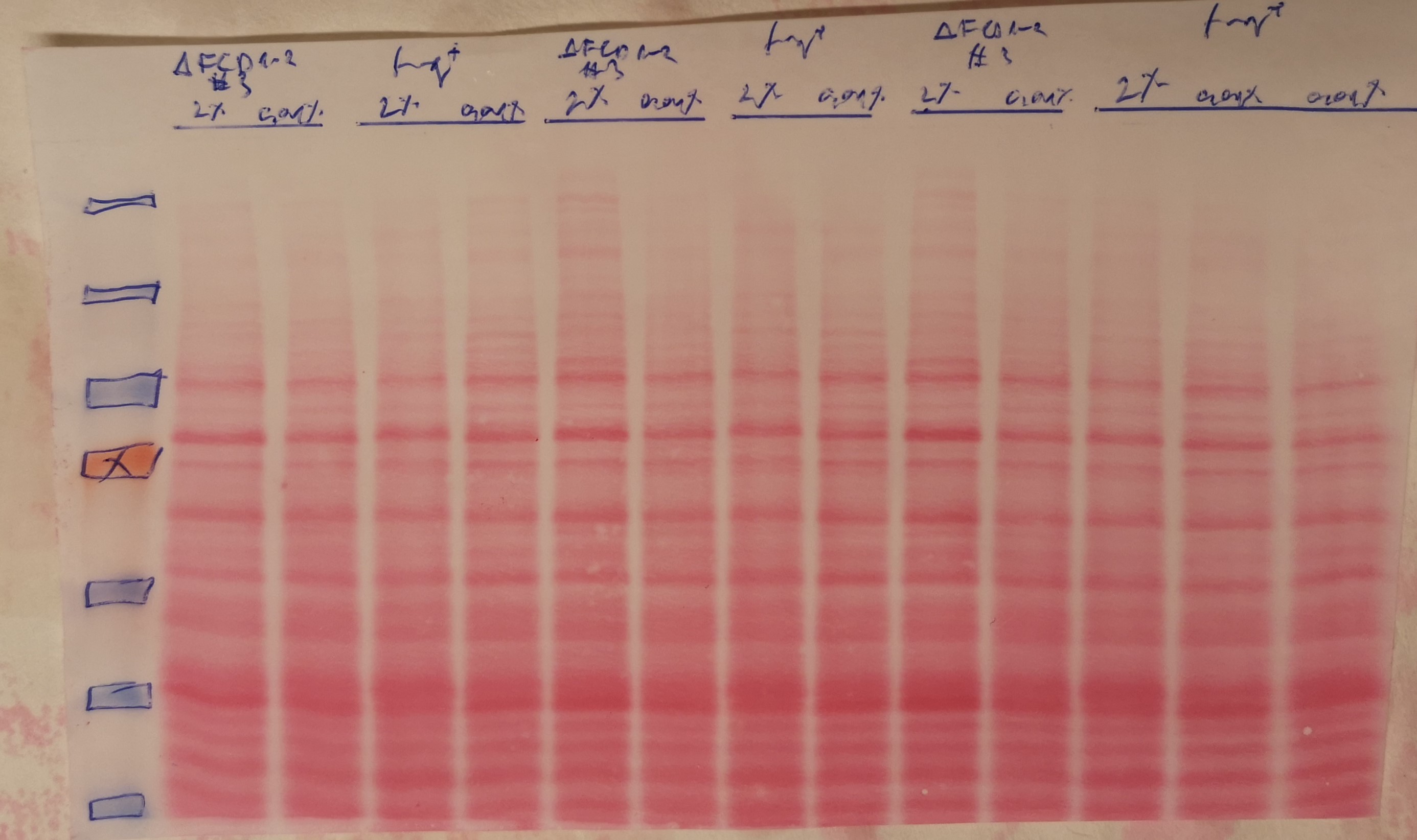

Supplement: Figure 3—source data 2. — (a) Western blots were used to detect expression of FRQ in the indicated samples for Figure 3C. (b) Figure with the area highlighted was used to develop the Figure 3C for FRQ. (c) Western blots were used to detect expression of WC-1 in the indicated samples for Figure 3C. (d) Figure with the area highlighted was used to develop the Figure 3C for WC-1. (e) Western blots were used to detect expression of WC-2 in the indicated samples for Figure 3C. (f) Figure with the area highlighted was used to develop the Figure 3C for WC-2. (g) Ponceau S staining was used to detect loading control of WC-1 for Figure 3C. (h) Figure with the area highlighted was used to develop the Figure 3C for LC for WC-1. (i) Ponceau S staining was used to detect loading control of FRQ and WC-2 for Figure 3C. (j) Figure with the area highlighted was used as LC for FRQ and WC-2 for Figure 3C. [file elife-79765-fig3-data2.zip › 79765Figure3C/Figure_3-source_data_2i.jpg]

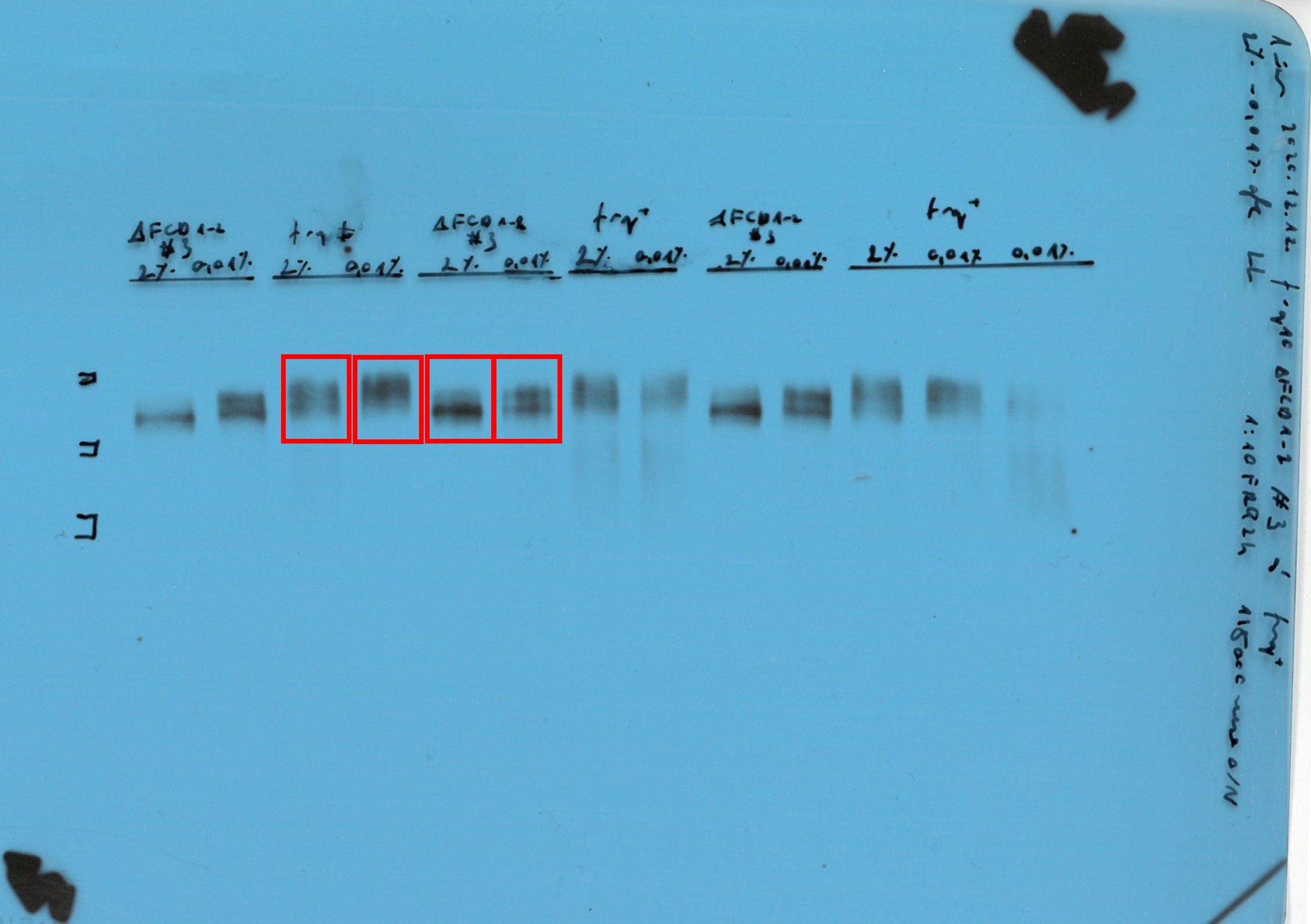

Supplement: Figure 3—source data 2. — (a) Western blots were used to detect expression of FRQ in the indicated samples for Figure 3C. (b) Figure with the area highlighted was used to develop the Figure 3C for FRQ. (c) Western blots were used to detect expression of WC-1 in the indicated samples for Figure 3C. (d) Figure with the area highlighted was used to develop the Figure 3C for WC-1. (e) Western blots were used to detect expression of WC-2 in the indicated samples for Figure 3C. (f) Figure with the area highlighted was used to develop the Figure 3C for WC-2. (g) Ponceau S staining was used to detect loading control of WC-1 for Figure 3C. (h) Figure with the area highlighted was used to develop the Figure 3C for LC for WC-1. (i) Ponceau S staining was used to detect loading control of FRQ and WC-2 for Figure 3C. (j) Figure with the area highlighted was used as LC for FRQ and WC-2 for Figure 3C. [file elife-79765-fig3-data2.zip › 79765Figure3C/Figure_3-source_data_2b.jpg]

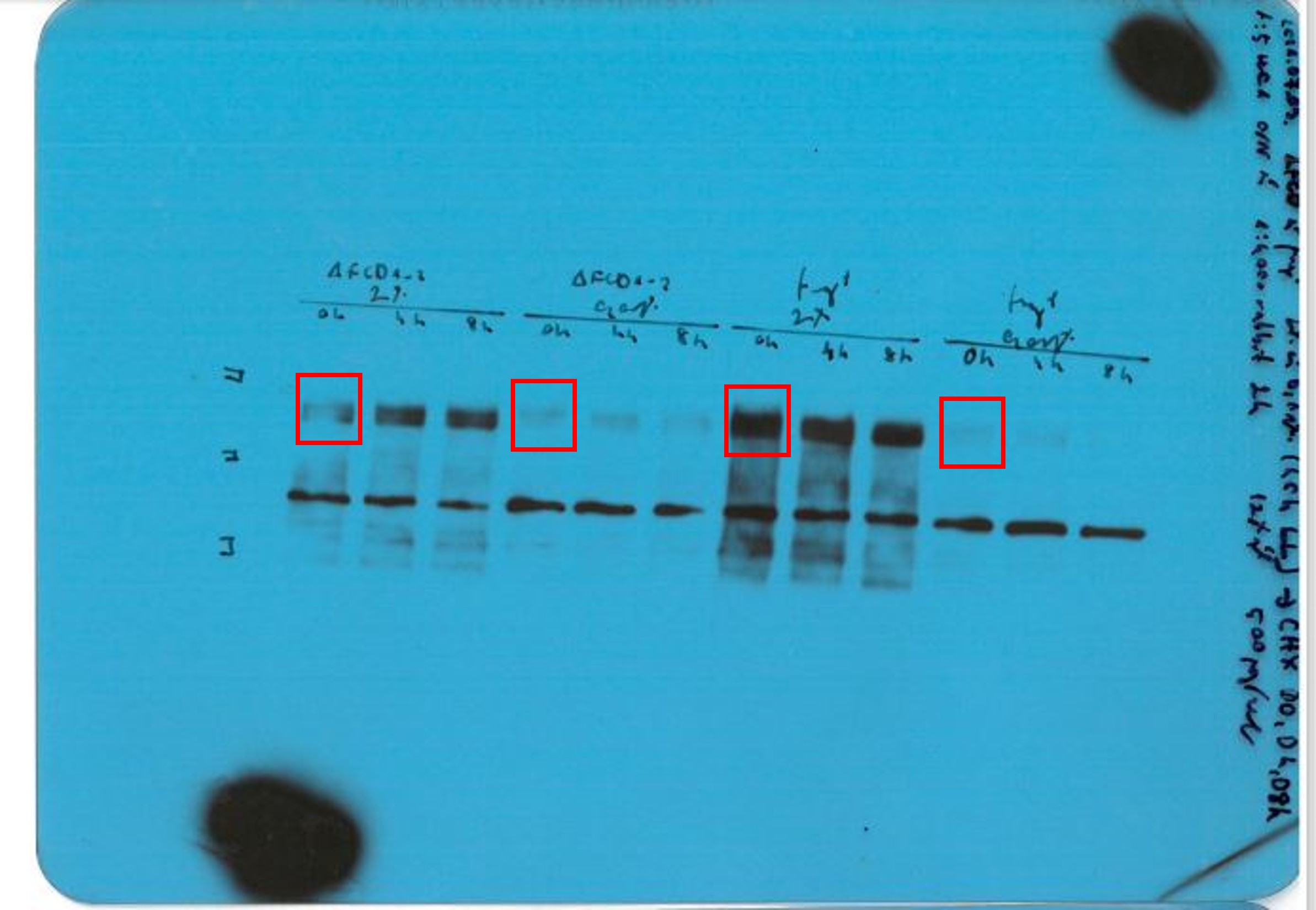

Supplement: Figure 3—source data 2. — (a) Western blots were used to detect expression of FRQ in the indicated samples for Figure 3C. (b) Figure with the area highlighted was used to develop the Figure 3C for FRQ. (c) Western blots were used to detect expression of WC-1 in the indicated samples for Figure 3C. (d) Figure with the area highlighted was used to develop the Figure 3C for WC-1. (e) Western blots were used to detect expression of WC-2 in the indicated samples for Figure 3C. (f) Figure with the area highlighted was used to develop the Figure 3C for WC-2. (g) Ponceau S staining was used to detect loading control of WC-1 for Figure 3C. (h) Figure with the area highlighted was used to develop the Figure 3C for LC for WC-1. (i) Ponceau S staining was used to detect loading control of FRQ and WC-2 for Figure 3C. (j) Figure with the area highlighted was used as LC for FRQ and WC-2 for Figure 3C. [file elife-79765-fig3-data2.zip › 79765Figure3C/Figure_3-source_data_2c.JPG]

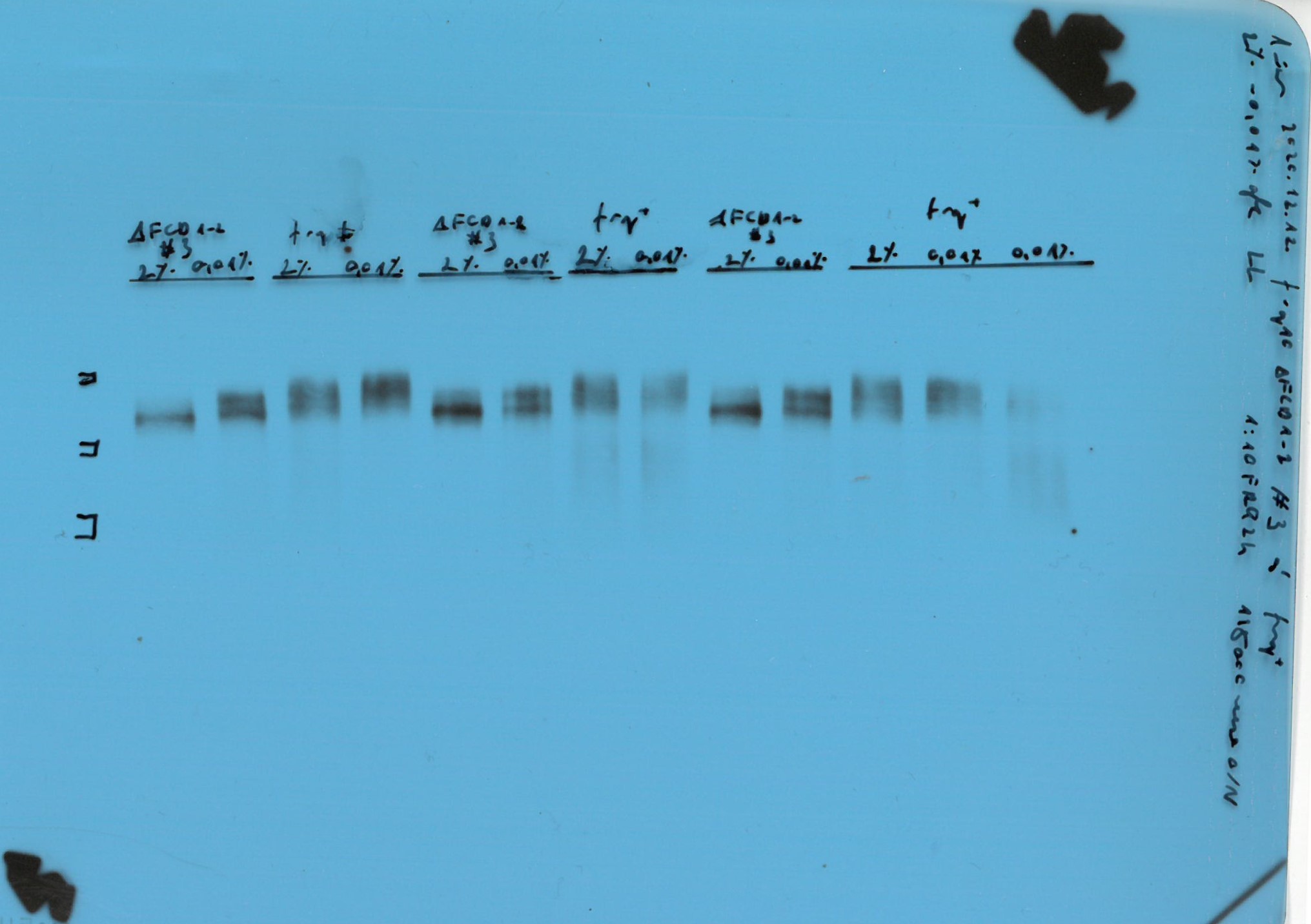

Supplement: Figure 3—source data 2. — (a) Western blots were used to detect expression of FRQ in the indicated samples for Figure 3C. (b) Figure with the area highlighted was used to develop the Figure 3C for FRQ. (c) Western blots were used to detect expression of WC-1 in the indicated samples for Figure 3C. (d) Figure with the area highlighted was used to develop the Figure 3C for WC-1. (e) Western blots were used to detect expression of WC-2 in the indicated samples for Figure 3C. (f) Figure with the area highlighted was used to develop the Figure 3C for WC-2. (g) Ponceau S staining was used to detect loading control of WC-1 for Figure 3C. (h) Figure with the area highlighted was used to develop the Figure 3C for LC for WC-1. (i) Ponceau S staining was used to detect loading control of FRQ and WC-2 for Figure 3C. (j) Figure with the area highlighted was used as LC for FRQ and WC-2 for Figure 3C. [file elife-79765-fig3-data2.zip › 79765Figure3C/Figure_3-source_data_2a.jpg]

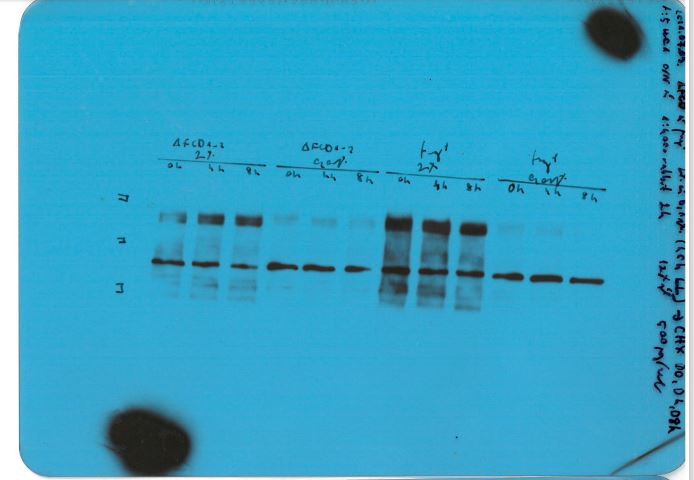

Supplement: Figure 3—source data 2. — (a) Western blots were used to detect expression of FRQ in the indicated samples for Figure 3C. (b) Figure with the area highlighted was used to develop the Figure 3C for FRQ. (c) Western blots were used to detect expression of WC-1 in the indicated samples for Figure 3C. (d) Figure with the area highlighted was used to develop the Figure 3C for WC-1. (e) Western blots were used to detect expression of WC-2 in the indicated samples for Figure 3C. (f) Figure with the area highlighted was used to develop the Figure 3C for WC-2. (g) Ponceau S staining was used to detect loading control of WC-1 for Figure 3C. (h) Figure with the area highlighted was used to develop the Figure 3C for LC for WC-1. (i) Ponceau S staining was used to detect loading control of FRQ and WC-2 for Figure 3C. (j) Figure with the area highlighted was used as LC for FRQ and WC-2 for Figure 3C. [file elife-79765-fig3-data2.zip › 79765Figure3C/Figure_3-source_data_2d.JPG]

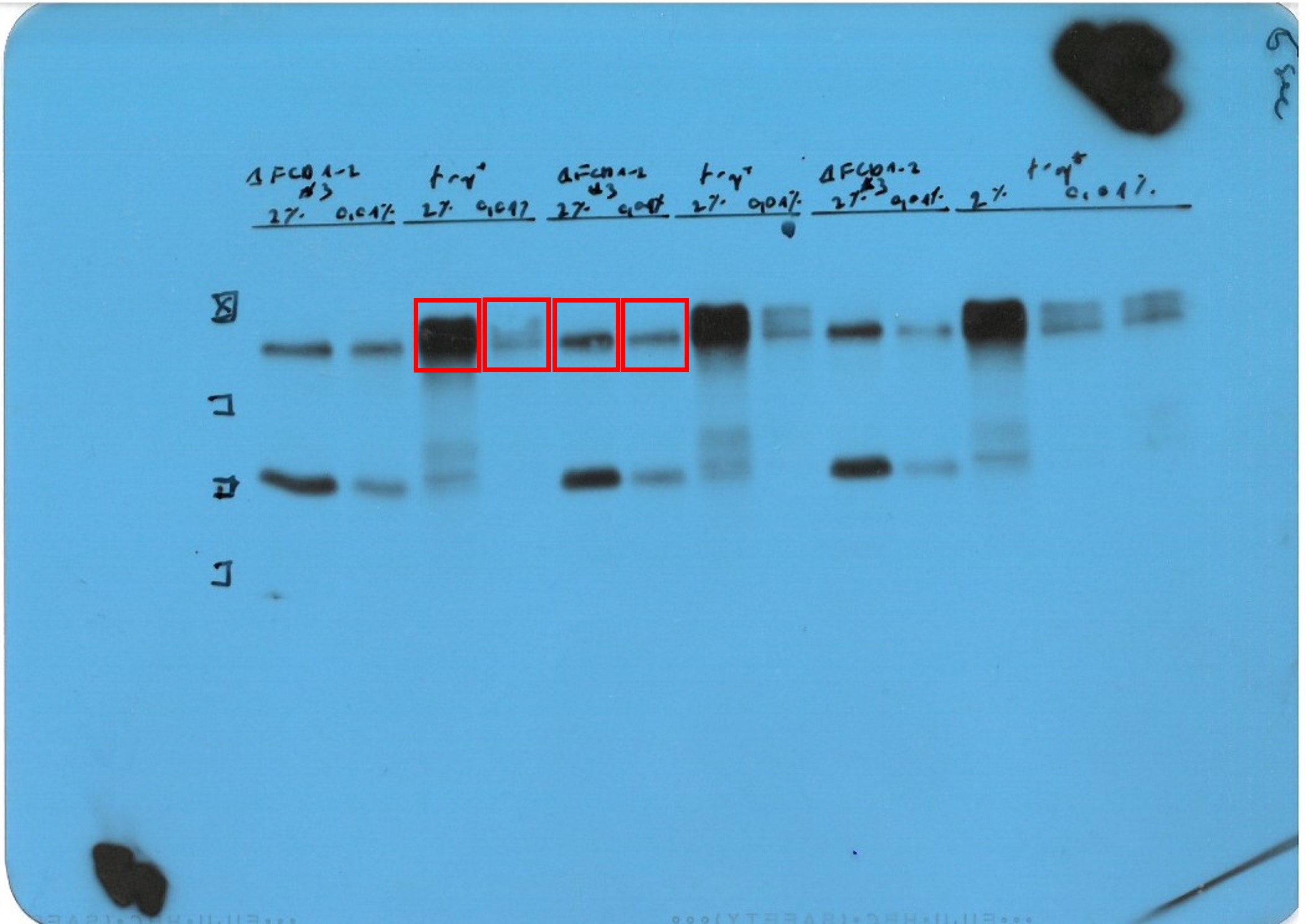

Supplement: Figure 3—source data 2. — (a) Western blots were used to detect expression of FRQ in the indicated samples for Figure 3C. (b) Figure with the area highlighted was used to develop the Figure 3C for FRQ. (c) Western blots were used to detect expression of WC-1 in the indicated samples for Figure 3C. (d) Figure with the area highlighted was used to develop the Figure 3C for WC-1. (e) Western blots were used to detect expression of WC-2 in the indicated samples for Figure 3C. (f) Figure with the area highlighted was used to develop the Figure 3C for WC-2. (g) Ponceau S staining was used to detect loading control of WC-1 for Figure 3C. (h) Figure with the area highlighted was used to develop the Figure 3C for LC for WC-1. (i) Ponceau S staining was used to detect loading control of FRQ and WC-2 for Figure 3C. (j) Figure with the area highlighted was used as LC for FRQ and WC-2 for Figure 3C. [file elife-79765-fig3-data2.zip › 79765Figure3C/Figure_3-source_data_2e.jpg]

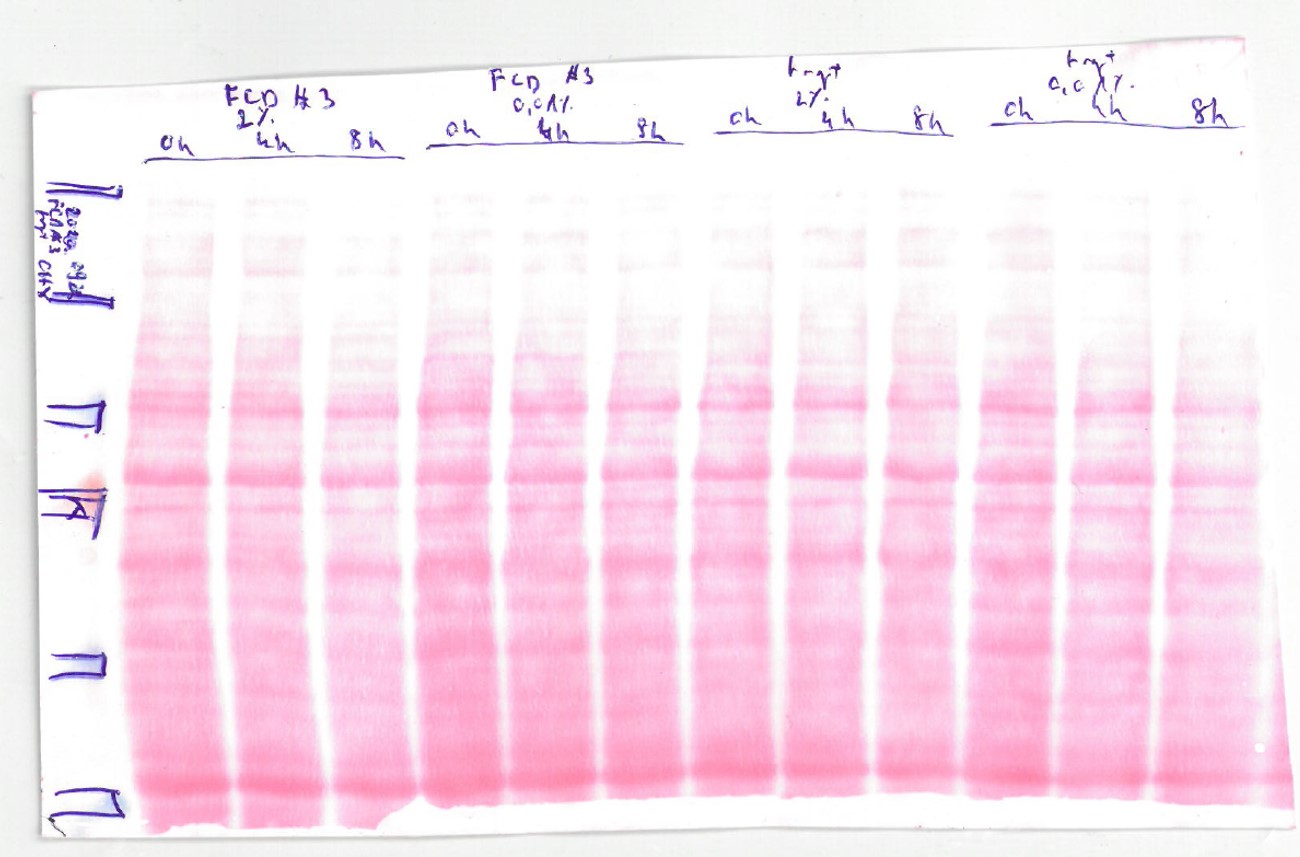

Supplement: Figure 3—source data 2. — (a) Western blots were used to detect expression of FRQ in the indicated samples for Figure 3C. (b) Figure with the area highlighted was used to develop the Figure 3C for FRQ. (c) Western blots were used to detect expression of WC-1 in the indicated samples for Figure 3C. (d) Figure with the area highlighted was used to develop the Figure 3C for WC-1. (e) Western blots were used to detect expression of WC-2 in the indicated samples for Figure 3C. (f) Figure with the area highlighted was used to develop the Figure 3C for WC-2. (g) Ponceau S staining was used to detect loading control of WC-1 for Figure 3C. (h) Figure with the area highlighted was used to develop the Figure 3C for LC for WC-1. (i) Ponceau S staining was used to detect loading control of FRQ and WC-2 for Figure 3C. (j) Figure with the area highlighted was used as LC for FRQ and WC-2 for Figure 3C. [file elife-79765-fig3-data2.zip › 79765Figure3C/Figure_3-source_data_2g.jpg]

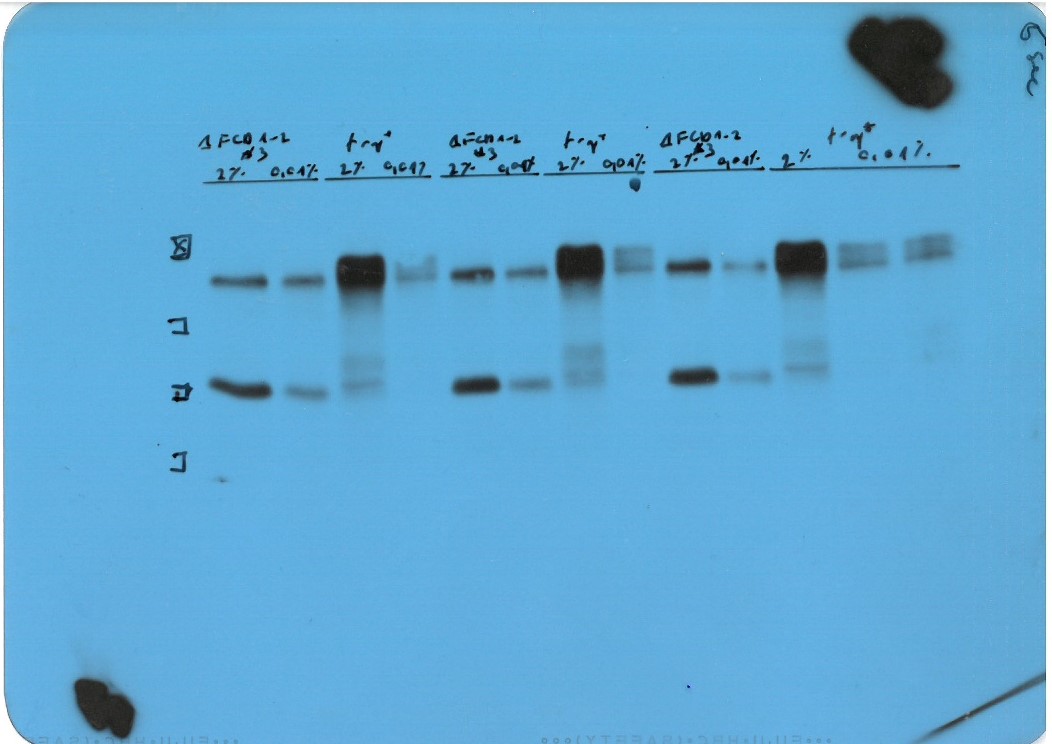

Supplement: Figure 3—source data 2. — (a) Western blots were used to detect expression of FRQ in the indicated samples for Figure 3C. (b) Figure with the area highlighted was used to develop the Figure 3C for FRQ. (c) Western blots were used to detect expression of WC-1 in the indicated samples for Figure 3C. (d) Figure with the area highlighted was used to develop the Figure 3C for WC-1. (e) Western blots were used to detect expression of WC-2 in the indicated samples for Figure 3C. (f) Figure with the area highlighted was used to develop the Figure 3C for WC-2. (g) Ponceau S staining was used to detect loading control of WC-1 for Figure 3C. (h) Figure with the area highlighted was used to develop the Figure 3C for LC for WC-1. (i) Ponceau S staining was used to detect loading control of FRQ and WC-2 for Figure 3C. (j) Figure with the area highlighted was used as LC for FRQ and WC-2 for Figure 3C. [file elife-79765-fig3-data2.zip › 79765Figure3C/Figure_3-source_data_2f.jpg]

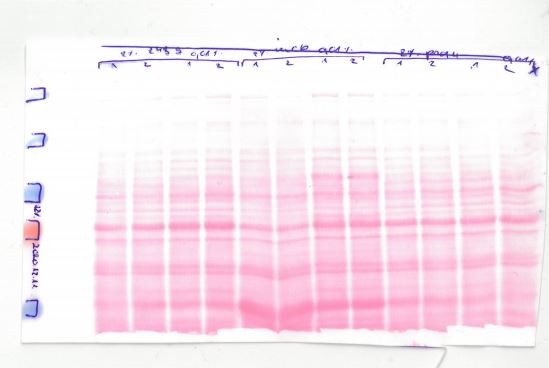

Supplement: Figure 3—source data 3. — (a) Western blots were used to detect expression of FRQ in the indicated samples for Figure 3D (s: short exposure). (b) Figure with the area highlighted was used to develop the Figure 3D for FRQ (s: short exposure). (c) Western blots were used to detect expression of FRQ in the indicated samples for Figure 3D (l: long exposure). (d) Figure with the area highlighted was used to develop the Figure 3D for FRQ (l: long exposure). (e) Western blots were used to detect expression of WC-1 in the indicated samples for Figure 3D. (f) Figure with the area highlighted was used to develop the Figure 3D for WC-1. (g) Western blots were used to detect expression of WC-2 in the indicated samples for Figure 3D. (h) Figure with the area highlighted was used to develop the Figure 3D for WC-2. (i) Ponceau S staining was used to detect loading control of the indicated samples for Figure 3D. (j) Figure with the area highlighted was used as LC for FRQ in Figure 3D. (k) Figure with the area highlighted was used as LC for WC-1 and for WC-2 in Figure 3D . [file elife-79765-fig3-data3.zip › 79765Figure3D/Figure_3-source_data_3i.JPG]

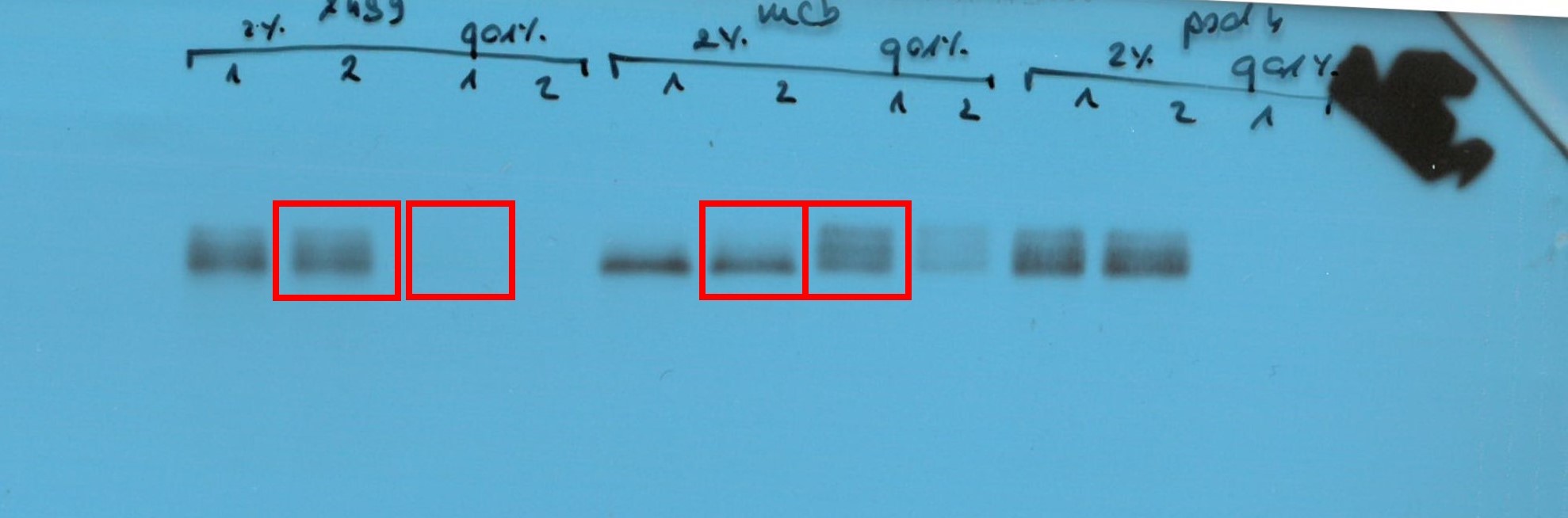

Supplement: Figure 3—source data 3. — (a) Western blots were used to detect expression of FRQ in the indicated samples for Figure 3D (s: short exposure). (b) Figure with the area highlighted was used to develop the Figure 3D for FRQ (s: short exposure). (c) Western blots were used to detect expression of FRQ in the indicated samples for Figure 3D (l: long exposure). (d) Figure with the area highlighted was used to develop the Figure 3D for FRQ (l: long exposure). (e) Western blots were used to detect expression of WC-1 in the indicated samples for Figure 3D. (f) Figure with the area highlighted was used to develop the Figure 3D for WC-1. (g) Western blots were used to detect expression of WC-2 in the indicated samples for Figure 3D. (h) Figure with the area highlighted was used to develop the Figure 3D for WC-2. (i) Ponceau S staining was used to detect loading control of the indicated samples for Figure 3D. (j) Figure with the area highlighted was used as LC for FRQ in Figure 3D. (k) Figure with the area highlighted was used as LC for WC-1 and for WC-2 in Figure 3D . [file elife-79765-fig3-data3.zip › 79765Figure3D/Figure_3-source_data_3h.jpg]

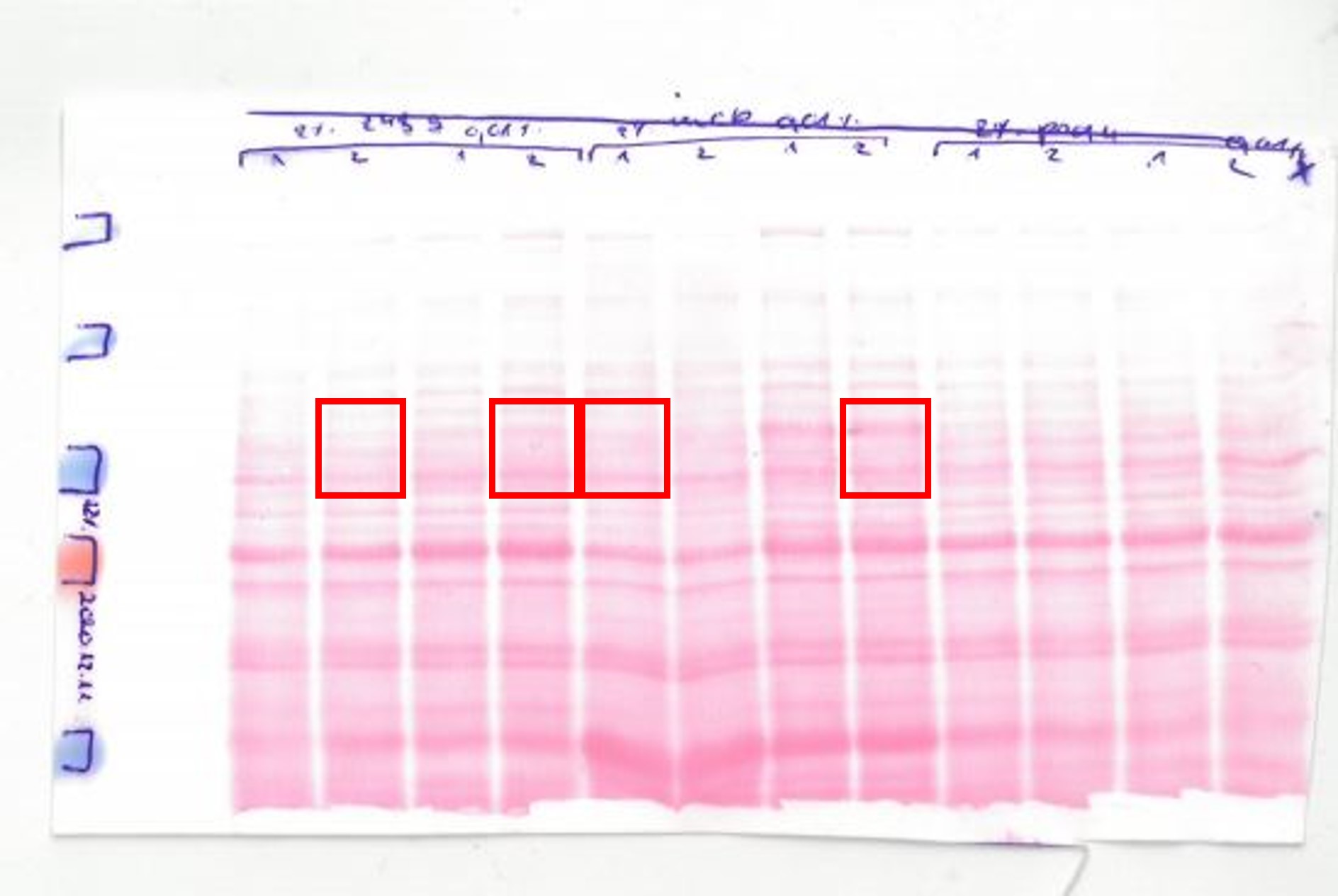

Supplement: Figure 3—source data 3. — (a) Western blots were used to detect expression of FRQ in the indicated samples for Figure 3D (s: short exposure). (b) Figure with the area highlighted was used to develop the Figure 3D for FRQ (s: short exposure). (c) Western blots were used to detect expression of FRQ in the indicated samples for Figure 3D (l: long exposure). (d) Figure with the area highlighted was used to develop the Figure 3D for FRQ (l: long exposure). (e) Western blots were used to detect expression of WC-1 in the indicated samples for Figure 3D. (f) Figure with the area highlighted was used to develop the Figure 3D for WC-1. (g) Western blots were used to detect expression of WC-2 in the indicated samples for Figure 3D. (h) Figure with the area highlighted was used to develop the Figure 3D for WC-2. (i) Ponceau S staining was used to detect loading control of the indicated samples for Figure 3D. (j) Figure with the area highlighted was used as LC for FRQ in Figure 3D. (k) Figure with the area highlighted was used as LC for WC-1 and for WC-2 in Figure 3D . [file elife-79765-fig3-data3.zip › 79765Figure3D/Figure_3-source_data_3j.jpg]

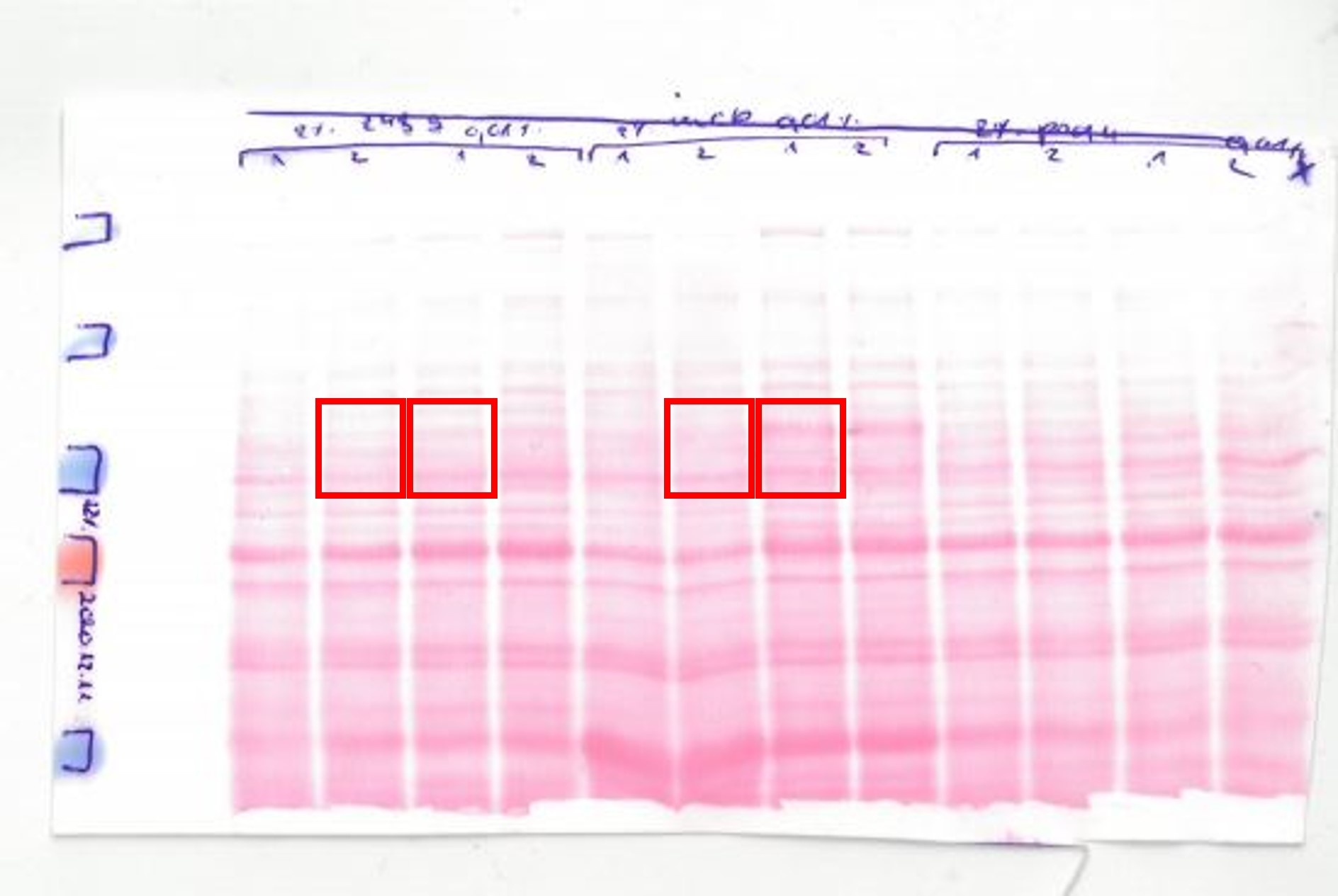

Supplement: Figure 3—source data 3. — (a) Western blots were used to detect expression of FRQ in the indicated samples for Figure 3D (s: short exposure). (b) Figure with the area highlighted was used to develop the Figure 3D for FRQ (s: short exposure). (c) Western blots were used to detect expression of FRQ in the indicated samples for Figure 3D (l: long exposure). (d) Figure with the area highlighted was used to develop the Figure 3D for FRQ (l: long exposure). (e) Western blots were used to detect expression of WC-1 in the indicated samples for Figure 3D. (f) Figure with the area highlighted was used to develop the Figure 3D for WC-1. (g) Western blots were used to detect expression of WC-2 in the indicated samples for Figure 3D. (h) Figure with the area highlighted was used to develop the Figure 3D for WC-2. (i) Ponceau S staining was used to detect loading control of the indicated samples for Figure 3D. (j) Figure with the area highlighted was used as LC for FRQ in Figure 3D. (k) Figure with the area highlighted was used as LC for WC-1 and for WC-2 in Figure 3D . [file elife-79765-fig3-data3.zip › 79765Figure3D/Figure_3-source_data_3k.jpg]

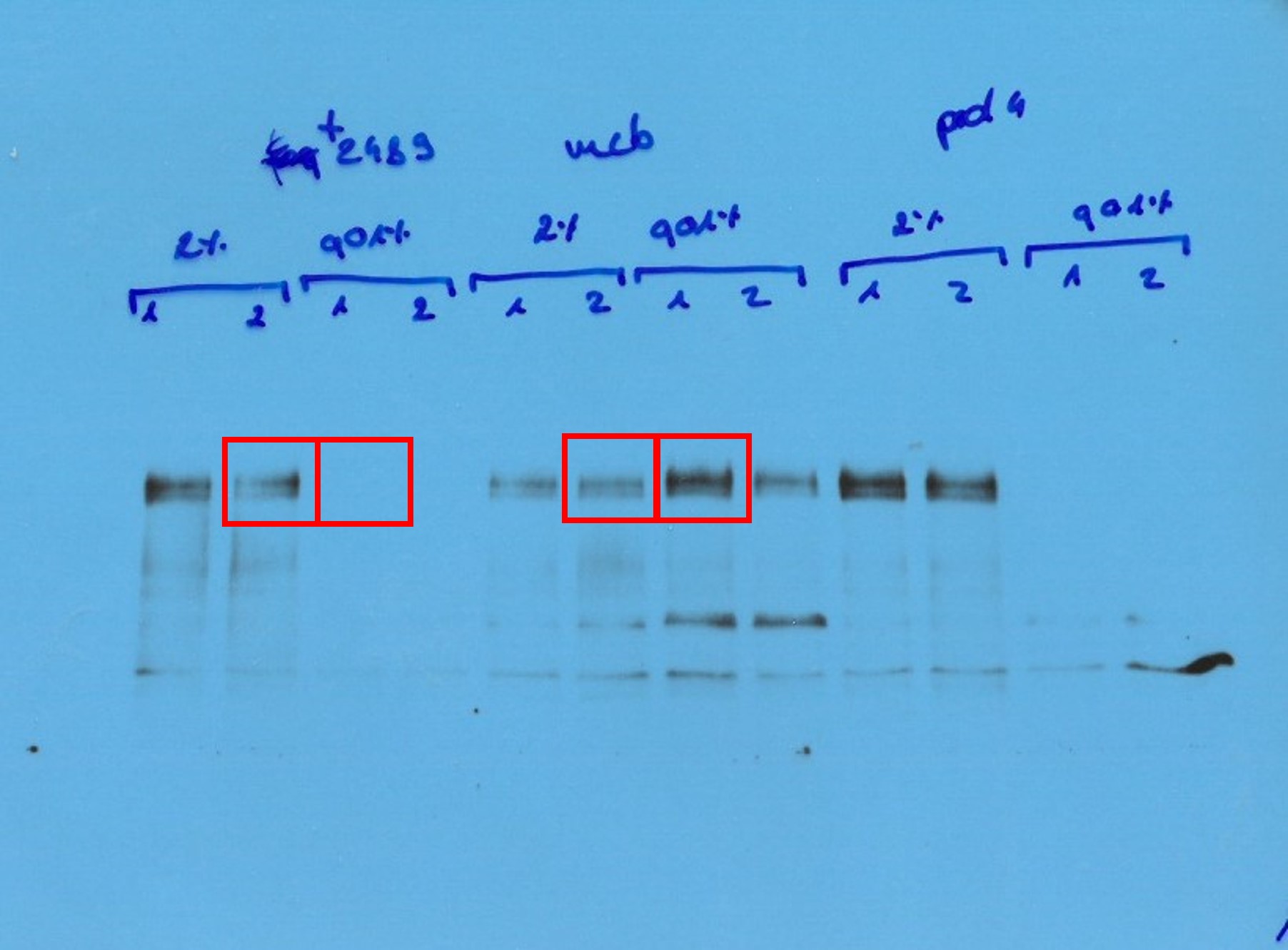

Supplement: Figure 3—source data 3. — (a) Western blots were used to detect expression of FRQ in the indicated samples for Figure 3D (s: short exposure). (b) Figure with the area highlighted was used to develop the Figure 3D for FRQ (s: short exposure). (c) Western blots were used to detect expression of FRQ in the indicated samples for Figure 3D (l: long exposure). (d) Figure with the area highlighted was used to develop the Figure 3D for FRQ (l: long exposure). (e) Western blots were used to detect expression of WC-1 in the indicated samples for Figure 3D. (f) Figure with the area highlighted was used to develop the Figure 3D for WC-1. (g) Western blots were used to detect expression of WC-2 in the indicated samples for Figure 3D. (h) Figure with the area highlighted was used to develop the Figure 3D for WC-2. (i) Ponceau S staining was used to detect loading control of the indicated samples for Figure 3D. (j) Figure with the area highlighted was used as LC for FRQ in Figure 3D. (k) Figure with the area highlighted was used as LC for WC-1 and for WC-2 in Figure 3D . [file elife-79765-fig3-data3.zip › 79765Figure3D/Figure_3-source_data_3f.jpg]

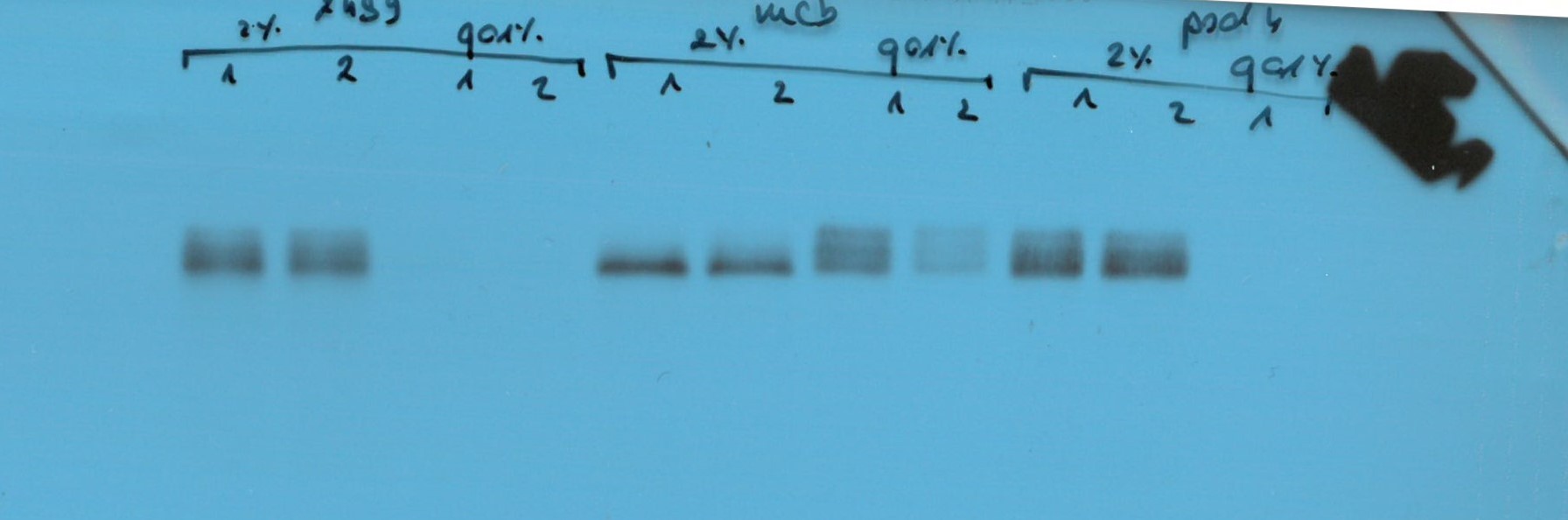

Supplement: Figure 3—source data 3. — (a) Western blots were used to detect expression of FRQ in the indicated samples for Figure 3D (s: short exposure). (b) Figure with the area highlighted was used to develop the Figure 3D for FRQ (s: short exposure). (c) Western blots were used to detect expression of FRQ in the indicated samples for Figure 3D (l: long exposure). (d) Figure with the area highlighted was used to develop the Figure 3D for FRQ (l: long exposure). (e) Western blots were used to detect expression of WC-1 in the indicated samples for Figure 3D. (f) Figure with the area highlighted was used to develop the Figure 3D for WC-1. (g) Western blots were used to detect expression of WC-2 in the indicated samples for Figure 3D. (h) Figure with the area highlighted was used to develop the Figure 3D for WC-2. (i) Ponceau S staining was used to detect loading control of the indicated samples for Figure 3D. (j) Figure with the area highlighted was used as LC for FRQ in Figure 3D. (k) Figure with the area highlighted was used as LC for WC-1 and for WC-2 in Figure 3D . [file elife-79765-fig3-data3.zip › 79765Figure3D/Figure_3-source_data_3g.jpg]

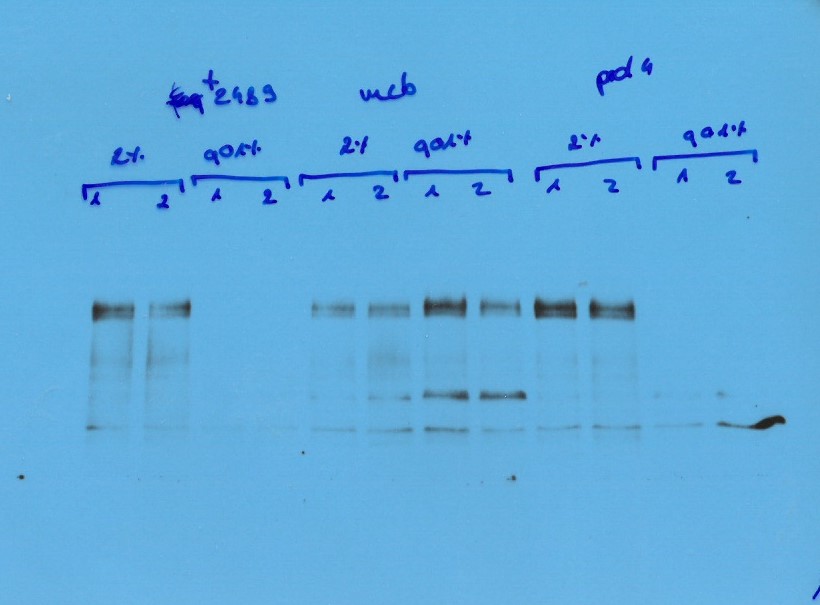

Supplement: Figure 3—source data 3. — (a) Western blots were used to detect expression of FRQ in the indicated samples for Figure 3D (s: short exposure). (b) Figure with the area highlighted was used to develop the Figure 3D for FRQ (s: short exposure). (c) Western blots were used to detect expression of FRQ in the indicated samples for Figure 3D (l: long exposure). (d) Figure with the area highlighted was used to develop the Figure 3D for FRQ (l: long exposure). (e) Western blots were used to detect expression of WC-1 in the indicated samples for Figure 3D. (f) Figure with the area highlighted was used to develop the Figure 3D for WC-1. (g) Western blots were used to detect expression of WC-2 in the indicated samples for Figure 3D. (h) Figure with the area highlighted was used to develop the Figure 3D for WC-2. (i) Ponceau S staining was used to detect loading control of the indicated samples for Figure 3D. (j) Figure with the area highlighted was used as LC for FRQ in Figure 3D. (k) Figure with the area highlighted was used as LC for WC-1 and for WC-2 in Figure 3D . [file elife-79765-fig3-data3.zip › 79765Figure3D/Figure_3-source_data_3e.jpg]

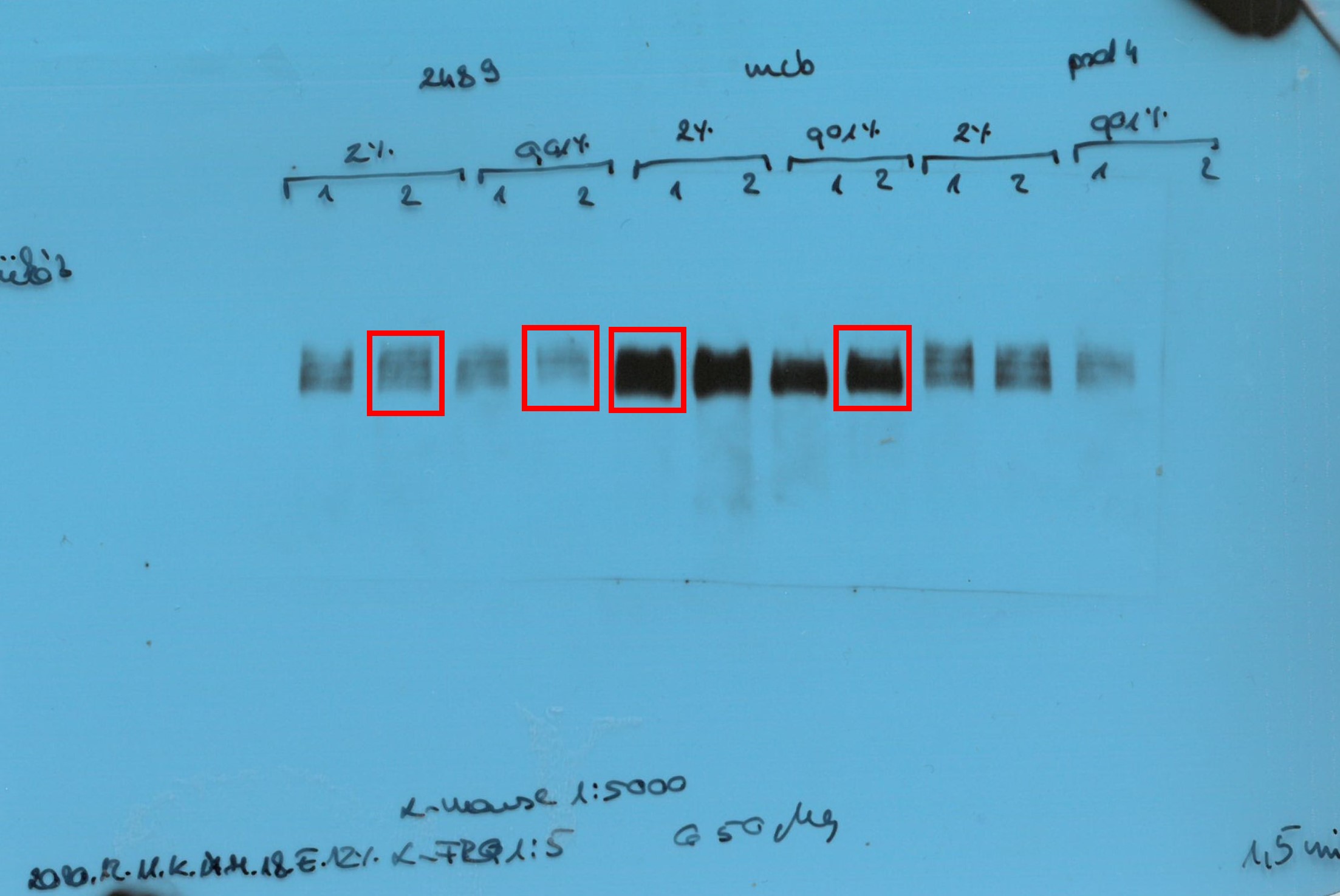

Supplement: Figure 3—source data 3. — (a) Western blots were used to detect expression of FRQ in the indicated samples for Figure 3D (s: short exposure). (b) Figure with the area highlighted was used to develop the Figure 3D for FRQ (s: short exposure). (c) Western blots were used to detect expression of FRQ in the indicated samples for Figure 3D (l: long exposure). (d) Figure with the area highlighted was used to develop the Figure 3D for FRQ (l: long exposure). (e) Western blots were used to detect expression of WC-1 in the indicated samples for Figure 3D. (f) Figure with the area highlighted was used to develop the Figure 3D for WC-1. (g) Western blots were used to detect expression of WC-2 in the indicated samples for Figure 3D. (h) Figure with the area highlighted was used to develop the Figure 3D for WC-2. (i) Ponceau S staining was used to detect loading control of the indicated samples for Figure 3D. (j) Figure with the area highlighted was used as LC for FRQ in Figure 3D. (k) Figure with the area highlighted was used as LC for WC-1 and for WC-2 in Figure 3D . [file elife-79765-fig3-data3.zip › 79765Figure3D/Figure_3-source_data_3d.jpg]

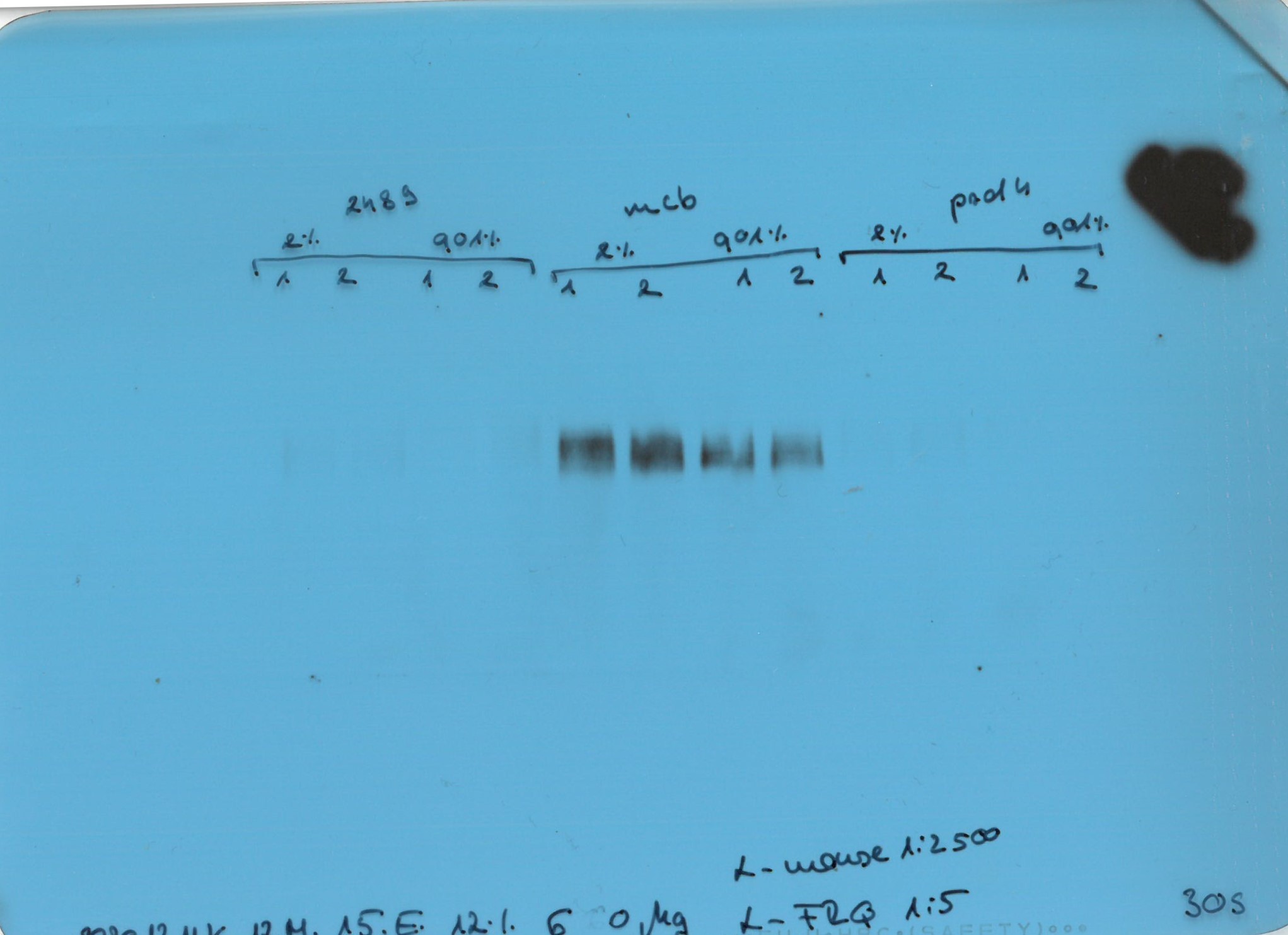

Supplement: Figure 3—source data 3. — (a) Western blots were used to detect expression of FRQ in the indicated samples for Figure 3D (s: short exposure). (b) Figure with the area highlighted was used to develop the Figure 3D for FRQ (s: short exposure). (c) Western blots were used to detect expression of FRQ in the indicated samples for Figure 3D (l: long exposure). (d) Figure with the area highlighted was used to develop the Figure 3D for FRQ (l: long exposure). (e) Western blots were used to detect expression of WC-1 in the indicated samples for Figure 3D. (f) Figure with the area highlighted was used to develop the Figure 3D for WC-1. (g) Western blots were used to detect expression of WC-2 in the indicated samples for Figure 3D. (h) Figure with the area highlighted was used to develop the Figure 3D for WC-2. (i) Ponceau S staining was used to detect loading control of the indicated samples for Figure 3D. (j) Figure with the area highlighted was used as LC for FRQ in Figure 3D. (k) Figure with the area highlighted was used as LC for WC-1 and for WC-2 in Figure 3D . [file elife-79765-fig3-data3.zip › 79765Figure3D/Figure_3-source_data_3a.jpg]

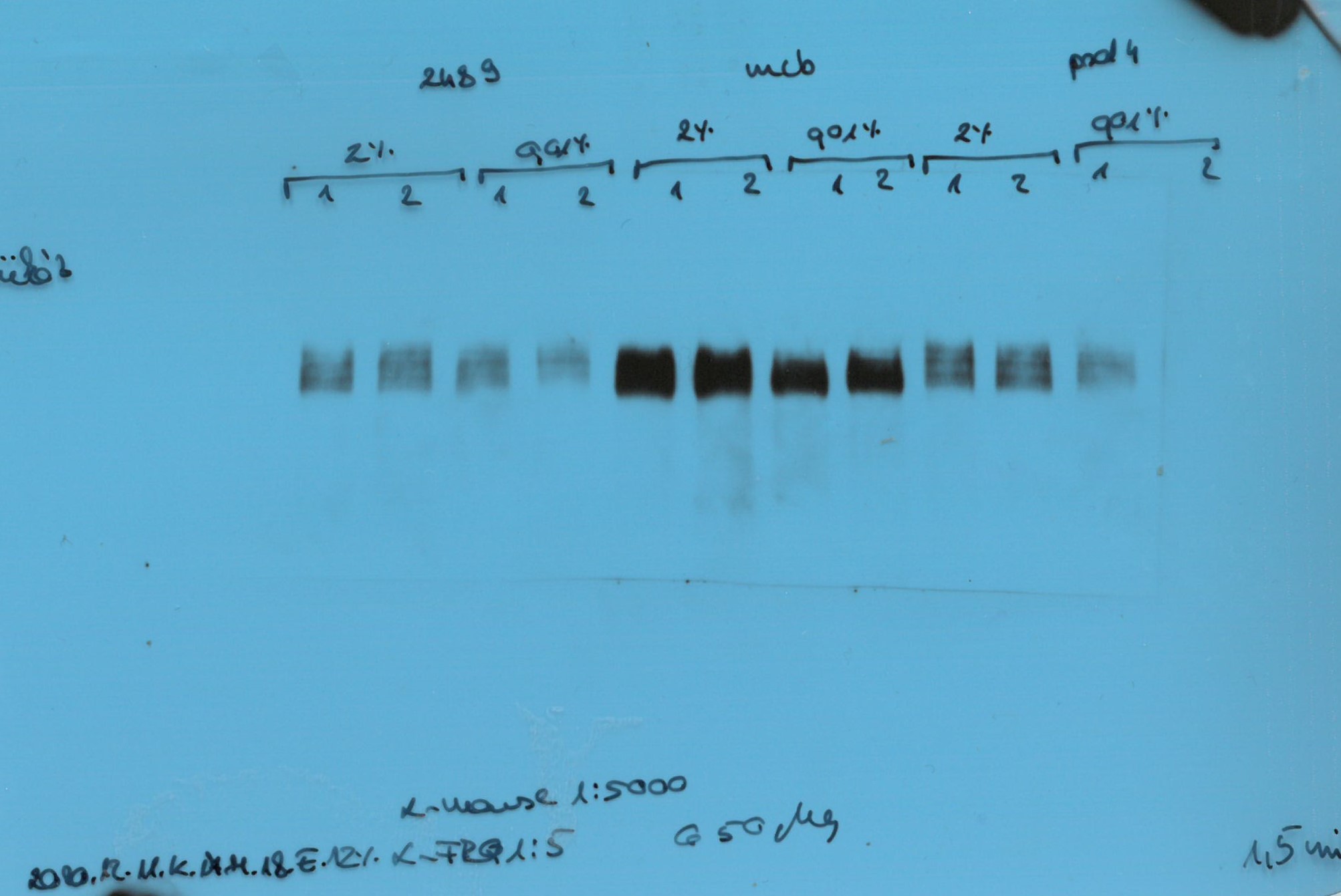

Supplement: Figure 3—source data 3. — (a) Western blots were used to detect expression of FRQ in the indicated samples for Figure 3D (s: short exposure). (b) Figure with the area highlighted was used to develop the Figure 3D for FRQ (s: short exposure). (c) Western blots were used to detect expression of FRQ in the indicated samples for Figure 3D (l: long exposure). (d) Figure with the area highlighted was used to develop the Figure 3D for FRQ (l: long exposure). (e) Western blots were used to detect expression of WC-1 in the indicated samples for Figure 3D. (f) Figure with the area highlighted was used to develop the Figure 3D for WC-1. (g) Western blots were used to detect expression of WC-2 in the indicated samples for Figure 3D. (h) Figure with the area highlighted was used to develop the Figure 3D for WC-2. (i) Ponceau S staining was used to detect loading control of the indicated samples for Figure 3D. (j) Figure with the area highlighted was used as LC for FRQ in Figure 3D. (k) Figure with the area highlighted was used as LC for WC-1 and for WC-2 in Figure 3D . [file elife-79765-fig3-data3.zip › 79765Figure3D/Figure_3-source_data_3c.jpg]

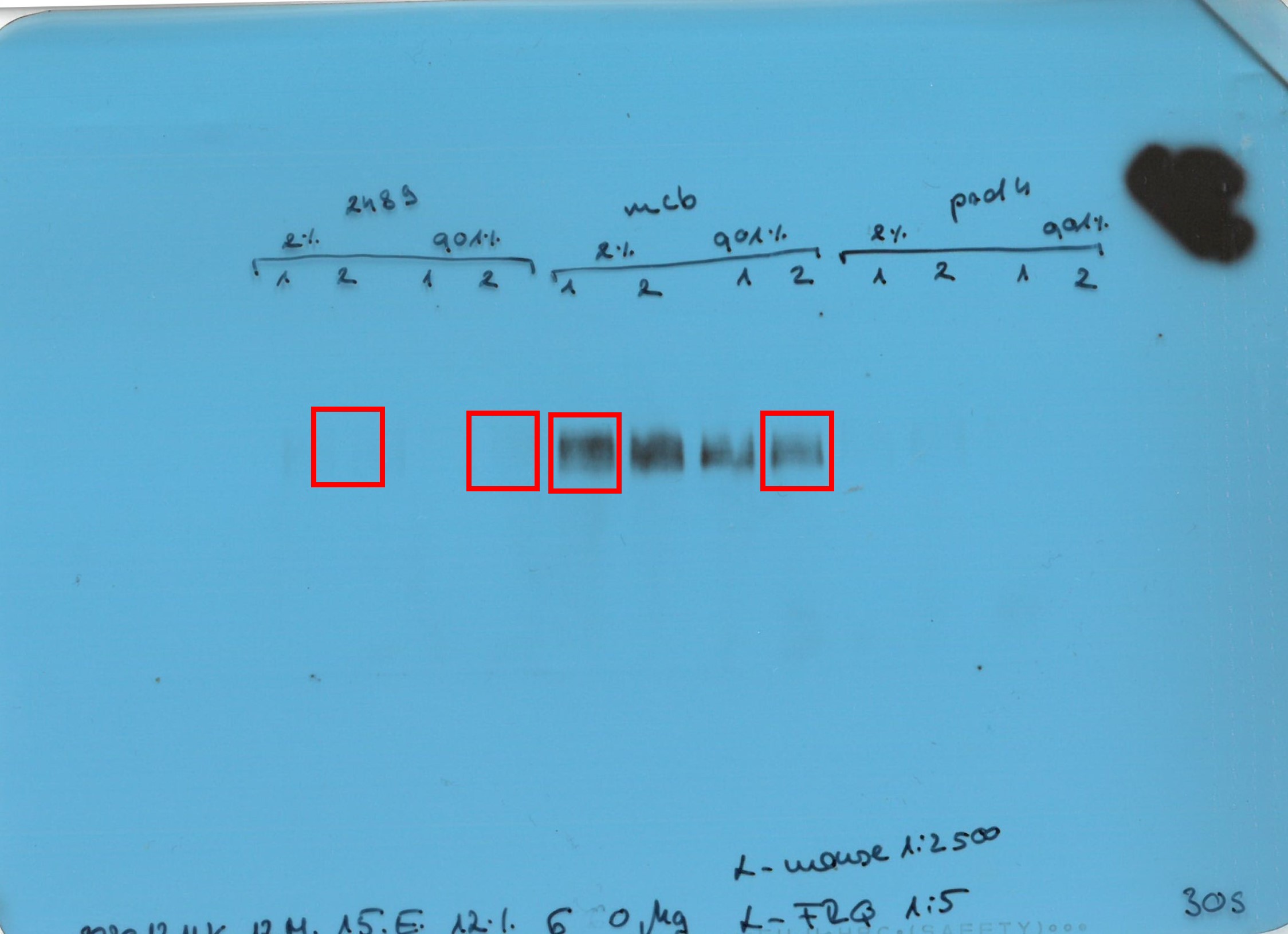

Supplement: Figure 3—source data 3. — (a) Western blots were used to detect expression of FRQ in the indicated samples for Figure 3D (s: short exposure). (b) Figure with the area highlighted was used to develop the Figure 3D for FRQ (s: short exposure). (c) Western blots were used to detect expression of FRQ in the indicated samples for Figure 3D (l: long exposure). (d) Figure with the area highlighted was used to develop the Figure 3D for FRQ (l: long exposure). (e) Western blots were used to detect expression of WC-1 in the indicated samples for Figure 3D. (f) Figure with the area highlighted was used to develop the Figure 3D for WC-1. (g) Western blots were used to detect expression of WC-2 in the indicated samples for Figure 3D. (h) Figure with the area highlighted was used to develop the Figure 3D for WC-2. (i) Ponceau S staining was used to detect loading control of the indicated samples for Figure 3D. (j) Figure with the area highlighted was used as LC for FRQ in Figure 3D. (k) Figure with the area highlighted was used as LC for WC-1 and for WC-2 in Figure 3D . [file elife-79765-fig3-data3.zip › 79765Figure3D/Figure_3-source_data_3b.jpg]

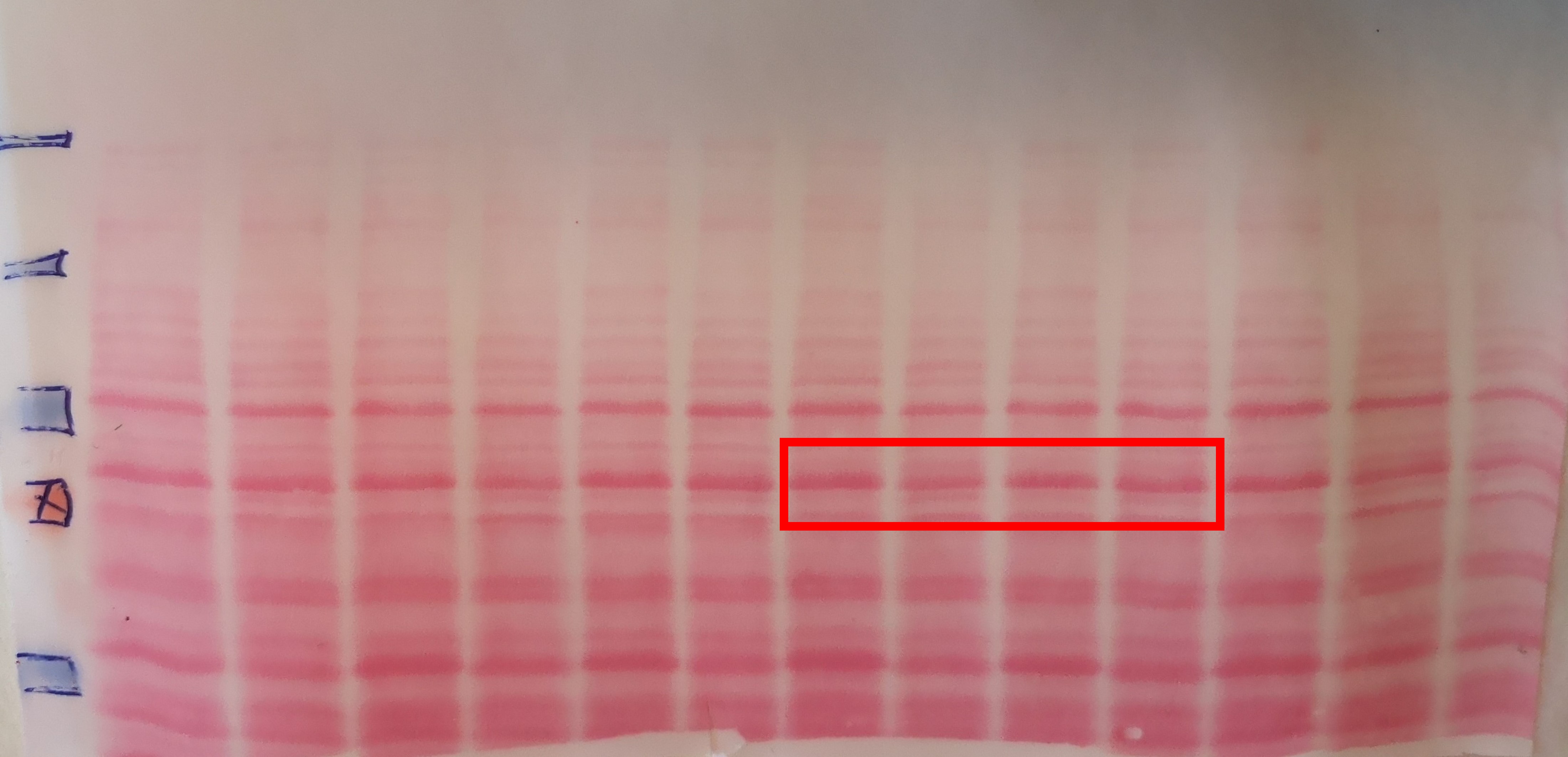

Supplement: Figure 3—source data 4. — (a) Western blots were used to detect expression of FRQ in the indicated samples for Figure 3E. (b) Figure with the area highlighted was used to develop the Figure 3E for FRQ. (c) Western blots were used to detect expression of WC-1 in the indicated samples for Figure 3E. (d) Figure with the area highlighted was used to develop the Figure 3E for WC-1. (e) Western blots were used to detect expression of WC-2 in the indicated samples for Figure 3E. (f) Figure with the area highlighted was used to develop the Figure 3E for WC-2. (g) Ponceau S staining was used to detect loading control of the indicated samples for Figure 3E. (h) Figure with the area highlighted was used as LC of Figure 3E. [file elife-79765-fig3-data4.zip › 79765Figure3E/Figure_3-source_data_4h.jpg]

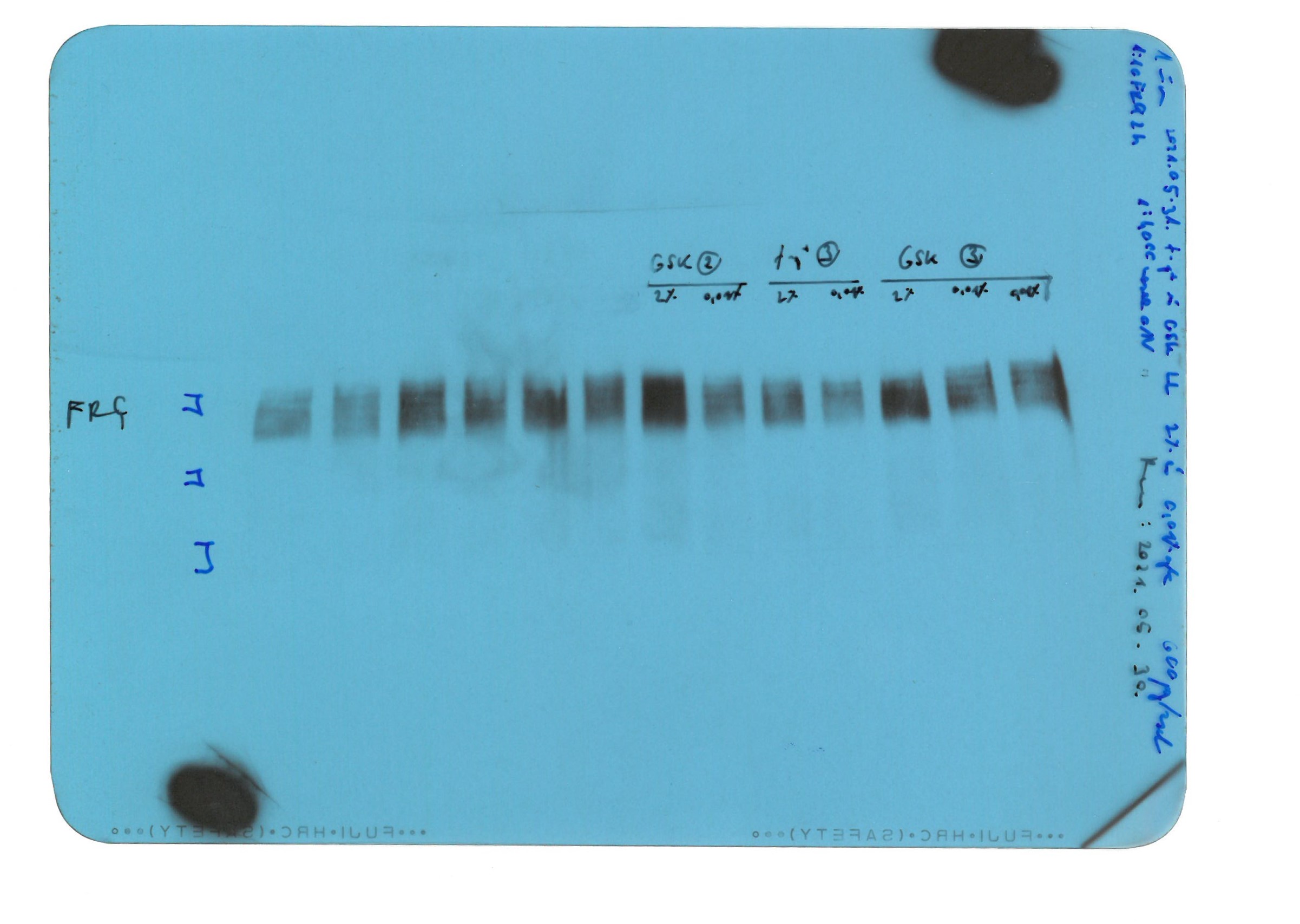

Supplement: Figure 3—source data 4. — (a) Western blots were used to detect expression of FRQ in the indicated samples for Figure 3E. (b) Figure with the area highlighted was used to develop the Figure 3E for FRQ. (c) Western blots were used to detect expression of WC-1 in the indicated samples for Figure 3E. (d) Figure with the area highlighted was used to develop the Figure 3E for WC-1. (e) Western blots were used to detect expression of WC-2 in the indicated samples for Figure 3E. (f) Figure with the area highlighted was used to develop the Figure 3E for WC-2. (g) Ponceau S staining was used to detect loading control of the indicated samples for Figure 3E. (h) Figure with the area highlighted was used as LC of Figure 3E. [file elife-79765-fig3-data4.zip › 79765Figure3E/Figure_3-source_data_4a.jpg]

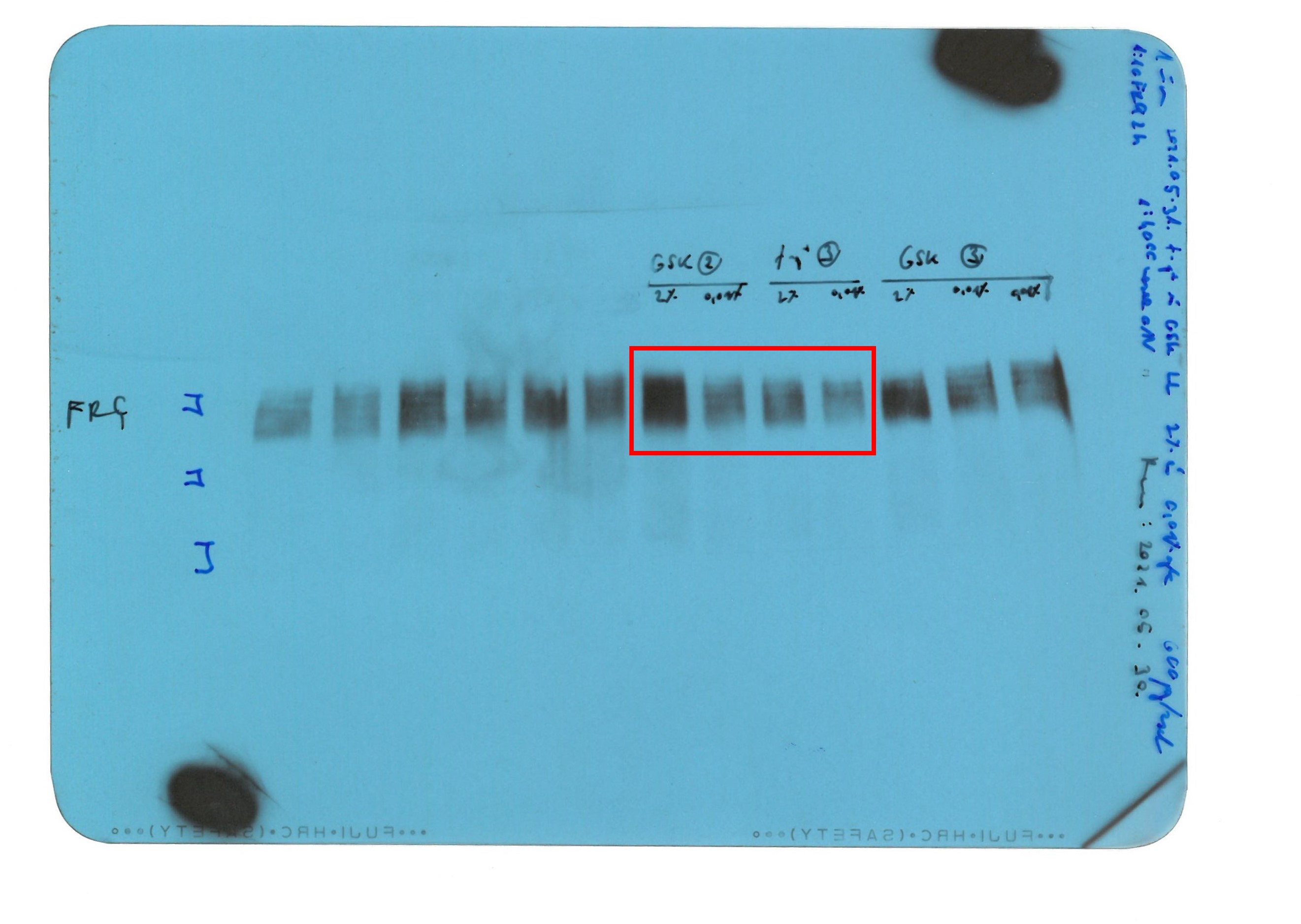

Supplement: Figure 3—source data 4. — (a) Western blots were used to detect expression of FRQ in the indicated samples for Figure 3E. (b) Figure with the area highlighted was used to develop the Figure 3E for FRQ. (c) Western blots were used to detect expression of WC-1 in the indicated samples for Figure 3E. (d) Figure with the area highlighted was used to develop the Figure 3E for WC-1. (e) Western blots were used to detect expression of WC-2 in the indicated samples for Figure 3E. (f) Figure with the area highlighted was used to develop the Figure 3E for WC-2. (g) Ponceau S staining was used to detect loading control of the indicated samples for Figure 3E. (h) Figure with the area highlighted was used as LC of Figure 3E. [file elife-79765-fig3-data4.zip › 79765Figure3E/Figure_3-source_data_4b.jpg]

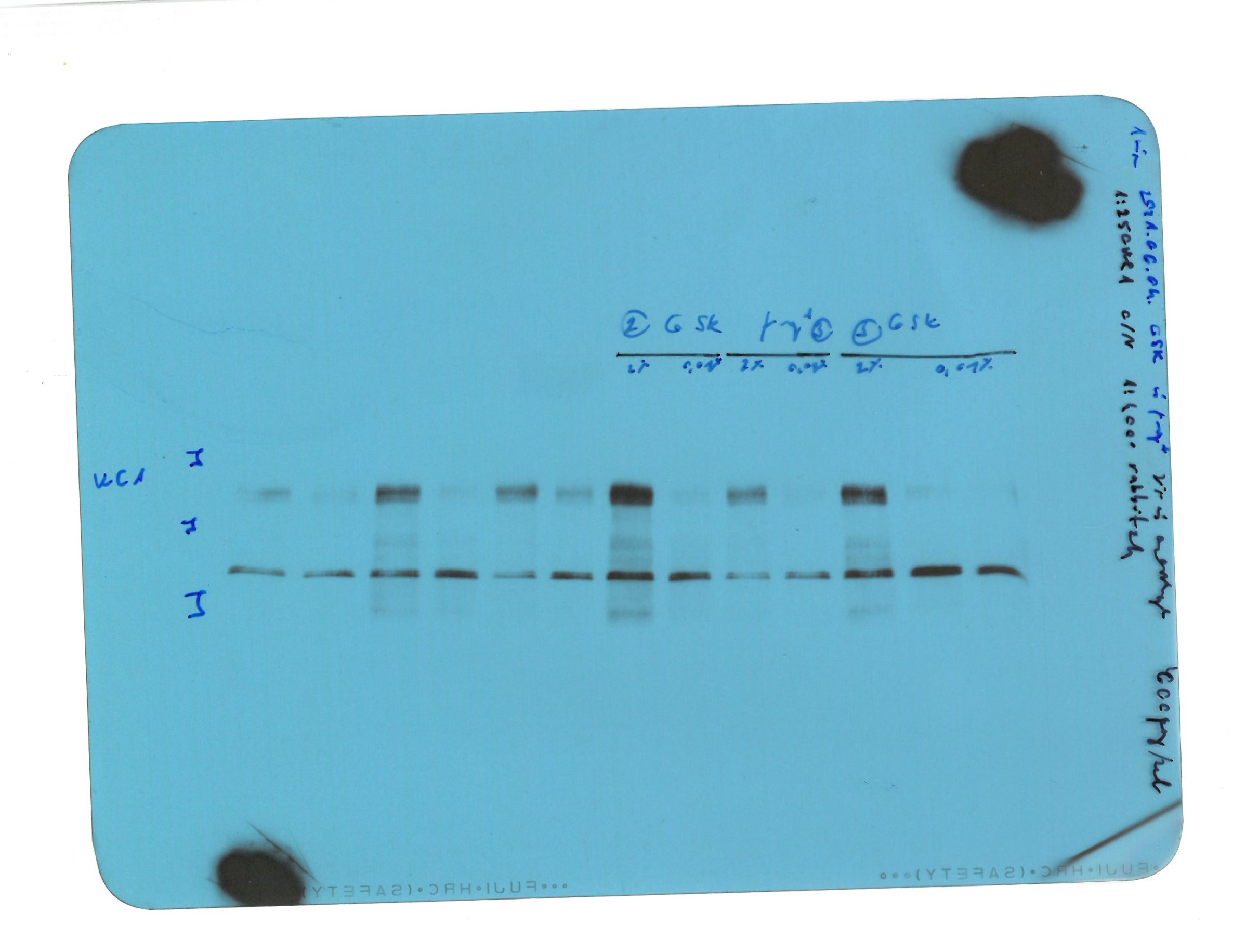

Supplement: Figure 3—source data 4. — (a) Western blots were used to detect expression of FRQ in the indicated samples for Figure 3E. (b) Figure with the area highlighted was used to develop the Figure 3E for FRQ. (c) Western blots were used to detect expression of WC-1 in the indicated samples for Figure 3E. (d) Figure with the area highlighted was used to develop the Figure 3E for WC-1. (e) Western blots were used to detect expression of WC-2 in the indicated samples for Figure 3E. (f) Figure with the area highlighted was used to develop the Figure 3E for WC-2. (g) Ponceau S staining was used to detect loading control of the indicated samples for Figure 3E. (h) Figure with the area highlighted was used as LC of Figure 3E. [file elife-79765-fig3-data4.zip › 79765Figure3E/Figure_3-source_data_4c.jpg]

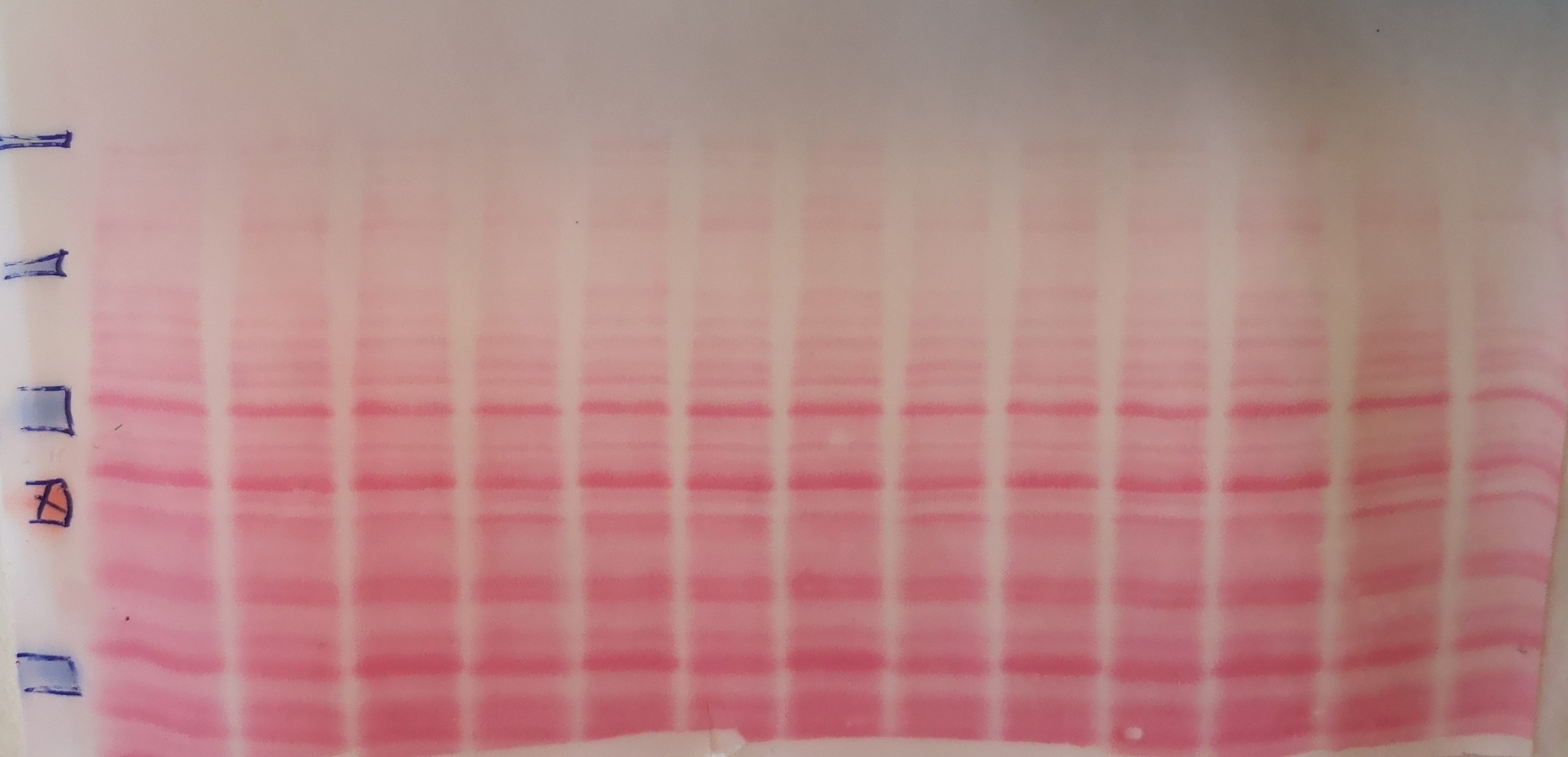

Supplement: Figure 3—source data 4. — (a) Western blots were used to detect expression of FRQ in the indicated samples for Figure 3E. (b) Figure with the area highlighted was used to develop the Figure 3E for FRQ. (c) Western blots were used to detect expression of WC-1 in the indicated samples for Figure 3E. (d) Figure with the area highlighted was used to develop the Figure 3E for WC-1. (e) Western blots were used to detect expression of WC-2 in the indicated samples for Figure 3E. (f) Figure with the area highlighted was used to develop the Figure 3E for WC-2. (g) Ponceau S staining was used to detect loading control of the indicated samples for Figure 3E. (h) Figure with the area highlighted was used as LC of Figure 3E. [file elife-79765-fig3-data4.zip › 79765Figure3E/Figure_3-source_data_4g.jpg]

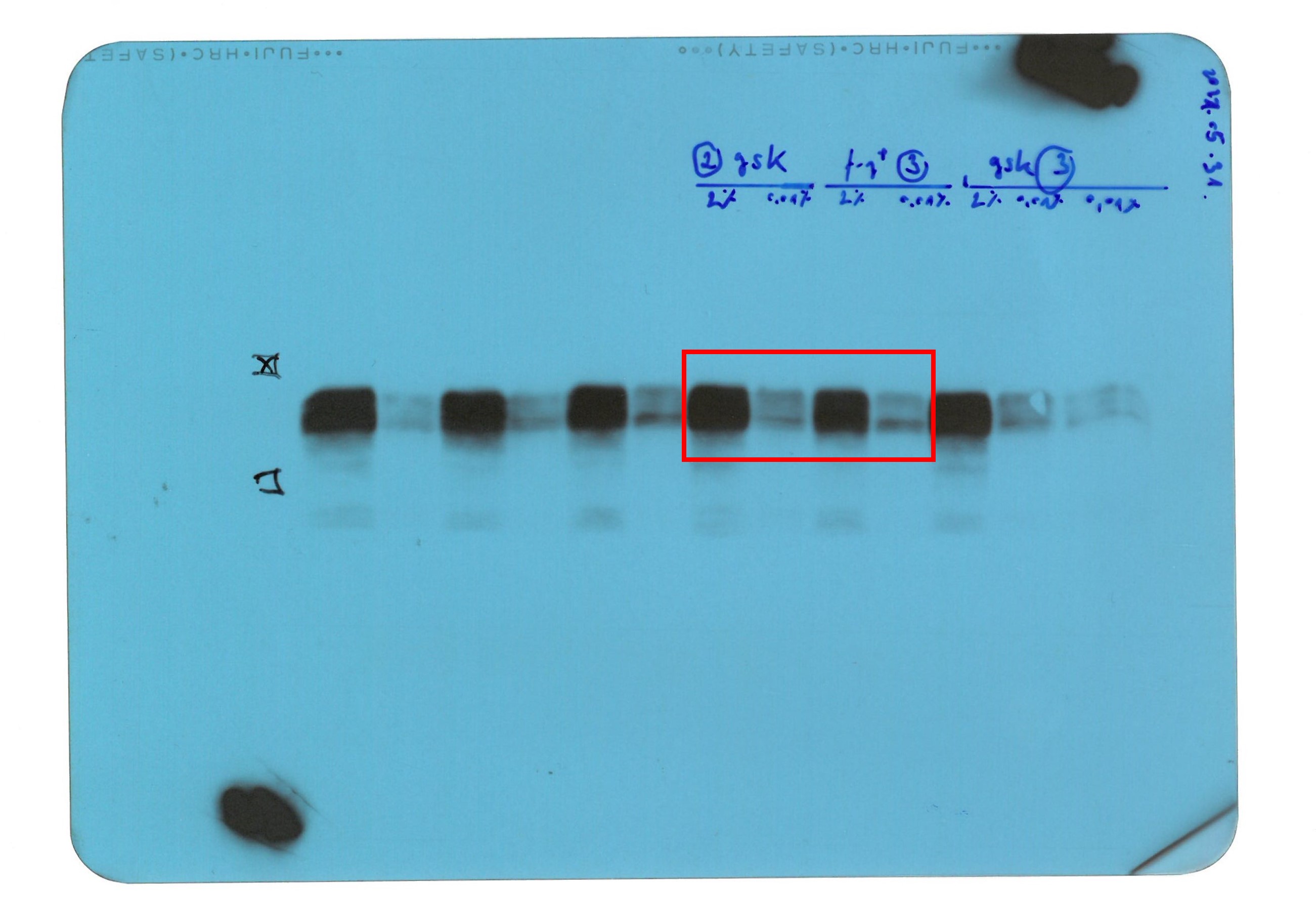

Supplement: Figure 3—source data 4. — (a) Western blots were used to detect expression of FRQ in the indicated samples for Figure 3E. (b) Figure with the area highlighted was used to develop the Figure 3E for FRQ. (c) Western blots were used to detect expression of WC-1 in the indicated samples for Figure 3E. (d) Figure with the area highlighted was used to develop the Figure 3E for WC-1. (e) Western blots were used to detect expression of WC-2 in the indicated samples for Figure 3E. (f) Figure with the area highlighted was used to develop the Figure 3E for WC-2. (g) Ponceau S staining was used to detect loading control of the indicated samples for Figure 3E. (h) Figure with the area highlighted was used as LC of Figure 3E. [file elife-79765-fig3-data4.zip › 79765Figure3E/Figure_3-source_data_4f.jpg]

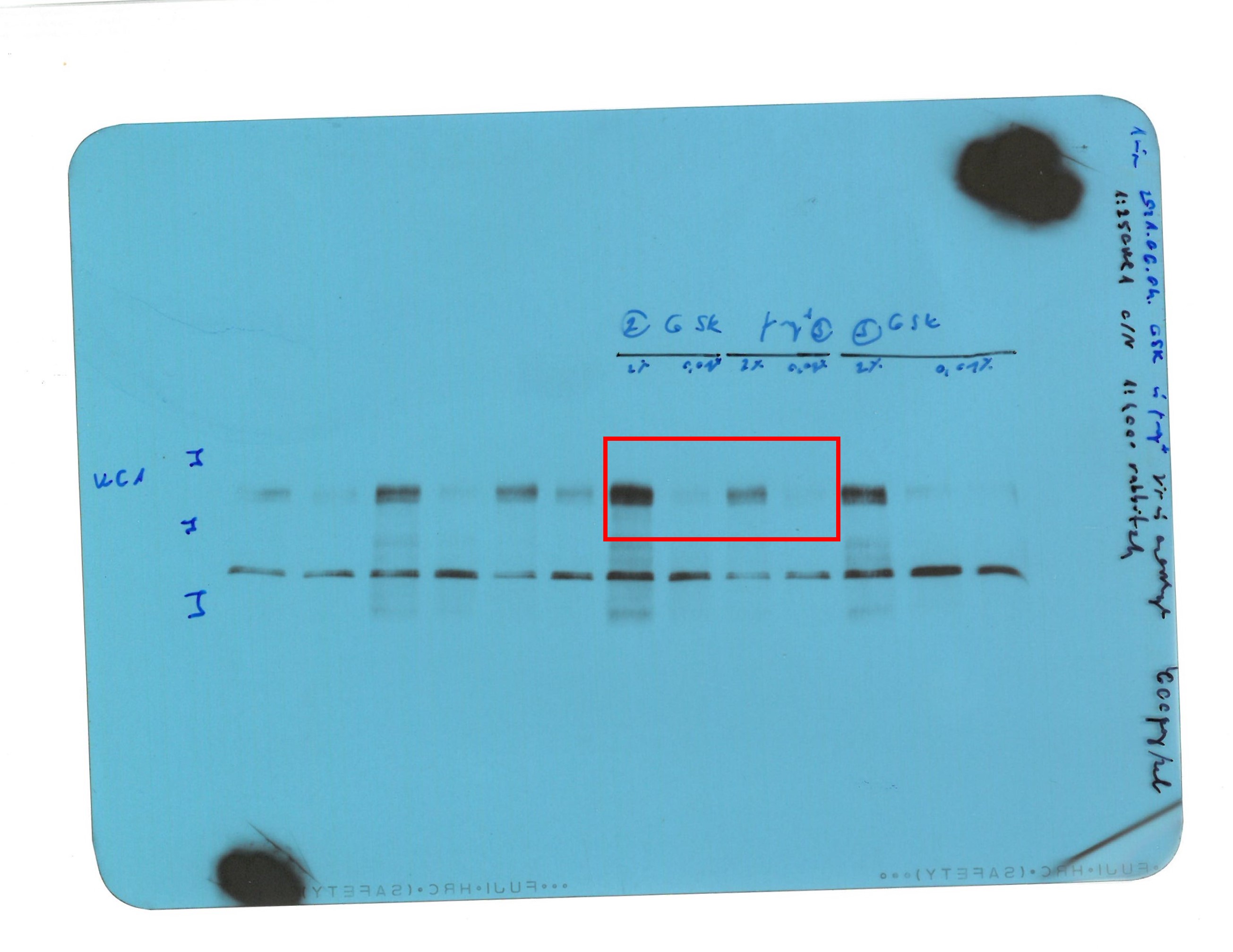

Supplement: Figure 3—source data 4. — (a) Western blots were used to detect expression of FRQ in the indicated samples for Figure 3E. (b) Figure with the area highlighted was used to develop the Figure 3E for FRQ. (c) Western blots were used to detect expression of WC-1 in the indicated samples for Figure 3E. (d) Figure with the area highlighted was used to develop the Figure 3E for WC-1. (e) Western blots were used to detect expression of WC-2 in the indicated samples for Figure 3E. (f) Figure with the area highlighted was used to develop the Figure 3E for WC-2. (g) Ponceau S staining was used to detect loading control of the indicated samples for Figure 3E. (h) Figure with the area highlighted was used as LC of Figure 3E. [file elife-79765-fig3-data4.zip › 79765Figure3E/Figure_3-source_data_4d.jpg]

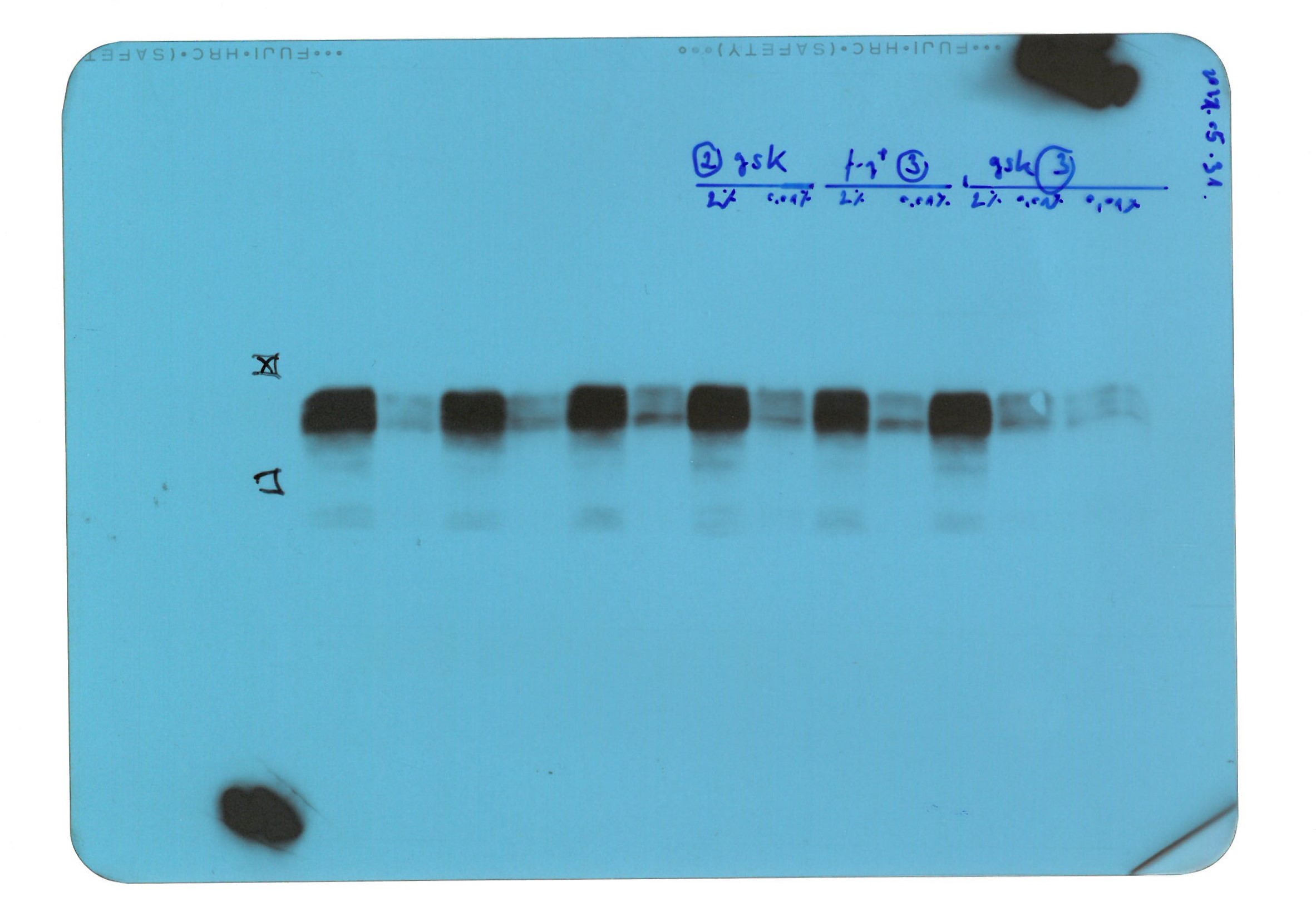

Supplement: Figure 3—source data 4. — (a) Western blots were used to detect expression of FRQ in the indicated samples for Figure 3E. (b) Figure with the area highlighted was used to develop the Figure 3E for FRQ. (c) Western blots were used to detect expression of WC-1 in the indicated samples for Figure 3E. (d) Figure with the area highlighted was used to develop the Figure 3E for WC-1. (e) Western blots were used to detect expression of WC-2 in the indicated samples for Figure 3E. (f) Figure with the area highlighted was used to develop the Figure 3E for WC-2. (g) Ponceau S staining was used to detect loading control of the indicated samples for Figure 3E. (h) Figure with the area highlighted was used as LC of Figure 3E. [file elife-79765-fig3-data4.zip › 79765Figure3E/Figure_3-source_data_4e.jpg]

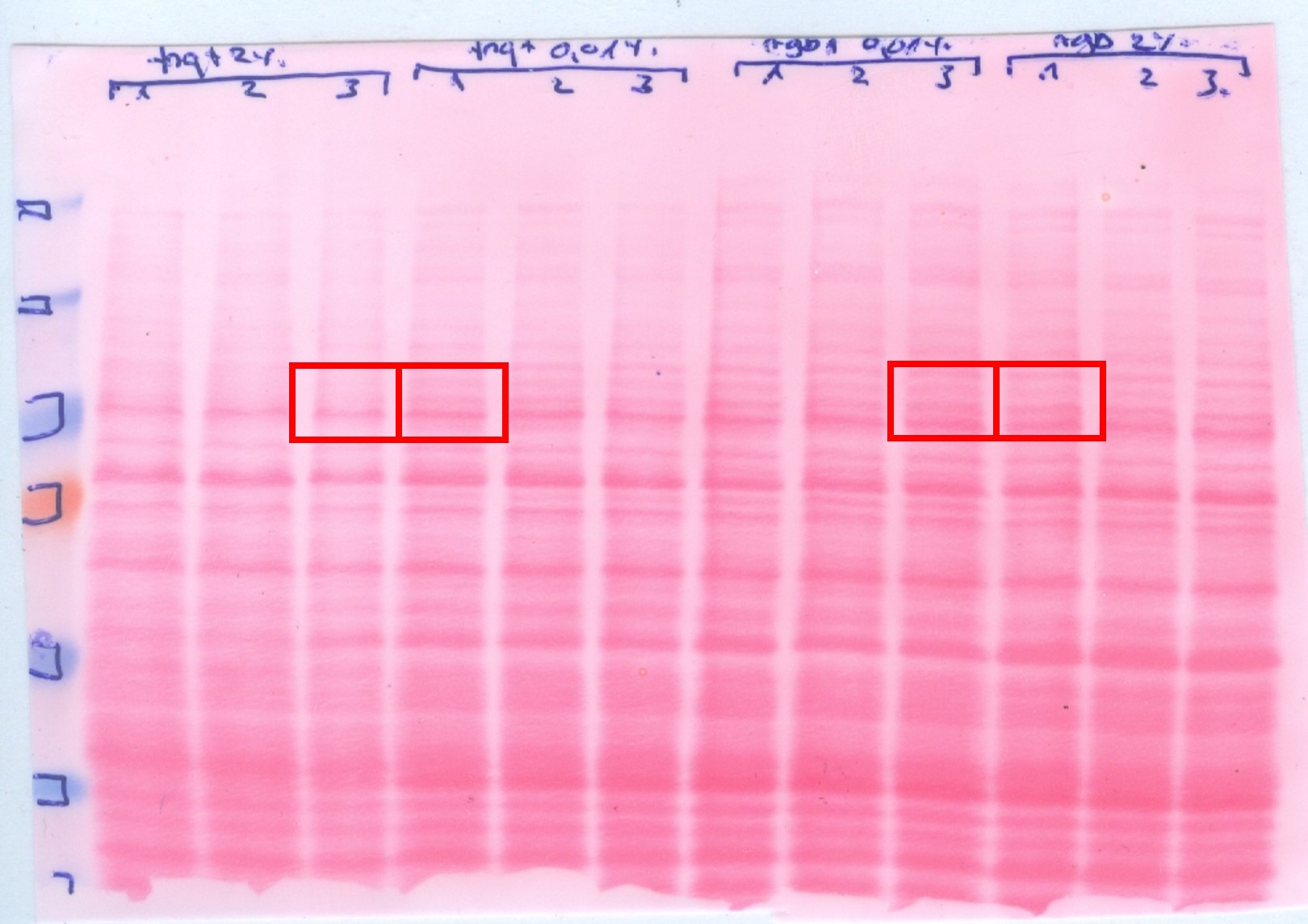

Supplement: Figure 3—source data 5. — (a) Western blots were used to detect expression of FRQ in the indicated samples for Figure 3G. (b) Figure with the area highlighted was used to develop the Figure 3G for FRQ. (c) Western blots were used to detect expression of WC-1 in the indicated samples for Figure 3G. (d) Figure with the area highlighted was used to develop the Figure 3G for WC-1. (e) Western blots were used to detect expression of WC-2 in the indicated samples for Figure 3G. (f) Figure with the area highlighted was used to develop the Figure 3G for WC-2. (g) Ponceau S staining was used to detect loading control of the indicated samples for Figure 3G for FRQ. (h) Figure with the area highlighted was used as LCfor FRQ in Figure 3G. (i) Figure with the area highlighted was used as LC for WC-1 develop the Figure 3G for LC for WC-1. (j) Figure with the area highlighted was used to develop the Figure 3G for LC for WC-2. [file elife-79765-fig3-data5.zip › 79765Figure3G/Figure_3-source_data_5j.jpg]

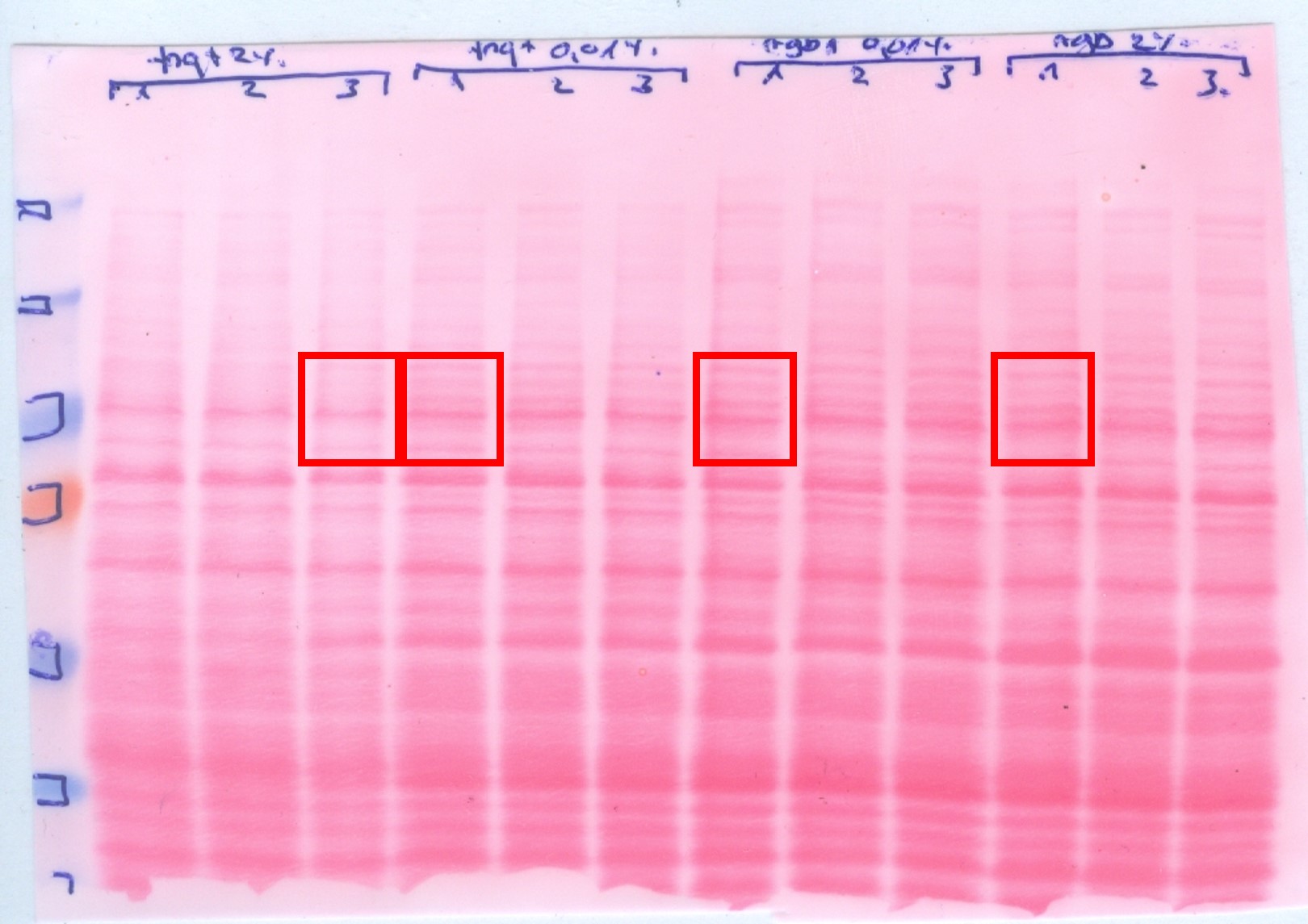

Supplement: Figure 3—source data 5. — (a) Western blots were used to detect expression of FRQ in the indicated samples for Figure 3G. (b) Figure with the area highlighted was used to develop the Figure 3G for FRQ. (c) Western blots were used to detect expression of WC-1 in the indicated samples for Figure 3G. (d) Figure with the area highlighted was used to develop the Figure 3G for WC-1. (e) Western blots were used to detect expression of WC-2 in the indicated samples for Figure 3G. (f) Figure with the area highlighted was used to develop the Figure 3G for WC-2. (g) Ponceau S staining was used to detect loading control of the indicated samples for Figure 3G for FRQ. (h) Figure with the area highlighted was used as LCfor FRQ in Figure 3G. (i) Figure with the area highlighted was used as LC for WC-1 develop the Figure 3G for LC for WC-1. (j) Figure with the area highlighted was used to develop the Figure 3G for LC for WC-2. [file elife-79765-fig3-data5.zip › 79765Figure3G/Figure_3-source_data_5i.jpg]

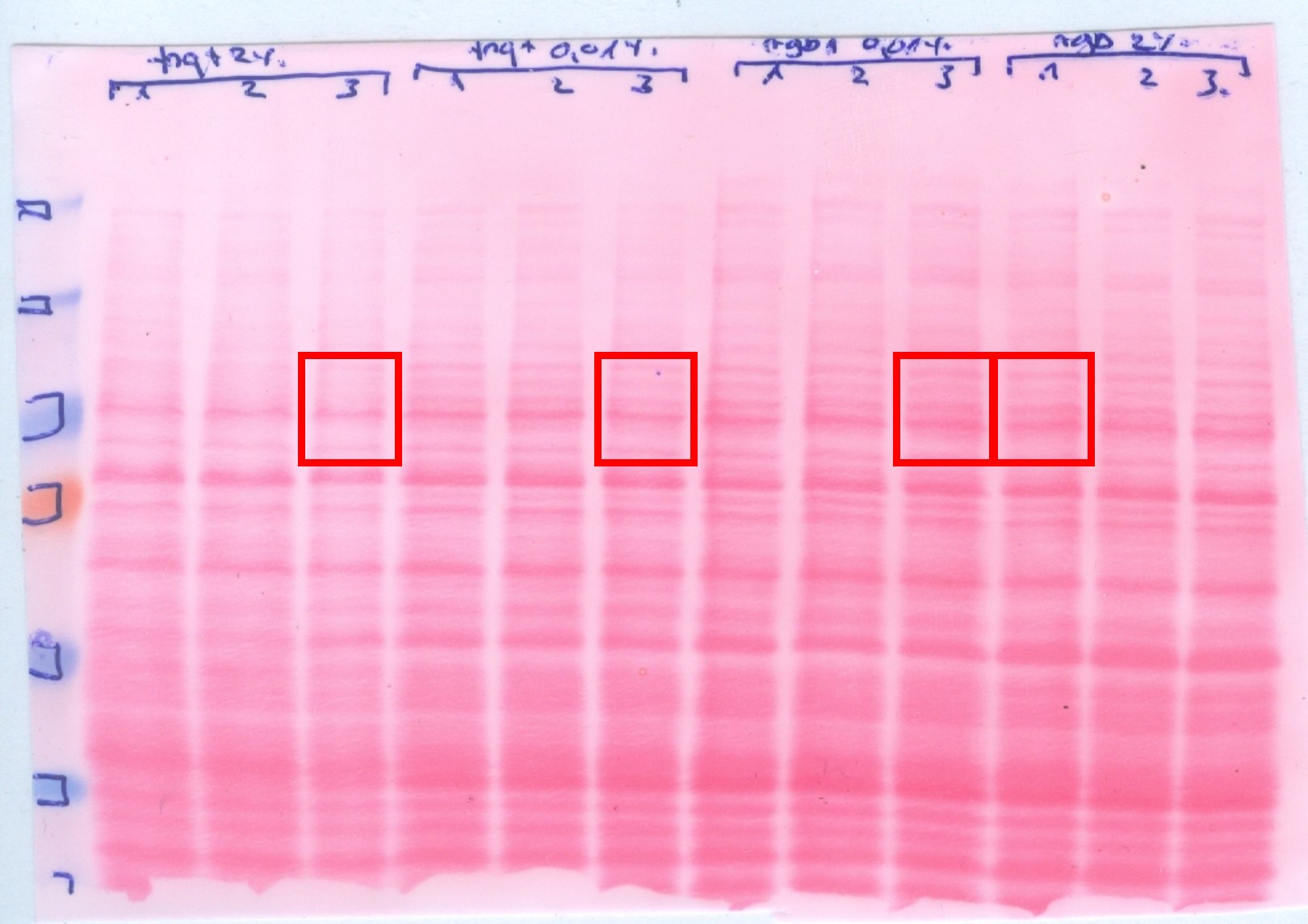

Supplement: Figure 3—source data 5. — (a) Western blots were used to detect expression of FRQ in the indicated samples for Figure 3G. (b) Figure with the area highlighted was used to develop the Figure 3G for FRQ. (c) Western blots were used to detect expression of WC-1 in the indicated samples for Figure 3G. (d) Figure with the area highlighted was used to develop the Figure 3G for WC-1. (e) Western blots were used to detect expression of WC-2 in the indicated samples for Figure 3G. (f) Figure with the area highlighted was used to develop the Figure 3G for WC-2. (g) Ponceau S staining was used to detect loading control of the indicated samples for Figure 3G for FRQ. (h) Figure with the area highlighted was used as LCfor FRQ in Figure 3G. (i) Figure with the area highlighted was used as LC for WC-1 develop the Figure 3G for LC for WC-1. (j) Figure with the area highlighted was used to develop the Figure 3G for LC for WC-2. [file elife-79765-fig3-data5.zip › 79765Figure3G/Figure_3-source_data_5h.jpg]

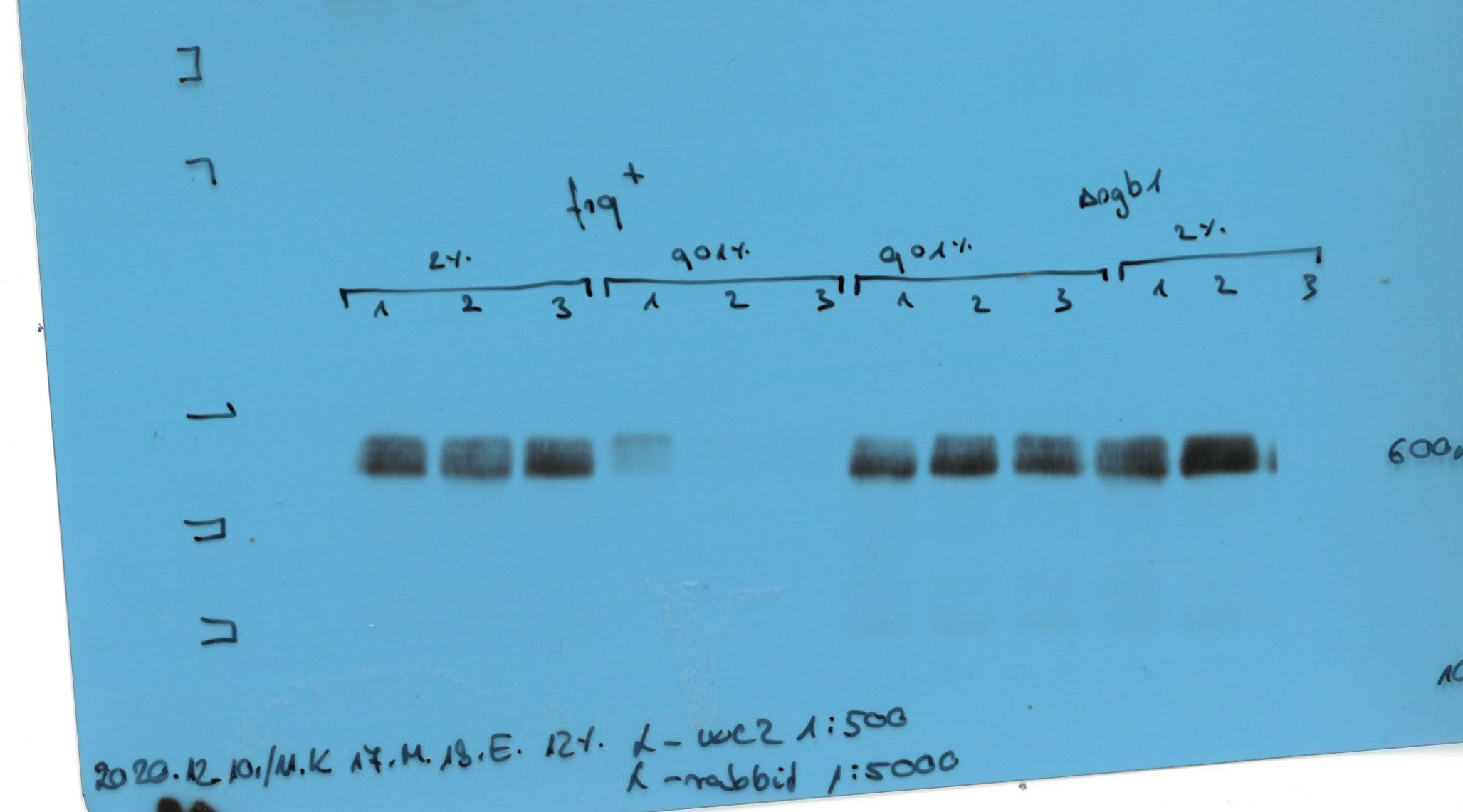

Supplement: Figure 3—source data 5. — (a) Western blots were used to detect expression of FRQ in the indicated samples for Figure 3G. (b) Figure with the area highlighted was used to develop the Figure 3G for FRQ. (c) Western blots were used to detect expression of WC-1 in the indicated samples for Figure 3G. (d) Figure with the area highlighted was used to develop the Figure 3G for WC-1. (e) Western blots were used to detect expression of WC-2 in the indicated samples for Figure 3G. (f) Figure with the area highlighted was used to develop the Figure 3G for WC-2. (g) Ponceau S staining was used to detect loading control of the indicated samples for Figure 3G for FRQ. (h) Figure with the area highlighted was used as LCfor FRQ in Figure 3G. (i) Figure with the area highlighted was used as LC for WC-1 develop the Figure 3G for LC for WC-1. (j) Figure with the area highlighted was used to develop the Figure 3G for LC for WC-2. [file elife-79765-fig3-data5.zip › 79765Figure3G/Figure_3-source_data_5e.jpg]

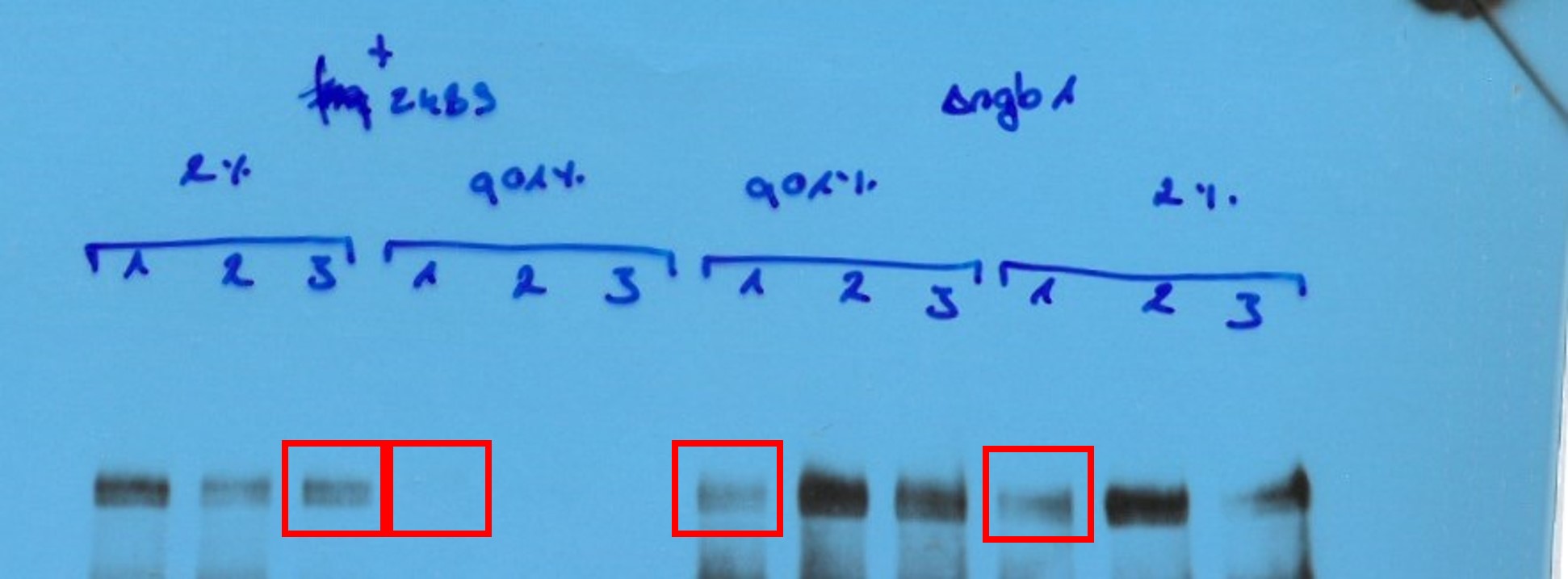

Supplement: Figure 3—source data 5. — (a) Western blots were used to detect expression of FRQ in the indicated samples for Figure 3G. (b) Figure with the area highlighted was used to develop the Figure 3G for FRQ. (c) Western blots were used to detect expression of WC-1 in the indicated samples for Figure 3G. (d) Figure with the area highlighted was used to develop the Figure 3G for WC-1. (e) Western blots were used to detect expression of WC-2 in the indicated samples for Figure 3G. (f) Figure with the area highlighted was used to develop the Figure 3G for WC-2. (g) Ponceau S staining was used to detect loading control of the indicated samples for Figure 3G for FRQ. (h) Figure with the area highlighted was used as LCfor FRQ in Figure 3G. (i) Figure with the area highlighted was used as LC for WC-1 develop the Figure 3G for LC for WC-1. (j) Figure with the area highlighted was used to develop the Figure 3G for LC for WC-2. [file elife-79765-fig3-data5.zip › 79765Figure3G/Figure_3-source_data_5d.jpg]

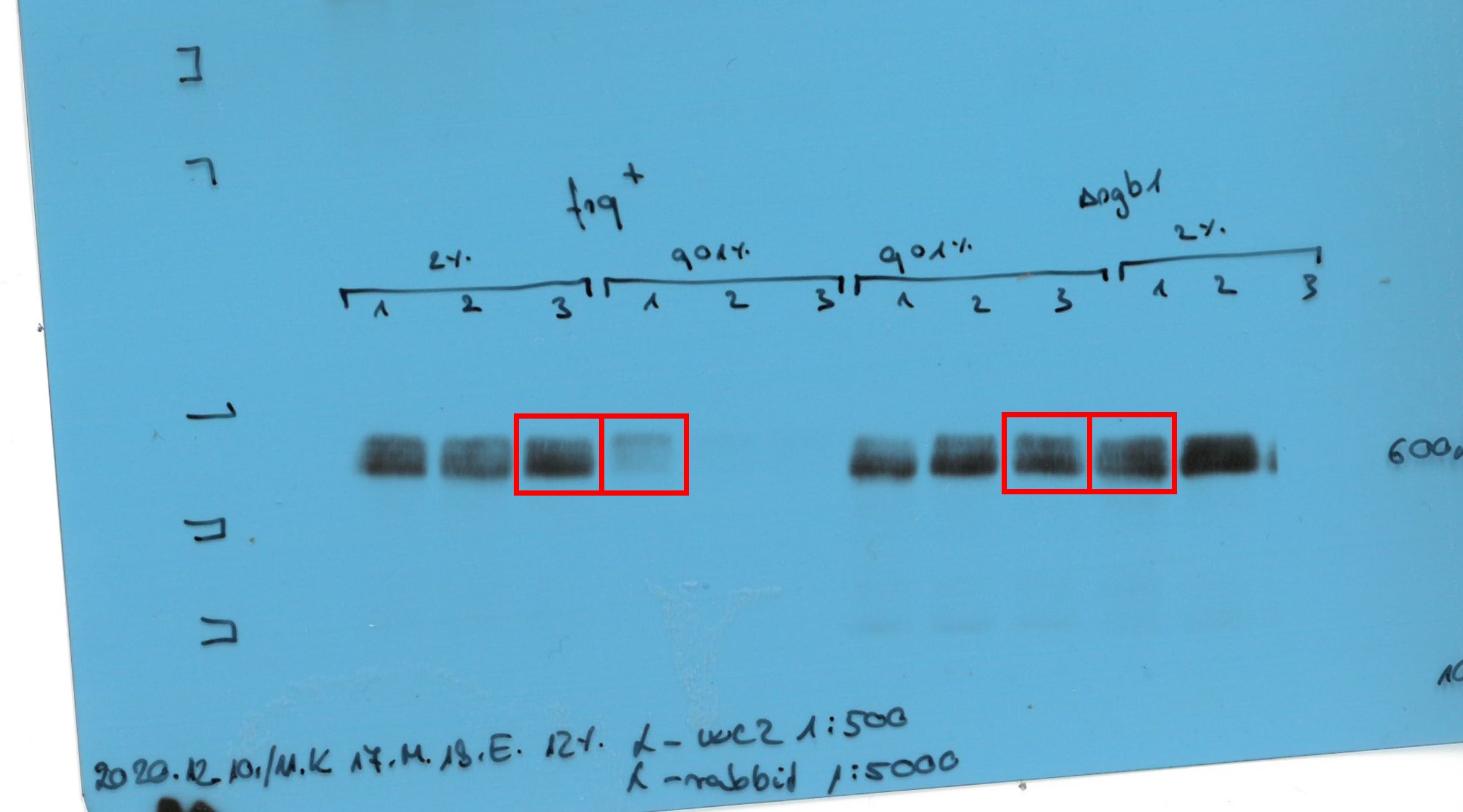

Supplement: Figure 3—source data 5. — (a) Western blots were used to detect expression of FRQ in the indicated samples for Figure 3G. (b) Figure with the area highlighted was used to develop the Figure 3G for FRQ. (c) Western blots were used to detect expression of WC-1 in the indicated samples for Figure 3G. (d) Figure with the area highlighted was used to develop the Figure 3G for WC-1. (e) Western blots were used to detect expression of WC-2 in the indicated samples for Figure 3G. (f) Figure with the area highlighted was used to develop the Figure 3G for WC-2. (g) Ponceau S staining was used to detect loading control of the indicated samples for Figure 3G for FRQ. (h) Figure with the area highlighted was used as LCfor FRQ in Figure 3G. (i) Figure with the area highlighted was used as LC for WC-1 develop the Figure 3G for LC for WC-1. (j) Figure with the area highlighted was used to develop the Figure 3G for LC for WC-2. [file elife-79765-fig3-data5.zip › 79765Figure3G/Figure_3-source_data_5f.jpg]

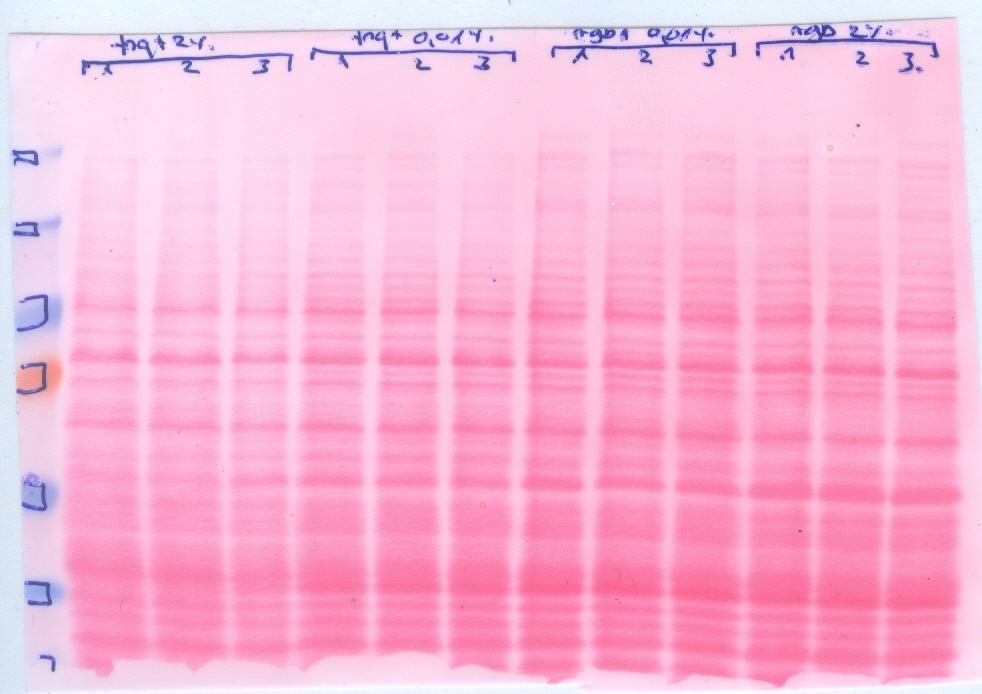

Supplement: Figure 3—source data 5. — (a) Western blots were used to detect expression of FRQ in the indicated samples for Figure 3G. (b) Figure with the area highlighted was used to develop the Figure 3G for FRQ. (c) Western blots were used to detect expression of WC-1 in the indicated samples for Figure 3G. (d) Figure with the area highlighted was used to develop the Figure 3G for WC-1. (e) Western blots were used to detect expression of WC-2 in the indicated samples for Figure 3G. (f) Figure with the area highlighted was used to develop the Figure 3G for WC-2. (g) Ponceau S staining was used to detect loading control of the indicated samples for Figure 3G for FRQ. (h) Figure with the area highlighted was used as LCfor FRQ in Figure 3G. (i) Figure with the area highlighted was used as LC for WC-1 develop the Figure 3G for LC for WC-1. (j) Figure with the area highlighted was used to develop the Figure 3G for LC for WC-2. [file elife-79765-fig3-data5.zip › 79765Figure3G/Figure_3-source_data_5g.jpg]

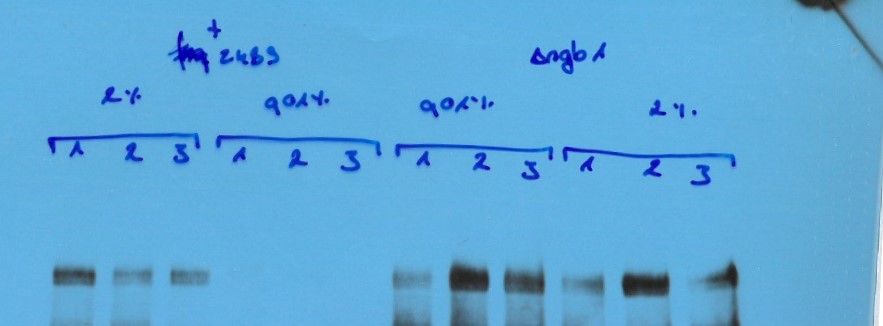

Supplement: Figure 3—source data 5. — (a) Western blots were used to detect expression of FRQ in the indicated samples for Figure 3G. (b) Figure with the area highlighted was used to develop the Figure 3G for FRQ. (c) Western blots were used to detect expression of WC-1 in the indicated samples for Figure 3G. (d) Figure with the area highlighted was used to develop the Figure 3G for WC-1. (e) Western blots were used to detect expression of WC-2 in the indicated samples for Figure 3G. (f) Figure with the area highlighted was used to develop the Figure 3G for WC-2. (g) Ponceau S staining was used to detect loading control of the indicated samples for Figure 3G for FRQ. (h) Figure with the area highlighted was used as LCfor FRQ in Figure 3G. (i) Figure with the area highlighted was used as LC for WC-1 develop the Figure 3G for LC for WC-1. (j) Figure with the area highlighted was used to develop the Figure 3G for LC for WC-2. [file elife-79765-fig3-data5.zip › 79765Figure3G/Figure_3-source_data_5c.jpg]

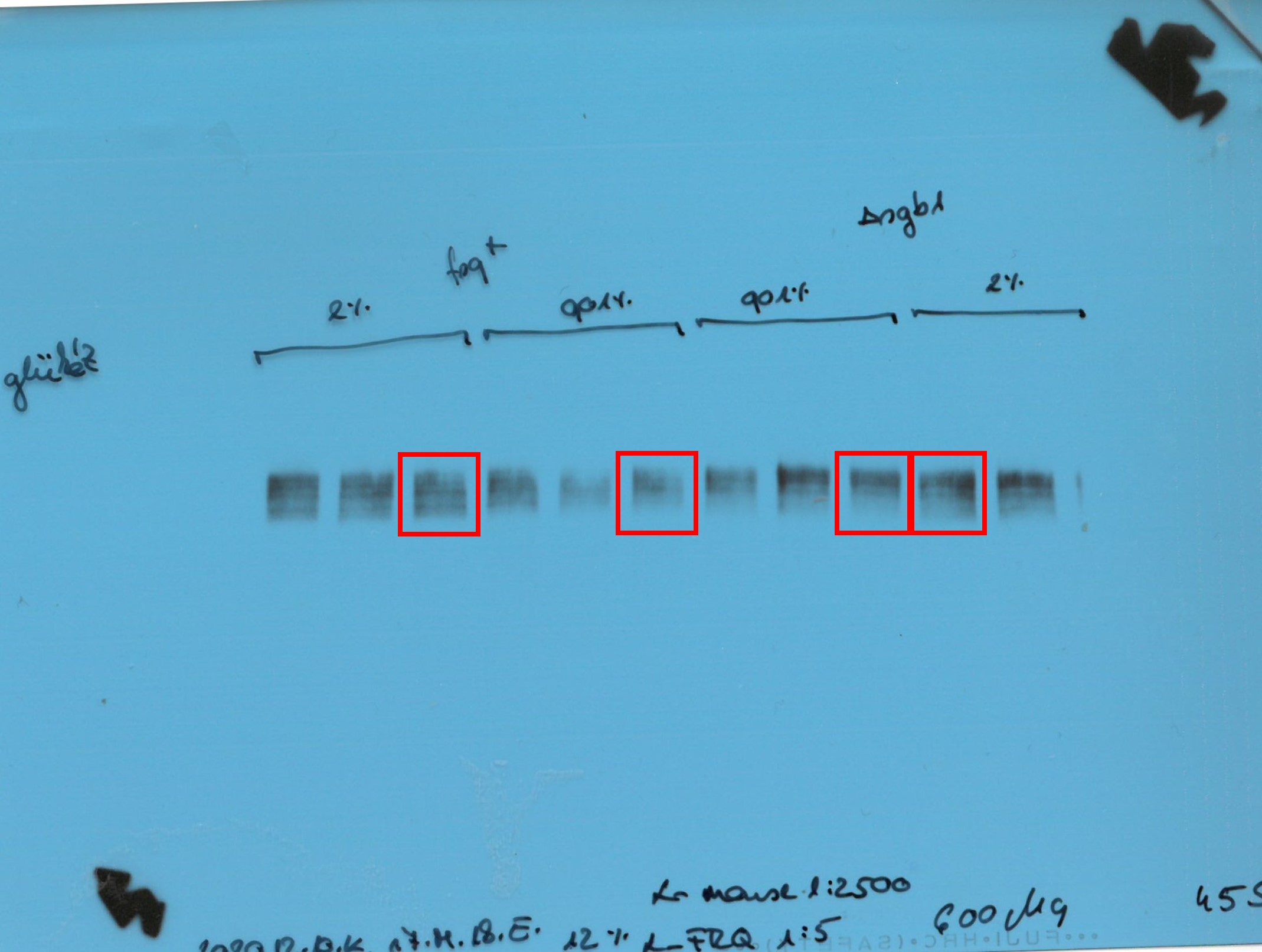

Supplement: Figure 3—source data 5. — (a) Western blots were used to detect expression of FRQ in the indicated samples for Figure 3G. (b) Figure with the area highlighted was used to develop the Figure 3G for FRQ. (c) Western blots were used to detect expression of WC-1 in the indicated samples for Figure 3G. (d) Figure with the area highlighted was used to develop the Figure 3G for WC-1. (e) Western blots were used to detect expression of WC-2 in the indicated samples for Figure 3G. (f) Figure with the area highlighted was used to develop the Figure 3G for WC-2. (g) Ponceau S staining was used to detect loading control of the indicated samples for Figure 3G for FRQ. (h) Figure with the area highlighted was used as LCfor FRQ in Figure 3G. (i) Figure with the area highlighted was used as LC for WC-1 develop the Figure 3G for LC for WC-1. (j) Figure with the area highlighted was used to develop the Figure 3G for LC for WC-2. [file elife-79765-fig3-data5.zip › 79765Figure3G/Figure_3-source_data_5b.jpg]

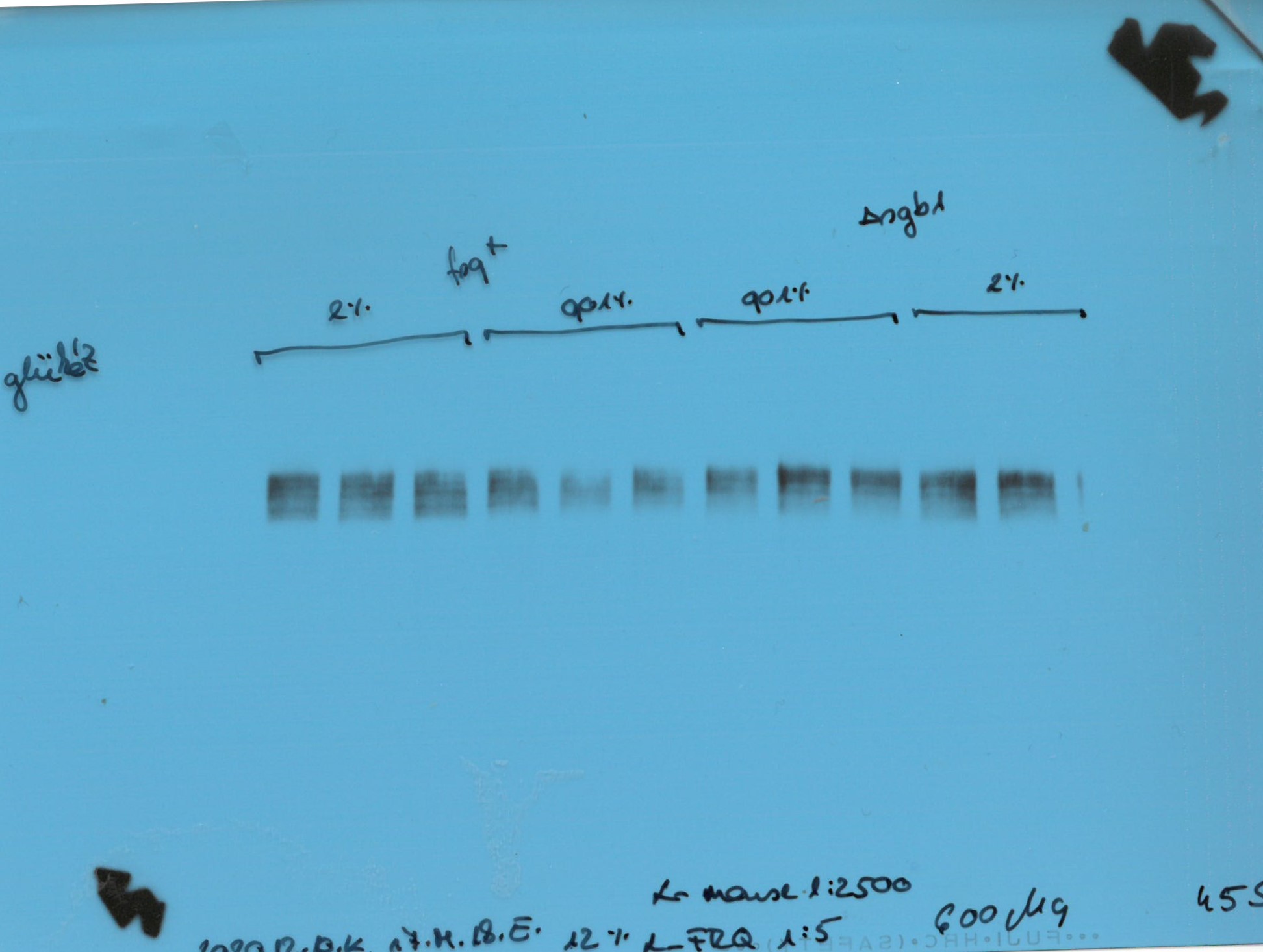

Supplement: Figure 3—source data 5. — (a) Western blots were used to detect expression of FRQ in the indicated samples for Figure 3G. (b) Figure with the area highlighted was used to develop the Figure 3G for FRQ. (c) Western blots were used to detect expression of WC-1 in the indicated samples for Figure 3G. (d) Figure with the area highlighted was used to develop the Figure 3G for WC-1. (e) Western blots were used to detect expression of WC-2 in the indicated samples for Figure 3G. (f) Figure with the area highlighted was used to develop the Figure 3G for WC-2. (g) Ponceau S staining was used to detect loading control of the indicated samples for Figure 3G for FRQ. (h) Figure with the area highlighted was used as LCfor FRQ in Figure 3G. (i) Figure with the area highlighted was used as LC for WC-1 develop the Figure 3G for LC for WC-1. (j) Figure with the area highlighted was used to develop the Figure 3G for LC for WC-2. [file elife-79765-fig3-data5.zip › 79765Figure3G/Figure_3-source_data_5a.jpg]
