# Supplementary material for: Adaptation to glucose starvation is associated with molecular reorganization of the circadian clock in Neurospora crassa
Source: eLife. 2023 Jan 10;12:e79765. doi: 10.7554/eLife.79765 (PMC9831608; doi:10.7554/eLife.79765)
Supplement: Figure 1—source data 4. — Experimental procedures were performed as described in Figure 1C. Ct values of the indicated genes were determined by qPCR. (n=3, ± SEM) In the last row, ratio of the expression levels (wt 0.01%/2%) are shown based on the RNAseq dataset. [file elife-79765-fig1-data4.docx]

**Figure 4 – Source data 1**

*Actin levels are decreased in glucose starvation.*

Experimental procedures were performed as described in Figure 1C. C_t_ values of the indicated genes were determined by qPCR. (n=3, ±SEM) In the last row ratio of the expression levels (*wt* 0.01%/2%) are shown based on the RNAseq dataset.

|  | ***act*** | ***gna-3*** | ***tfc-1*** | ***sarA*** |
| --- | --- | --- | --- | --- |
| **C_t_ (*wt* 2%)** | 18.34 ±0.047 | 24.04 ±0.046 | 26.94 ±0.027 | 20.61 ±0.053 |
| **C_t_ (*wt* 0.01%)** | 20.81 ±0.021 | 24.43 ±0.082 | 27.51 ±0.372 | 21.93 ±0.191 |
| **RNAseq (0.01%/2%)** | 0.73 | 1.01 | 1.07 | 0.82 |
