# Supplementary material for: Adaptation to glucose starvation is associated with molecular reorganization of the circadian clock in Neurospora crassa
Source: eLife. 2023 Jan 10;12:e79765. doi: 10.7554/eLife.79765 (PMC9831608; doi:10.7554/eLife.79765)
Supplement: Figure 4—source data 1. [file elife-79765-fig4-data1.docx]

**Figure 4 – Source data 1**

*Genes, that changed in a strain-specific manner in response to glucose starvation and are direct targets of the WCC.*

| *NCU00011* | *NCU02138* | *NCU04480* | *NCU05948* | *NCU07067* | *NCU09235* |
| --- | --- | --- | --- | --- | --- |
| *NCU00069* | *NCU02333* | *NCU04639* | *NCU05949* | *NCU07159* | *NCU09335* |
| *NCU00399* | *NCU02369* | *NCU04959* | *NCU05964* | *NCU07448* | *NCU09350* |
| *NCU00552* | *NCU02609* | *NCU05126* | *NCU06010* | *NCU07569* | *NCU09615* |
| *NCU00554* | *NCU02622* | *NCU05131* | *NCU06043* | *NCU07600* | *NCU09678* |
| *NCU00575* | *NCU02712* | *NCU05133* | *NCU06123* | *NCU07786* | *NCU09685* |
| *NCU00582* | *NCU02713* | *NCU05143* | *NCU06436* | *NCU08167* | *NCU09738* |
| *NCU00584* | *NCU02801* | *NCU05316* | *NCU06597* | *NCU08283* | *NCU09872* |
| *NCU00585* | *NCU03388* | *NCU05338* | *NCU06651* | *NCU08319* | *NCU09904* |
| *NCU00746* | *NCU03448* | *NCU05373* | *NCU06660* | *NCU08491* | *NCU09906* |
| *NCU00859* | *NCU03466* | *NCU05448* | *NCU06724* | *NCU08533* | *NCU10387* |
| *NCU01385* | *NCU03699* | *NCU05721* | *NCU06785* | *NCU08699* | *NCU10457* |
| *NCU01417* | *NCU04179* | *NCU05732* | *NCU06795* | *NCU08791* | *NCU11201* |
| *NCU01653* | *NCU04260* | *NCU05830* | *NCU07008* | *NCU08820* | *NCU11290* |
| *NCU01862* | *NCU04315* | *NCU05897* | *NCU07022* | *NCU09068* | *NCU11395* |
