# Supplementary material for: Adaptation to glucose starvation is associated with molecular reorganization of the circadian clock in Neurospora crassa
Source: eLife. 2023 Jan 10;12:e79765. doi: 10.7554/eLife.79765 (PMC9831608; doi:10.7554/eLife.79765)
Supplement: Figure 4—source data 3. — Significantly enriched functions are shown. GO enrichment analysis was performed on data obtained by the analysis shown in Figure 4—figure supplement 1, that is on genes showing exclusive or significantly higher change in their expression rate in one of the strains. (FDR: false discovery rate). [file elife-79765-fig4-data3.docx]

**Figure 4 – Source data 3**

*Gene Ontology (GO) enrichment analysis of genes showing strain-specific response to starvation.* Significantly enriched functions are shown. Go enrichment analysis was performed on data obtained by the analysis shown in Figure 4 – Figure supplement 1, *i.e.* on genes showing exclusive or significantly higher change in their expression rate in one of the strains. (FDR: false discovery rate)

| **Genes showing significant enrichment among strain-specifically upregulated ones in *wt*** | | | | | | | | |
| --- | --- | --- | --- | --- | --- | --- | --- | --- |
|  | ***Neurospora crassa* (REF)** | **Upload** | | | |  | | |
|  | **#** | **#** | **Expected** | | **Fold Enrichment** | **+/-** | **Raw p value** | **FDR** |
| Unclassified | 4789 | 295 | 200.24 | | 1.47 | + | 1.41E-20 | 3.09E-17 |
| **Genes showing significant enrichment among strain-specifically upregulated ones in *∆wc-1*** | | | | | | | | |
|  | ***Neurospora crassa* (REF)** | **Upload** | | | |  | | |
|  | **#** | **#** | **Expected** | | **Fold Enrichment** | **+/-** | **Raw P value** | **FDR** |
| Unclassified | 4789 | 332 | 256.19 | | 1.30 | + | 1.03E-10 | 5.03E-0 |
|  | | | | | | | | |
| Alpha-amino acid catabolic process | 36 | 10 | 1.93 | | 5.19 | + | 8.14E-05 | 6.75E-03 |
| Cellular amino acid catabolic process | 48 | 12 | 2.57 | | 4.67 | + | 3.88E-05 | 3.55E-03 |
| L-phenylalanine catabolic process | 4 | 4 | 0.21 | | 18.69 | + | 3.91E-04 | 2.45E-02 |
|  | | | | | | | | |
| Organic acid catabolic process | 83 | 14 | 4.44 | | 3.15 | + | 3.64E-04 | 2.42E-02 |
| Carboxylic acid catabolic process | 79 | 14 | 4.23 | | 3.31 | + | 2.32E-04 | 1.67E-02 |
|  | | | | | | | | |
| Erythrose 4-phosphate/phosphoenolpyruvate family amino acid catabolic process | 4 | 4 | 0.21 | | 18.69 | + | 3.91E-04 | 2.49E-02 |
|  | | | | | | | | |
| Eisosome assembly | 5 | 4 | 0.27 | | 14.95 | + | 6.75E-04 | 3.90E-02 |
|  |  |  |  | |  |  |  |  |
| **Genes showing significant enrichment among strain-specifically downregulated ones in *wt*** | | | | | | | | |
|  | ***Neurospora crassa* (REF)** | **Upload** | | | |  | | |
|  | **#** | **#** | | **Expected** | **Fold Enrichment** | **+/-** | **Raw P value** | **FDR** |
| Small molecule biosynthetic process | 293 | 18 | | 6.64 | 2.71 | + | 1.85E-04 | 4.07E-02 |
| Small molecule metabolic process | 696 | 33 | | 15.76 | 2.09 | + | 6.91E-05 | 2.33E-02 |
|  | | | | | | | | |
| Cellular amino acid metabolic process | 209 | 18 | 4.73 | | 3.80 | + | 2.75E-06 | 1.73E-03 |
| Alpha-amino acid metabolic process | 134 | 13 | 3.03 | | 4.28 | + | 2.20E-05 | 1.07E-02 |
|  | | | | | | | | |
| Cellular amino acid biosynthetic process | 100 | 11 | | 2.26 | 4.86 | + | 3.32E-05 | 1.46E-02 |
| Alpha-amino acid biosynthetic process | 81 | 9 | | 1.83 | 4.91 | + | 1.62E-04 | 3.75E-02 |
|  | | | | | | | | |
| Organic acid metabolic process | 380 | 26 | 8.61 | | 3.02 | + | 1.01E-06 | 1.48E-03 |
| Oxoacid metabolic process | 369 | 25 | 8.36 | | 2.99 | + | 1.97E-06 | 1.73E-03 |
| Carboxylic acid metabolic process | 358 | 24 | 8.11 | | 2.96 | + | 3.80E-06 | 2.08E-03 |
|  | | | | | | | | |
| Organic acid biosynthetic process | 156 | 13 | | 3.53 | 3.68 | + | 9.45E-05 | 2.96E-02 |
|  |  |  | |  |  |  |  |  |
| **Genes showing significant enrichment among strain-specifically downregulated ones in *∆wc-1*** | | | | | | | | |
|  | ***Neurospora crassa* (REF)** | **Upload** | | | |  | | |
|  | **#** | **#** | | **Expected** | **Fold Enrichment** | **+/-** | **Raw P value** | **FDR** |
| Monocarboxylic acid biosynthetic process | 40 | 7 | | 0.70 | 9.99 | + | 1.40E-05 | 6.15E-02 |
| Fatty acid biosynthetic process | 27 | 6 | | 0.47 | 12.68 | + | 1.80E-05 | 2.64E-02 |
