# Supplementary material for: Adaptation to glucose starvation is associated with molecular reorganization of the circadian clock in Neurospora crassa
Source: eLife. 2023 Jan 10;12:e79765. doi: 10.7554/eLife.79765 (PMC9831608; doi:10.7554/eLife.79765)
Supplement: Figure 4—source data 4. — Genes were selected with the help of the KEGG Mapper tool. Numbering of genes in Figure 4—figure supplements 4–6 can be found in the last column. Genes, that are direct targets of WCC are marked with bold typesetting. [file elife-79765-fig4-data4.docx]

**Figure 4 – Source data 4**

*Genes of central carbon metabolism, amino acid biosynthesis and fatty acid metabolism, that showed strain-specific expression change to starvation.* Genes were selected with the help of the KEGG Mapper tool. Numbering of genes in Figure 4 – Figure supplement 4-6 can be found in the last column. Genes, that are direct targets of WCC are marked with bold typesetting.

|  |  |  | | |  | | ***wt*** | | ***∆wc-1*** | | | |  |
| --- | --- | --- | --- | --- | --- | --- | --- | --- | --- | --- | --- | --- | --- |
|  | **gene ID** | | | **protein** | | **fold change to starvation** | **adjusted p** | **fold change to starvation** | | **adjusted p** | **#** | |  |
| **Central carbon metabolism** |  | |  | | |  |  |  | |  | |  |  |
| ***wt* specific** | NCU04400 | | triose/dihydroxyacetone kinase / FAD-AMP lyase | | | 70.57 | 1.42312E-07 | 25.91 | | 0.001830917 | | 1a |  |
|  |  |  |  |  |  |  |  |  |  |  |  |  |  |
|  | NCU10107 | | ribose 5-phosphate isomerase | | | 45.11 | 7.76669E-06 | 5.62 | | ns | | 2a |  |
|  | NCU09873 | | acu-6; acetate utilization-6 | | | 39.04 | 1.60306E-59 | 11.60 | | 3.71073E-21 | | 3a |  |
|  | NCU02366 | | tca-3; tricarboxylic acid-3 | | | 2.98 | 5.33757E-24 | 1.39 | | 0.002131163 | | 4a |  |
|  | NCU06836 | | acu-5; acetate utilization-5 | | | 0.50 | 2.60354E-10 | ns | | ns | | 5a |  |
|  | NCU07608 | | ppm-3; pentose phosphate metabolism-3 | | | 0.44 | 4.48606E-11 | ns | | ns | | 6a |  |
|  | NCU02004 | | phosphoserine phosphatase | | | 0.43 | 2.44333E-15 | ns | | ns | | 7a |  |
|  | NCU02505 | | suc; succinate | | | 0.37 | 5.69527E-33 | ns | | ns | | 8a |  |
|  | NCU07281 | | gpi-1; glucose-6-phosphate isomerase | | | 0.35 | 3.47405E-10 | ns | | ns | | 9a |  |
|  | NCU09810 | | succinyl-CoA synthetase subunit alpha | | | 0.27 | 3.97874E-06 | ns | | ns | | 10a |  |
|  | NCU02274 | | for; formate | | | 0.17 | 1.43926E-26 | ns | | ns | | 11a |  |
|  | NCU01439 | | D-3-phosphoglycerate dehydrogenase 1 | | | 0.10 | 2.5872E-28 | 0.21 | | 2.98019E-09 | | 12a |  |
|  | NCU04797 | | fbp-1; fructose-bisphosphatase-1 | | | 6.05 | 1.09682E-45 | 14.35 | | 4.00722E-17 | | 1b |  |
| ***∆wc-1* specific** | NCU09732 | | acetyl-CoA acetyltransferase | | | 4.78 | 3.85062E-53 | 13.23 | | 1.80206E-83 | | 2b |  |
|  | NCU03761 | | L-serine/L-threonine ammonia-lyase | | | 3.04 | 2.03651E-21 | 9.41 | | 3.88952E-67 | | 3b |  |
|  | NCU05169 | | cat-4; catalase-4 | | | 3.87 | 1.87553E-39 | 8.73 | | 9.9475E-21 | | 4b |  |
|  | NCU08827 | | D-glycerate 3-kinase | | | 2.38 | 9.0648E-10 | 6.14 | | 1.59766E-26 | | 5b |  |
|  | **NCU08791** | | **cat-1; catalase-1** | | | **ns** | **Ns** | **5.63** | | **5.7231E-25** | | **6b** |  |
|  | NCU01866 | | lactonohydrolase | | | ns | ns | 5.19 | | 4.62773E-06 | | 7b |  |
|  | NCU01870 | | Eno3 protein | | | ns | ns | 3.43 | | 6.01917E-06 | | 8b |  |
|  | NCU02482 | | tca-2; tricarboxylic acid-2 | | | 0.59 | 8.36369E-07 | 2.89 | | 1.30995E-09 | | 9b |  |
|  | **NCU00575** | | **Glucokinase** | | | **ns** | **ns** | **2.78** | | **8.4829E-07** | | **10b** |  |
|  | NCU09266 | | methylmalonate-semialdehyde dehydrogenase | | | 1.30 | 0.002723935 | 2.75 | | 1.35703E-25 | | 11b |  |
|  | **NCU02712** | | **acetate kinase** | | | **ns** | **ns** | **2.48** | | **5.0573E-08** | | **12b** |  |
|  | NCU04230 | | acu-3; acetate utilization-3 | | | ns | ns | 2.43 | | 0.003354321 | | 13b |  |
|  | NCU09553 | | 3-hydroxybutyryl CoA dehydrogenase | | | ns | ns | 2.05 | | 2.24894E-08 | | 14b |  |

|  | | |  |  | | |  | | | | ***wt*** | | | | | ***∆wc-1*** | | | | |  |
| --- | --- | --- | --- | --- | --- | --- | --- | --- | --- | --- | --- | --- | --- | --- | --- | --- | --- | --- | --- | --- | --- |
|  |  |  | **gene ID** | | | **protein** | | | **fold change to starvation** | | **adjusted p** | | | **fold change to starvation** | | | **adjusted p** | | | **#** |  |
| **Amino acid biosynthesis** | |  | | |  | | |  | | | | |  |  | | | |  |  | |  |
| ***wt* specific** | | NCU10107 | | | ribose 5-phosphate isomerase | | | 45.11 | | | | | 7.76669E-06 | ns | | | | 0.000123509 | 1a | |  |
|  |  | NCU02366 | | | tricarboxylic acid-3 | | | 2.98 | | | | | 5.33757E-24 | 1.39 | | | | ns | 2a | |  |
|  |  | NCU05526 | | | lysine-5 | | | 0.48 | | | | | 1.11532E-14 | ns | | | | 0.038773183 | 3a | |  |
|  |  | **NCU06724** | | | **glutamine-1** | | | **0.48** | | | | | **6.72569E-18** | **ns** | | | | **0.000618487** | **4a** | |  |
|  |  | NCU04292 | | | branched-chain-amino-acid aminotransferase | | | 0.44 | | | | | 2.85537E-08 | ns | | | | 2.98019E-09 | 5a | |  |
|  |  | NCU07608 | | | pentose phosphate metabolism-3 | | | 0.44 | | | | | 4.48606E-11 | ns | | | | ns | 6a | |  |
|  |  | NCU02004 | | | phosphoserine phosphatase | | | 0.43 | | | | | 2.44333E-15 | ns | | | | ns | 7a | |  |
|  |  | NCU07725 | | | chorismate mutase | | | 0.41 | | | | | 1.21477E-10 | ns | | | | ns | 8a | |  |
|  |  | NCU08216 | | | cystathionine beta-synthase | | | 0.39 | | | | | 4.64984E-13 | ns | | | | 0.002131163 | 9a | |  |
|  |  | NCU02505 | | | succinate | | | 0.37 | | | | | 5.69527E-33 | 1.16 | | | | ns | 10a | |  |
|  |  | NCU02479 | | | glutamine synthetase | | | 0.26 | | | | | 1.84704E-33 | 0.82 | | | | ns | 11a | |  |
|  |  | NCU02785 | | | aromatic-8 | | | 0.20 | | | | | 4.50433E-24 | 0.42 | | | | ns | 12a | |  |
|  |  | **NCU00554** | | | **homoserine-1** | | | **0.19** | | | | | **9.32897E-23** | **0.42** | | | | **ns** | **13a** | |  |
|  |  | NCU02274 | | | formate | | | 0.17 | | | | | 1.43926E-26 | 1.56 | | | | ns | 14a | |  |
|  |  | NCU01439 | | | D-3-phosphoglycerate dehydrogenase 1 | | | 0.10 | | | | | 2.5872E-28 | 0.21 | | | | ns | 15a | |  |
| ***∆wc-1* specific** | | NCU05093 | | | cystathionine gamma-synthase | | | ns | | | | | ns | 28.37 | | | | 3.03124E-09 | 1b | |  |
|  |  | NCU03761 | | | hypothetical protein | | | 3.04 | | | | | 2.03651E-21 | 9.41 | | | | 3.88952E-67 | 2b | |  |
|  |  | NCU01870 | | | Eno3 protein | | | ns | | | | | ns | 3.43 | | | | 6.01917E-06 | 3b | |  |
|  |  | NCU02482 | | | tricarboxylic acid-2 | | | 0.59 | | | | | 8.36369E-07 | 2.89 | | | | 1.30995E-09 | 4b | |  |
|  |  | **NCU02333** | | | **arginase-1** | | | **ns** | | | | | **ns** | **2.24** | | | | **1.06577E-12** | **5b** | |  |
|  | | NCU04280 | | | aconitate hydratase | | | 0.55 | | | | | 9.00499E-11 | 0.25 | | | | 1.17999E-18 | 6b | |  |
| **Fatty acid metabolism** |  | | | |  | | | | |  | |  | | |  | |  | | |  |  |
| ***wt* specific** | *NCU06905* | | | | *tetrahydroxynaphthalene reductase-2* | | | | | 64.91 | | 6.22898E-29 | | | ns | | ns | | | 1a |  |
| ***∆wc-1* specific** | *NCU09732* | | | | *acetyl-CoA acetyltransferase* | | | | | 4.78 | | 3.85062E-53 | | | 13.22 | | 1.80206E-83 | | | 1b |  |
|  | *NCU04796* | | | | *3-ketoacyl-CoA thiolase* | | | | | 2.56 | | 4.87813E-29 | | | 6.11 | | 9.49553E-48 | | | 2b |  |
|  | *NCU04462* | | | | *3-ketoacyl-CoA reductase* | | | | | ns | | ns | | | 0.48 | | 2.29782E-05 | | | 3b |  |
|  | *NCU08535* | | | | *acetyl-CoA carboxylase* | | | | | 0.66 | | 0.015656731 | | | 0.15 | | 2.75695E-14 | | | 4b |  |
|  | *NCU02209* | | | | *delta-12 fatty acid desaturase* | | | | | 0.34 | | 1.50063E-31 | | | 0.12 | | 4.11109E-26 | | | 5b |  |
|  | *NCU07307* | | | | *cel-2; chain elongation-2* | | | | | 0.42 | | 3.57317E-12 | | | 0.10 | | 5.72319E-25 | | | 6b |  |
|  | *NCU07308* | | | | *cel-1; chain elongation-1* | | | | | 0.32 | | 1.8954E-24 | | | 0.09 | | 4.39545E-22 | | | 7b |  |
