# Supplementary material for: Adaptation to glucose starvation is associated with molecular reorganization of the circadian clock in Neurospora crassa
Source: eLife. 2023 Jan 10;12:e79765. doi: 10.7554/eLife.79765 (PMC9831608; doi:10.7554/eLife.79765)
Supplement: Figure 4—source data 5. — Experimental procedures were performed as described in Figure 4—figure supplement 7. (n=4, two sample t-test). [file elife-79765-fig4-data5.docx]

**Figure 4 – Source data 5**

Comparison of the results from RNA-seq and the experimental validation of the chosen genes with qPCR. Experimental procedures were performed as described in Figure 4 – Figure supplement 7. (n=4, two sample t-test)

| **ID** | **Name** | **Sample** | **Fold change in RNAseq** | **p_adj_** | **Fold change in qPCR** | **p** |
| --- | --- | --- | --- | --- | --- | --- |
| NCU02333 | arginase (*aga-1*) | *wt* (0.01%/2%) | 0.823393 | 0.0576 | 0.6073 | 0.0271 |
|  |  | *∆wc-1* (0.01%/2%) | 2.240553 | 1.0657E-12 | 1.5609 | 0.0584 |
|  |  | *∆wc-1*/*wt* (2%) | 0.710738 | 0.0017 | 0.4268 | 0.0045 |
|  |  | *∆wc-1*/*wt* (0.01%) | 1.889453 | 1.2669E-09 | 1.0972 | 0.5413 |
| NCU06724 | glutamine synthetase (*gln-1*) | *wt* (0.01%/2%) | 0.475988 | 6.7256E-18 | 0.415391 | 0.0043 |
|  |  | *∆wc-1* (0.01%/2%) | 0.761916 | 0.1045 | 0.527959 | 0.0483 |
|  |  | *∆wc-1*/*wt* (2%) | 0.507442 | 4.5308E-07 | 0.313942 | 0.0028 |
|  |  | *∆wc-1*/*wt* (0.01%) | 0.793371 | 0.0394 | 0.399018 | 0.0174 |
| NCU00461 | NAD-specific glutamate dehydrogenase (*gdh-1*) | *wt* (0.01%/2%) | 3.321658 | 2.1057E-16 | 3.531126 | 0.0331 |
|  |  | *∆wc-1* (0.01%/2%) | 7.186952 | 1.8685E-68 | 7.139931 | 0.0197 |
|  |  | *∆wc-1*/*wt* (2%) | 0.75745 | 0.0225 | 0.563023 | 0.0054 |
|  |  | *∆wc-1*/*wt* (0.01%) | 1.602065 | 0.0057 | 1.138431 | 0.6459 |
| NCU07325 | conidiation-specific protein 10 (*con-10*) | *wt* (0.01%/2%) | 70.05014 | 4.4385E-121 | 72.287593 | 0.0088 |
|  |  | *∆wc-1* (0.01%/2%) | 13.54775 | 6.5037E-18 | 10.24047 | 2.2E-05 |
|  |  | *∆wc-1*/*wt* (2%) | 0.913622 | 0.8203 | 0.568599 | 0.0831 |
|  |  | *∆wc-1*/*wt* (0.01%) | 0.172141 | 3.5345E-20 | 0.080549 | 0.0107 |
| NCU08726 | conidial development protein fluffy (*fl*) | *wt* (0.01%/2%) | 1.580692 | 1.4253E-05 | 1.56442 | 0.0724 |
|  |  | *∆wc-1* (0.01%/2%) | 4.56848 | 1.1494E-14 | 3.295842 | 0.0036 |
|  |  | *∆wc-1*/*wt* (2%) | 1.034767 | 0.8160 | 0.622065 | 0.0317 |
|  |  | *∆wc-1*/*wt* (0.01%) | 2.915692 | 5.8028E-08 | 1.310535 | 0.1231 |
| NCU10045 | pectinesterase (*pect*) | *wt* (0.01%/2%) | 1.114059 | 0.5946 | 0.991918 | 0.9617 |
|  |  | *∆wc-1* (0.01%/2%) | 0.486009 | 3.8996E-13 | 0.352179 | 0.0075 |
|  |  | *∆wc-1*/*wt* (2%) | 0.830954 | 0.2165 | 0.537041 | 0.0215 |
|  |  | *∆wc-1*/*wt* (0.01%) | 0.354272 | 1.3201E-13 | 0.190675 | 0.0059 |
